# Functional group tolerant iron-catalyzed cyclotrimerization of terminal and internal alkynes

**DOI:** 10.1038/s42004-025-01859-7

**Published:** 2025-12-27

**Authors:** Benedict Klinnert, Bernd Plietker

**Affiliations:** Professur für Organische Chemie I, Fakultät Chemie und Lebensmittelchemie, TU Dresden, Dresden, DE Germany

**Keywords:** Catalysis, Homogeneous catalysis, Synthetic chemistry methodology

## Abstract

Arenes play a crucial role in various fields of chemistry. Since Kekulé proposed the molecular structure of benzene in 1865, chemists have been working to develop methods for preparing functionalized arenes. Most of these methods focus on introducing functional groups through substitution reactions (electrophilic aromatic substitution, cross-coupling, C-H activation) using aromatic starting materials. In contrast, the formal [2 + 2 + 2] cycloaddition of three different alkyne moieties allows for the introduction of functional groups before synthesizing the aromatic core. Although straightforward, this strategy faces challenges, including the inherent reactivity of heteroatom-substituted alkynes, restrictions on the substitution patterns of the alkynes, and the use of noble metal-based catalysts. Herein, we report an iron-catalyzed cyclotrimerization of internal and terminal alkynes to provide densely functionalized aromatic products. The method accommodates alkoxy-, amido-, and even boryl alkynes, yielding the corresponding aryl ethers, amides, or boronic acids in up to quantitative yields at moderate temperatures within minutes.

## Abstract (web summary)

Arenes and cyclohexatrienes are pivotal in chemistry, yet synthesizing functionalized variants remains challenging. Here, the authors introduce an iron-catalyzed cyclotrimerization method for internal and terminal heteroatom-substituted alkynes, achieving efficient synthesis of densely functionalized aromatic products.

## Introduction

In 1931, Hückel defined rules for aromaticity in hydrocarbon structures^1–3^. According to these rules, aromatic hydrocarbons are planar cyclic molecules that feature alternating single and double bonds between atoms, with [4n + 2]-π-electrons. This specific combination of planarity and alternating π-electrons gives aromatic compounds unique chemical properties, which have found widespread applications across various fields of chemistry^4–6^. The ongoing development of tailored synthetic methods to create these molecules highlights their significance in organic chemistry^7–23^.

**Fig. 1:**
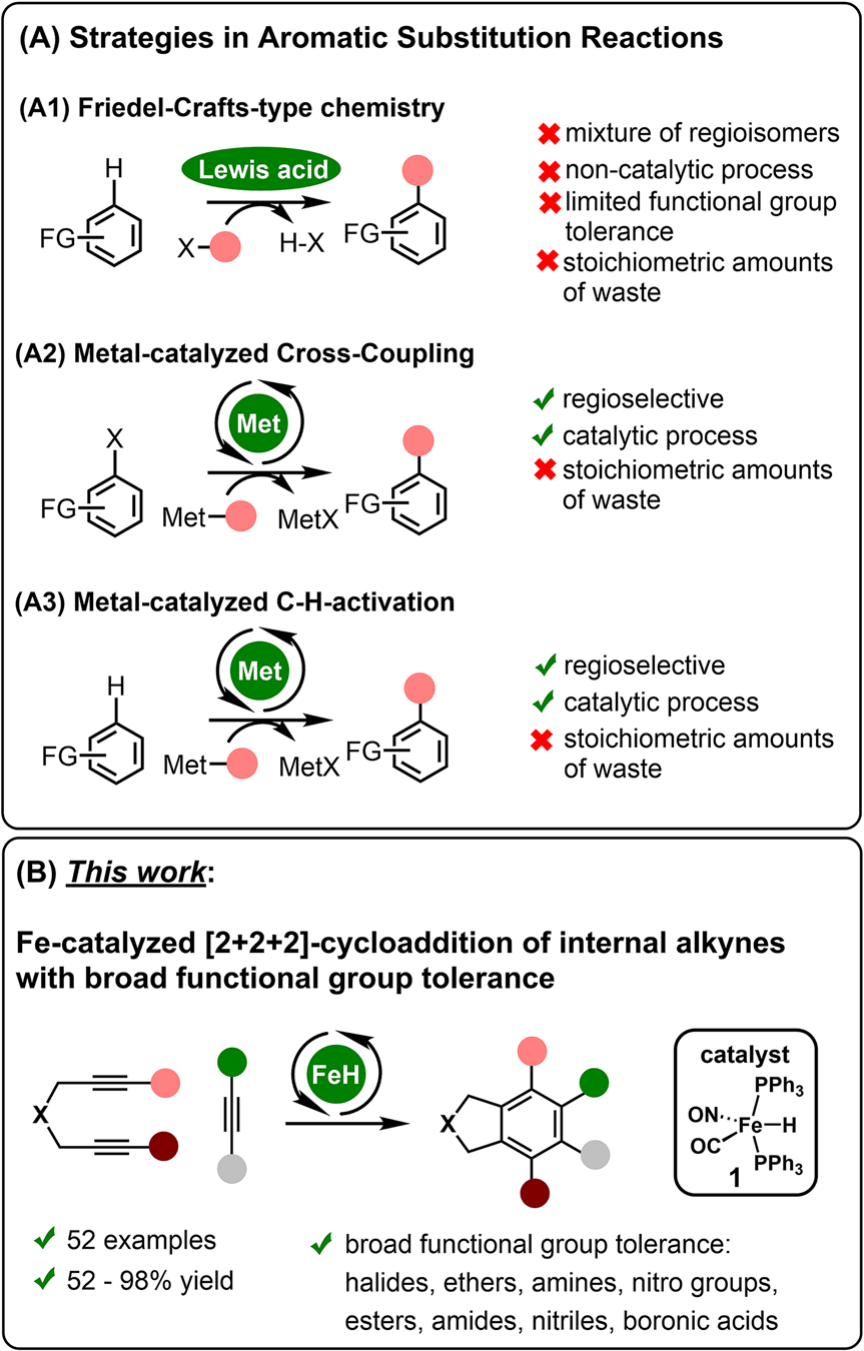
Strategies for the synthesis of functional arenes. **A** Aromatic substitution versus (**B**) alkyne trimerization.

Classical methods for synthesizing aromatic compounds typically involve the use of pre-existing aromatic starting materials through substitution reactions ((A1), Fig. 1)^7–10^. These methods often require high temperatures and reactive electrophiles or nucleophiles, resulting in considerable waste due to the stoichiometric amounts used. This has led to increased interest in developing milder aromatic derivatization strategies that primarily utilize transition metal-catalyzed cross-coupling technologies ((A2), Fig. 1)^11–14^ or C-H activations ((A3), Fig. 1)^15–19^. An alternative approach, first discovered in 1866 by Berthelot, involves the cyclotrimerization of alkynes^20–24^. Berthelot reported that using high temperatures could yield aromatic products^24^. Nearly 80 years later, reports emerged on nickel-catalyzed trimerizations of alkynes, establishing a foundation for systematically evaluating alternative transition metal catalysts^25^. To date, a variety of catalytic processes have been developed, primarily involving nickel, palladium, cobalt, and rhodium complexes^20–23^.

Iron is one of the most abundant metals in the Earth’s upper crust^26^. Therefore, catalysis using Fe-complexes offers both economic and ecological advantages. Although Fe-based catalytic processes have been reported as early as 1960, research in this area has only recently gained significant attention^27–30^. Most of reported Fe-catalyzed transformations employ primarily monosubstituted alkynes^30–40^. In contrast, internal alkynes or highly reactive substrates such as alkoxy-, amido-, or borylalkynes are underrepresented or not tolerated^30–45^. In this report, we present the results of an in-depth study on the use of defined Fe-H complexes as catalysts in [2 + 2 + 2] trimerizations of alkynes. This method is characterized by an exceptionally broad application range and mild reaction conditions ((B), Fig. 1). Both terminal and internal alkynes can be reacted to produce even hexasubstituted arenes in good to quantitative yields. Notably, this method demonstrates an unusual tolerance for functional groups in the context of Fe-catalyzed alkyne cyclotrimerizations. This allows for the use of heteroatom-substituted alkynes to yield the corresponding aryl ethers, amides, or boronic acid derivatives.

## Results and Discussion

**Fig. 2:**
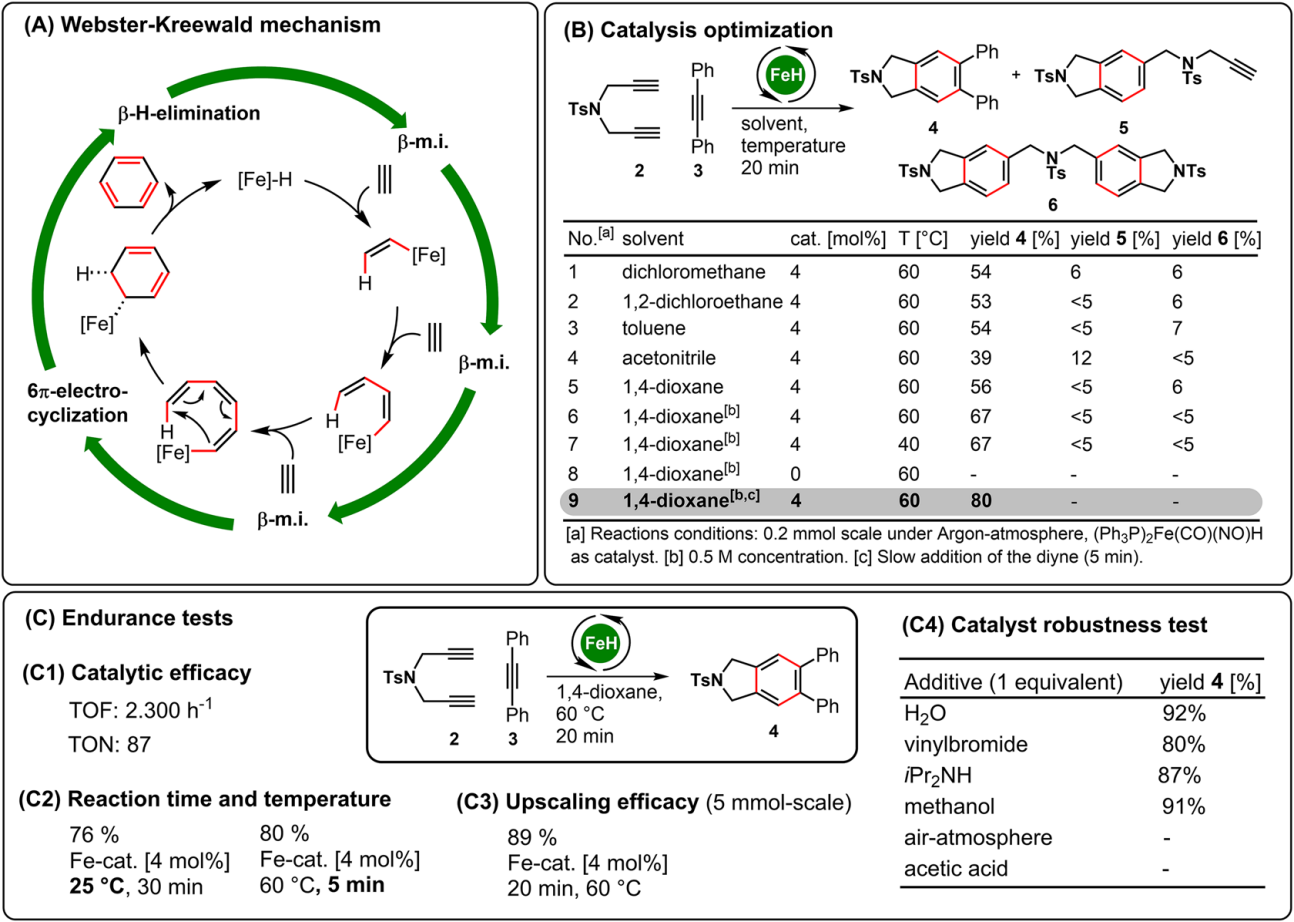
Mechanistic analogies, reaction development and robustness test. **A** Webster-Krewald mechanism for Fe-catalyzed [2 + 2 + 2]-cycloaddition; (**B**) temperature and solvent effects in [(Ph_3_P)_2_Fe(CO)(NO)H-catalyzed [2 + 2 + 2]-cycloaddition; (**C**) Upscaling, catalyst robustness and productivity testing.

In 1983 Roustan described for the first time the synthesis of the structurally defined and bench-stable bisphosphino-Fe(NO)-H-complex **1** starting from the readily available salt Na[Fe(CO)_3_(NO)]^46^. The catalytic portfolio of Fe-NO-complexes that are accessible from this prototype of ferrate are part of a larger research project in our group^47–51^. In a very recent landmark study, Webster and Krewald reported an in-situ-generated Fe-H-complex as a potent catalysts for the [2 + 2 + 2]-cycloaddition of alkynes^37^. Based on both experimental but also quantumchemical results a mechanistic sequence of three consecutive β-migratory insertions (i.e. hydroferration, and vinylferration), a 6-π-electrocyclization and β-hydride elimination to the final arene product was proposed ((A), Fig. 2). Inspired by this intriguing work and on our previous work on borylative cyclizations we initiated a research project devoted to telescope the potential catalytic activity of complex (Ph_3_P)_2_Fe(CO)(NO)H (**1**) in [2 + 2 + 2]-cycloisomerizations of alkynes using diyne **2** and 1,2-diphenylacetylene (**3**) as starting materials ((B), Fig. 2). While an initial screening revealed that most of the Fe-H-complexes of the general type (P,P´)Fe(CO)(NO)H did not catalyze the transformation (Ph_3_P)_2_Fe(CO)(NO)H (**1**) turned out to be a very active catalyst for the [2 + 2 + 2]-cycloaddition. After careful optimizations (Supplementary tables 1–7), we found 4 mol% of the catalyst **1**, 1,4-dioxane as a solvent and a substrate concentration of 0.5 mol/l at 60 °C to provide the desired cross-cycloaddition product **4** in good yields. Slow addition of the diyne **2** proved beneficial and led to formation of **4** in 80% yield after only 5 min. In the absence of catalyst **1** no conversion was obtained.

The catalytic system needed to undergo several “endurance tests” ((C), Fig. 2). The turnover frequencies (supplementary table 8, supplementary fig. 1) and turnover numbers (supplementary table 9, supplementary fig. 2) demonstrated stability across different catalyst concentrations, highlighting the catalyst’s reliability under the specified conditions ((C1), Fig. 2). When the reaction temperature was decreased to 25 °C, product **4** was formed with a yield of 74% after an extended reaction time of 30 min ((C2), Fig. 2). This indicates that scaling up the process is feasible ((C3), Fig. 2). Remarkably, catalyst loadings as low as 0.5 mol% can be utilized. In the context of complex organic synthesis, the tolerance of functional groups is crucial. To prepare for a larger screening of the substrate scope (as shown in Scheme 1), a robustness test of the catalyst was conducted ((C4), Fig. 2). This test involved adding competing and reactive functional molecules to the standard reaction. The catalyst demonstrated tolerance to a variety of these additives; however, it showed sensitivity to air and acidic conditions.

**Fig. 3:**
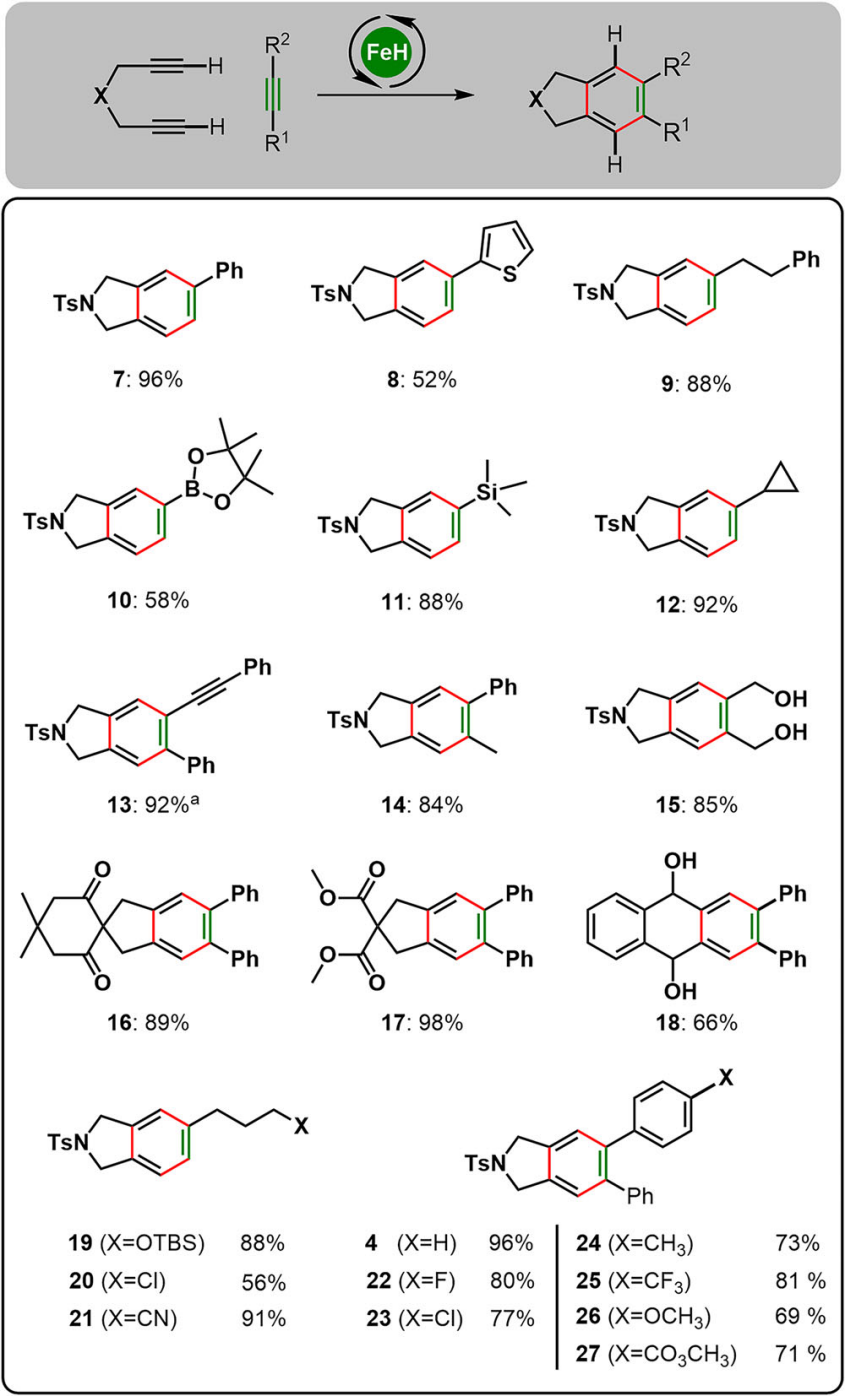
[2 + 2 + 2]-Cycloaddition of terminal diynes – scope and limitation. General conditions: Diyne (0.5 mmol), alkyne (10 eq.), catalyst 1 (4 mol%), 1,4-dioxane (0.5 M), 60 °C, 5 min. ^a^1,4-Dioxane (0.3 M).

**Fig. 4:**
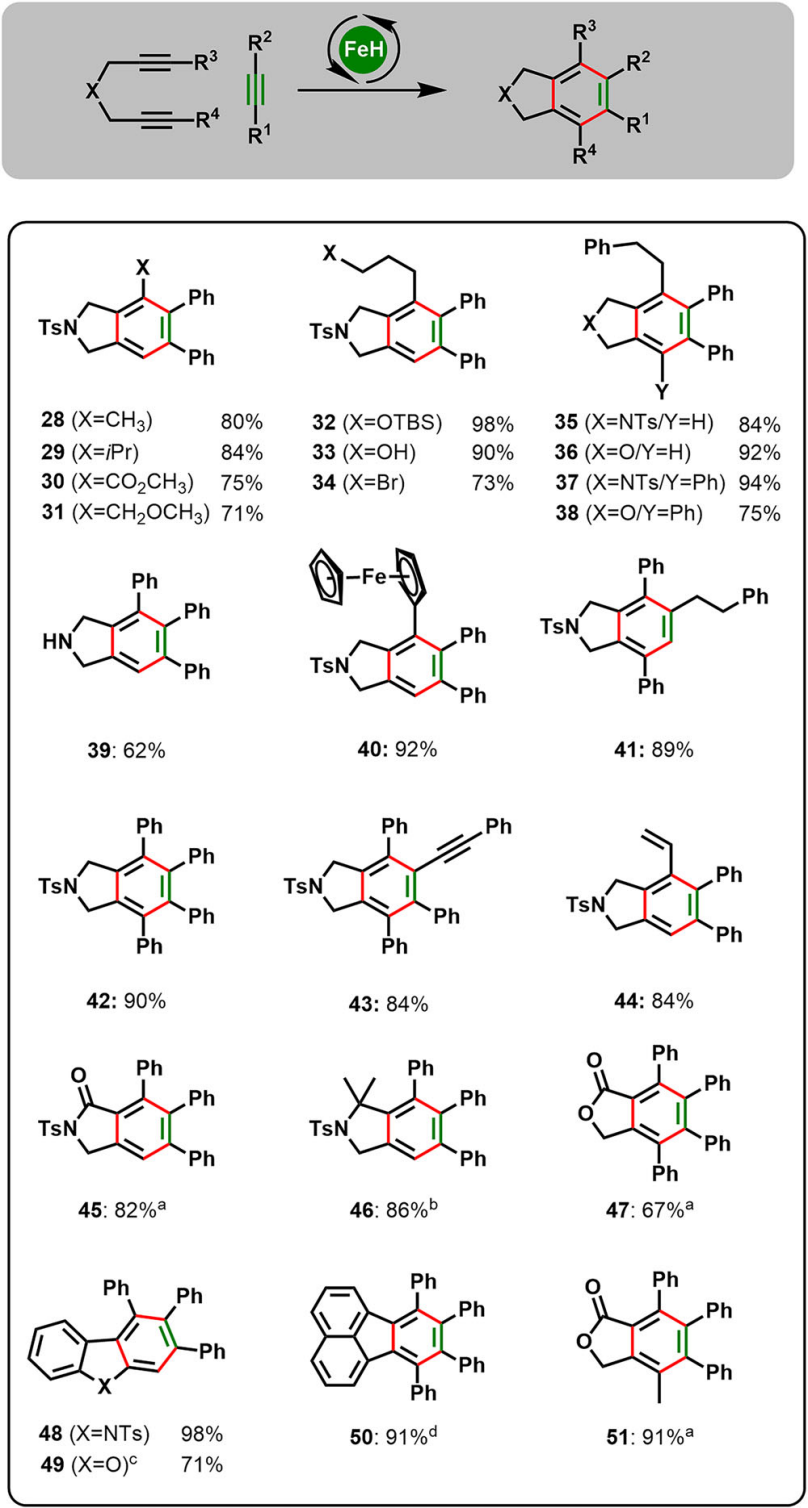
[2 + 2 + 2]-Cycloaddition of internal diynes – scope and limitation. General conditions: Diyne (0.5 mmol), alkyne (10 eq.), catalyst **1** (4 mol%), 1,4-dioxane (0.5 M), 60 °C, 5-60 min. ^a^Diyne (0.2 mmol). ^b^Catalyst **1** (10 mol%). ^c^Diyne (0.1 mmol). ^d^1,4-Dioxane (0.3 M).

Based on the results obtained, we performed a systematic screening of the substrate scope, as illustrated in Figs. 3, 4. Using terminal diyneamides, we successfully transformed a variety of mono- and disubstituted alkynes into their corresponding aromatic systems (**7**–**15, 19**–**27**). The catalyst demonstrated high tolerance to a diverse range of functional groups, including silyl ethers (**19**), halides (**20,** **22,** **23,** **25**), ethers (**26**), esters (**17,** **27**), alcohols (**15**) and secondary amines (**39**).

In contrast to reported iron-based catalysts^30,34,35,42,43^, (Ph_3_P)_2_Fe(CO)(NO)H (**1**) shows a clear preference for alkynes over nitriles in [2 + 2 + 2] cycloadditions (**21**), formation of the corresponding pyridine derivatives through incorporation of nitriles was not observed. To our delight, the high chemoselectivity of the Fe-catalyst **1** allows to use reactive and labile alkynes, such as alkynylboronates, -silanes, -ethers, and -amides. While these substrates do offer several competing reactivities, the alkyne moiety proved to be the only reactive structural entity yielding synthetically relevant products such as arylboronates (**10**), arylsilanes (**11**), dibenzofurans (**49**), and even carbazoles (**48**) (see Figs. 3 and 4).

Higher substituted diynes can be employed to generate densely substituted aromatic products in good to excellent yields (Fig. 4). Aromatic but also aliphatic substituents at the alkyne do not hamper the reaction outcome. Functional groups like esters, ethers, halides, or even unprotected alcohols and amines are tolerated (Fig. 4). Different linker units like ethers, 1,3-dicarbonyl derivatives, esters, lactams, or aromatics can be employed (Figs. 3 and 4). Importantly, even alkynyl amide or ether linkages are compatible to furnish carbazole **48** or dibenzofuran **49** (Fig. 4). To the best of our knowledge, these are the first reports on the use of yneamides or ethers in Fe-catalyzed [2 + 2 + 2]-cycloadditions, underlining the potential of well-defined Fe-complexes in catalysis.

**Fig. 5:**
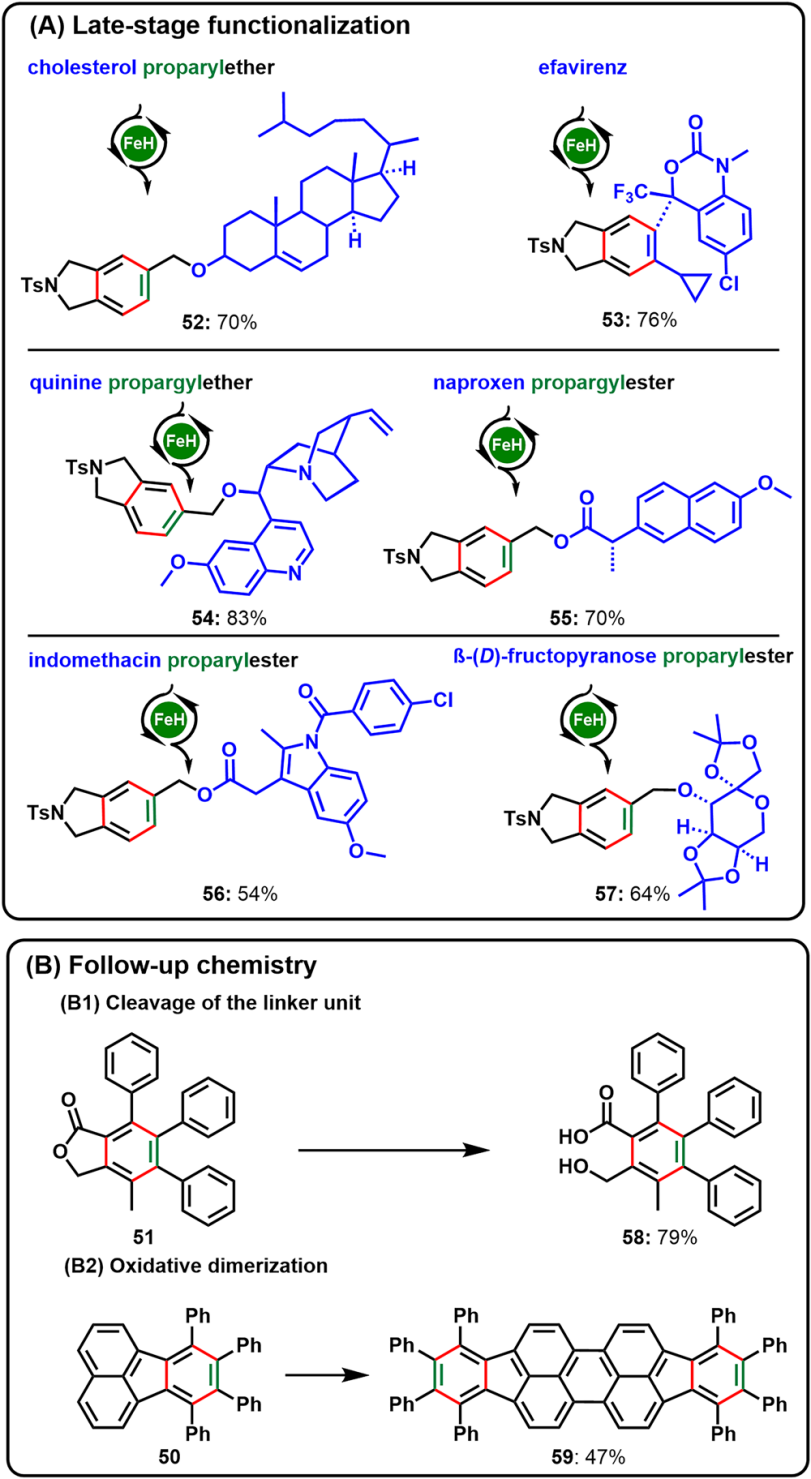
Applications of Fe-catalyzed [2 + 2 + 2]-cycloadditions. **A** Late-stage modifications, General conditions: Diyne (0.5–0.1 mmol), alkyne (3-10 eq.), catalyst **1** (4-10 mol%), 1,4-dioxane (0.5 M), 60 °C, 5–20 min. ^a^1,4-Dioxane (0.17 M).; **B** Follow-up chemistry: (B1) cleavable linkers, General conditions: Lactone **51** (0.3 mmol), NaOH (10% aq. solution, 16.5 eq.), THF (0.03 M), 65 °C, 4.5 h; and (B2) synthesis of carbon-rich materials through [2 + 2 + 2]-cycloaddition/oxidative dimerization, General conditions: Fluoranthene **50** (0.2 mmol), FeCl_3_ (12 eq.), DCM/CH_3_NO_2_ (13/1, 0.02 M), 25 °C, 20 min.

Knowing the exceptional broad functional group tolerance we employed the catalytic transformation to a late-stage functionalization of polyfunctional natural product derivatives like cholesterol, efavirenz, quinine, naproxen, indomethacin, or even a fructopyranose ((A), Fig. 5). In all of these cases good to excellent yields of the corresponding [2 + 2 + 2]-cycloaddition product were observed, functional groups present in the complex scaffolds were not affected.

Not only the cycloaddition itselfs allows for skeletal variation on a late-stage but the obtained products themselves pave the way for interesting follow-up chemistry ((B), Fig. 5). Cleavage of the linker moiety, e.g. via saponification yields a hexasubstituted benzoic acid derivative **58** in high yields that would not be accessible using established stepwise functionalization processes. Moreover, octaphenyl-diindenoperylene **50**, accessible in two steps from 1,8-dibromonaphtalene, is obtained using e.g., Fe-mediated oxidative dehydrogenative cross-coupling of **59**^52^. Such materials are discussed as promising lead structures in organic semiconductor technologies^53^.

**Fig. 6:**
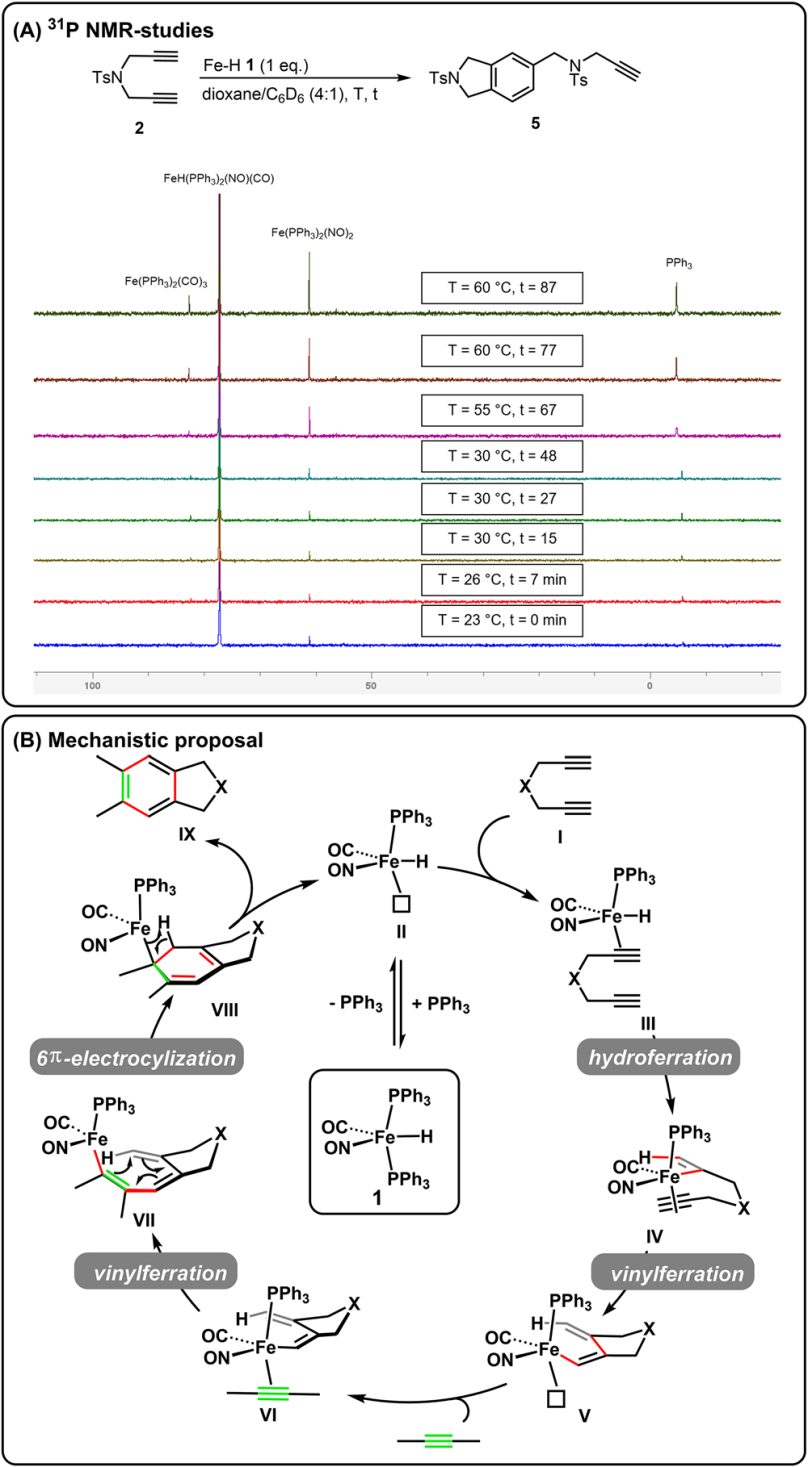
Mechanistic investigations and proposal. **A** ^31^P NMR studies; (**B**) Proposed mechanism for Fe-catalyzed [2 + 2 + 2]-cycloaddition.

Several mechanistic models for metal-catalyzed formal [2 + 2 + 2]-cycloadditions are discussed in literature^20–23,30,54^. In 2021 Krewald and Webster reported the in-situ preparation of a highly reactive Fe-H-complex and its application in catalytic [2 + 2 + 2]-cycloadditions^37^. Based on experimental evidence and supported by quantumchemical calculations they proposed a mechanism in which the catalysis is initiated by a hydroferration of one of the alkyne units. This mechanistic model inspired us to perform control experiments (details are found in chapter 3, supporting information). In order to get deeper insights, we attempted to prepare the deuterated Fe-catalyst D-**1**, however, due to deuterium scrambling a mixture of **1** and D-**1** (60/40) was obtained. Despite this we set out to analyze the conversion rate of the reaction catalyzed by pure **1** versus a 60/40-mixture of **1**/D-**1**. Products were analyzed not only for conversion but also for deuterium incorporation^55^. Although no deuterium was detectable in the final products, a significant kinetic isotope effect was visible (supplementary table 10, supplementary fig. 3), however, due to the fact that we were yet not able to prepare D-**1** in pure form, an exact ratio was not calculated. Moreover, a very short induction period was observed indicating that a catalyst-activating upstream-process is necessary prior to catalytic turnover. In previous reports, we showed that phosphine dissociation and subsequent phosphine association in homoleptic Fe-complex **1** allows the synthesis of P,P´-heteroleptic Fe-complexes^49^. To test whether the dissociation of one of the two phosphine ligands might explain the observed short induction period, **1** was heated with stoichiometric amounts of diyne **9** and the course of the reaction was followed by ^31^P NMR-spectroscopy ((A), Fig. 6)^56^. Formation of free PPh_3_ was observed even at room temperature. Upon increasing the temperature, formation of PPh_3_ increased but also formation of two new complexes, i.e., (Ph_3_P)_2_Fe(CO)_3_ and (Ph_3_P)_2_Fe(NO)_2_, was observed. While (Ph_3_P)_2_Fe(NO)_2_ does not show any catalytic activity, its formation is indicative for a dissociation of one phosphine ligand from complex **1** to the tentatively assigned unsaturated species (Ph_3_P)Fe(CO)(NO)H (**II**)). Further support that an upstream phosphine dissociation process is necessary for catalyst activation came from an experiment in which we added additional PPh_3_ to the catalytic system^57^. This led to full catalyst deactivation, no conversion of the starting material was observed.

These results led us to propose a preliminary mechanistic model ((B), Fig. 6), in which phosphine dissociation of **1** to **II** represents the initial precatalyst-activating step. This complex can coordinate to one out of the three alkyne groups present in the reaction mixture. Coordination to the diyne **I** results in formation of **III** which upon hydroferration reacts to the vinyl-Fe-complex **IV**. Intramolecular vinylferration results in the formation of vinyl-Fe-species **V**, which upon coordination to the external alkyne yields complex **VI**. Final intermolecular vinylferration to **VII** and subsequent 6-π-electrocyclization to **VIII** would set the stage for a β-H-elimination/aromatization to regenerate the active catalyst **II** with formation of the observed aromatic product **IX**.

## Methods

### General experimental procedure for Fe-catalyzed [2 + 2 + 2]-cycloaddition

In a heatgun-dried (2 min) 10 mL-screw cap Schlenk tube, the catalyst FeH(CO)(NO)(PPh_3_)_2_ **1** (4 mol%) and diphenylacetylene **3** (10.0 eq.) were dissolved in anhydrous 1,4-dioxane (*c* = 1.00 mol/L). The diyne in 1,4-dioxane (*c* = 1.00 mol/L) was slowly added at 60 °C. The reaction mixture was stirred for 5 min at 60 °C. The solvent was removed under reduced pressure and the crude products were purified by silica gel column chromatography (iso-hexanes/ethyl acetate mixtures).

## Conclusion

In summary, we present a Fe-based catalytic system for a broadly applicable alkyne trimerization toward densely functionalized arene products. Short reaction times and moderate temperatures warrant the overall applicability of the process. The catalyst is accessible in two synthetic operations from readily available Fe(CO)_5_ and can be handled without special precautions, pre-activation of the catalyst by addition of sacrificial reductands is not necessary. An exceptional high functional group tolerance is demonstrated giving the desired product in good to quantitative yield. Late-stage diversifications of biological relevant molecules but also follow-up chemistry underlines the synthetic usefulness of the presented catalytic system. In the field of alkyne [2 + 2 + 2]-cycloaddition the present catalyst shows the broadest functional group tolerance and thus paves the way for a straightforward access to synthetically relevant arene building blocks.

## Supplementary information

Supplemental material

## Data Availability

The authors confirm that all relevant data are included in the paper and/or its supplementary information files.
