# Supplementary material for: Functional group tolerant iron-catalyzed cyclotrimerization of terminal and internal alkynes
Source: Commun Chem. 2025 Dec 27;8:410. doi: 10.1038/s42004-025-01859-7 (PMC12743653; doi:10.1038/s42004-025-01859-7)
Supplement: Supplementary file 1 — Supplemental material [file 42004_2025_1859_MOESM1_ESM.pdf]

Functional group tolerant iron-catalyzed cyclotrimerization of terminal and internal alkynes

Benedict Klinnert,[a] and Bernd Plietker*[a]

[a] M. Sc. Benedict Klinnert, Prof. Dr. Bernd Plietker

Professur für Organische Chemie I

Fakultät Chemie und Lebensmittelchemie, TU Dresden

Bergstraße 66

DE-01069 Dresden, Germany.

bernd.plietker@tu-dresden.de

Contents

1. General Remarks 5

2. Reaction Optimisation 6

2.1 Catalyst Screening 6

2.2 Solvent Screening 6

2.3 Concentration Screening 7

2.4 Temperature Screening 7

2.5 Catalyst Loading 8

2.6 Time Screening 8

2.7 Equivalents Diphenylacetylene 9

3. TON-, TOF-Data and mechanistic studies 10

3.1 TOF 10

3.2 TON 11

3.3 Kinetic isotope effect 12

3.4 Control experiments 13

3.5 ^{31}P NMR-study 14

4. Procedures 15

4.1 Catalysis Products 15

4.1.1 Preparation of 5,6-Diphenyl-2-tosylisoindoline **4** 15

4.1.2 Preparation of 5-phenyl-2-tosylisoindoline **7** ... 16
4.1.3 Preparation of 5-(Thiophen-2-yl)-2-tosylisoindoline **8** ... 16
4.1.4 Preparation of 5-Phenethyl-2-tosylisoindoline **9** ... 17
4.1.5 Preparation of 5-(4,4,5,5-Tetramethyl-1,3,2-dioxaborolan-2-yl)-2-tosylisoindoline **10** ... 18
4.1.6 Preparation of 2-Tosyl-5-(trimethylsilyl)isoindoline **11** ... 18
4.1.7 Preparation of 5-Cyclopropyl-2-tosylisoindoline **12** ... 19
4.1.8 Preparation of 5-Phenyl-6-(phenylethynyl)-2-tosylisoindoline **13** ... 20
4.1.9 Preparation of 5-Methyl-6-phenyl-2-tosylisoindoline **14** ... 20
4.1.10 Preparation of (2-Tosylisoindoline-5,6-diyl)dimethanol **15** ... 21
4.1.11 Preparation of 4,4-Dimethyl-5',6'-diphenyl-1',3'-dihydrospiro[cyclohexane-1,2'-indene]-2,6-dione **16** ... 22
4.1.12 Preparation of Dimethyl 5,6-diphenyl-1,3-dihydro-2*H*-indene-2,2-dicarboxylate **17** ... 22
4.1.13 Preparation of 2,3-Diphenyl-9,10-dihydroanthracene-9,10-diol **18** ... 23
4.1.14 Preparation of 5-(3-((*tert*-Butyldimethylsilyl)oxy)propyl)-2-tosylisoindoline **19** ... 24
4.1.15 Preparation of 5-(3-Chloropropyl)-2-tosylisoindoline **20** ... 24
4.1.16 Preparation of 4-(2-Tosylisoindolin-5-yl)butanenitrile **21** ... 25
4.1.17 Preparation of 5-(4-Fluorophenyl)-6-phenyl-2-tosylisoindoline **22** ... 25
4.1.18 Preparation of 5-(4-Chlorophenyl)-6-phenyl-2-tosylisoindoline **23** ... 26
4.1.19 Preparation of 5-Phenyl-6-(*p*-tolyl)-2-tosylisoindoline **24** ... 27
4.1.20 Preparation of 5-Phenyl-2-tosyl-6-(4-(trifluoromethyl)phenyl)isoindoline **25** ... 27
4.1.21 Preparation of 5-(4-Methoxyphenyl)-6-phenyl-2-tosylisoindoline **26** ... 28
4.1.22 Preparation of methyl 4-(6-phenyl-2-tosylisoindolin-5-yl)benzoate **27** ... 29
4.1.23 Preparation of 4-methyl-5,6-diphenyl-2-tosylisoindoline **28** ... 29
4.1.24 Preparation of 4-isopropyl-5,6-diphenyl-2-tosylisoindoline **29** ... 30
4.1.25 Preparation of methyl 5,6-diphenyl-2-tosylisoindoline-4-carboxylate **30** ... 31
4.1.26 Preparation of 4-(methoxymethyl)-5,6-diphenyl-2-tosylisoindoline **31** ... 31
4.1.27 Preparation of 4-(3-((tert-butyldimethylsilyl)oxy)propyl)-5,6-diphenyl-2-tosylisoindoline **32** ... 32
4.1.28 Preparation of 3-(5,6-diphenyl-2-tosylisoindolin-4-yl)propan-1-ol **33** ... 33
4.1.29 Preparation of 4-(3-bromopropyl)-5,6-diphenyl-2-tosylisoindoline **34** ... 33
4.1.30 Preparation of 4-phenethyl-5,6-diphenyl-2-tosylisoindoline **35** ... 34
4.1.31 Preparation of 4-phenethyl-5,6-diphenyl-1,3-dihydroisobenzofuran **36** ... 35
4.1.32 Preparation of 4-phenethyl-5,6,7-triphenyl-2-tosylisoindoline **37** ... 35
4.1.33 Preparation of 4-phenethyl-5,6,7-triphenyl-1,3-dihydroisobenzofuran **38** ... 36
4.1.34 Preparation of 4,5,6-triphenylisoindoline **39** ... 37
4.1.35 Preparation of 4-ferrocene-5,6-diphenyl-2-tosylisoindoline **40** ... 37
4.1.36 Preparation of 5-phenethyl-4,7-diphenyl-2-tosylisoindoline **41** ... 38

4.1.37 Preparation of 4,5,6,7-tetraphenyl-2-tosylisoindoline **42**.. 39

4.1.38 Preparation of 4,5,7-triphenyl-6-(phenylethynyl)-2-tosylisoindoline **43** 40

4.1.39 Preparation of 5,6-diphenyl-2-tosyl-4-vinylisoindoline **44**.. 40

4.1.40 Preparation of 5,6,7-triphenyl-2-tosylisoindolin-1-one **45** 41

4.1.41 Preparation of 1,1-dimethyl-5,6,7-triphenyl-2-tosylisoindoline **46** 42

4.1.42 Preparation of 4,5,6,7-tetraphenylisobenzofuran-1(3*H*)-one **47**..................................... 43

4.1.43 Preparation of 2,3,4-triphenyl-9-tosyl-9*H*-carbazole **48** 44

4.1.44 Preparation of 1,2,3-triphenyldibenzo[*b*,*d*]furan **49**.. 45

4.1.45 Preparation of 7,8,9,10-Tetraphenylfluoranthene **50**.. 46

4.1.46 Preparation of 4-Methyl-5,6,7-triphenylisobenzofuran-1(3*H*)-one **51** 46

4.1.47 Preparation of 5-((((3*S*,9*S*,10*R*,13*R*,14*S*,17*R*)-10,13-Dimethyl-17-((*R*)-6-methylheptan-2-yl)-2,3,4,7,8,9,10,11,12,13,14,15,16,17-tetradecahydro-1*H*-cyclopenta[*a*]phenanthren-3-yl)oxy)methyl)-2-tosylisoindolinete **52**... 47

4.1.48 Preparation of (*S*)-6-Chloro-4-(6-cyclopropyl-2-tosylisoindolin-5-yl)-1-methyl-4-(trifluoromethyl)-1,4-dihydro-2*H*-benzo[*d*][1,3]oxazin-2-one **53** 48

4.1.49 Preparation of (2*S*)-2-((*R*)-(6-Methoxyquinolin-4-yl)((2-tosylisoindolin-5-yl)methoxy)methyl)-5-vinylquinuclidine **54** 49

4.1.50 Preparation of (2-Tosylisoindolin-5-yl)methyl (*S*)-2-(6-methoxynaphthalen-2-yl)propanoate **55** 49

4.1.51 Preparation of (2-Tosylisoindolin-5-yl)methyl 2-(1-(4-chlorobenzoyl)-5-methoxy-2-methyl-1*H*-indol-3-yl)acetate **56**.. 50

4.1.52 Preparation of 5-((((3a'*R*,4*S*,7'*S*,7a'*R*)-2,2,2',2'-Tetramethyltetrahydrospiro[[1,3]dioxolane-4,6'-[1,3]dioxolo[4,5-c]pyran]-7'-yl)oxy)methyl)-2-tosylisoindoline **57**.. 51

4.2 Substrate Synthesis 52

4.2.1 Preparation of 4-Methyl-*N*-(prop-2-yn-1-yl)benzenesulfonamide **S7**................................. 52

4.2.2 Preparation of Methyl 4-((4-methyl-*N*-(prop-2-yn-1-yl)phenyl)sulfonamido)but-2-ynoate **S8** and *N*-(4-Methoxybut-2-yn-1-yl)-4-methyl-*N*-(prop-2-yn-1-yl)benzenesulfonamide **S9**............. 53

4.2.3 Preparation of 5,5-dimethyl-2,2-di(prop-2-yn-1-yl)cyclohexane-1,3-dione **S11** 55

4.2.4 Preparation of dimethyl 2,2-di(prop-2-yn-1-yl)malonate **S13** 56

4.2.5 Preparation of 4-methyl-*N*-(4-methylpent-2-yn-1-yl)-*N*-(prop-2-yn-1-yl)benzenesulfonamide **S16** 57

4.2.6 Preparation of 4-methyl-*N*-(5-phenylpent-2-yn-1-yl)-*N*-(prop-2-yn-1-yl)benzenesulfonamide **S19** 59

4.2.7 Preparation of *N*-(6-((tert-butyldimethylsilyl)oxy)hex-2-yn-1-yl)-4-methyl-*N*-(prop-2-yn-1-yl)benzenesulfonamide **S24** 61

4.2.8 Preparation of Ferrocene Diyne **S26** 63

4.2.9 Preparation of *N*-(but-2-yn-1-yl)-4-methyl-*N*-(prop-2-yn-1-yl)benzenesulfonamide **S27** ... 65

4.2.10 Preparation of 3-phenyl-*N*-(prop-2-yn-1-yl)prop-2-yn-1-amine **S31** 66

4.2.11 Preparation of 4-methyl-*N*-(2-methyl-4-phenylbut-3-yn-2-yl)-*N*-(prop-2-yn-1-yl)benzenesulfonamide **S35** 69

4.2.12 Preparation of 4-methyl-*N*-(pent-4-en-2-yn-1-yl)-*N*-(prop-2-yn-1-yl)benzenesulfonamide **S37** 71

4.2.13 Preparation of *N*-(6-bromohex-2-yn-1-yl)-4-methyl-*N*-(prop-2-yn-1-yl)benzenesulfonamide **S39** 73

4.2.14 Preparation of (3-((5-phenylpent-2-yn-1-yl)oxy)prop-1-yn-1-yl)benzene **S41** 75

4.2.15 Preparation of 4-methyl-*N*-(5-phenylpent-2-yn-1-yl)-*N*-(3-phenylprop-2-yn-1-yl)benzenesulfonamide **S42** 76

4.2.16 Preparation of phenylethynylbenzene derivatives **S43** – **S48** 77

4.2.17 Preparation of *N*-ethynyl-4-methyl-*N*-(2-(phenylethynyl)phenyl)benzenesulfonamide **S52** 82

4.2.18 Preparation of 1,8-bis(phenylethynyl)naphthalene **S54** 86

4.2.19 Preparation of 3,4,5,6,11,12,13,14-octaphenyldiindeno[1,2,3-cd:1',2',3'-lm]perylene **59** 87

4.2.20 Preparation of 1-(ethynyloxy)-2-(phenylethynyl)benzene **S58** 88

4.2.21 Preparation of 4-methyl-*N*,*N*-bis(3-phenylprop-2-yn-1-yl)benzenesulfonamide **S59** 90

4.2.22 Preparation of 3-phenylprop-2-yn-1-yl 3-phenylpropiolate **S61** 91

4.2.23 Preparation of 3-phenyl-*N*-(prop-2-yn-1-yl)-*N*-tosylpropiolamide **S62** 92

4.2.24 Preparation of But-2-yn-1-yl 3-phenylpropiolate **S64** 93

4.2.25 Preparation of 1,1'-(1,2-Phenylene)bis(prop-2-yn-1-ol) **S66** 94

4.2.26 Preparation of (3*S*,9*S*,10*R*,13*R*,14*S*,17*R*)-10,13-Dimethyl-17-((*R*)-6-methylheptan-2-yl)-3-(prop-2-yn-1-yloxy)-2,3,4,7,8,9,10,11,12,13,14,15,16,17-tetradecahydro-1*H*-cyclopenta[*a*]phenanthrene **S68** 95

4.2.27 Preparation of (*S*)-6-Chloro-4-(cyclopropylethynyl)-1-methyl-4-(trifluoromethyl)-1,4-dihydro-2*H*-benzo[*d*][1,3]oxazin-2-one **S70** 96

4.2.28 Preparation of (2*S*,5*R*)-2-((*R*)-(6-Methoxyquinolin-4-yl)(prop-2-yn-1-yloxy)methyl)-5-vinylquinuclidine **S72** 97

4.2.29 Preparation of Prop-2-yn-1-yl 2-(1-(4-chlorobenzoyl)-5-methoxy-2-methyl-1*H*-indol-3-yl)acetate **S74** 99

4.2.30 Preparation of Prop-2-yn-1-yl (*S*)-2-(6-methoxynaphthalen-2-yl)propanoate **S76** 100

4.2.31 Preparation of (3a'*R*,4*S*,7'*S*,7a'*R*)-2,2,2',2'-Tetramethyl-7'-(prop-2-yn-1-yloxy)tetrahydrospiro[[1,3]dioxolane-4,6'-[1,3]dioxolo[4,5-*c*]pyran] **S78** 101

4.2.32 Preparation of 4'-(Hydroxymethyl)-5'-methyl-6'-phenyl-[1,1':2',1''-terphenyl]-3'-carboxylic acid **58** 102

5. References 103

. Copies of ^{1}H, ^{13}C and ^{31}P NMR 106

7. X-ray Crystal Data 159

1. General Remarks

All reactions that are sensitive towards moisture or air were performed by application of standard Schlenk techniques under dry argon. All chemicals were purchased from Sigma Aldrich, Acros Organics, Alfa Aesar, BLDpharm or TCI and used as received. Solvents were either taken from a MBRAUN SPS (diethyl ether, dichloromethane, acetonitrile, THF, toluene) or dried using the following methods: distillation from magnesium turnings (methanol), distillation from moleculare sieve 4Å (ethyl acetate). Anhydrous, oxygen-free THF for iron catalyses was obtained by distillation from sodium using benzophenone as indicator. The catalysts **1**, **S1 – S5** were prepared as described in the literature.[1,2] NMR spectra were recorded on a Bruker Avance II 300 at 300 MHz (^{1}H NMR) and 75 MHz (^{13}C NMR) or a Bruker Avance Neo 400 at 400 MHz (^{1}H NMR) and 101 MHz (^{13}C NMR) or a Bruker Avance III 600 at 600 MHz (^{1}H NMR) and 151 MHz (^{13}C NMR). Chemical shifts are reported in ppm and were corrected with residual NMR solvent peaks. IR spectrometry was performed on a FT-IR spectrometer in ATR mode (Agilent Technologies Cary 630). Band intensities are abbreviated as follows: weak (w), medium (m), strong (s). High resolution mass spectrometry were measured on a Waters XEVO G2-XS QTOF ESI spectrometer by Anne Jäger or a Agilant Technologies G6538A QTOF APCI. GC-MS spectrometry were measured on a Agilant Technologies GC6890N spectrometer with quadrupol MDS 597 3N. For column chromatography, Silica Gel 60 (Dm 0.040-0.063 mm mesh) from Macherey-Nagel was used. Precoated ALUGRAM Xtra Sil G/UV254 (silica on aluminium) sheets from Macherey-Nagel were used for TLC's. All eluent mixtures are given as volumetric ratios. For high performance liquid chromatography (HPLC) a Knauer K-504 pump, Knauer RI-detector K 2400 and a Macherey-Nagel VP250/21 Nucleodur 100-5 column were used. Unless otherwise stated, the flow rate was set to 10 mL/min. X-ray structures were measured on a Bruker D8 VENTURE by Anne Jäger.The heating of the reactions always took place with an oil bath. Crystals for x-ray structures were produced by slowly evaporating the NMR solvent at room temperature or by dissolving the substance in small amounts of CH_2Cl_2 and then layering it with pentane.

2. Reaction Optimisation

2.1 Supplementary Table 1: Catalyst Screening

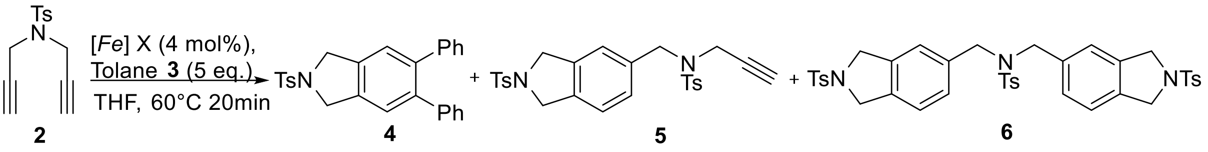

Entry[a,b]	[*Fe*]	**4**/%	**5**/%	**6**/%	**2**/%
**1**	**FeH(CO)(NO)(PPh$_3$)$_2$ 1**	**49 (50)**	**<5**	**<5**	**-**
2	FeH(CO)(NO)(dppp) **S1**	-	-	-	86
3	FeH(CO)(NO)(dppf) **S2**	-	-	-	91
4	FeH(CO)(NO)(PPh$_3$)(PCy$_3$) **S3**	-	-	-	83
5	FeH(CO)(NO)(P-(*p*-MeO-C$_6$H$_4$)$_3$)$_2$ **S4**	-	-	-	90
6	FeH(CO)(NO)(PPh$_3$)(P-(*p*-MeO-C$_6$H$_4$)$_3$) **S5**	-	-	-	87

[a] Reactions were run on 0.2 mmol scale. [b] Yield was determined by ^{1}H NMR integration with 1,3,5-trimethoxybenzene as internal standard. Isolated yields are given in parentheses.

2.2 Supplementary Table 2: Solvent Screening

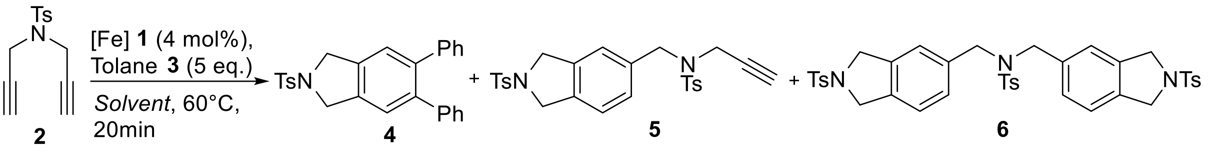

Entry[a,b]	*Solvent*	**4**/%	**5**/%	**6**/%	**2**/%
1	DCM	54	6	6	-
2	DCE	53	<5	6	-
3	Toluol	54	<5	7	-
4	MeCN	39	12	<5	-
5	EtOAc	42	<5	6	-
6	MTBE	53	<5	6	-
**7**	**1,4-dioxane**	**56 (54)**	**<5**	**6**	**-**

8	DMA	36	<5	<5	-
9	DMSO	24	15	traces	19
10	MeOH	40	8	<5	-
11	THF (standard)	49 (50)	<5	<5	-

[a] Reactions were run on 0.2 mmol scale. [b] Yield was determined by ^{1}H NMR integration with 1,3,5-trimethoxybenzene as internal standard. Isolated yields are given in parentheses.

.

2.3 Supplementary Table 3: Concentration Screening

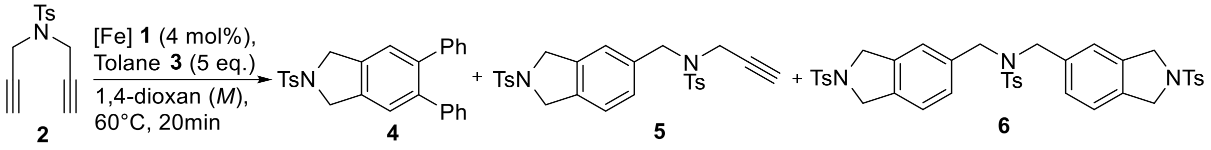

Entry[a,b]	*Concentration* / M	**4**/%	**5**/%	**6**/%	**2**/%
1	3.0	58	<5	<5	-
2	2.0 (standard)	56 (54)	<5	6	-
3	1.0	60	<5	<5	-
**4**	**0.5**	**67 (65)**	**<5**	**<5**	**-**
5	0.2	50	5	<5	-

[a] Reactions were run on 0.2 mmol scale. [b] Yield was determined by ^{1}H NMR integration with 1,3,5-trimethylbenzene as internal standard.

2.4 Supplementary Table 4:Temperature Screening

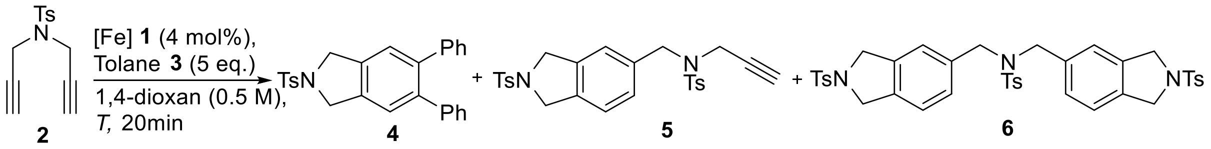

Entry[a,b]	*T* / °C	**4**/%	**5**%	**6**/%	**2**/%
1	rt	54	9	<5	<5
2	40	54	8	5	-
**3**	**60 (standard)**	**67 (65)**	**<5**	**<5**	**-**

4	80	62 (61)	<5	<5	-
5	100	57	traces	6	-

[a] Reactions were run on 0.2 mmol scale. [b] Yield was determined by ^{1}H NMR integration with 1,3,5-trimethylbenzene as internal standard.

2.5 Supplementary Table 5:Catalyst Loading

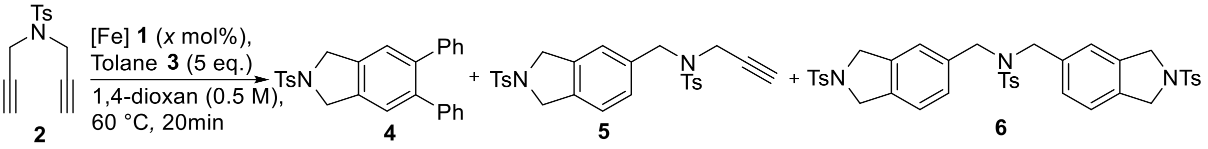

Entry[a,b]	*Catalyst Loading x* / mol%	**4**/%	**5**/%	**6**/%	**2**/%
1	1	59	9	5	-
2	2	60 (59)	6	5	-
**3**	**4 (standard)**	**67 (65)**	**<5**	**<5**	**-**
4	6	57	<5	<5	-
5	10	60	<5	<5	-
6	PPh_3 (4 mol%)	-	-	-	94
7	Without catalyst	-	-	-	87

[a] Reactions were run on 0.2 mmol scale. [b] Yield was determined by ^{1}H NMR integration with 1,3,5-trimethylbenzene as internal standard.

2.6 Supplementary Table 6:Time Screening

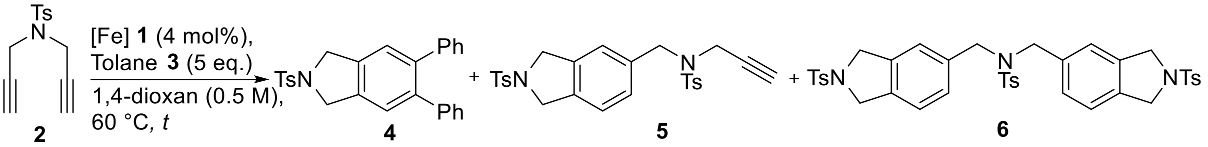

Entry[a,b]	*Time t* / min	**4**/%	**5**/%	**6**/%	**2**/%
1	1	65	9	<5	-
**2**	**5**	**67 (65)**	**6**	**5**	**-**
3	10	63 (63)	<5	<5	-
4	20 (standard)	67 (65)	<5	<5	-

[a] Reactions were run on 0.2 mmol scale. [b] Yield was determined by ^{1}H NMR integration with 1,3,5-trimethylbenzene as internal standard.

2.7 Supplementary Table 7: Equivalents Diphenylacetylene

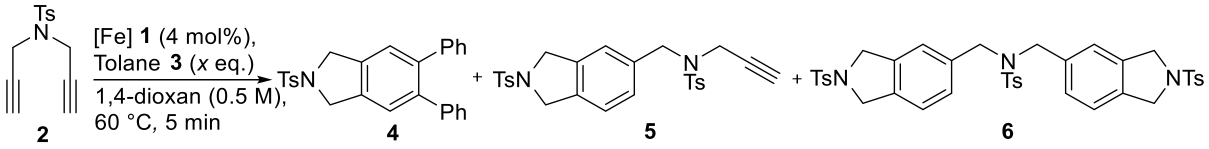

Entry[a,b]	*x* / eq.	**4**/%	**5**/%	**6**/%	**2**/%
1	1	25	<5	12	-
2	2	38	6	10	-
3	5 (standard)	67 (65)	6	5	-
4	10	72 (73)	<5	<5	-
**5**[c]	**10**	**(80)**	**-**	**-**	**-**

[a] Reactions were run on 0.2 mmol scale. [b] Yield was determined by ^{1}H NMR integration with 1,3,5-trimethylbenzene as internal standard. [c] Slowly addition of diyne **2** in 1,4-dioxane to a solution of catalyst **1** and diphenylacetylene **3** in 1,4-dioxane at 60 °C, 5 minutes counts after complete addition of diyne **2**.

3. TON-, TOF-Data and mechanistic studies

3.1 Supplementary Table 8: TOF

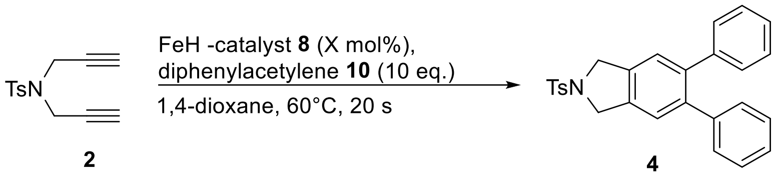

Entry	Catalys loading/mol%	Catalyst/mmol	n (used Substrat)/mmol	TOF (h^{-1})
1	4	0,02	0,25	2250
2	2	0,01	0,12	2160
3	1	0,005	0,065	2340
4	0,5	0,0025	0,035	2520

Reactions were run on 0.5 mmol scale. Yield/Conversion was determined by ^{1}H NMR integration with 1,3,5-trimethylbenzene as internal standard. Diyne **2** was added in one portion (solved in 0.5 mL of 1,4-dioxane) to the catalyst and diphenylacetylene **3** solved in 0.5 mL of 1,4-dioxane at 60 °C. The reactions were quenched after 20 s via filtration ofer a silica pad.

Average TOF: 2318 h^{-1}

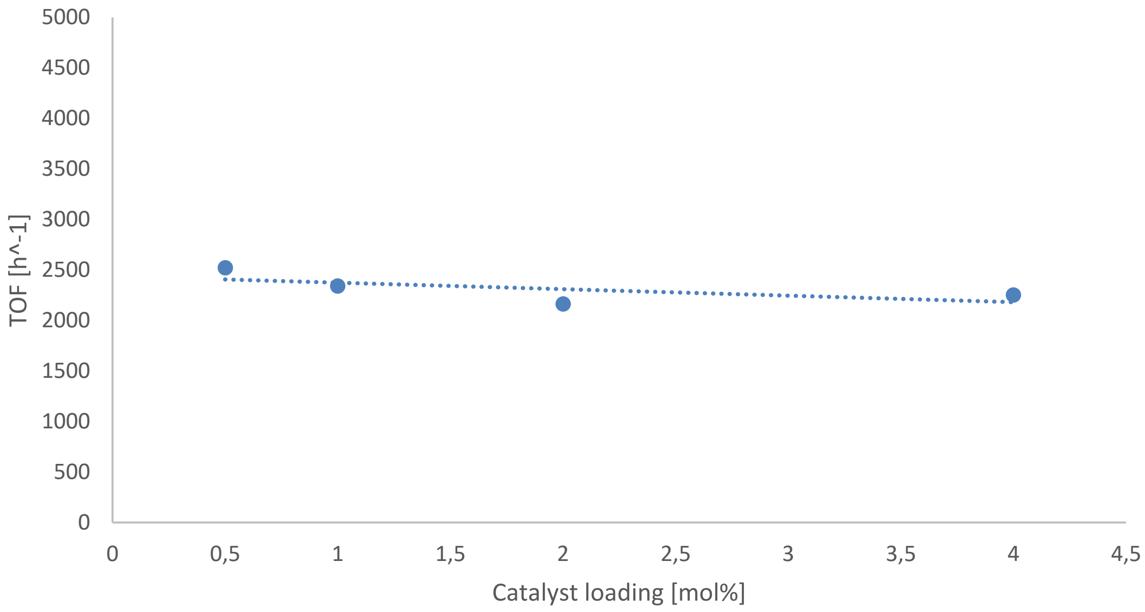

Supplementary Figure 1: TOF versus catalyst loading

3.2 Supplementary Table 9: TON

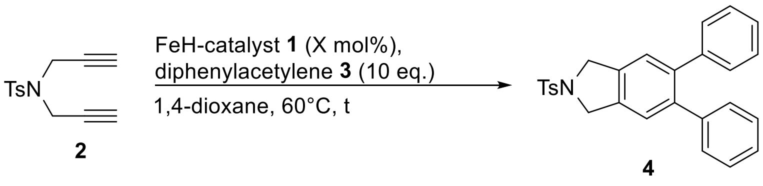

Entry	Catalys loading/mol%	Catalyst/mmol	n (used Substrat)/mmol	t/h	TON
1	4	0,02	0,5	1	25
2	2	0,01	0,5	1	50
3	1	0,005	0,44	1	88
4	1	0,005	0,43	15	86

Reactions were run on 0.5 mmol scale. Yield/Conversion was determined by ^{1}H NMR integration with 1,3,5-trimethylbenzene as internal standard. Diyne **2** was added in one portion (solved in 0.5 mL of 1,4-dioxane) to the catalyst and diphenylacetylene **3** solved in 0.5 mL of 1,4-dioxane at 60 °C. The reactions were quenched after 1 h or 15 h via filtration ofer a silica pad.

Average TON: 87

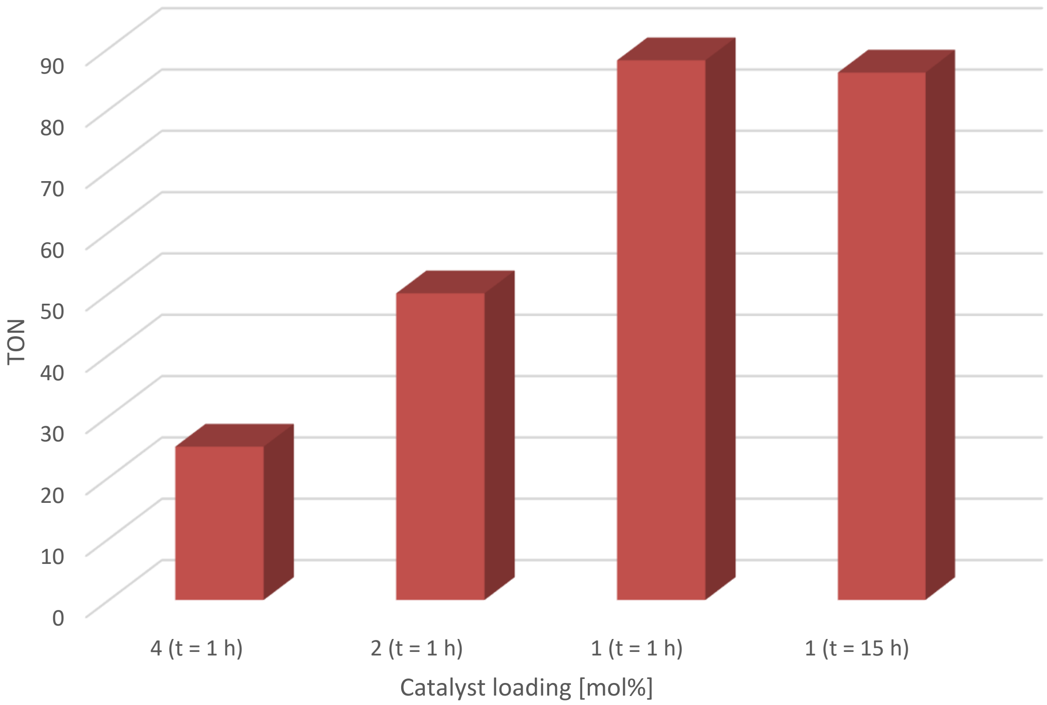

Supplementary Figure 2: TON versus catalyst loading

3.3 Supplementary Table 10: Kinetic isotope effect

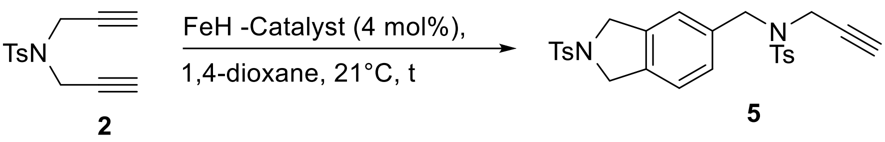

Entry	t/s	Conversion/% (Fe-H)	Conversion/% (Fe-H/Fe-D 60:40)
1	60	3	2
2	120	21	18
2	240	35	28
3	480	45	35
4	600	49	38
5	900	53	41
6	1200	56	44

Reactions were run on 0.5 mmol scale. Conversion was determined by GC with Dodecane as internal standard.

Reaction procedure:

Diyne **2** (124 mg, 500 µmol, 1.0 eq.) and Dodecane (56.8 µL, 0.25 mmol, 0.5 eq.) was solved in 1,4-dioxane (1.00 mL) and the solution was cooled to 21 °C with a water bath. Catalyst (12.8 mg, 20.0 µmol, 4 mol%, Fe-H or Fe-H/Fe-D (60/40)) was added and after defined times (see table) 30 µL of the reaction mixture was taken. After a little workup with a dilute hydrochloride acide solution (1N)/ethyl acetate mixture (1 mL/0.5 mL, to deactivate the catalyst) the ethyl acetate phase was taken and measured via GC.

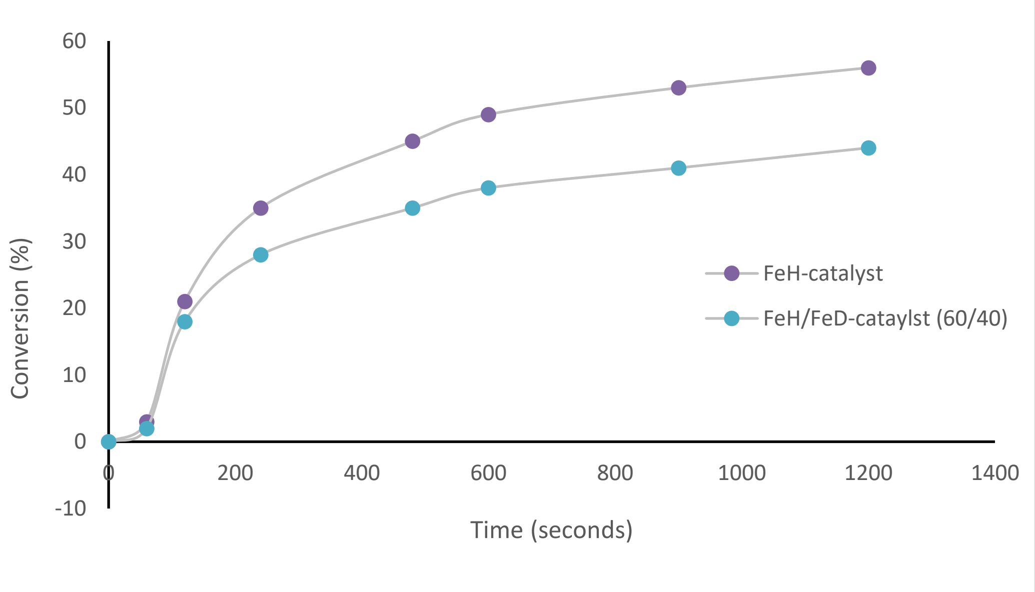

Supplementary Figure 3: Kinetic isotope effect: conversion-time correlation

3.4 Control experiments

Addition of PPh_3.

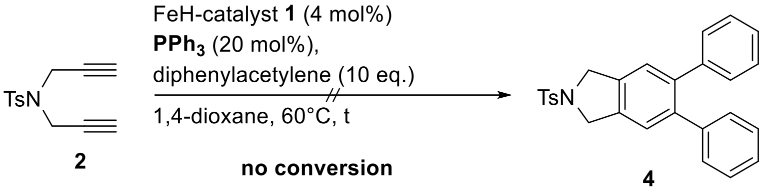

Supplementary Figure 4:

➔ No conversion. Yield/Conversion was determined by ^1H NMR integration with 1,3,5-trimethylbenzene as internal standard.

$Fe(NO)_2(PPh_3)_2$ as catalyst.

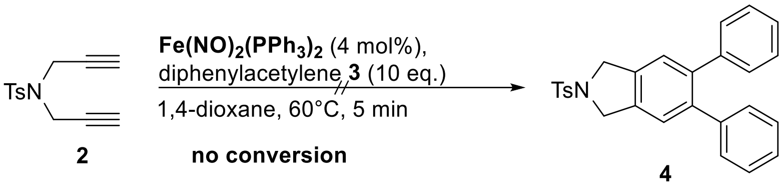

Supplementary Figure 5:

3.5 ^{31}P NMR-study

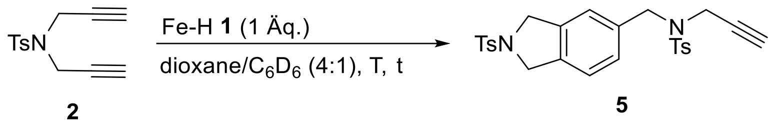

Supplementary Figure 6:

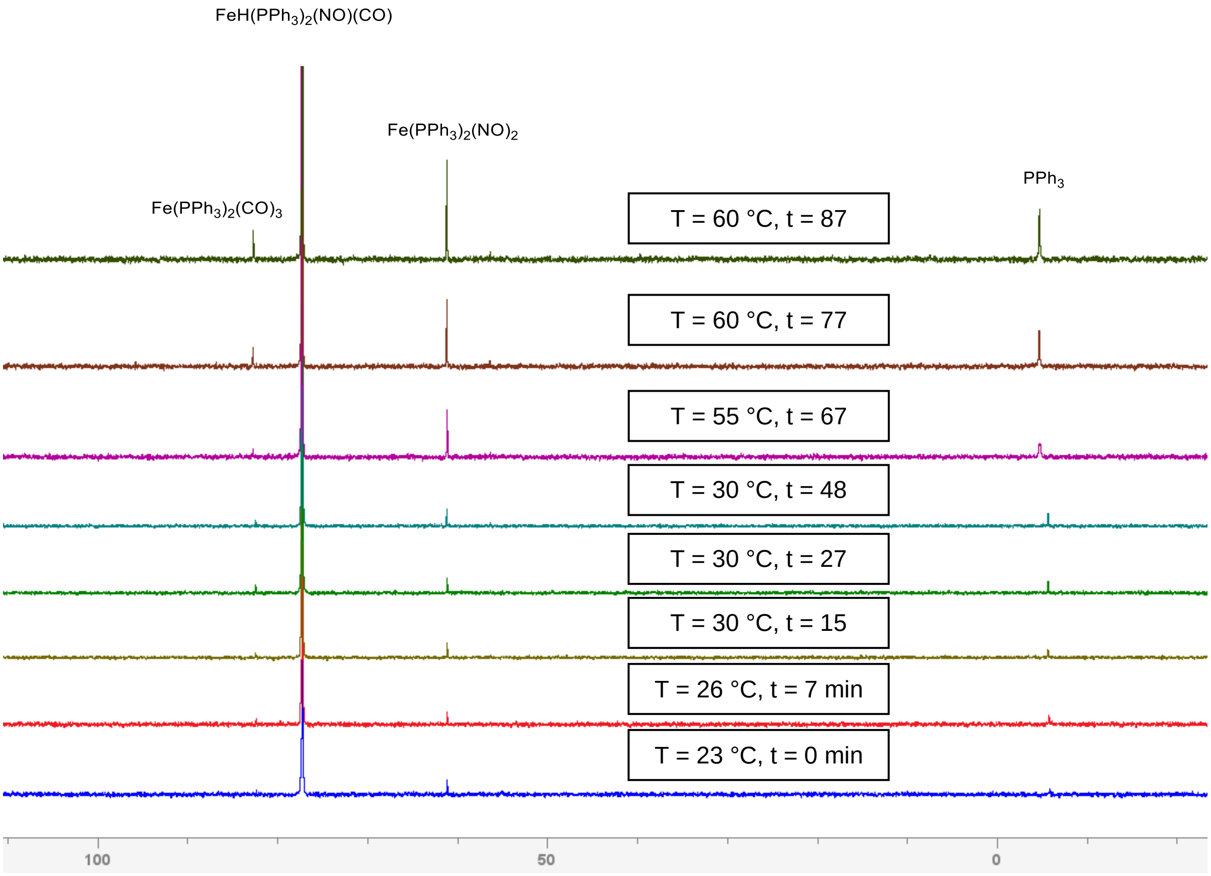

Supplementary Figure 6: Time-dependant ^{31}P NMR-studies.

4. Procedures

General Procedure 1 (GP1): Catalysis Products

In a Heatgun-dried (2 minutes) 10 mL-screw cap Schlenk tube, the catalyst $FeH(CO)(NO)(PPh_3)_2$ **1** (4 mol%) and diphenylacetylene **3** (10.0 eq.) were dissolved in anhydrous 1,4-dioxane (*c* = 1.00 mol/L). The diyne in 1,4-dioxane (*c* = 1.00 mol/L) was slowly added at 60°C. The reaction mixture was stirred for 5 minutes at 60 °C. The solvent was removed under reduced pressure and the crude products were purified by silica gel column chromatography (isohexane/ethyl acetate mixtures).

4.1 Catalysis Products

4.1.1 Preparation of 5,6-Diphenyl-2-tosylisoindoline **4**

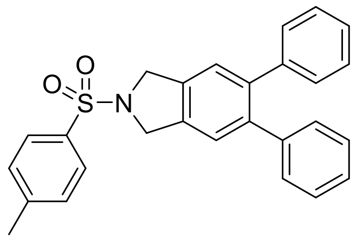

4

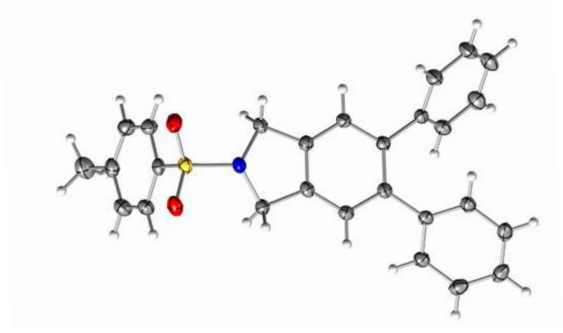

According to GP1, $FeH(CO)(NO)(PPh_3)_2$ **1** (12.8 mg, 20.0 µmol, 4 mol%) and diphenylacetylene **3** (891 mg, 5.00 mmol, 10 eq.) was dissolved in anhydrous 1,4-dioxane (0.5 mL). Diyne **2** (124 mg, 500 µmol, 1.0 eq.) in anhydrous 1,4-dioxane (0.5 mL) was slowly added at 60 °C. The reaction mixture was stirred for 5 minutes at 60 °C. After solvent evaporation and purification by silica gel column chromatography (isohexane/ethyl acetate – 10/1 to 8/1), the product **4** was obtained as a pale yellow solid in a yield of 204 mg (479 µmol, 96%).

^{1}H NMR (300 MHz, $CDCl_3$) δ = 7.92–7.74 (m, 2H), 7.39–7.30 (m, 2H), 7.22 (s, 2H), 7.21–7.14 (m, 6H), 7.09–6.98 (m, 4H), 4.70 (s, 4H), 2.42 (s, 3H) ppm; **^{13}C NMR** (75 MHz, $CDCl_3$) δ = 143.9, 141.1, 140.8, 135.6, 133.8, 130.0, 129.9, 128.1, 127.8, 126.9, 124.8, 53.8, 21.7 ppm; **IR** (ATR) $\tilde{v}$ = 667 (s), 701 (s), 734 (m), 768 (m), 816 (w), 842 (w), 909 (m), 1021 (w), 1062 (m), 1096 (m), 1163 (s), 1346 (m), 1405 (w), 1476 (w), 1599 (w), 2851 (w), 2922 (w), 3027 (w), 3056 (w) cm^{-1}; **HRMS** (ESI): calcd. for $C_{27}H_{23}NO_2SNa^+$: 448.1342; found: 448.1334 ***R*f** = 0.11 (isohexane/ethyl acetate – 10/1).

The analytical data are in good accordance with the literature.[3]

The X-Ray structure of **4** is deposited at the Cambridge Crystallographic Data Centre (www.ccdc.cam.ac.uk) under CCDC 242 4455.

4.1.2 Preparation of 5-phenyl-2-tosylisoindoline **7**

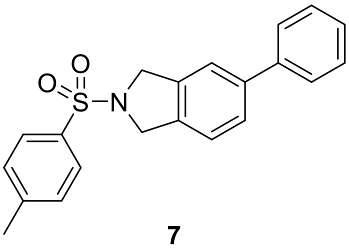

7

According to GP1, $FeH(CO)(NO)(PPh_3)_2$ **1** (12.8 mg, 20.0 µmol, 4 mol%) and Phenylacetylene (0.55 mL, 5.00 mmol, 10 eq.) was dissolved in anhydrous 1,4-dioxane (0.5 mL). Diyne **2** (124 mg, 500 µmol, 1.0 eq.) in anhydrous 1,4-dioxane (0.5 mL) was slowly added at 60 °C. The reaction mixture was stirred for 5 minutes at 60 °C. After solvent evaporation and purification by silica gel column chromatography isohexane/ethyl acetate – 5/1), the product **7** was obtained as a yellow solid in a yield of 168 mg (480 µmol, 96%).

^{1}H NMR (400 MHz, $CDCl_3$) δ = 7.84–7.76 (m, 2H), 7.55–7.49 (m, 2H), 7.48–7.39 (m, 3H), 7.39–7.30 (m, 4H), 7.23 (d, J = 7.9 Hz, 1H), 4.71–4.64 (m, 4H), 2.41 (s, 3H) ppm; **^{13}C NMR** (101 MHz, $CDCl_3$) δ = 143.9, 141.4, 140.9, 137.0, 135.3, 133.8, 130.0, 129.0, 127.8, 127.7, 127.2, 127.1, 123.1, 121.5, 53.9, 53.7, 21.6 ppm; **IR** (ATR) $\tilde{\nu}$ = 667 (s), 701 (m), 731 (m), 760 (m), 816 (m), 909 (m), 1018 (w), 1062 (m), 1096 (s), 1159 (s), 1342 (m), 1420 (w), 1483 (m), 1595 (w), 2851 (w), 2922 (w), 3030 (w), 3056 (w), 3056 (w), 3056 (w) cm^{-1}; **HRMS** (ESI): calcd. for $C_{21}H_{19}NO_2SNa^+$: 372.1029; found: 372.1035; $\boldsymbol{R_f}$ = 0.11 (isohexane/ethyl acetate – 10/1).

The analytical data are in good accordance with the literature.[4]

4.1.3 Preparation of 5-(Thiophen-2-yl)-2-tosylisoindoline **8**

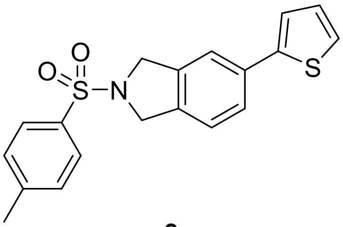

8

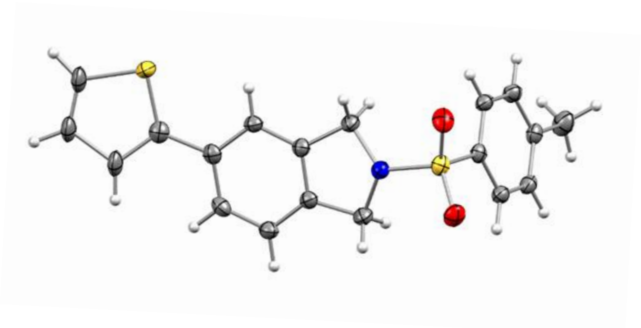

According to GP1, $FeH(CO)(NO)(PPh_3)_2$ **1** (12.8 mg, 20.0 µmol, 4 mol%) and 2-ethynylthiophene (541 mg, 5.00 mmol, 10 eq.) was dissolved in anhydrous 1,4-dioxane (0.5 mL). Diyne **2** (124 mg, 500 µmol, 1.0 eq.) in anhydrous 1,4-dioxane (0.5 mL) was slowly added at 60 °C.

The reaction mixture was stirred for 5 minutes at 60 °C. After solvent evaporation and purification by silica gel column chromatography isohexane/ethyl acetate – 5/1), the product **8** was obtained as a yellow solid in a yield of 93.1 mg (261 µmol, 52%).

^{1}H NMR (400 MHz, $CDCl_3$) δ = 7.84–7.75 (m, 2H), 7.56–7.44 (m, 1H), 7.39 (s, 1H), 7.35–7.30 (m, 2H), 7.28–7.23 (m, 2H), 7.17 (d, J = 8.0 Hz, 1H), 7.06 (dd, J = 5.1, 3.7 Hz, 1H), 4.69–4.53 (m, 4H), 2.40 (s, 3H) ppm; **^{13}C NMR** (101 MHz, $CDCl_3$) δ = 143.9, 143.8, 137.2, 135.5, 134.4, 133.8, 130.0, 128.2, 127.8, 125.8, 125.2, 123.5, 123.2, 120.2, 53.8, 53.7, 21.7 ppm; **IR** (ATR) $\tilde{\nu}$ = 667 (m), 704 (m), 731 (w), 775 (w), 816 (m), 842 (w), 909 (w), 1062 (w), 1096 (m), 1163 (s), 1342 (m), 1469 (w), 1491 (w), 2922 (w) cm^{-1} **HRMS** (ESI): calcd. for $C_{19}H_{17}NO_2S_2Na^+$: 378.0593; found: 378.0579; $\boldsymbol{R}_\mathbf{f}$ = 0.34 (isohexane/ethyl acetate – 5/1).

The X-Ray structure of **8** is deposited at the Cambridge Crystallographic Data Centre (www.ccdc.cam.ac.uk) under CCDC 242 4445.

4.1.4 Preparation of 5-Phenethyl-2-tosylisoindoline **9**

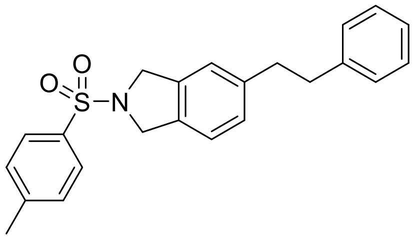

9

According to GP1, $FeH(CO)(NO)(PPh_3)_2$ **1** (12.8 mg, 20.0 µmol, 4 mol%) and 3-butyn-1-ylbenzene **S17** (0.70 mL, 5.00 mmol, 10 eq.) was dissolved in anhydrous 1,4-dioxane (0.5 mL). Diyne **2** (124 mg, 500 µmol, 1.0 eq.) in anhydrous 1,4-dioxane (0.5 mL) was slowly added at 60 °C. The reaction mixture was stirred for 5 minutes at 60 °C. After solvent evaporation and purification by silica gel column chromatography isohexane/ethyl acetate – 5/1), the product **9** was obtained as a pale yellow solid in a yield of 167 mg (441 µmol, 88%).

^{1}H NMR (400 MHz, $CDCl_3$) δ = 7.81–7.74 (m, 2H), 7.36–7.24 (m, 4H), 7.23–7.17 (m, 1H), 7.17–7.12 (m, 2H), 7.10–7.02 (m, 2H), 6.98 (s, 1H), 4.59 (s, 4H), 2.93–2.81 (m, 4H), 2.41 (s, 3H) ppm; **^{13}C NMR** (101 MHz, $CDCl_3$) δ = 143.7, 141.8, 141.5, 136.4, 133.9, 133.8, 129.9, 128.5, 128.5, 128.2, 127.7, 126.2, 122.7, 122.6, 53.8, 53.7, 38.1, 37.8, 21.6 ppm; **IR** (ATR) $\tilde{\nu}$ = 667 (s), 701 (m), 738 (m), 813 (m), 909 (w), 1059 (m), 1096 (s), 1163 (s), 1342 (m), 1495 (m), 1599 (w), 2855 (w), 2922 (w), 3027 (w), 3027 (w) cm^{-1}; **HRMS** (ESI): calcd. for $C_{23}H_{23}NO_2SNa^+$: 400.1342; found: 400.1339; $\boldsymbol{R}_\mathbf{f}$ = 0.14 (isohexane/ethyl acetate – 10/1).

4.1.5 Preparation of 5-(4,4,5,5-Tetramethyl-1,3,2-dioxaborolan-2-yl)-2-tosylisoindoline **10**

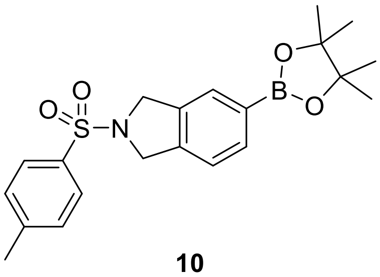

10

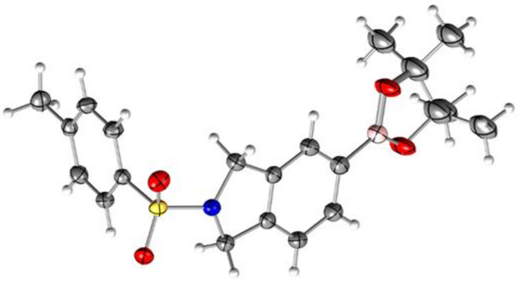

According to GP1, $FeH(CO)(NO)(PPh_3)_2$ **1** (12.8 mg, 20.0 µmol, 4 mol%) and 2-ethynyl-4,4,5,5-tetramethyl-1,3,2-dioxaborolane (760 mg, 5.00 mmol, 10 eq.) was dissolved in anhydrous 1,4-dioxane (0.5 mL). Diyne **2** (124 mg, 500 µmol, 1.0 eq.) in anhydrous 1,4-dioxane (0.5 mL) was slowly added at 60 °C. The reaction mixture was stirred for 5 minutes at 60 °C. After solvent evaporation and purification by silica gel column chromatography isohexane/ethyl acetate – 10/1), the product **10** was obtained as a colourless solid in a yield of 116 mg (291 µmol, 58%).

^{1}H NMR (400 MHz, $CDCl_3$) δ = 7.79–7.72 (m, 2H), 7.67 (d, J = 7.7 Hz, 1H), 7.61 (s, 1H), 7.33–7.27 (m, 2H), 7.17 (d, J = 7.8 Hz, 1H), 4.79–4.50 (m, 4H), 2.39 (s, 3H), 1.32 (s, 12H) ppm; **^{13}C NMR** (101 MHz, $CDCl_3$) δ = 143.8, 139.4, 135.7, 134.3, 133.9, 130.0, 129.0, 127.7, 122.1, 84.1, 54.0, 53.7, 25.0, 21.6 ppm; **IR** (ATR) $\tilde{\nu}$ = 663 (s), 723 (m), 768 (m), 820 (m), 854 (m), 909 (m), 965 (m), 1066 (m), 1096 (m), 1163 (s), 1219 (w), 1271 (m), 1342 (s), 1424 (m), 1469 (w), 1618 (w), 2978 (w) cm^{-1}; **HRMS** (ESI): calcd. for $C_{21}H_{26}BNO_4SNa^+$: 422.1568; found: 422.1576; $\boldsymbol{R_f}$ = 0.20 (isohexane/ethyl acetate – 10/1).

The X-Ray structure of **10** is deposited at the Cambridge Crystallographic Data Centre (www.ccdc.cam.ac.uk) under CCDC 242 4452.

4.1.6 Preparation of 2-Tosyl-5-(trimethylsilyl)isoindoline **11**

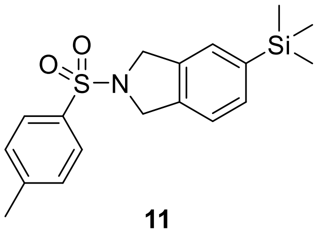

11

According to GP1, $FeH(CO)(NO)(PPh_3)_2$ **1** (12.8 mg, 20.0 µmol, 4 mol%) and (trimethylsilyl)acetylene (0.69 mL, 5.0 mmol, 10 eq.) was dissolved in anhydrous 1,4-dioxane (0.5 mL). Diyne **2** (124 mg, 500 µmol, 1.0 eq.) in anhydrous 1,4-dioxane (0.5 mL) was slowly added at 60 °C. The reaction mixture was stirred for 5 minutes at 60 °C. After solvent evaporation and

purification by silica gel column chromatography isohexane/ethyl acetate – 10/1), the product **11** was obtained as a brown solid in a yield of 152 mg (440 mmol, 88%).

^{1}H NMR (400 MHz, $CDCl_3$) δ = 7.82–7.73 (m, 2H), 7.39 (d, J = 7.5 Hz, 1H), 7.34–7.27 (m, 3H), 7.17 (d, J = 7.6 Hz, 1H), 4.62 (s, 4H), 2.40 (s, 3H), 0.23 (s, 9H) ppm; **^{13}C NMR** (101 MHz, $CDCl_3$) δ = 143.8, 140.5, 136.9, 135.7, 133.8, 132.8, 129.9, 127.8, 127.5, 122.2, 53.9, 53.8, 21.6, −1.0 ppm; **IR** (ATR) $\tilde{\nu}$ = 663 (s), 753 (m), 835 (s), 902 (m), 1096 (m), 1163 (s), 1249 (m), 1346 (m), 1401 (w), 1469 (w), 1595 (w), 2952 (w) cm^{-1}; **HRMS** (ESI): calcd. for $C_{18}H_{23}NO_2SSiNa^+$: 368.1111; found: 368.1118; ***R*$_f$** = 0.37 (isohexane/ethyl acetate – 10/1).

4.1.7 Preparation of 5-Cyclopropyl-2-tosylisoindoline **12**

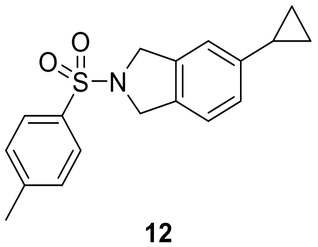

12

According to GP1, $FeH(CO)(NO)(PPh_3)_2$ **1** (12.8 mg, 20.0 µmol, 4 mol%) and cyclopropylacetylene (0.42 mL, 5.0 mmol, 10 eq.) was dissolved in anhydrous 1,4-dioxane (0.5 mL). Diyne **2** (124 mg, 500 µmol, 1.0 eq.) in anhydrous 1,4-dioxane (0.5 mL) was slowly added at 60 °C. The reaction mixture was stirred for 5 minutes at 60 °C. After solvent evaporation and purification by silica gel column chromatography isohexane/ethyl acetate – 10/1 to 8/1), the product **12** was obtained as a colourless solid in a yield of 145 mg (462 µmol, 92%).

^{1}H NMR (400 MHz, $CDCl_3$) δ = 7.83–7.69 (m, 2H), 7.37–7.27 (m, 2H), 7.04 (d, J = 7.8 Hz, 1H), 6.94 (dd, J = 7.9, 1.5 Hz, 1H), 6.86 (d, J = 1.5 Hz, 1H), 4.57 (s, 4H), 2.40 (s, 3H), 1.93 – 1.77 (m, 1H), 0.97–0.90 (m, 2H), 0.67–0.57 (m, 2H) ppm; **^{13}C NMR** (101 MHz, $CDCl_3$) δ = 144.1, 143.7, 136.4, 133.9, 133.3, 129.9, 127.7, 125.6, 122.5, 119.8, 53.8, 53.6, 21.6, 15.4, 9.4 ppm; **IR** (ATR) $\tilde{\nu}$ = 663 (s), 731 (m), 813 (m), 909 (m), 1044 (m), 1096 (m), 1159 (s), 1342 (m), 1401 (w), 1461 (w), 1498 (w), 1618 (w), 2851 (w), 2919 (w), 3004 (w) cm^{-1}; **HRMS** (ESI): calcd. for $C_{18}H_{19}NO_2SNa^+$: 336.1029; found: 336.1049; ***R*$_f$** = 0.46 (isohexane/ethyl acetate – 5/1).

The analytical data are in good accordance with the literature.[5]

4.1.8 Preparation of 5-Phenyl-6-(phenylethynyl)-2-tosylisoindoline **13**

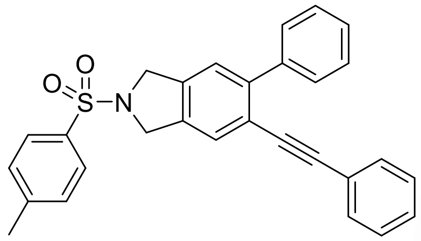

13

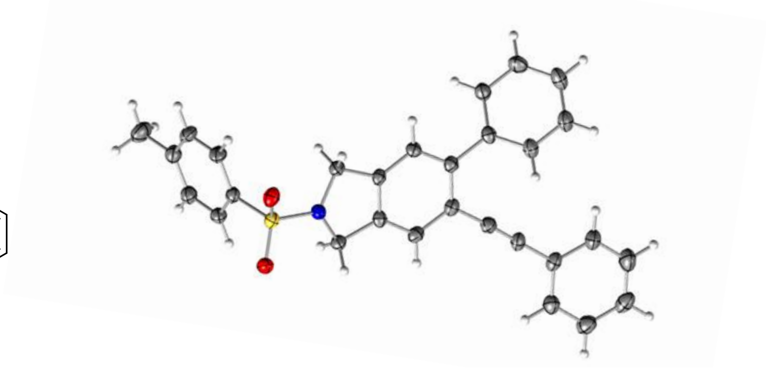

According to GP1, $FeH(CO)(NO)(PPh_3)_2$ **1** (12.8 mg, 20.0 µmol, 4 mol%) and 1,4-diphenyl-1,3-butadiyne (1.264 g, 5.00 mmol, 10 eq.) was dissolved in anhydrous 1,4-dioxane (1.0 mL). Diyne **2** (124 mg, 500 µmol, 1.0 eq.) in anhydrous 1,4-dioxane (0.5 mL) was slowly added at 60 °C. The reaction mixture was stirred for 5 minutes at 60 °C. After solvent evaporation and purification by silica gel column chromatography isohexane/ethyl acetate – 10/1), the product **13** was obtained as a yellow solid in a yield of 207 mg (460 µmol, 92%).

^{1}H NMR (400 MHz, $CDCl_3$) δ = 7.83–7.75 (m, 2H), 7.62–7.52 (m, 2H), 7.48–7.37 (m, 4H), 7.35–7.31 (m, 2H), 7.27 (s, 5H), 7.21 (s, 1H), 4.66 (s, 4H), 2.42 (s, 3H) ppm; **^{13}C NMR** (101 MHz, $CDCl_3$) δ = 144.1, 144.0, 140.1, 136.9, 135.3, 133.8, 131.5, 130.0, 129.4, 128.4, 128.1, 127.9, 127.7, 126.8, 123.8, 123.2, 121.6, 92.8, 89.0, 53.8, 53.5, 21.7 ppm (due to coincidental chemical equivalence of two carbon resonances one signal is missing); **IR** (ATR) $\tilde{\nu}$ = 667 (s), 701 (m), 757 (m), 813 (m), 909 (m), 1066 (m), 1096 (m), 1159 (s), 1305 (m), 1346 (m), 1442 (w), 1491 (m), 1595 (w), 2851 (w), 2922 (w), 3030 (w), 3056 (w) cm^{-1}; **HRMS** (ESI): calcd. for $C_{29}H_{24}NO_2S^+$: 450.1522; found: 450.1533; ***R*f** = 0.33 (isohexane/ethyl acetate – 5/1).

The X-Ray structure of **13** is deposited at the Cambridge Crystallographic Data Centre (www.ccdc.cam.ac.uk) under CCDC 242 4456.

4.1.9 Preparation of 5-Methyl-6-phenyl-2-tosylisoindoline **14**

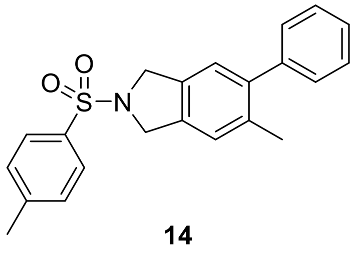

14

According to GP1, $FeH(CO)(NO)(PPh_3)_2$ **1** (12.8 mg, 20.0 µmol, 4 mol%) and methylphenylacetylene (726 mg, 5.00 mmol, 10 eq.) was dissolved in anhydrous 1,4-dioxane (0.5 mL). Diyne

2 (124 mg, 500 µmol, 1.0 eq.) in anhydrous 1,4-dioxane (0.5 mL) was slowly added at 60 °C. The reaction mixture was stirred for 5 minutes at 60 °C. After solvent evaporation and purification by silica gel column chromatography isohexane/ethyl acetate – 10/1), the product **14** was obtained as a yellow solid in a yield of 153 mg (422 µmol, 84%).

^{1}H NMR (400 MHz, $CDCl_3$) δ = 7.82–7.74 (m, 2H), 7.43–7.28 (m, 5H), 7.24–7.19 (m, 2H), 7.07 (s, 1H), 7.02 (s, 1H), 4.86–4.44 (m, 4H), 2.41 (s, 3H), 2.20 (s, 3H) ppm; **^{13}C NMR** (101 MHz, $CDCl_3$) δ = 143.8, 141.9, 141.5, 135.4, 135.3, 133.9, 133.8, 130.0, 129.2, 128.3, 127.8, 127.2, 124.3, 123.9, 53.7, 53.7, 21.7, 20.6 ppm; **IR** (ATR) $\tilde{\nu}$ = 667(s), 704 (m), 734 (m), 768 (m), 813 (w), 842 (w), 872 (w), 909 (w), 1062 (m), 1096 (m), 1163 (s), 1346 (m), 1401 (w), 1483 (w),1599 (w), 2855 (w), 2922 (w), 2952 (w), 3027 (w), 3027 (w) cm^{-1}; **HRMS** (ESI): calcd. for $C_{22}H_{21}NO_2SNa^+$: 386.1185; found: 386.1197; $\boldsymbol{R_f}$ = 0.20 (isohexane/ethyl acetate – 10/1).

The analytical data are in good accordance with the literature.[4]

4.1.10 Preparation of (2-Tosylisoindoline-5,6-diyl)dimethanol **15**

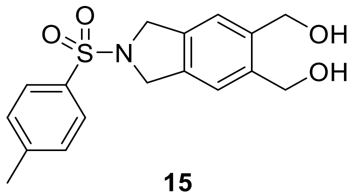

15

According to GP1, $FeH(CO)(NO)(PPh_3)_2$ **1** (12.8 mg, 20.0 µmol, 4 mol%) and 2-butyne-1,4-diol (431 mg, 5.00 mmol, 10 eq.) was dissolved in anhydrous 1,4-dioxane (0.5 mL). Diyne **2** (124 mg, 500 µmol, 1.0 eq.) in anhydrous 1,4-dioxane (0.5 mL) was slowly added at 60 °C. The reaction mixture was stirred for 5 minutes at 60 °C. After solvent evaporation and purification by silica gel column chromatography isohexane/ethyl acetate – 0/1), the product **15** was obtained as a colourless solid in a yield of 141 mg (423 µmol, 85%).

^{1}H NMR (300 MHz, DMSO-d_6) δ = 7.80–7.70 (m, 2H), 7.45–7.36 (m, 2H), 7.25 (s, 2H), 5.07 (t, J = 5.4 Hz, 2H), 4.54 (s, 4H), 4.46 (d, J = 5.3 Hz, 4H), 2.35 (s, 3H) ppm; **^{13}C NMR** (75 MHz, DMSO-d_6) δ = 143.6, 139.1, 133.9, 133.0, 130.0, 127.4, 120.8, 60.1, 53.5, 21.0 ppm; **IR** (ATR) $\tilde{\nu}$ = 663 (s), 772 (m), 816 (m), 872 (m), 995 (m), 1036 (m), 1070 (m), 1096 (m), 1159 (s), 1264 (m), 1305 (m), 1342 (s), 1454 (w), 3291 (m) cm^{-1}; **HRMS** (ESI): calcd. for $C_{17}H_{19}NO_4SNa^+$: 356.0927; found: 356.0934; $\boldsymbol{R_f}$ = 0.51 (isohexane/ethyl acetate – 0/1).

The analytical data are in good accordance with the literature.[6]

4.1.11 Preparation of 4,4-Dimethyl-5',6'-diphenyl-1',3'-dihydrospiro[cyclohexane-1,2'-indene]-2,6-dione **16**

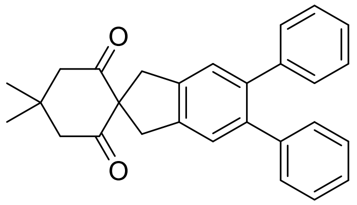

16

According to GP1, $FeH(CO)(NO)(PPh_3)_2$ **1** (12.8 mg, 20.0 µmol, 4 mol%) and diphenylacetylene **3** (891 mg, 5.00 mmol, 10 eq.) was dissolved in anhydrous 1,4-dioxane (0.5 mL). Diyne **S11** (108 mg, 500 µmol, 1.0 eq.) in anhydrous 1,4-dioxane (0.5 mL) was slowly added at 60 °C. The reaction mixture was stirred for 5 minutes at 60 °C. After solvent evaporation and purification by silica gel column chromatography isohexane/ethyl acetate – 10/1 to 8/1), the product **16** was obtained as a colourless solid in a yield of 175 mg (444 µmol, 89%).

^{1}H NMR (400 MHz, $CDCl_3$) δ = 7.22 (s, 2H), 7.19–7.15 (m, 6H), 7.11–7.06 (m, 4H), 3.55 (s, 4H), 2.75 (s, 4H), 1.06 (s, 6H) ppm; **^{13}C NMR** (101 MHz, $CDCl_3$) δ = 206.7, 141.8, 139.9, 139.0, 130.0, 127.9, 126.4, 126.4, 71.5, 51.6, 38.6, 30.8, 28.6 ppm; **IR** (ATR) $\tilde{\nu}$ = 731 (s), 768 (m), 909 (m), 954 (w), 1021 (w), 1073 (w), 1163 (w), 1237 (m), 1323 (m), 1372 (w), 1428 (m), 1476 (m), 1599 (w), 1692 (s), 1726 (m) ,2922 (w), 2952 (m), 3023 (w), 3056 (w) cm^{-1}; **HRMS** (ESI): calcd. for $C_{28}H_{26}O_2Na^+$: 417.1825; found: 462.1816; $\boldsymbol{R}_\mathbf{f}$ = 0.11 (isohexane/ethyl acetate – 10/1).

4.1.12 Preparation of Dimethyl 5,6-diphenyl-1,3-dihydro-2*H*-indene-2,2-dicarboxylate **17**

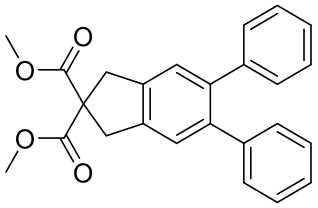

17

According to GP1, $FeH(CO)(NO)(PPh_3)_2$ **1** (12.8 mg, 20.0 µmol, 4 mol%) and diphenylacetylene **3** (891 mg, 5.00 mmol, 10 eq.) was dissolved in anhydrous 1,4-dioxane (0.5 mL). Diyne **S13** (104 mg, 500 µmol, 1.0 eq.) in anhydrous 1,4-dioxane (0.5 mL) was slowly added at 60 °C. The reaction mixture was stirred for 5 minutes at 60 °C. After solvent evaporation and purification by silica gel column chromatography isohexane/ethyl acetate – 10/1), the product **17** was obtained as a brown oil in a yield of 190 mg (492 µmol, 98%).

^{1}H NMR (400 MHz, $CDCl_3$) δ = 7.26 (s, 2H), 7.21–7.16 (m, 6H), 7.14–7.08 (m, 4H), 3.79 (s, 6H), 3.70 (s, 4H) ppm; **^{13}C NMR** (101 MHz, $CDCl_3$) δ = 172.2, 141.7, 139.8, 139.4, 130.0, 127.9, 126.5, 126.4, 60.6, 53.2, 40.6 ppm; **IR** (ATR) $\tilde{\nu}$ = 701 (s), 731 (s), 768 (m), 820 (w), 895 (m),

965 (w), 1066 (m), 1155 (m), 1200 (m), 1260 (s), 1431 (m), 1476 (m), 1603 (w), 1729 (s), 2844 (w), 2952 (w), 3023 (w) cm^{-1}; **HRMS** (ESI): calcd. for $C_{25}H_{21}O_4Na^+$: 409.1409; found: 409.1404; $\boldsymbol{R_f}$ = 0.26 (isohexane/ethyl acetate – 10/1).

The analytical data are in good accordance with the literature.[7]

4.1.13 Preparation of 2,3-Diphenyl-9,10-dihydroanthracene-9,10-diol **18**

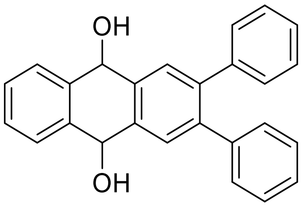

18

According to GP1, $FeH(CO)(NO)(PPh_3)_2$ **1** (12.8 mg, 20.0 µmol, 4 mol%) and diphenylacetylene **3** (891 mg, 5.00 mmol, 10 eq.) was dissolved in anhydrous 1,4-dioxane (0.5 mL). Diyne **S65** (93.1 mg, 500 µmol, 1.0 eq.) in anhydrous 1,4-dioxane (0.5 mL) was slowly added at 60 °C. The reaction mixture was stirred for 5 minutes at 60 °C. After solvent evaporation and purification by silica gel column chromatography isohexane/ethyl acetate – 5/1), the product **18** was obtained as a yellow solid (mixture of two diastereomers) in a yield of 121 mg (331 µmol, 66 %).

Diastereomer 1:

^{1}H NMR (300 MHz, DMSO-d$_6$) δ = 7.73–7.62 (m, 4H), 7.38–7.29 (m, 2H), 7.29–7.16 (m, 6H), 7.14–7.07 (m, 4H), 6.35 (d, J = 7.0 Hz, 2H), 5.47 (d, J = 6.8 Hz, 2H) ppm; **^{13}C NMR** (101 MHz, DMSO-d$_6$) δ = 142.0, 139.6, 1319.2, 138.2, 130.0, 128.5, 126.9, 126.6, 125.8, 123.5, 66.6 ppm; **IR** (ATR) $\tilde{\nu}$ = 693 (s), 734 (m), 764 (m), 902 (m), 1029 (m), 1148 (m), 1178 (m), 1338 (m), 1465 (m), 3250 (m) cm^{-1}; **HRMS** (ESI): calcd. for $C_{26}H_{20}O_2Na^+$: 387.1356; found: 387.1360; $\boldsymbol{R_f}$ = 0.14 (isohexane/ethyl acetate – 6/1).

Diastereomer 2:

^{1}H NMR (300 MHz, $CDCl_3$) δ = 7.75 (s, 2H), 7.74–7.68 (m, 2H), 7.48–7.36 (m, 2H), 7.24–7.08 (m, 10H), 5.90 (s, 2H) ppm; **^{13}C NMR** (101 MHz, $CDCl_3$) δ = 141.2, 140.7, 138.8, 138.0, 130.0, 129.1, 128.5, 128.1, 126.8, 125.7, 69.6 ppm; **IR** (ATR) $\tilde{\nu}$ = 697 (s), 757 (s), 895 (m), 977 (m), 1044 (m), 1148 (m), 1211 (m), 1252 (m), 1308 (m), 1375 (m), 1409 (m), 1472 (m), 2851 (m), 2919 (m), 3340 (m) cm^{-1}; **HRMS** (ESI): berechnet für calcd. for $C_{26}H_{20}O_2Na^+$: 387.1356; found: 387.1355; $\boldsymbol{R_f}$ = 0.07 (isohexane/ethyl acetate – 6/1).

4.1.14 Preparation of 5-(3-((*tert*-Butyldimethylsilyl)oxy)propyl)-2-tosylisoindoline **19**

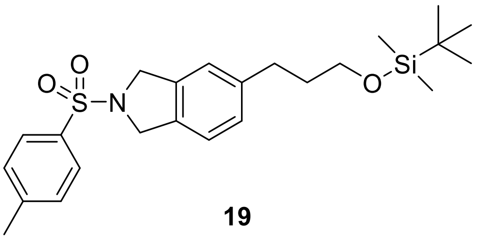

19

According to GP1, $FeH(CO)(NO)(PPh_3)_2$ **1** (12.8 mg, 20.0 µmol, 4 mol%) and Alkyne **S21** (992 mg, 5.00 mmol, 10 eq.) was dissolved in anhydrous 1,4-dioxane (0.5 mL). Diyne **9** (124 mg, 500 µmol, 1.0 eq.) in anhydrous 1,4-dioxane (0.5 mL) was slowly added at 60 °C. The reaction mixture was stirred for 5 minutes at 60 °C. After solvent evaporation and purification by silica gel column chromatography isohexane/ethyl acetate – 10/1), the product **19** was obtained as a brown solid in a yield of 196 mg (440 µmol, 88%).

^{1}H NMR (300 MHz, $CDCl_3$) δ = 7.83–7.70 (m, 2H), 7.38–7.28 (m, 2H), 7.06 (s, 2H), 6.99 (s, 1H), 4.58 (s, 4H), 3.59 (t, J = 6.2 Hz, 2H), 2.72 – 2.56 (m, 2H), 2.40 (s, 3H), 1.86–1.68 (m, 2H), 0.89 (s, 9H), 0.03 (s, 6H) ppm; **^{13}C NMR** (75 MHz, $CDCl_3$) δ = 143.7, 142.3, 136.4, 134.0, 133.6, 129.9, 128.2, 127.8, 122.7, 122.5, 62.2, 53.8, 53.7, 34.7, 32.0, 26.1, 21.6, 18.4, −5.2 ppm; **IR** (ATR) $\tilde{\nu}$ = 667 (s), 775 (m), 835 (m), 1066 (m), 1096 (s), 1163 (s), 1252 (m), 1349 (m), 2855 (m), 2926 (m) cm^{-1}; **HRMS** (ESI): calcd. for $C_{24}H_{35}NO_3SSiNa^+$: 468.1999; found: 468.2006; $\boldsymbol{R_f}$ = 0.46 (isohexane/ethyl acetate – 6/1).

4.1.15 Preparation of 5-(3-Chloropropyl)-2-tosylisoindoline **20**

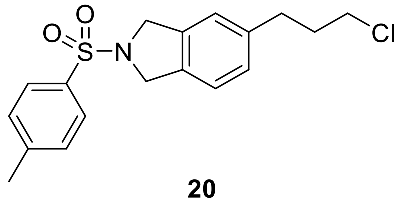

20

According to GP1, $FeH(CO)(NO)(PPh_3)_2$ **1** (12.8 mg, 20.0 µmol, 4 mol%) and 5-chloro-1-pentyne (0.53 mL, 5.00 mmol, 10 eq.) was dissolved in anhydrous 1,4-dioxane (0.5 mL). Diyne **2** (124 mg, 500 µmol, 1.0 eq.) in anhydrous 1,4-dioxane (0.5 mL) was slowly added at 60 °C. The reaction mixture was stirred for 5 minutes at 60 °C. After solvent evaporation and purification by silica gel column chromatography isohexane/ethyl acetate – 8/1), the product **20** was obtained as a brown oil in a yield of 99.1 mg (283 µmol, 56%).

^{1}H NMR (400 MHz, $CDCl_3$) δ = 7.79–7.74 (m, 2H), 7.33–7.28 (m, 2H), 7.12–7.03 (m, 2H), 7.00 (s, 1H), 4.58 (s, 4H), 3.48 (t, J = 6.4 Hz, 2H), 2.74 (t, J = 7.4 Hz, 2H), 2.40 (s, 3H), 2.08–1.97

(m, 2H) ppm; **^{13}C NMR** (101 MHz, $CDCl_3$) δ = 143.8, 140.7, 136.6, 134.1, 133.9, 129.9, 128.2, 127.7, 122.8, 122.8, 53.7, 53.6, 44.1, 34.2, 32.6, 21.6 ppm; **IR** (ATR) $\tilde{\nu}$ = 731 (m), 772 (w), 816 (m), 909 (w), 1059 (m), 1096 (s), 1159 (s), 1342 (m), 1442 (w), 1495 (w), 1595 (w), 1733 (w), 2855 (w), 2922 (w), 2922 (w) cm^{-1}; **HRMS** (ESI): calcd. for $C_{23}H_{23}NO_2SNa^+$: 372.0795; found: 372.0800; $\boldsymbol{R_f}$ = 0.14 (isohexane/ethyl acetate – 10/1).

4.1.16 Preparation of 4-(2-Tosylisoindolin-5-yl)butanenitrile **21**

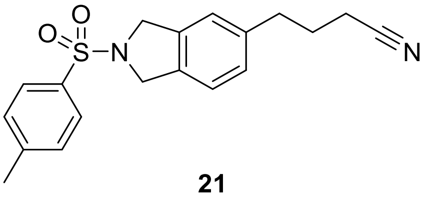

21

According to GP1, $FeH(CO)(NO)(PPh_3)_2$ **1** (12.8 mg, 20.0 µmol, 4 mol%) and 5-hexynenitril (0.52 mL, 5.0 mmol, 10 eq.) was dissolved in anhydrous 1,4-dioxane (0.5 mL). Diyne **2** (124 mg, 500 µmol, 1.0 eq.) in anhydrous 1,4-dioxane (0.5 mL) was slowly added at 60 °C. The reaction mixture was stirred for 5 minutes at 60 °C. After solvent evaporation and purification by silica gel column chromatography isohexane/ethyl acetate – 2/1), the product **21** was obtained as a black solid in a yield of 155 mg (455 µmol, 91%).

^{1}H NMR (400 MHz, $CDCl_3$) δ = 7.80–7.72 (m, 2H), 7.33–7.29 (m, 2H), 7.11 (d, J = 7.8 Hz, 1H), 7.05 (dd, J = 7.8, 1.3 Hz, 1H), 6.99 (s, 1H), 4.58 (s, 4H), 2.74 (t, J = 7.5 Hz, 2H), 2.40 (s, 3H), 2.29 (t, J = 7.0 Hz, 2H), 2.01–1.85 (m, 2H) ppm; **^{13}C NMR** (101 MHz, $CDCl_3$) δ = 143.8, 139.7, 136.9, 134.6, 133.8, 129.9, 128.1, 127.7, 123.0, 122.7, 119.4, 53.7, 53.6, 34.3, 27.1, 21.6, 16.5 ppm; **IR** (ATR) $\tilde{\nu}$ = 663 (s), 708 (m), 731 (m), 768 (w), 816 (m), 909 (m), 1059 (m), 1096 (m), 1159 (s), 1342 (m), 1457 (w), 1495 (w), 1595 (w), 1692 (w), 2855 (w), 2922 (w) cm^{-1}; **HRMS** (ESI): calcd. for $C_{19}H_{20}N_2O_2SNa^+$: 363.1138; found: 363.1141; $\boldsymbol{R_f}$ = 0.29 (isohexane/ethyl acetate – 2/1).

4.1.17 Preparation of 5-(4-Fluorophenyl)-6-phenyl-2-tosylisoindoline **22**

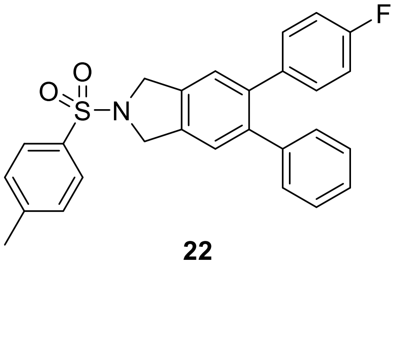

22

According to GP1, $FeH(CO)(NO)(PPh_3)_2$ **1** (12.8 mg, 20.0 µmol, 4 mol%) 1-fluoro-4-(2-phenylethynyl)benzene **S44** (981 mg, 5.00 mmol, 10 eq.) was dissolved in anhydrous 1,4-dioxane (0.5 mL). Diyne **2** (124 mg, 500 µmol, 1.0 eq.) in anhydrous 1,4-dioxane (0.5 mL) was slowly added at 60 °C. The reaction mixture was stirred for 5 minutes at 60 °C. After solvent evaporation and purification by silica gel column chromatography isohexane/ethyl acetate – 8/1), the product **22** was obtained as a colourless solid in a yield of 178 mg (402 µmol, 80%).

^{1}H NMR (600 MHz, $CDCl_3$) δ = 7.82–7.79 (m, 2H), 7.35–7.32 (m, 2H), 7.22–7.17 (m, 5H), 7.04–6.97 (m, 4H), 6.87 (t, J = 8.8 Hz, 2H), 4.69 (s, 4H), 2.42 (s, 3H) ppm; **^{13}C NMR** (151 MHz, $CDCl_3$) δ = 162.0 (d, J = 246.3 Hz), 143.9, 140.9, 140.8, 139.7, 137.0 (d, J = 3.4 Hz), 135.8, 135.7, 133.8, 131.4 (d, J = 8.0 Hz), 130.0, 129.9, 128.2, 127.8, 127.0, 124.8, 124.7, 115.1 (d, J = 21.4 Hz), 53.7, 53.7, 21.7 ppm; **IR** (ATR) $\tilde{\nu}$ = 775 (w), 839 (m), 909 (w), 1018 (w), 1062 (w), 1096 (m), 1163 (s), 1223 (m), 1349 (m), 1480 (w), 1513 (w), 1603 (w), 2855 (w), 2922 (w), 3053 (w), 3053 (w) cm^{-1}; **HRMS** (ESI): calcd. for $C_{27}H_{22}FNO_2SNa^+$: 466.1247; found: 466.1250; ***R***$_f$ = 0.09 (isohexane/ethyl acetate –10/1).

4.1.18 Preparation of 5-(4-Chlorophenyl)-6-phenyl-2-tosylisoindoline **23**

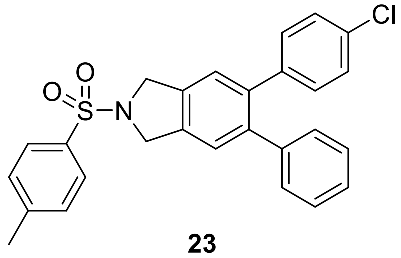

23

According to GP1, $FeH(CO)(NO)(PPh_3)_2$ **1** (12.8 mg, 20.0 µmol, 4 mol%) 1-chloro-4-(2-phenylethynyl)benzene **45** (1.06 g, 5.00 mmol, 10 eq.) was dissolved in anhydrous 1,4-dioxane (0.5 mL). Diyne **2** (124 mg, 0500 µmol, 1.0 eq.) in anhydrous 1,4-dioxane (0.5 mL) was slowly added at 60 °C. The reaction mixture was stirred for 5 minutes at 60 °C. After solvent evaporation and purification by silica gel column chromatography isohexane/ethyl acetate – 8/1), the product **23** was obtained as a yellow solid in a yield of 177 mg (384 µmol, 77%).

^{1}H NMR (400 MHz, $CDCl_3$) δ = 7.83–7.78 (m, 2H), 7.36–7.31 (m, 2H), 7.24–7.11 (m, 7H), 7.05–7.00 (m, 2H), 6.99–6.94 (m, 2H), 4.69 (s, 4H), 2.42 (s, 3H) ppm; **^{13}C NMR** (101 MHz, $CDCl_3$) δ = 143.9, 140.7, 140.7, 139.5, 139.4, 136.0, 135.8, 133.7, 133.0, 131.2, 130.0, 129.8, 128.3, 128.3, 127.8, 127.1, 124.9, 124.6, 53.7, 53.7, 21.7 ppm; **IR** (ATR) $\tilde{\nu}$ = 663 (s), 727 (s), 775 (m), 831 (m), 906 (m), 1088 (s), 1159 (s), 1346 (m), 1476 (m), 1595 (m), 1733 (w), 1770 (w), 2851 (w), 2922 (w), 3030 (w) cm^{-1}; **HRMS** (ESI): calcd. for $C_{27}H_{22}ClNO_2SNa^+$: 482.0952; found: 482.0959; $\boldsymbol{R_f}$ = 0.14 (isohexane/ethyl acetate –10/1).

4.1.19 Preparation of 5-Phenyl-6-(*p*-tolyl)-2-tosylisoindoline **24**

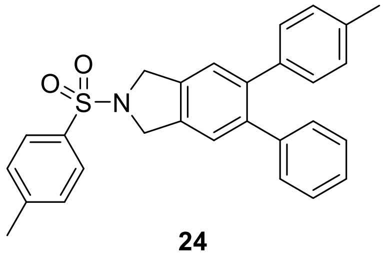

24

According to GP1, $FeH(CO)(NO)(PPh_3)_2$ **1** (12.8 mg, 20.0 µmol, 4 mol%) 1-methyl-4-(2-phenylethynyl)benzene **S46** (961 mg, 5.00 mmol, 10 eq.) was dissolved in anhydrous 1,4-dioxane (0.5 mL). Diyne **2** (124 mg, 500 µmol, 1.0 eq.) in anhydrous 1,4-dioxane (0.5 mL) was slowly added at 60 °C. The reaction mixture was stirred for 5 minutes at 60 °C. After solvent evaporation and purification by silica gel column chromatography isohexane/ethyl acetate –10/1), the product **24** was obtained as a brown oil in a yield of 160 mg (365 µmol, 73%).

^{1}H NMR (400 MHz, $CDCl_3$) δ = 7.84–7.79 (m, 2H), 7.37–7.32 (m, 2H), 7.23–7.17 (m, 5H), 7.09–7.05 (m, 2H), 7.02–6.98 (m, 2H), 6.97–6.92 (m, 2H), 4.70 (s, 4H), 2.43 (s, 3H), 2.29 (s, 3H) ppm; **^{13}C NMR** (101 MHz, $CDCl_3$) δ = 143.9, 141.2, 140.7, 140.7, 138.0, 136.5, 135.6, 135.3, 133.7, 130.0, 129.9, 129.7, 128.8, 128.1, 127.8, 126.8, 124.7, 124.7, 53.7, 53.7, 21.6, 21.2 ppm; **IR** (ATR) $\tilde{\nu}$ = 663 (m), 723 (s), 775 (w), 820 (w), 902 (s), 1066 (w), 1096 (m), 1163 (m), 1346 (w) cm^{-1}; **HRMS** (ESI): calcd. for $C_{28}H_{25}NO_2SNa^+$: 462.1498; found: 462.1497; $\boldsymbol{R_f}$ = 0.20 (isohexane/ethyl acetate –10/1).

4.1.20 Preparation of 5-Phenyl-2-tosyl-6-(4-(trifluoromethyl)phenyl)isoindoline **25**

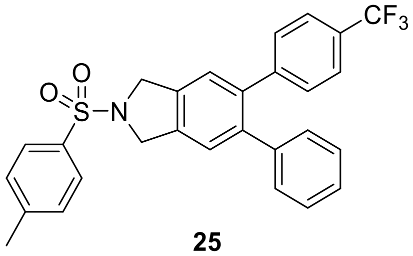

25

According to GP1, $FeH(CO)(NO)(PPh_3)_2$ **1** (12.8 mg, 20.0 µmol, 4 mol%) 1-(2-phenylethynyl)-4-(trifluoromethyl)benzene **S47** (1.22 g, 5.00 mmol, 10 eq.) was dissolved in anhydrous 1,4-dioxane (0.5 mL). Diyne **2** (124 mg, 500 µmol, 1.0 eq.) in anhydrous 1,4-dioxane (0.5 mL) was slowly added at 60 °C. The reaction mixture was stirred for 5 minutes at 60 °C. After solvent evaporation and purification by silica gel column chromatography isohexane/ethyl acetate –10/1), the product **25** was obtained as a brown solid in a yield of 200 mg (405 µmol, 81%).

^{1}H NMR (400 MHz, $CDCl_3$) δ = 7.84–7.79 (m, 2H), 7.44 (d, J = 8.1 Hz, 2H), 7.35 (d, J = 8.0 Hz, 2H), 7.25 (s, 1H), 7.23–7.15 (m, 6H), 7.06–7.01 (m, 2H), 4.71 (s, 4H), 2.42 (s, 3H) ppm; **^{13}C NMR** (101 MHz, $CDCl_3$) δ = 144.8 (q, J = 1.3 Hz), 144.0, 140.8, 140.4, 139.2, 136.5, 135.9, 133.7, 130.2, 130.0, 129.8, 129.0 (q, J = 32.8 Hz), 128.3, 127.8, 127.2, 125.0 (q, J = 3.8 Hz), 125.0, 124.3 (q, J = 272.2 Hz), 124.7, 53.7, 53.7, 21.7 ppm; **IR** (ATR) $\tilde{\nu}$ = 663 (s), 727 (s), 775 (m), 842 (m), 906 (s), 1018 (m), 1066 (s), 1107 (m), 1163 (s), 1323 (s), 1401 (w), 1472 (w), 1618 (w), 2851 (w), 2922 (w), 3030 (w) cm^{-1}; **HRMS** (ESI): calcd. for $C_{28}H_{22}F_3NO_2SNa^+$: 516.1216; found: 516.1217; ***R*$_f$** = 0.20 (isohexane/ethyl acetate –10/1).

4.1.21 Preparation of 5-(4-Methoxyphenyl)-6-phenyl-2-tosylisoindoline **26**

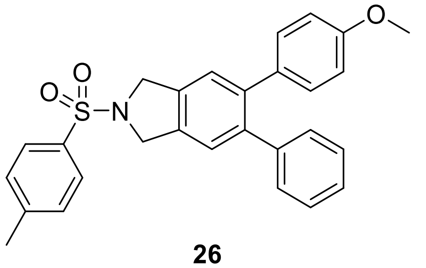

26

According to GP1, $FeH(CO)(NO)(PPh_3)_2$ **1** (12.8 mg, 20.0 µmol, 4 mol%) 1-methoxy-4-(2-phenylethynyl)benzene **S43** (1.04 g, 5.00 mmol, 10 eq.) was dissolved in anhydrous 1,4-dioxane (0.5 mL). Diyne **2** (124 mg, 500 µmol, 1.0 eq.) in anhydrous 1,4-dioxane (0.5 mL) was slowly added at 60 °C. The reaction mixture was stirred for 5 minutes at 60 °C. After solvent evaporation and purification by silica gel column chromatography isohexane/ethyl acetate – 5/1), the product **26** was obtained as a colourless solid in a yield of 157 mg (345 µmol, 69%).

^{1}H NMR (400 MHz, $CDCl_3$) δ = 7.84–7.76 (m, 2H), 7.37–7.29 (m, 2H), 7.22–7.15 (m, 5H), 7.09–7.01 (m, 2H), 6.99–6.91 (m, 2H), 6.77–6.65 (m, 2H), 4.69 (s, 4H), 3.76 (s, 3H), 2.42 (s, 3H) ppm; **^{13}C NMR** (101 MHz, $CDCl_3$) δ = 158.6, 143.9, 141.3, 140.6, 140.3, 135.6, 135.2, 133.8, 133.4, 131.0, 130.0, 129.9, 128.1, 127.8, 126.8, 124.8, 124.7, 113.6, 55.3, 53.8, 53.7, 21.7 ppm; **IR** (ATR) $\tilde{\nu}$ = 727 (s), 775 (m), 831 (m), 909 (m), 1029 (m), 1062 (m), 1096 (m), 1159 (s), 1245 (m), 1290 (m), 1346 (m), 1469 (m), 1517 (m), 1606 (m), 2837 (w), 2922 (w), 3027 (w) cm^{-1}; **HRMS** (ESI): calcd. for $C_{28}H_{25}NO_3SNa^+$: 478.1447; found: 478.1454; ***R*$_f$** = 0.34 (isohexane/ethyl acetate –5/1).

4.1.22 Preparation of methyl 4-(6-phenyl-2-tosylisoindolin-5-yl)benzoate **27**

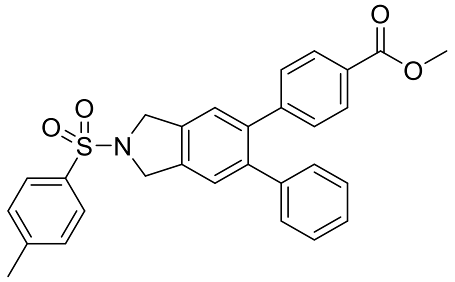

27

According to GP1, FeH(CO)(NO)(PPh$_3$)$_2$ **1** (12.8 mg, 20.0 µmol, 4 mol%) methyl-4-(phenylethynyl)benzoate **S48** (1.18 g, 5.00 mmol, 10 eq.) was dissolved in anhydrous 1,4-dioxane (0.5 mL). Diyne **2** (124 mg, 500 µmol, 1.0 eq.) in anhydrous 1,4-dioxane (0.5 mL) was slowly added at 60 °C. The reaction mixture was stirred for 5 minutes at 60 °C. After solvent evaporation and purification by silica gel column chromatography isohexane/ethyl acetate – 5/1), the product **27** was obtained as a colourless foam in a yield of 173 mg (357 µmol, 71%).

^{1}H NMR (300 MHz, CDCl$_3$) δ = 7.86–7.77 (m, 2H), 7.35 (d, J = 8.0 Hz, 2H), 7.30 (s, 1H), 7.19–7.06 (m, 6H), 7.00–6.89 (m, 4H), 4.78 (s, 2H), 4.71 (s, 2H), 3.49 (s, 3H), 2.43 (s, 3H) ppm; **^{13}C NMR** (75 MHz, CDCl$_3$) δ = 167.1, 145.9, 143.9, 140.9, 140.6, 139.7, 136.4, 135.8, 133.7, 130.0, 129.9, 129.8, 129.4, 128.5, 128.3, 127.8, 127.1, 124.9, 124.6, 53.7, 53.7, 52.2, 21.7 ppm; **IR** (ATR) $\tilde{\nu}$ = 663 (s), 727 (s), 775 (m), 816 (m), 857 (m), 909 (m), 965 (w), 1018 (m), 1062 (m), 1096 (s), 1163 (s), 1275 (s),1346 (m), 1398 (w), 1435 (m), 1472 (w), 1606 (m), 1718 (m), 2848 (w), 2922 (w), 2948 (w), 2993 (w), 3027 (w) cm^{-1}; **HRMS** (ESI): calcd. for $C_{29}H_{25}NO_4SNa^+$: 506.1397; found: 506.1340; $\boldsymbol{R_f}$ = 0.19 (isohexane/ethyl acetate –5/1).

4.1.23 Preparation of 4-methyl-5,6-diphenyl-2-tosylisoindoline **28**

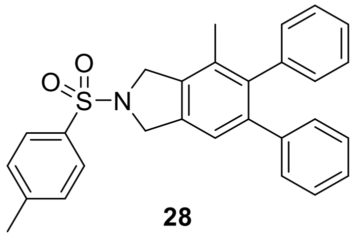

28

According to GP1, FeH(CO)(NO)(PPh$_3$)$_2$ **1** (12.8 mg, 20.0 µmol, 4 mol%) and diphenylacetylene **3** (891 mg, 5.00 mmol, 10 eq.) was dissolved in anhydrous 1,4-dioxane (0.5 mL). Diyne **S27** (131 mg, 500 µmol, 1.0 eq.) in anhydrous 1,4-dioxane (0.5 mL) was slowly added at 60 °C. The reaction mixture was stirred for 5 minutes at 60 °C. After solvent evaporation and purification by silica gel column chromatography isohexane/ethyl acetate – 10/1), the product **28** was obtained as a yellow solid in a yield of 176 mg (401 µmol, 80%).

^{1}H NMR (300 MHz, $CDCl_3$) δ = 7.86–7.79 (m, 2H), 7.40–7.32 (m, 2H), 7.23–7.14 (m, 3H), 7.13–7.04 (m, 4H), 7.02–6.89 (m, 4H), 4.73 (s, 2H), 4.66 (s, 2H), 2.43 (s, 3H), 2.00 (s, 3H) ppm; **^{13}C NMR** (101 MHz, $CDCl_3$) δ = 143.8, 142.1, 141.7, 140.3, 139.5, 135.0, 134.95, 133.9, 131.4, 130.6, 130.0, 129.8, 127.9, 127.8, 127.7, 126.7, 126.4, 121.6, 54.3, 53.7, 21.7, 17.5 ppm; **IR** (in $CDCl_3$) $\tilde{\nu}$ = 667 (s), 731 (s), 772 (m), 813 (m), 909 (m), 1066 (m), 1096 (m), 1163 (s), 1346 (m), 1413 (w), 1461 (w), 1491 (w), 1595 (w), 2848 (w), 2919 (w), 3027 (w), 3056 (w) cm^{-1}; **HRMS** (ESI): calcd. for $C_{28}H_{25}NO_2SNa^+$: 462.1498; found: 462.1503; ***R*f** = 0.41 (isohexane/ethyl acetate – 5/1).

4.1.24 Preparation of 4-isopropyl-5,6-diphenyl-2-tosylisoindoline **29**

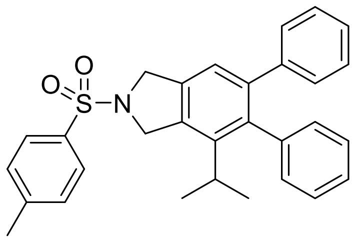

29

According to GP1, $FeH(CO)(NO)(PPh_3)_2$ **1** (12.8 mg, 20.0 µmol, 4 mol%) and diphenylacetylene **3** (891 mg, 5.00 mmol, 10 eq.) was dissolved in anhydrous 1,4-dioxane (0.5 mL). Diyne **S16** (145 mg, 500 µmol, 1.0 eq.) in anhydrous 1,4-dioxane (0.5 mL) was slowly added at 60 °C. The reaction mixture was stirred for 5 minutes at 60 °C. After solvent evaporation and purification by silica gel column chromatography isohexane/ethyl acetate – 10/1), the product **29** was obtained as a brown solid in a yield of 198 mg (422 µmol, 84%).

^{1}H NMR (400 MHz, $CDCl_3$) δ = 7.91–7.77 (m, 2H), 7.39–7.31 (m, 2H), 7.19–6.83 (m, 11H), 4.84 (s, 2H), 4.62 (s, 2H), 3.10–2.85 (m, 1H), 2.44 (s, 3H), 1.14–0.99 (m, 6H) ppm; **^{13}C NMR** (101 MHz, $CDCl_3$) δ = 143.9, 142.5, 141.9, 141.6, 140.1, 140.0, 136.6, 133.6, 133.1, 130.6, 130.0, 129.7, 127.9, 127.7, 127.5, 126.6, 126.3, 121.8, 53.52, 53.0, 30.6, 21.7, 21.3 ppm; **IR** (ATR) $\tilde{\nu}$ = 667 (s), 701 (s), 731 (s), 768 (m), 813 (m), 909 (m), 1029 (w), 1096 (m), 1163 (s), 1245 (w), 1346 (m), 1405 (w), 1442 (w), 1495 (w), 1599 (w), 1737 (w), 2870 (w), 2926 (w), 2963 (w), 3056 (w) cm^{-1}; **HRMS** (ESI): calcd. for $C_{30}H_{29}NO_2SNa^+$: 490.1811; found: 490.1806; ***R*f** = 0.34 (isohexane/ethyl acetate – 10/1).

4.1.25 Preparation of methyl 5,6-diphenyl-2-tosylisoindoline-4-carboxylate **30**

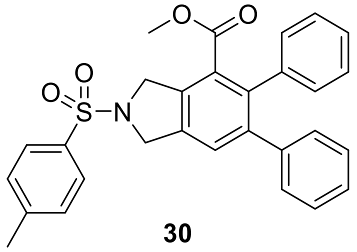

30

According to GP1, $FeH(CO)(NO)(PPh_3)_2$ **1** (12.8 mg, 20.0 µmol, 4 mol%) and diphenlacetylene **3** (891 g, 5.00 mmol, 10 eq.) was dissolved in anhydrous 1,4-dioxane (0.5 mL). Diyne **S8** (153 mg, 500 µmol, 1.0 eq.) in anhydrous 1,4-dioxane (0.5 mL) was slowly added at 60 °C. The reaction mixture was stirred for 20 minutes at 60 °C. After solvent evaporation and purification by silica gel column chromatography isohexane/ethyl acetate – 10/1), the product **30** was obtained as a colourless solid in a yield of 181 mg (374 µmol, 75%).

^{1}H NMR (300 MHz, $CDCl_3$) δ = 7.87–7.75 (m, 2H), 7.40–7.32 (m, 2H), 7.30 (s, 1H), 7.20–7.07 (m, 6H), 7.03–6.88 (m, 4H), 4.78 (s, 2H), 4.71 (s, 2H), 3.49 (s, 3H), 2.43 (s, 3H) ppm; **^{13}C NMR** (75 MHz, $CDCl_3$) δ = 168.3, 144.0, 142.4, 140.4, 139.6, 138.9, 136.2, 135.3, 133.7, 130.1, 129.9, 129.8, 128.5, 127.9, 127.8, 127.8, 127.1, 126.9, 126.6, 53.9, 53.6, 52.2, 21.7 ppm; **IR** (ATR) $\tilde{\nu}$ = 667 (s), 727 (s), 813 (m), 909 (m), 1003 (m), 1066 (m), 1096 (m), 1163 (s), 1208 (m), 1286 (m), 1349 (m), 1454 (w), 1495 (w), 1599 (w), 1718 (m), 2851 (w), 2922 (w) cm^{-1}; **HRMS** (ESI): calcd. for $C_{29}H_{26}NO_4S^+$: 484.1577; found: 484.1579; ***R*$_f$** = 0.29 (isohexane/ethyl acetate – 5/1).

4.1.26 Preparation of 4-(methoxymethyl)-5,6-diphenyl-2-tosylisoindoline **31**

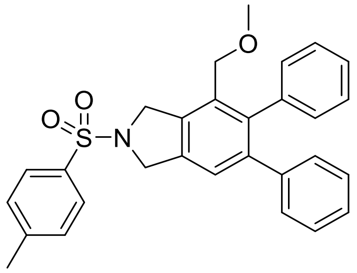

31

According to GP1, $FeH(CO)(NO)(PPh_3)_2$ **1** (12.8 mg, 20.0 µmol, 4 mol%) diphenylacetylene **10** (891 mg, 5.00 mmol, 10 eq.) was dissolved in anhydrous 1,4-dioxane (0.5 mL). Diyne **S9** (146 mg, 500 µmol, 1.0 eq.) in anhydrous 1,4-dioxane (0.5 mL) was slowly added at 60 °C. The reaction mixture was stirred for 5 minutes at 60 °C. After solvent evaporation and purification by silica gel column chromatography (isohexane/ethyl acetate – 5/1), the product **31** was obtained as a colourless solid in a yield of 169 mg (361 µmol, 72%).

^{1}**H NMR** (400 MHz, $CDCl_3$) δ = 7.85–7.77 (m, 2H), 7.38–7.30 (m, 2H), 7.20–7.13 (m, 4H), 7.11–7.05 (m, 3H), 7.00–6.95 (m, 2H), 6.96–6.90 (m, 2H), 4.80 (s, 2H), 4.69 (s, 2H), 4.12 (s, 2H), 3.22 (s, 3H), 2.43 (s, 3H) ppm; ^{13}**C NMR** (101 MHz, $CDCl_3$) δ = 143.8, 142.2, 141.4, 140.5, 138.5, 136.0, 135.9, 133.9, 131.2, 130.7, 130.0, 129.8, 127.8, 127.7, 127.7, 127.0, 126.5, 123.9, 70.6, 58.6, 53.7, 53.4, 21.7 ppm; **IR** (ATR) $\tilde{\nu}$ = 701 (m), 775 (w), 835 (w), 1062 (w), 1096 (m), 1163 (m), 1342 (w), 1454 (w), 1599 (w), 2822 (w), 2855 (w), 2930 (w) cm^{-1}; **HRMS** (ESI): calcd. for $C_{29}H_{27}NO_3SK^+$: 508.1343; found: 508.1342; $\boldsymbol{R_f}$ = 0.23 (isohexane/ethyl acetate – 5/1).

4.1.27 Preparation of 4-(3-((tert-butyldimethylsilyl)oxy)propyl)-5,6-diphenyl-2-tosylisoindoline **32**

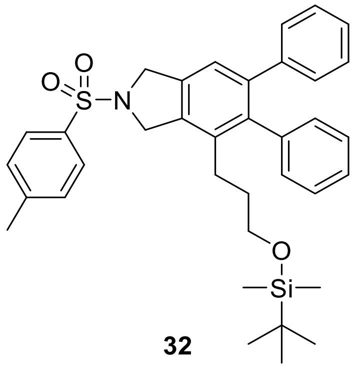

32

According to GP1, $FeH(CO)(NO)(PPh_3)_2$ **1** (12.8 mg, 20.0 µmol, 4 mol%) and diphenylacetylene **3** (891 mg, 5.00 mmol, 10 eq.) was dissolved in anhydrous 1,4-dioxane (0.5 mL). Diyne **S23** (210 mg, 500 µmol, 1.0 eq.) in anhydrous 1,4-dioxane (0.5 mL) was slowly added at 60 °C. The reaction mixture was stirred for 5 minutes at 60 °C. After solvent evaporation and purification by silica gel column chromatography isohexane/ethyl acetate – 10/1), the product **32** was obtained as a brown oil in a yield of 292 mg (488 µmol, 98%).

^{1}**H NMR** (300 MHz, $CDCl_3$) δ = 7.87–7.77 (m, 2H), 7.40–7.31 (m, 2H), 7.20–7.12 (m, 3H), 7.11–7.04 (m, 4H), 6.99–6.89 (m, 4H), 4.76–4.62 (m, 4H), 3.39 (t, J = 6.2 Hz, 2H), 2.47–2.32 (m, 5H), 1.51–1.38 (m, 2H), 0.85 (s, 9H), −0.03 (s, 6H) ppm; ^{13}**C NMR** (75 MHz, $CDCl_3$) δ = 143.8, 142.4, 141.7, 140.3, 139.2, 135.9, 135.4, 134.7, 133.9, 130.7, 130.0, 129.8, 127.8, 127.8, 127.6, 126.7, 126.4, 121.8, 62.8, 54.1, 53.3, 33.2, 27.9, 26.1, 21.7, 18.4, −5.2 ppm; **IR** (ATR) $\tilde{\nu}$ =667 (m), 701 (m), 753 (w), 775 (m), 835 (m), 909 (w), 962 (w), 1096 (s), 1163 (s), 1252 (w), 1349 (m), 1387 (w), 1416 (w), 1461 (w), 1495 (w), 1599 (w), 2359 (w), 2855 (w), 2952 (m), 2952 (m) cm^{-1}; **HRMS** (ESI): calcd. for $C_{36}H_{43}NO_3SSiNa^+$: 620.2625; found: 620.2624; $\boldsymbol{R_f}$ = 0.21 (isohexane/ethyl acetate – 10/1).

4.1.28 Preparation of 3-(5,6-diphenyl-2-tosylisoindolin-4-yl)propan-1-ol **33**

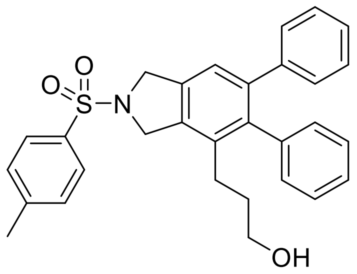

33

According to GP1, FeH(CO)(NO)(PPh$_3$)$_2$ **1** (12.8 mg, 20.0 µmol, 4 mol%) and diphenylacetylene **3** (891 mg, 5.00 mmol, 10 eq.) was dissolved in anhydrous 1,4-dioxane (0.5 mL). Diyne **S38** (153 mg, 500 µmol, 1.0 eq.) in anhydrous 1,4-dioxane (0.5 mL) was slowly added at 60 °C. The reaction mixture was stirred for 5 minutes at 60 °C. After solvent evaporation and purification by silica gel column chromatography isohexane/ethyl acetate – 10/1 to 2/1), the product **33** was obtained as a colourless foam in a yield of 217 mg (448 µmol, 90%).

^{1}H NMR (300 MHz, CDCl$_3$) δ = 7.88–7.76 (m, 2H), 7.44–7.28 (m, 2H), 7.23–7.14 (m, 3H), 7.12–7.04 (m, 4H), 7.01–6.91 (m, 4H), 4.71 (s, 4H), 3.39 (t, J = 6.2 Hz, 2H), 2.54–2.38 (m, 5H), 1.56–1.43 (m, 2H) ppm; **^{13}C NMR** (75 MHz, CDCl$_3$) δ = 143.9, 142.5, 141.6, 140.3, 139.1, 135.5, 135.4, 134.7, 133.9, 130.7, 130.0, 129.8, 127.9, 127.8, 127.6, 126.9, 126.5, 122.0, 62.3, 54.1, 53.3, 32.8, 27.4, 21.7 ppm; **IR** (ATR) $\tilde{\nu}$ = 663(s), 701 (s), 731 (s), 772 (m), 813 (m), 909 (m), 1062 (m), 1096 (m), 1163 (s), 1342 (m), 1416 (w), 1457 (m), 1495(w), 1599 (w), 2870 (w), 2922 (w), 3027 (w), 3056 (w), 3526 (w), 3526 (w) cm^{-1}; **HRMS** (ESI): calcd. for $C_{30}H_{29}NO_3SNa^+$: 506.1760; found: 506.1762; $\boldsymbol{R}_\mathbf{f}$ = 0.46 (isohexane/ethyl acetate – 1/1).

4.1.29 Preparation of 4-(3-bromopropyl)-5,6-diphenyl-2-tosylisoindoline **34**

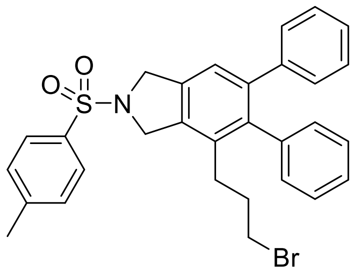

34

According to GP1, FeH(CO)(NO)(PPh$_3$)$_2$ **1** (12.8 mg, 20.0 µmol, 4 mol%) and diphenylacetylene **3** (891 mg, 5.00 mmol, 10 eq.) was dissolved in anhydrous 1,4-dioxane (0.5 mL). Diyne **S39** (184 mg, 500 µmol, 1.0 eq.) in anhydrous 1,4-dioxane (0.5 mL) was slowly added at 60 °C.

The reaction mixture was stirred for 20 minutes at 60 °C. After solvent evaporation and purification by silica gel column chromatography isohexane/ethyl acetate – 10/1), the product **34** was obtained as a brown oil in a yield of 200 mg (365 µmol, 73%).

^{1}H NMR (600 MHz, $CDCl_3$) δ = 7.96–7.76 (m, 2H), 7.43–7.33 (m, 2H), 7.23–7.12 (m, 3H), 7.11–7.06 (m, 4H), 6.99–6.89 (m, 4H), 4.83–4.55 (m, 4H), 3.14 (t, J = 6.5 Hz, 2H), 2.60–2.50 (m, 2H), 2.44 (s, 3H), 1.82–1.71 (m, 2H) ppm; **^{13}C NMR** (151 MHz, $CDCl_3$) δ = 143.9, 142.5, 141.4, 140.4, 138.9, 135.6, 134.7, 134.3, 133.8, 130.6, 130.1, 129.7, 128.0, 127.8, 127.6, 126.9, 126.5, 122.3, 54.1, 53.3, 33.2, 32.7, 30.0, 21.7 ppm; **IR** (ATR) $\tilde{\nu}$ = 701 (s), 731 (m), 772 (m), 816 (w), 909 (m), 1066 (m), 1096 (m), 1163 (s), 1245 (w), 1346 (m), 1457 (w), 1495 (w), 1599 (w), 2863 (w), 2922 (w), 2952 (w), 3056 (w) cm^{-1}; **HRMS** (ESI): calcd. for $C_{30}H_{29}BrNO_2S^+$: 546.1097; found: 546.1087; ***R*f** = 0.22 (isohexane/ethyl acetate –10/1)

4.1.30 Preparation of 4-phenethyl-5,6-diphenyl-2-tosylisoindoline **35**

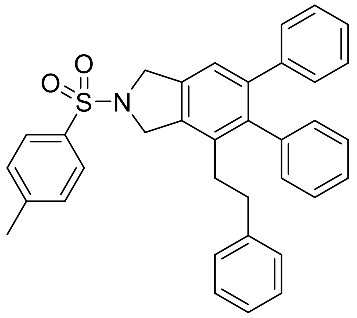

35

According to GP1, $FeH(CO)(NO)(PPh_3)_2$ **1** (12.8 mg, 20.0 µmol, 4 mol%) and diphenylacetylene **3** (891 mg, 5.00 mmol, 10 eq.) was dissolved in anhydrous 1,4-dioxane (0.5 mL). Diyne **S19** (176 mg, 500 µmol, 1.0 eq.) in anhydrous 1,4-dioxane (0.5 mL) was slowly added at 60 °C. The reaction mixture was stirred for 5 minutes at 60 °C. After solvent evaporation and purification by silica gel column chromatography (isohexane/ethyl acetate – 10/1), the product **35** was obtained as a yellow solid in a yield of 221 mg (418 µmol, 84%).

^{1}H NMR (400 MHz, $CDCl_3$) δ = 7.85–7.77 (m, 2H), 7.39–7.32 (m, 2H), 7.24–7.07 (m, 10H), 7.04–6.95 (m, 4H), 6.85–6.76 (m, 2H), 4.71 (s, 2H), 4.68 (s, 2H), 2.72–2.59 (m, 2H), 2.54–2.45 (m, 2H), 2.44 (s, 3H) ppm; **^{13}C NMR** (101 MHz, $CDCl_3$) δ = 143.9, 142.5, 141.6, 141.4, 140.4, 139.0, 135.5, 135.1, 134.7, 133.9, 130.7, 130.0, 129.8, 128.5, 128.3, 127.9, 127.8, 127.7, 126.9, 126.4, 126.2, 122.1, 54.1, 53.3, 36.1, 34.0, 21.7 ppm; **IR** (ATR) $\tilde{\nu}$ = 701 (s), 731 (m), 816 (w), 909 (m), 1066 (m), 1096 (m), 1163 (s), 1349 (m), 1416 (w), 1454 (w), 1495 (w), 1599 (w), 2855 (w), 2922 (w), 3027 (w), 3056 (w) cm^{-1}; **HRMS** (ESI): calcd. for $C_{35}H_{31}NO_2SNa^+$: 552.1968; found: 552.1949; ***R*f** = 0.20 (isohexane/ethyl acetate – 10/1).

4.1.31 Preparation of 4-phenethyl-5,6-diphenyl-1,3-dihydroisobenzofuran **36**

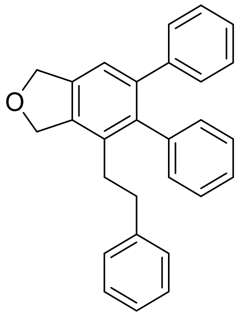

36

According to GP1, FeH(CO)(NO)(PPh_3)$_2$ **1** (12.8 mg, 20.0 µmol, 4 mol%) and diphenylacetylene **3** (891 mg, 5.00 mmol, 10 eq.) was dissolved in anhydrous 1,4-dioxane (0.5 mL). Diyne **S40** (99.1 mg, 500 µmol, 1.0 eq.) in anhydrous 1,4-dioxane (0.5 mL) was slowly added at 60 °C. The reaction mixture was stirred for 5 minutes at 60 °C. After solvent evaporation and purification by silica gel column chromatography (isohexane/ethyl acetate – 20/1), the product **36** was obtained as a brown oil in a yield of 173 mg (460 µmol, 92%).

^{1}H NMR (300 MHz, $CDCl_3$) δ = 7.33–7.03 (m, 14H), 6.91–6.80 (m, 2H), 5.35–5.11 (m, 4H), 2.78–2.66 (m, 2H), 2.65–2.53 (m, 2H) ppm; **^{13}C NMR** (75 MHz, $CDCl_3$) δ = 142.1, 142.0, 141.7, 139.9, 139.5, 138.4, 137.7, 133.8, 130.9, 129.9, 128.5, 128.3, 127.9, 127.6, 126.7, 126.3, 126.1, 120.5, 74.2, 73.2, 36.3, 34.3 ppm; **IR** (ATR) $\tilde{\nu}$ = 697 (s), 727 (s), 906 (m), 1051 (m), 1178 (w), 1305 (w), 1360 (w), 1413 (w), 1454 (w), 1495 (w),1603 (w), 1759 (w),2851 (w), 2930 (w), 3027 (w), 3056 (w) cm^{-1}; **HRMS** (ESI): calcd. for $C_{28}H_{23}O^+$: 375.1743; found: 375.1744; $\boldsymbol{R_f}$ = 0.20 (isohexane/ethyl acetate – 20/1).

4.1.32 Preparation of 4-phenethyl-5,6,7-triphenyl-2-tosylisoindoline **37**

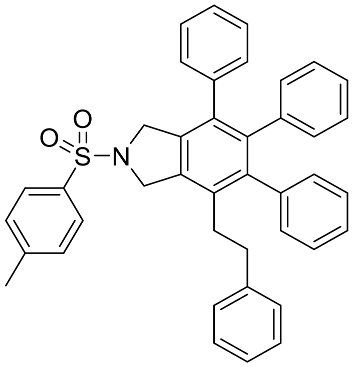

37

According to GP1, FeH(CO)(NO)(PPh_3)$_2$ **1** (12.8 mg, 20.0 µmol, 4 mol%) and diphenylacetylene **3** (891 mg, 5.00 mmol, 10 eq.) was dissolved in anhydrous 1,4-dioxane (0.5 mL). Diyne **S42** (214 mg, 500 µmol, 1.0 eq.) in anhydrous 1,4-dioxane (0.5 mL) was slowly added at 60 °C.

The reaction mixture was stirred for 30 minutes at 60 °C. After solvent evaporation and purification by silica gel column chromatography (isohexane/ethyl acetate – 10/1), the product **37** was obtained as a brown solid in a yield of 284 mg (469 µmol, 94%).

^{1}H NMR (300 MHz, $CDCl_3$) δ = 7.81–7.70 (m, 2H), 7.39–7.30 (m, 2H), 7.24–7.08 (m, 9H), 7.03–6.91 (m, 4H), 6.88–6.76 (m, 5H), 6.74–6.63 (m, 2H), 4.76 (s, 2H), 4.50 (s, 2H), 2.70–2.50 (m, 4H), 2.44 (s, 3H) ppm; **^{13}C NMR** (75 MHz, $CDCl_3$) δ = 143.8, 143.1, 141.9, 141.5, 141.4, 139.4, 139.4, 138.9, 135.0, 134.7, 134.5, 134.0, 133.9, 131.2, 130.5, 130.0, 129.5, 128.5, 128.3, 128.1, 127.8, 127.7, 126.9, 126.6, 126.2, 125.8, 54.3, 53.7, 36.2, 34.2, 21.7 ppm; **IR** (ATR) $\tilde{\nu}$ = 671 (m), 701 (s), 731 (m), 816 (w), 909 (m), 1070 (m), 1096 (m), 1163 (s), 1349 (m), 1446 (w), 1495 (w), 1599 (w), 2859 (w), 2922 (w), 3027 (w), 3056 (w) cm^{-1}; **HRMS** (ESI): calcd. for $C_{41}H_{35}NO_2Na^+$: 628.2281; found: 628.2283; ***R*$_f$** = 0.26 (isohexane/ethyl acetate – 10/1).

4.1.33 Preparation of 4-phenethyl-5,6,7-triphenyl-1,3-dihydroisobenzofuran **38**

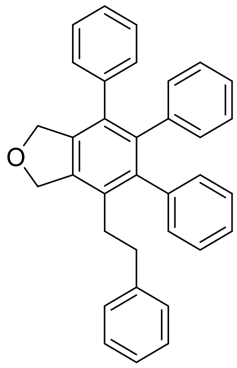

38

According to GP1, $FeH(CO)(NO)(PPh_3)_2$ **1** (12.8 mg, 20.0 µmol, 4 mol%) and diphenylacetylene **3** (891 mg, 5.00 mmol, 10 eq.) was dissolved in anhydrous 1,4-dioxane (0.5 mL). Diyne **S41** (137 mg, 500 µmol, 1.0 eq.) in anhydrous 1,4-dioxane (0.5 mL) was slowly added at 60 °C. The reaction mixture was stirred for 30 minutes at 60 °C. After solvent evaporation and purification by silica gel column chromatography (isohexane/ethyl acetate – 30/1), the product **38** was obtained as a brown foam in a yield of 170 mg (375 µmol, 75%).

^{1}H NMR (600 MHz, $CDCl_3$) δ = 7.21–7.17 (m, 4H), 7.16–7.10 (m, 5H), 7.08–7.05 (m, 2H), 7.05–7.01 (m, 2H), 6.88–6.83 (m, 5H), 6.79–6.77 (m, 2H), 5.28 (s, 2H), 5.05 (s, 2H), 2.71–2.57 (m, 4H) ppm; **^{13}C NMR** (151 MHz, $CDCl_3$) δ = 141.8, 141.5, 140.8, 139.8, 139.8, 139.6, 137.7, 137.5, 133.5, 132.8, 131.4, 130.7, 129.5, 128.5, 128.3, 127.9, 127.6, 126.9, 126.6, 126.5, 126.1, 125.6, 74.5, 73.8, 36.4, 34.5 ppm; **IR** (ATR) $\tilde{\nu}$ = 697 (s), 757 (m), 909 (m), 1055 (m), 1364 (w), 1442 (w), 1495 (m), 1603 (w) cm^{-1}; **HRMS** (APCI): calcd. for $C_{34}H_{28}O^+$: 452.2135; found: 452.2112; ***R*$_f$** = 0.24 (isohexane/ethyl acetate – 30/1).

4.1.34 Preparation of 4,5,6-triphenylisoindoline **39**

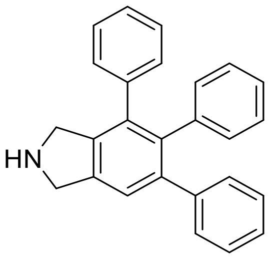

39

According to GP1, FeH(CO)(NO)(PPh_3)$_2$ **1** (12.8 mg, 20.0 µmol, 4 mol%) diphenylacetylene **3** (891 mg, 5.00 mmol, 10 eq.) was dissolved in anhydrous 1,4-dioxane (0.5 mL). Diyne **S31** (84.6 mg, 500 µmol, 1.0 eq.) in anhydrous 1,4-dioxane (0.5 mL) was slowly added at 60 °C. The reaction mixture was stirred for 5 minutes at 60 °C. After solvent evaporation and purification by silica gel column chromatography (DCM/methanol –1/1), the product **39** was obtained as a colourless foam in a yield of 108 mg (310 µmol, 62%).

^{1}H NMR (300 MHz, $CDCl_3$) δ = 7.35 (s, 1H), 7.23–7.06 (m, 8H), 7.04–6.99 (m, 2H), 6.96–6.89 (m, 3H), 6.85–6.77 (m, 2H), 4.42 (s, 2H), 4.14 (s, 2H), 2.92 (s, 1H) ppm; **^{13}C NMR** (75 MHz, $CDCl_3$) δ = 142.3, 141.3, 140.7, 140.2, 139.8, 139.6, 138.6, 136.9, 131.8, 130.1, 129.7, 127.8, 127.7, 127.1, 126.5, 126.3, 125.8, 123.5, 53.5, 53.2 ppm; **IR** (ATR) $\tilde{\nu}$ = 727 (s) ,760 (m), 906 (s), 1029 (m), 1073 (m) ,1249 (w), 1401 (m), 1431 (m), 1495 (m), 1543 (w), 1599 (m), 2848 (w), 3027 (w), 3056 (w) cm^{-1}; **HRMS** (ESI): calcd. for $C_{26}H_{22}N^+$: 348.1747; found: 348.1779; ***R*$_f$** = 0.31 (DCM/methanol –1/1).

4.1.35 Preparation of 4-ferrocene-5,6-diphenyl-2-tosylisoindoline **40**

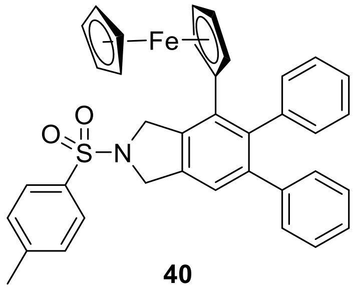

40

According to GP1, FeH(CO)(NO)(PPh_3)$_2$ **1** (12.8 mg, 20.0 µmol, 4 mol%) and diphenylacetylene **3** (891 g, 5.00 mmol, 10 eq.) was dissolved in anhydrous 1,4-dioxane (0.5 mL). Diyne **S26** (281 mg, 500 µmol, 1.0 eq.) in anhydrous 1,4-dioxane (0.5 mL) was slowly added at 60 °C.

The reaction mixture was stirred for 5 minutes at 60 °C. After solvent evaporation and purification by silica gel column chromatography (isohexane/ethyl acetate – 10/1), the product **40** was obtained as a red solid in a yield of 281 mg (462 µmol, 92%).

^{1}H NMR (300 MHz, $CDCl_3$) δ = 7.95–7.85 (m, 2H), 7.45–7.31 (m, 2H), 7.14–6.97 (m, 7H), 6.93–6.86 (m, 2H), 6.78–6.65 (m, 2H), 5.17 (s, 2H), 4.73 (s, 2H), 4.12–4.03 (m, 7H), 3.83 (s, 2H), 2.42 (s, 3H) ppm; **^{13}C NMR** (75 MHz, $CDCl_3$) δ = 144.0, 142.6, 141.9, 140.7, 139.9, 136.0, 134.2, 133.6, 133.0, 131.7, 130.1, 129.8, 128.0, 127.5, 126.3, 126.3, 122.4, 83.9, 70.4, 69.4, 67.6, 54.9, 53.5, 21.7 ppm; **IR** (ATR) $\tilde{\nu}$ = 667(s), 727 (s), 816 (m), 909 (m), 1059 (m), 1096 (m), 1163 (s), 1346 (m), 1405 (w), 1442 (w), 1599 (w), 2251 (w), 2848 (w), 2922 (w), 2952 (w), 3083 (w) cm^{-1}; **HRMS** (ESI): calcd. for $C_{37}H_{31}FeNO_2SNa^+$: 632.1317; found: 632.1317; ***R*f** = 0.34 (isohexane/ethyl acetate – 5/1).

4.1.36 Preparation of 5-phenethyl-4,7-diphenyl-2-tosylisoindoline **41**

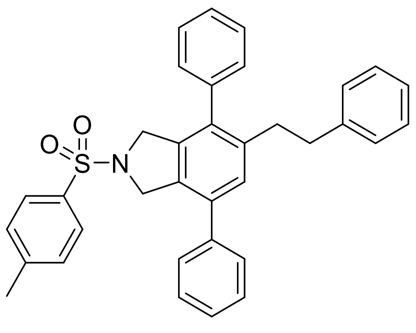

41

According to GP1, $FeH(CO)(NO)(PPh_3)_2$ **1** (12.8 mg, 20.0 µmol, 4 mol%) and 3-butyn-1-ylbenzene **S17** (0.70 mL, 5.0 mmol, 10 eq.) was dissolved in anhydrous 1,4-dioxane (0.5 mL). Diyne **S58** (200 mg, 500 µmol, 1.0 eq.) in anhydrous 1,4-dioxane (0.5 mL) was slowly added at 60 °C. The reaction mixture was stirred for 60 minutes at 60 °C. After solvent evaporation and purification by silica gel column chromatography (isohexane/ethyl acetate – 10/1), the product **41** was obtained as a colourless solid in a yield of 235 mg (444 µmol, 89%).

^{1}H NMR (300 MHz, $CDCl_3$) δ = 7.75–7.62 (m, 2H), 7.55–7.28 (m, 10H), 7.25–7.09 (m, 6H), 6.98–6.84 (m, 2H), 4.72 (s, 2H), 4.37 (s, 2H), 2.84 – 2.61 (m, 4H), 2.42 (s, 3H) ppm; **^{13}C NMR** (75 MHz, $CDCl_3$) δ = 143.7, 141.5, 140.1, 139.7, 138.3, 136.4, 136.2, 133.9, 131.7, 129.9, 129.7, 128.9, 128.7, 128.4, 128.4, 128.1, 127.8, 127.8, 127.7, 126.0, 54.0, 53.8, 38.0, 35.3, 21.6 ppm (due to coincidental chemical equivalence of carbon resonances two signal is missing); **IR** (ATR) $\tilde{\nu}$ = 663 (s), 701 (s), 727 (s), 813 (m), 906 (s), 1070 (m), 1096 (m), 1163 (m), 1342 (m), 1398 (w), 1465 (w), 1495 (w), 1599 (w), 2855 (w), 2922 (w), 3027 (w), 3056 (w) cm-

[1]; **HRMS** (ESI): calcd. for $C_{35}H_{31}NO_2Na^+$: 552.1968; found: 552.1970; ***R*f** = 0.30 (isohexane/ethyl acetate –10/1).

4.1.37 Preparation of 4,5,6,7-tetraphenyl-2-tosylisoindoline **42**

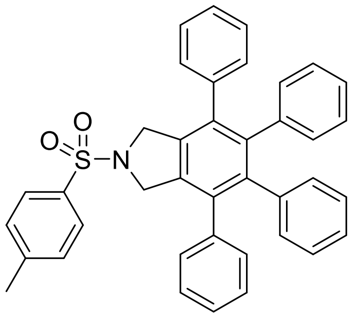

42

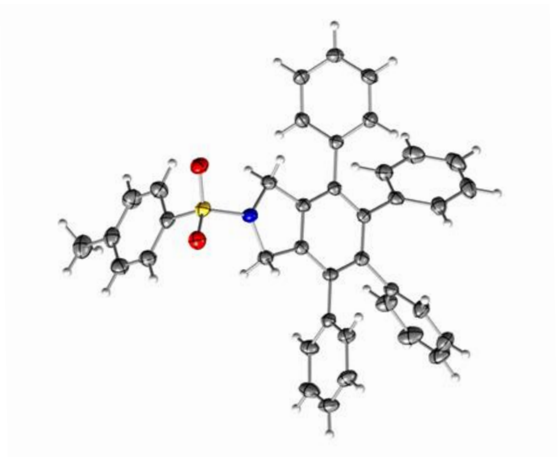

According to GP1, $FeH(CO)(NO)(PPh_3)_2$ **1** (12.8 mg, 20.0 µmol, 4 mol%) and diphenylacetylene **3** (891 g, 5.00 mmol, 10 eq.) was dissolved in anhydrous 1,4-dioxane (0.5 mL). Diyne **S58** (200 mg, 500 µmol, 1.0 eq.) in anhydrous 1,4-dioxane (0.5 mL) was slowly added at 60 °C. The reaction mixture was stirred for 30 minutes at 60 °C. After solvent evaporation and purification by silica gel column chromatography (isohexane/ethyl acetate – 10/1), the product **42** was obtained as a pale red foam in a yield of 261 mg (452 µmol, 90%).

^{1}H NMR (400 MHz, $CDCl_3$) δ = 7.73–7.65 (m, 2H), 7.36–7.28 (m, 2H), 7.22–7.12 (m, 6H), 7.05–6.98 (m, 4H), 6.90 – 6.80 (m, 6H), 6.76 – 6.68 (m, 4H), 4.54 (s, 4H), 2.43 (s, 3H) ppm; **^{13}C NMR** (101 MHz, $CDCl_3$) δ = 143.7, 141.1, 139.3, 138.8, 136.2, 134.6, 133.9, 131.4, 130.0, 129.4, 128.1, 127.8, 127.0, 126.9, 125.8, 54.4, 21.7 ppm; **IR** (ATR) $\tilde{\nu}$ = 671 (s), 701 (s), 731, (s), 813 (w), 909 (m), 1029 (w), 1070 (m), 1096 (m), 1163 (s), 1349 (m), 1442 (w), 1495 (w), 1599 (w), 2844 (w), 2922 (w), 2952 (w), 3027 (w), 3056 (w) cm^{-1}; **HRMS** (ESI): calcd. for $C_{39}H_{31}NO_2Na^+$: 600.1968; found: 600.1960; ***R*f** = 0.20 (isohexane/ethyl acetate – 10/1).

The X-Ray structure of **42** is deposited at the Cambridge Crystallographic Data Centre (www.ccdc.cam.ac.uk) under CCDC 242 4454.

4.1.38 Preparation of 4,5,7-triphenyl-6-(phenylethynyl)-2-tosylisoindoline **43**

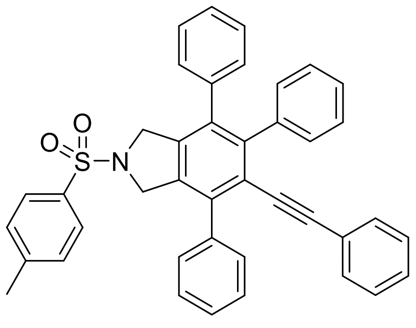

43

According to GP1, FeH(CO)(NO)$(PPh_3)_2$ **1** (12.8 mg, 20.0 µmol, 4 mol%) and 1,4-diphenyl-1,3-butadiyne (1.26 g, 5.00 mmol, 10 eq.) was dissolved in anhydrous 1,4-dioxane (1.0 mL). Diyne **S58** (200 mg, 500 µmol, 1.0 eq.) in anhydrous 1,4-dioxane (0.5 mL) was slowly added at 60 °C. The reaction mixture was stirred for 60 minutes at 60 °C. After solvent evaporation and purification by silica gel column chromatography (isohexane/ethyl acetate –10/1 to 5/1), the product **43** was obtained as a colourless solid in a yield of 210 mg (348 µmol, 70%).

^{1}H NMR (300 MHz, $CDCl_3$) δ = 7.76–7.64 (m, 2H), 7.54–7.39 (m, 5H), 7.34–7.28 (m, 2H), 7.24–7.05 (m, 11H), 7.04–6.97 (m, 2H), 6.76–6.67 (m, 2H), 4.58 (s, 2H), 4.51 (s, 2H), 2.43 (s, 3H) ppm; **^{13}C NMR** (75 MHz, $CDCl_3$) δ = 144.1, 143.8, 139.3, 138.8, 138.4, 138.1, 136.0, 135.8, 134.5, 133.8, 131.2, 130.9, 130.0, 129.4, 129.3, 128.4, 128.3, 128.1, 128.1, 127.7, 127.4, 127.3, 126.9, 123.3, 123.0, 97.0, 88.5, 54.4, 54.1, 21.7 ppm (due to coincidental chemical equivalence of two carbon resonances one signal is missing); **IR** (ATR) $\tilde{\nu}$ = 701 (s), 757 (m), 813 (m), 909 (m), 1070 (m), 1096 (m), 1159 (s), 1256 (w), 1346 (m), 1401 (w), 1442 (m), 1491 (m), 1595 (m), 2851 (w), 2922 (w), 3056 (w) cm^{-1}; **HRMS** (ESI): calcd. for $C_{41}H_{39}NO_2Na^+$: 624.1968; found: 624.1970; ***R*$_f$** = 0.21 (isohexane/ethyl acetate – 10/1).

4.1.39 Preparation of 5,6-diphenyl-2-tosyl-4-vinylisoindoline **44**

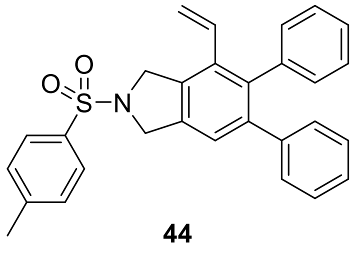

44

According to GP1, FeH(CO)(NO)$(PPh_3)_2$ **1** (12.8 mg, 20.0 µmol, 4 mol%) and diphenylacetylene **3** (891 mg, 5.00 mmol, 10 eq.) was dissolved in anhydrous 1,4-dioxane (0.5 mL). Diyne **S37** (137 mg, 500 µmol, 1.0 eq.) in anhydrous 1,4-dioxane (0.5 mL) was slowly added at 60 °C.

The reaction mixture was stirred for 5 minutes at 60 °C. After solvent evaporation and purification by silica gel column chromatography isohexane/ethyl acetate – 8/1), the product **44** was obtained as a colourless solid in a yield of 190 mg (420 µmol, 84%).

^{1}H NMR (400 MHz, $CDCl_3$) δ = 7.86–7.80 (m, 2H), 7.39–7.33 (m, 2H), 7.19–7.14 (m, 4H), 7.14–7.09 (m, 3H), 7.00–6.93 (m, 4H), 6.34 (dd, J = 18.0, 11.7 Hz, 1H), 5.35–5.18 (m, 2H), 4.81 (s, 2H), 4.72 (s, 2H), 2.44 (s, 3H) ppm; **^{13}C NMR** (101 MHz, $CDCl_3$) δ = 143.9, 142.1, 141.3, 139.8, 138.9, 136.1, 135.1, 133.7, 133.5, 132.8, 131.2, 130.0, 129.7, 127.8, 127.7, 127.7, 126.8, 126.5, 123.2, 118.7, 54.5, 53.7, 21.7 ppm; **IR** (ATR) $\tilde{\nu}$ = 772 (m), 835 (w), 909 (m), 991 (w), 1029 (m), 1066 (m), 1096 (s),1163 (s), 1346 (m), 1398 (w), 1446 (w), 1495 (w), 1595 (w), 2848 (w), 2922 (w), 3027 (w), 3056 (w) cm^{-1}; **HRMS** (ESI): calcd. for $C_{29}H_{26}NO_2S^+$: 452.1679; found: 452.1689; $\boldsymbol{R_f}$ = 0.49 (isohexane/ethyl acetate – 5/1).

4.1.40 Preparation of 5,6,7-triphenyl-2-tosylisoindolin-1-one **45**

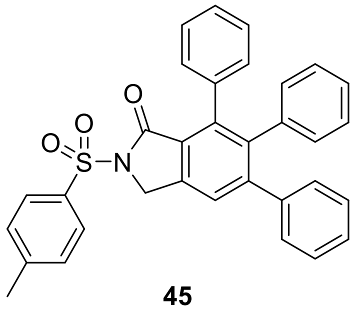

45

According to GP1, $FeH(CO)(NO)(PPh_3)_2$ **1** (32.1 mg, 50.2 µmol, 10 mol%) and diphenylacetylene **3** (891 mg, 5.00 mmol, 10 eq.) was dissolved in anhydrous 1,4-dioxane (0.5 mL). Diyne **S62** (169 mg, 500 µmol, 1.0 eq.) in anhydrous 1,4-dioxane (0.5 mL) was slowly added at 60 °C. The reaction mixture was stirred for 60 minutes at 60 °C. After solvent evaporation and purification by silica gel column chromatography (isohexane/ethyl acetate – 5/1 to 2:1), the product **45** was obtained as a pale yellow solid in a yield of 212 mg (411 µmol, 82%).

^{1}H NMR (300 MHz, $CDCl_3$) δ = 8.01–7.91 (m, 2H), 7.51 (s, 1H), 7.32–7.26 (m, 2H), 7.20–7.09 (m, 6H), 7.06–7.00 (m, 2H), 6.99–6.94 (m, 2H), 6.93–6.86 (m, 3H), 6.71–6.64 (m, 2H), 4.96 (s, 2H), 2.41 (s, 3H) ppm. **^{13}C NMR** (101 MHz, $CDCl_3$) δ = 165.0, 147.6, 145.0, 141.9, 141.7, 140.9, 140.8, 137.9, 135.6, 135.4, 131.5, 130.3, 129.8, 129.6, 128.2, 127.9, 127.2, 127.2, 127.2, 126.3, 126.2, 124.2, 48.6, 21.8 ppm (due to coincidental chemical equivalence of two carbon resonances one signal is missing); **IR** (ATR) $\tilde{\nu}$ = 671 (s), 701 (s), 731 (s), 764 (m), 813 (m), 909 (m), 1088 (s), 1170 (s), 1290 (w), 1349 (m), 1413 (w), 1446 (m) 1495 (w), 1599 (m), 1729

(m), 2926 (w), 3030 (w), 3056 (w) cm^{-1}; **HRMS** (ESI): calcd. for $C_{33}H_{25}NO_3SNa^+$: 538.1447; found: 538.1450; $\boldsymbol{R_f}$ = 0.29 (isohexane/ethyl acetate – 5/1).

4.1.41 Preparation of 1,1-dimethyl-5,6,7-triphenyl-2-tosylisoindoline **46**

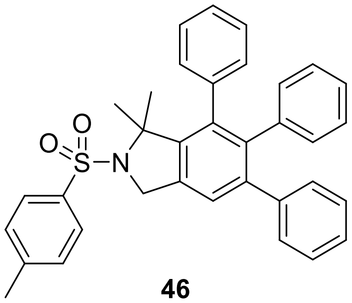

46

According to GP1, $FeH(CO)(NO)(PPh_3)_2$ **1** (5.1 mg, 8.0 µmol, 4 mol%) diphenylacetylene **3** (357 mg, 2.00 mmol, 10 eq.) was dissolved in anhydrous 1,4-dioxane (0.2 mL). Diyne **S35** (70.3 mg, 200 µmol, 1.0 eq.) in anhydrous 1,4-dioxane (0.2 mL) was slowly added at 60 °C. The reaction mixture was stirred for 5 minutes at 60 °C. After solvent evaporation and purification by silica gel column chromatography (isohexane/ethyl acetate – 10/1), the product **43** was obtained as a colourless oil in a yield of 91.3 mg (172 µmol, 86%).

^{1}H NMR (300 MHz, $CDCl_3$) δ = 7.87–7.76 (m, 2H), 7.32–7.23 (m, 3H), 7.15–6.96 (m, 10H), 6.89–6.80 (m, 3H), 6.76–6.66 (m, 2H), 4.74 (s, 2H), 2.42 (s, 3H), 1.61 (s, 6H) ppm; **^{13}C NMR** (75 MHz, $CDCl_3$) δ = 143.1, 142.6, 141.5, 139.3, 138.6, 137.7, 137.6, 133.6, 131.5, 131.4, 129.8, 129.5, 127.7, 127.5, 127.0, 126.9, 126.9, 126.5, 125.7, 123.3, 73.3, 52.4, 28.6, 21.6 ppm; **IR** (ATR) $\tilde{\nu}$ = 813 (m), 906 (s), 1036 (m), 1092 (m), 1155 (m), 1204 (w), 1245 (w), 1334 (m), 1409 (w), 1439 (w), 1495 (w), 1599 (w), 1670 (w), 1715 (w), 2863 (w), 2937 (w), 2989 (w), 3027 (w), 3056 (w) cm^{-1}; **HRMS** (ESI): calcd. for $C_{35}H_{31}NO_2SNa^+$: 552.1968; found: 552.2024; $\boldsymbol{R_f}$ = 0.60 (isohexane/ethyl acetate –5/1).

4.1.42 Preparation of 4,5,6,7-tetraphenylisobenzofuran-1(3*H*)-one **47**

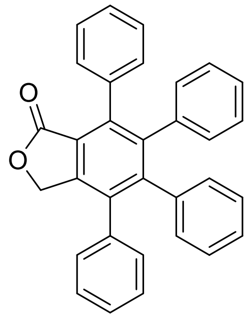

47

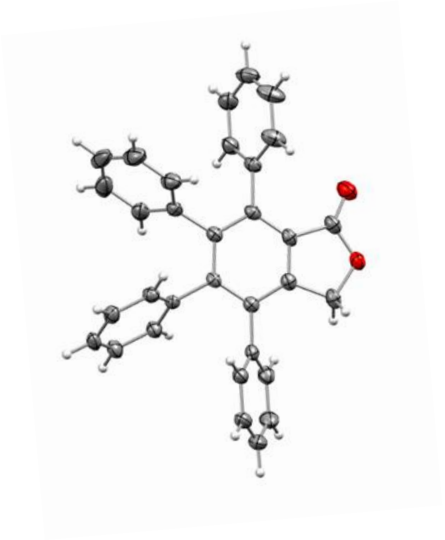

According to GP1, $FeH(CO)(NO)(PPh_3)_2$ **1** (32.1 mg, 50.2 µmol, 10 mol%) and diphenylacetylene **3** (891 mg, 5.00 mmol, 10 eq.) was dissolved in anhydrous 1,4-dioxane (0.5 mL). Diyne **S60** (130 mg, 500 µmol, 1.0 eq.) in anhydrous 1,4-dioxane (0.5 mL) was slowly added at 60 °C. The reaction mixture was stirred for 60 minutes at 60 °C. After solvent evaporation and purification by silica gel column chromatography (isohexane/ethyl acetate – 10/1), the product **47** was obtained as a colourless solid in a yield of 146 mg (333 µmol, 67%).

^{1}H NMR (400 MHz, $CDCl_3$) δ = 7.25–7.17 (m, 6H), 7.16–7.08 (m, 4H), 6.96–6.87 (m, 6H), 6.83–6.73 (m, 4H), 5.16 (s, 2H) ppm; **^{13}C NMR** (101 MHz, $CDCl_3$) δ = 170.0, 146.6, 145.8, 143.4, 140.8, 138.6, 138.5, 137.0, 135.7, 135.5, 131.4, 130.9, 130.3, 129.3, 128.5, 127.6, 127.3, 127.3, 127.2, 127.1, 126.4, 126.1, 122.1, 68.3 ppm; **IR** (ATR) $\tilde{\nu}$ = 697 (s), 723 (s), 783 (m), 813 (m), 909 (s), 1029 (s), 1111 (m), 1185 (m), 1256 (w), 1323 (m), 1353 (m), 1387 (w), 1442 (m), 1495 (m), 1565 (m), 1759 (s), 2251 (w), 2878 (w), 2933 (w), 3027 (w), 3056 (m) cm^{-1}; **HRMS** (ESI): calcd. for $C_{32}H_{22}O_2Na^+$: 461.1512; found: 461.1541; $\boldsymbol{R_f}$ = 0.09 (isohexane/ethyl acetate – 15/1).

The X-Ray structure of **47** is deposited at the Cambridge Crystallographic Data Centre (www.ccdc.cam.ac.uk) under CCDC 242 4453.

4.1.43 Preparation of 2,3,4-triphenyl-9-tosyl-9*H*-carbazole **48**

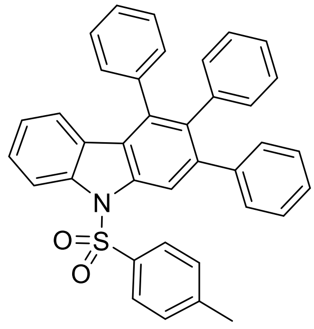

48

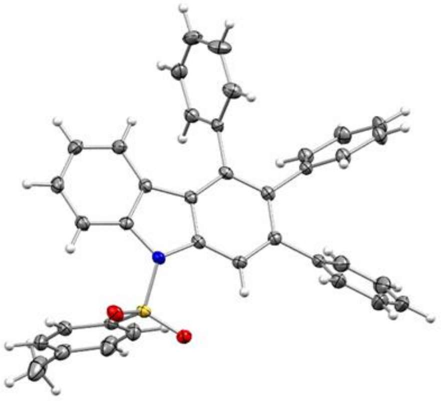

According to GP1, $FeH(CO)(NO)(PPh_3)_2$ **8** (12.8 mg, 20.0 µmol, 4 mol%) and diphenylacetylene **3** (891 g, 5.00 mmol, 10 eq.) was dissolved in anhydrous 1,4-dioxane (0.5 mL). Diyne **S52** (186 mg, 500 µmol, 1.0 eq.) in anhydrous 1,4-dioxane (0.5 mL) was slowly added at 60 °C. The reaction mixture was stirred for 5 minutes at 60 °C. After solvent evaporation and purification by silica gel column chromatography (isohexane/ethyl acetate – 10/1), the product **48** was obtained as a yellow solid in a yield of 246 mg (447 µmol, 89%).

^{1}H NMR (400 MHz, $CDCl_3$) δ = 8.46 (s, 1H), 8.39–8.31 (m, 1H), 7.87–7.78 (m, 2H), 7.38 (ddd, J = 8.5, 7.3, 1.3 Hz, 1H), 7.29–7.24 (m, 4H), 7.23–7.16 (m, 6H), 7.14–7.09 (m, 2H), 7.02–6.96 (m, 1H), 6.95–6.90 (m, 3H), 6.87–6.81 (m, 2H), 6.55–6.48 (m, 1H), 2.34 (s, 3H) ppm; **^{13}C NMR** (101 MHz, $CDCl_3$) δ = 145.1, 142.2, 141.2, 139.2, 139.1, 137.5, 136.7, 136.4, 135.5, 131.8, 130.4, 130.2, 130.0, 128.3, 127.7, 127.3, 127.1, 127.0, 126.9, 126.5, 126.4, 125.8, 123.8, 123.5, 122.5, 115.4, 114.7, 21.8 ppm (due to coincidental chemical equivalence of two carbon resonances one signal is missing); **IR** (ATR) $\tilde{\nu}$ = 663 (s) ,731 (s), 813 (m), 909 (m), 988 (m), 1029 (m), 1092 (m), 1122 (m), 1174 (s), 1230 (m), 1331 (m), 1368 (m), 1409 (m), 1442 (m), 1595 (m), 2851 (w), 2922 (w), 3056 (w) cm^{-1}; **HRMS** (ESI): calcd. for $C_{37}H_{27}NO_2SK^+$: 588.1394; found: 588.1394; ***R*$_f$** = 0.44 (isohexane/ethyl acetate – 10/1).

The X-Ray structure of **48** is deposited at the Cambridge Crystallographic Data Centre (www.ccdc.cam.ac.uk) under CCDC 242 4457.

4.1.44 Preparation of 1,2,3-triphenyldibenzo[*b*,*d*]furan **49**

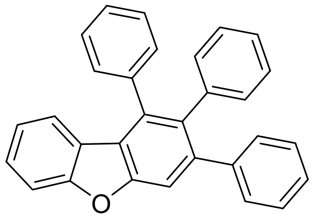

49

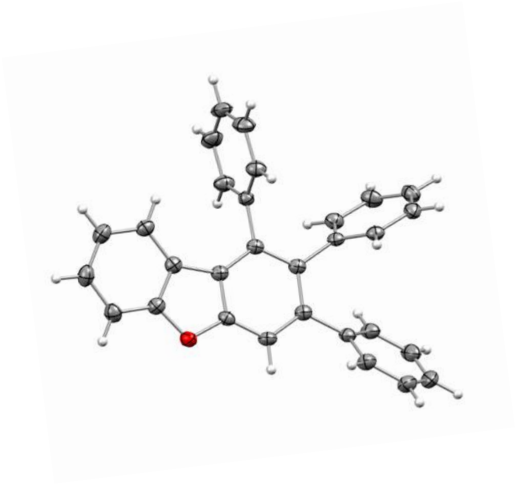

According to GP1, $FeH(CO)(NO)(PPh_3)_2$ **1** (2.6 mg, 4.0 µmol, 4 mol%) and diphenylacetylene **3** (178 mg, 1.00 mmol, 10 eq.) was dissolved in anhydrous 1,4-dioxane (0.1 mL). Diyne **S57** (21.8 mg, 99.9 µmol, 1.0 eq.) in anhydrous 1,4-dioxane (0.1 mL) was slowly added at 60 °C. The reaction mixture was stirred for 5 minutes at 60 °C. After solvent evaporation and purification by silica gel column chromatography (isohexane/ethyl acetate – 1/0), the product **55** was obtained as a colourless solid in a yield of 28.4 mg (71.6 µmol, 72%).

^{1}H NMR (300 MHz, $CDCl_3$) δ = 7.66 (s, 1H), 7.57 (d, J = 8.2 Hz, 1H), 7.42–7.35 (m, 1H), 7.33–7.20 (m, 5H), 7.18 (s, 5H), 7.08–7.00 (m, 1H), 6.99–6.93 (m, 3H), 6.92–6.82 (m, 3H) ppm; **^{13}C NMR** (151 MHz, $CDCl_3$) δ = 157.0, 155.2, 142.1, 141.5, 139.3, 139.0, 136.8, 135.2, 132.1, 130.2, 130.2, 128.2, 127.7, 127.2, 127.1, 127.0, 126.5, 125.8, 124.4, 122.6, 122.4, 122.3, 112.2, 111.5 ppm; **IR** (ATR) $\tilde{\nu}$ = 693 (s), 738 (s) ,798 (m), 872 (m), 913 (m), 1014 (m), 1070 (m), 1103 (m), 1141 (m), 1193 (m), 1260 (m), 1334 (m), 1409 (m), 1442 (m), 1498 (m), 1592 (m) cm^{-1}; **HRMS** (APCI): calcd for $C_{30}H_{20}O^+$: 396.1509; found: 396.1505; $\boldsymbol{R_f}$ = 0.20 (isohexane/ethyl acetate – 20/1).

The X-Ray structure of **49** is deposited at the Cambridge Crystallographic Data Centre (www.ccdc.cam.ac.uk) under CCDC 242 4450.

4.1.45 Preparation of 7,8,9,10-Tetraphenylfluoranthene **50**

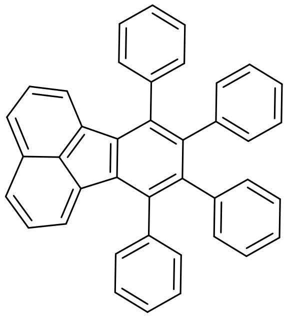

50

According to GP1, FeH(CO)(NO)(PPh_3)$_2$ **1** (12.8 mg, 20.0 µmol, 4 mol%) and diphenylacetylene **3** (891 g, 5.00 mmol, 10 eq.) was dissolved in anhydrous 1,4-dioxane (0.5 mL). Diyne **S54** (164 mg, 500 µmol, 1.0 eq.) in anhydrous 1,4-dioxane (1.0 mL) was slowly added at 60 °C. The reaction mixture was stirred for 40 minutes at 60 °C. After solvent evaporation and purification by silica gel column chromatography (isohexane/ethyl acetate – 1/0 to 50/1), the product **50** was obtained as a colourless solid in a yield of 230 mg (454 µmol, 91%).

^{1}H NMR (400 MHz, $CDCl_3$) δ = 7.71 (d, J = 7.9 Hz, 2H), 7.36–7.27 (m, 12H), 6.96–6.80 (m, 10H), 6.61 (d, J = 7.0 Hz, 2H) ppm; **^{13}C NMR** (101 MHz, $CDCl_3$) δ = 140.8, 140.0, 140.0, 137.3, 136.6, 136.6, 133.4, 131.4, 130.2, 129.7, 128.3, 127.8, 127.0, 126.8, 126.7, 125.6, 123.3 ppm; **IR** (ATR) $\tilde{\nu}$ = 701 (s), 775 (m), 1029 (w), 1070 (w), 1163 (w), 1375 (w), 1428 (m), 1435 (w), 1461 (w), 1498 (w), 1603 (w), 1737 (w), 2851 (w), 2922 (m), 3027 (w), 3056 (w) cm^{-1}; **HRMS** (ESI): calcd. for $C_{40}H_{26}K^+$: 545.1666; found: 545.1666; $\boldsymbol{R_f}$ = 0.11 (isohexane/ethyl acetate – 1/0).

4.1.46 Preparation of 4-Methyl-5,6,7-triphenylisobenzofuran-1(3*H*)-one **51**

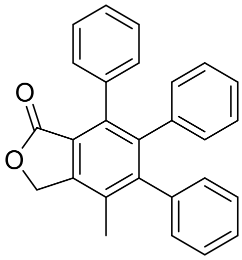

51

According to GP1, FeH(CO)(NO)(PPh_3)$_2$ **1** (32.1 mg, 50.2 µmol, 10 mol%) and diphenylacetylene **3** (891 mg, 5.00 mmol, 10 eq.) was dissolved in anhydrous 1,4-dioxane (0.5 mL). Diyne **S63** (130 mg, 500 µmol, 1.0 eq.) in anhydrous 1,4-dioxane (0.5 mL) was slowly added at 60 °C.

The reaction mixture was stirred for 60 minutes at 60 °C. After solvent evaporation and purification by silica gel column chromatography (isohexane/ethyl acetate – 6/1), the product **51** was obtained as a colourless solid in a yield of 172 mg (456 µmol, 91 %).

^{1}H NMR (300 MHz, $CDCl_3$) δ = 7.23–7.11 (m, 6H), 7.09–7.02 (m, 2H), 7.00–6.94 (m, 2H), 6.90–6.82 (m, 3H), 6.76–6.66 (m, 2H), 5.31 (s, 2H), 2.13 (s, 3H) ppm; **^{13}C NMR** (75 MHz, $CDCl_3$) δ = 170.2, 147.6, 145.8, 143.0, 139.2, 139.2, 138.6, 135.6, 131.2, 130.4, 129.8, 129.8, 127.9, 127.3, 127.2, 127.0, 127.0, 126.0, 121.7, 68.0, 16.3 ppm; **IR** (ATR) $\tilde{\nu}$ = 701 (s), 731 (s), 760 (m), 801 (w), 909 (m), 954 (w), 1029 (m), 1066 (m), 1111 (w), 1163 (m), 1196 (m), 1267 (w), 1305 (w), 1353 (m), 1442 (m), 1495 (w), 1588 (w), 1759 (s), 2926 (w), 3027 (w), 3056 (w) cm^{-1}; **HRMS** (ESI): calcd. for $C_{27}H_{20}O_2Na^+$: 399.1356; found: 399.1356; ***R*f** = 0.21 (*i*Hexan/Essigsäureethylester –6/1).

The analytical data are in good accordance with the literature.[8]

4.1.47 Preparation of 5-((((3*S*,9*S*,10*R*,13*R*,14*S*,17*R*)-10,13-Dimethyl-17-((*R*)-6-methylheptan-2-yl)-2,3,4,7,8,9,10,11,12,13,14,15,16,17-tetradecahydro-1*H*-cyclopenta[*a*]phenanthren-3-yl)oxy)methyl)-2-tosylisoindolinete **52**

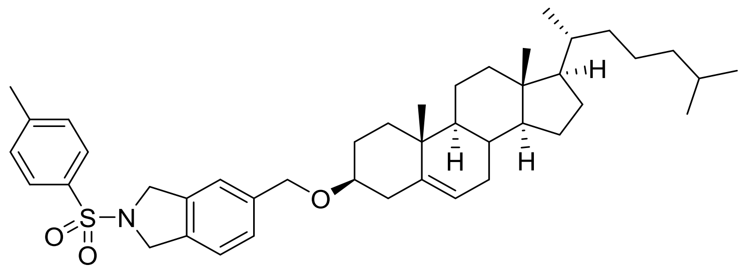

52

According to GP1, $FeH(CO)(NO)(PPh_3)_2$ **1** (5.1 mg, 8.0 µmol, 4 mol%) and alkyne **S68** (495 mg, 2.00 mmol, 4.0 eq.) was dissolved in anhydrous 1,4-dioxane (1.0 mL). Diyne **2** (49.5 mg, 200 µmol, 1.0 eq.) in anhydrous 1,4-dioxane (0.2 mL) was slowly added at 60 °C. The reaction mixture was stirred for 5 minutes at 60 °C. After solvent evaporation and purification by silica gel column chromatography (isohexane/ethyl acetate – 8/1), the product **53** was obtained as a colourless solid in a yield of 92.0 mg (137 µmol, 68%).

^{1}H NMR (300 MHz, $CDCl_3$) δ = 7.80–7.72 (m, 2H), 7.34–7.27 (m, 2H), 7.22–7.09 (m, 3H), 5.40–5.30 (m, 1H), 4.60 (s, 4H), 4.49 (s, 2H), 3.31–3.15 (m, 1H), 2.46–2.32 (m, 4H), 2.30–2.18 (m, 1H), 2.07–1.73 (m, 5H), 1.65–0.77 (m, 33H), 0.67 (s, 3H) ppm; **^{13}C NMR** (75 MHz, $CDCl_3$) δ = 143.8, 141.0, 139.2, 136.5, 135.4, 133.9, 129.9, 127.7, 127.3, 122.6, 121.9, 121.8, 80.0, 69.7, 56.9, 56.3, 53.8, 53.7, 50.3, 42.5, 39.9, 39.7, 39.2, 37.3, 37.0, 36.3, 35.9, 32.0, 32.0, 28.5, 28.4, 28.1, 24.4, 24.0, 22.9, 22.7, 21.6, 21.2, 19.5, 18.8, 12.0 ppm; **IR** (ATR) $\tilde{\nu}$ = 667 (s), 731

(m), 813 (m), 909 (m), 1096 (s), 1163 (s), 1346 (m), 1465 (m), 2866 (m), 2930 (m) cm^{-1}; **HRMS** (ESI): calcd. for $C_{43}H_{61}NO_3SNa^+$: 694.4264; found: 694.4270; ***R*f** = 0.26 (isohexane/ethyl acetate – 8/1).

4.1.48 Preparation of (*S*)-6-Chloro-4-(6-cyclopropyl-2-tosylisoindolin-5-yl)-1-methyl-4-(trifluoromethyl)-1,4-dihydro-2*H*-benzo[*d*][1,3]oxazin-2-one **53**

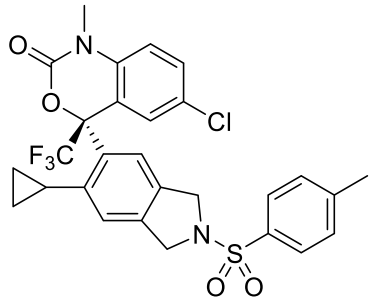

53

According to GP1, $FeH(CO)(NO)(PPh_3)_2$ **1** (12.8 mg, 20.0 µmol, 4 mol%) and alkyne **S70** (659 mg, 2.00 mmol, 10 eq.) was dissolved in anhydrous 1,4-dioxane (0.4 mL). Diyne **2** (49.5 mg, 200 µmol, 1.0 eq.) in anhydrous 1,4-dioxane (0.2 mL) was slowly added at 60 °C. The reaction mixture was stirred for 20 minutes at 60 °C. After solvent evaporation and purification by silica gel column chromatography (isohexane/ethyl acetate – 6/1 to 1/1), the product **53** was obtained as a colourless solid in a yield of 87.7 mg (152 µmol, 76 %).

^{1}H NMR (400 MHz, $CDCl_3$) δ = 7.84–7.70 (m, 2H), 7.42–7.28 (m, 4H), 6.99–6.86 (m, 2H), 6.75 (d, *J* = 1.7 Hz, 1H), 4.75–4.49 (m, 4H), 3.45 (s, 3H), 2.42 (s, 3H), 1.69–1.58 (m, 1H), 0.82–0.57 (m, 2H), 0.38–0.21 (m, 1H), 0.03–−0.12 (m, 1H) ppm; **^{13}C NMR** (101 MHz, $CDCl_3$) δ = 148.6, 144.0, 142.9, 138.6, 136.8, 133.7, 133.6, 133.5, 131.2, 130.1, 128.8, 127.9, 127.7, 123.3, 121.3, 121.3, 119.4, 114.7, 53.8, 53.7, 32.0, 21.7, 14.5, 8.2, 8.1 ppm (due to coincidental chemical equivalence of two carbon resonances one signal is missing); **IR** (ATR) $\tilde{\nu}$ = 667 (m), 731 (m), 816 (m), 880 (w), 909 (m), 1051 (m), 1096 (m), 1159 (s), 1185 (m), 1267 (m), 1342 (m), 1424 (w), 1498 (m), 1599 (w), 1729 (s) cm^{-1}; **HRMS** (ESI): calcd. for $C_{28}H_{24}ClF_3N_2O_4SNa^+$: 694.4264; found: 694.4270; ***R*f** = 0.23 (isohexane/ethyl acetate – 2/1).

4.1.49 Preparation of (2*S*)-2-((*R*)-(6-Methoxyquinolin-4-yl)((2-tosylisoindolin-5-yl)methoxy)methyl)-5-vinylquinuclidine **54**

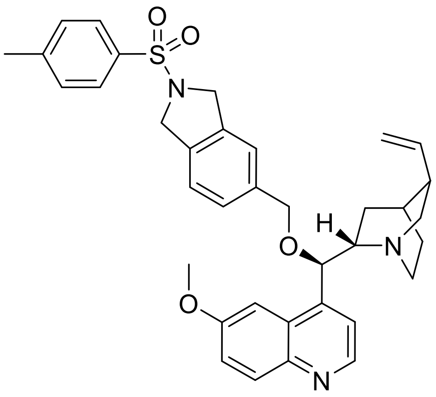

54

Diyne **2** (24.7 mg, 100 µmol, 1.0 eq.) and alkyne **S72** (363 mg, 1.00 mmol, 10 eq.) was dissolved in anhydrous 1,4-dioxane (0.2 mL). $FeH(CO)(NO)(PPh_3)_2$ **1** (6.4 mg, 10 µmol, 10 mol%) was added in one portion at 60 °C. The reaction mixture was stirred for 20 minutes at 60 °C. After solvent evaporation and purification by silica gel column chromatography (isohexane/ethyl acetate – 1/2 + 5% Et_3N → 1/6 + 5% Et_3N), the product **54** was obtained as a yellow oil in a yield of 50.6 mg (83.0 µmol, 83%).

^{1}H NMR (400 MHz, $CDCl_3$) δ = 8.73 (d, J = 4.5 Hz, 1H), 8.03 (d, J = 9.2 Hz, 1H), 7.80–7.73 (m, 2H), 7.45–7.28 (m, 5H), 7.20–7.09 (m, 3H), 5.77–5.61 (m, 1H), 5.00–4.85 (m, 2H), 4.59 (s, 4H), 4.44–4.32 (m, 2H), 4.01–3.85 (m, 3H), 3.49–3.37 (m, 1H), 3.31–3.22 (m, 1H), 3.20–3.04 (m, 2H), 2.79–2.59 (m, 2H), 2.39 (s, 3H), 2.34–2.25 (m, 1H), 1.86–1.77 (m, 2H), 1.77–1.65 (m, 1H), 1.63–1.43 (m, 2H) ppm; **^{13}C NMR** (101 MHz, $CDCl_3$) δ = 170.1, 158.1, 147.6, 144.8, 144.2, 143.8, 141.4, 137.8, 136.6, 135.8, 133.7, 132.0, 129.9, 127.7, 127.4, 122.8, 122.0, 114.7, 101.2, 71.0, 60.1, 56.7, 55.9, 53.7, 53.6, 43.2, 39.7, 34.5, 29.8, 27.8, 27.5, 23.4, 21.6, 14.9 ppm; **IR** (ATR) $\tilde{\nu}$ = 663 (s), 727 (s), 813 (m), 909 (m), 1029 (m), 1062 (m), 1096 (m), 1163 (s), 1241 (m), 1346 (m), 1469 (m), 1506 (m), 1558 (w), 1592 (w), 1618 (m), 2866 (w), 2926 (w) cm^{-1}; **HRMS** (ESI): calcd. for $C_{36}H_{40}N_3O_4S^+$: 610.2734; found: 610.2758; ***R***R_f = 0.09 (isohexane/ethyl acetate – 1/2 + 5% Et_3N).

4.1.50 Preparation of (2-Tosylisoindolin-5-yl)methyl (*S*)-2-(6-methoxynaphthalen-2-yl)propanoate **55**

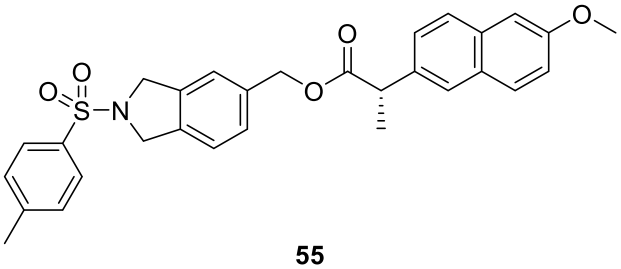

55

Diyne **2** (49.5 mg, 200 µmol, 1.0 eq.) and alkyne **S76** (238 mg, 600 µmol, 3.0 eq.) was dissolved in anhydrous 1,4-dioxane (0.4 mL). FeH(CO)(NO)$(PPh_3)_2$ **1** (12.8 mg, 20.0 µmol, 10 mol%) was added in one portion at 60 °C. The reaction mixture was stirred for 20 minutes at 60 °C. After solvent evaporation and purification by silica gel column chromatography (isohexane/ethyl acetate – 4/1), the product **56** was obtained as a colourless solid in a yield of 92.0 mg (137 µmol, 68 %).

^{1}H NMR (300 MHz, $CDCl_3$) δ = 7.80–7.72 (m, 2H), 7.34–7.27 (m, 2H), 7.22–7.09 (m, 3H), 5.40–5.30 (m, 1H), 4.60 (s, 4H), 4.49 (s, 2H), 3.31–3.15 (m, 1H), 2.46–2.32 (m, 4H), 2.30–2.18 (m, 1H), 2.07–1.73 (m, 5H), 1.65–0.77 (m, 33H), 0.67 (s, 3H) ppm; **^{13}C NMR** (75 MHz, $CDCl_3$) δ = 143.8, 141.0, 139.2, 136.5, 135.4, 133.9, 129.9, 127.7, 127.3, 122.6, 121.9, 121.8, 80.0, 69.7, 56.9, 56.3, 53.8, 53.7, 50.3, 42.5, 39.9, 39.7, 39.2, 37.3, 37.0, 36.3, 35.9, 32.0, 32.0, 28.5, 28.4, 28.1, 24.4, 24.0, 22.9, 22.7, 21.6, 21.2, 19.5, 18.8, 12.0 ppm; **IR** (ATR) $\tilde{\nu}$ = 667 (s), 731 (m), 813 (m), 909 (m), 1096 (s), 1163 (s), 1346 (m), 1465 (m), 2866 (m), 2930 (m) cm^{-1}; **HRMS** (ESI): calcd. for $C_{43}H_{61}NO_3SNa^+$: 694.4264; found: 694.4270; ***R*$_f$** = 0.26 (isohexane/ethyl acetate – 8/1).

4.1.51 Preparation of (2-Tosylisoindolin-5-yl)methyl 2-(1-(4-chlorobenzoyl)-5-methoxy-2-methyl-1*H*-indol-3-yl)acetate **56**

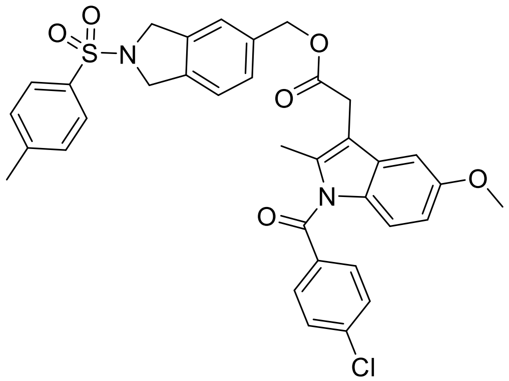

56

Diyne **2** (49.5 mg, 200 µmol, 1.0 eq.) and alkyne **S74** (238 mg, 600 µmol, 3.0 eq.) was dissolved in anhydrous 1,4-dioxane (0.4 mL). FeH(CO)(NO)$(PPh_3)_2$ **1** (12.8 mg, 20.0 µmol, 10 mol%) was added in one portion at 60 °C. The reaction mixture was stirred for 20 minutes at 60 °C. After solvent evaporation and purification by silica gel column chromatography (isohexane/ethyl acetate – 3/1), the product **56** was obtained as a yellow oil in a yield of 69.8 mg (109 µmol, 54 %).

^{1}H NMR (400 MHz, $CDCl_3$) δ = 7.80–7.73 (m, J = 8.3 Hz, 2H), 7.67–7.60 (m, 2H), 7.49–7.43 (m, 2H), 7.31 (d, J = 8.0 Hz, 2H), 7.18–7.08 (m, 2H), 7.05 (s, 1H), 6.91–6.82 (m, 2H), 6.65 (dd,

J = 9.0, 2.5 Hz, 1H), 5.08 (s, 2H), 4.58 (s, 2H), 4.54 (s, 2H), 3.73 (s, 3H), 3.68 (s, 2H), 2.40 (s, 3H), 2.35 (s, 3H) ppm; **^{13}C NMR** (101 MHz, $CDCl_3$) δ = 170.7, 168.4, 156.1, 143.9, 139.5, 136.8, 136.4, 136.1, 135.8, 133.9, 133.7, 131.3, 130.9, 130.6, 130.0, 129.3, 127.8, 127.7, 122.9, 122.5, 115.1, 112.4, 111.6, 101.6, 66.4, 55.7, 53.6, 30.5, 21.6, 13.5 ppm (due to coincidental chemical equivalence of two carbon resonances one signal is missing); **IR** (ATR) $\tilde{\nu}$ = 663 (s), 727 (s), 813 (m), 906 (m), 1088 (m), 1163 (s), 1223 (m), 1260 (m), 1316 (m), 1349 (m), 1401 (w), 1476 (m), 1595 (m), 1677 (m), 1733 (m), 2926 (w) cm^{-1}; **HRMS** (ESI): calcd. for $C_{35}H_{31}ClN_2O_6SNa^+$: 665.1484; found: 665.1498; ***R*f** = 0.14 (isohexane/ethyl acetate – 3/1).

4.1.52 Preparation of 5-((((3a'*R*,4*S*,7'*S*,7a'*R*)-2,2,2',2'-Tetramethyltetrahydrospiro[[1,3]dioxolane-4,6'-[1,3]dioxolo[4,5-c]pyran]-7'-yl)oxy)methyl)-2-tosylisoindoline **57**

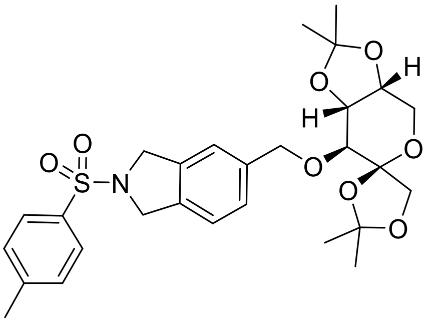

57

Diyne **2** (49.5 mg, 200 µmol, 1.0 eq.) and alkyne **S78** (179 mg, 600 µmol, 3.0 eq.) was dissolved in anhydrous 1,4-dioxane (0.4 mL). $FeH(CO)(NO)(PPh_3)_2$ **1** (12.8 mg, 20.0 µmol, 10 mol%) was added in one portion at 60 °C. The reaction mixture was stirred for 20 minutes at 60 °C. After solvent evaporation and purification by silica gel column chromatography (isohexane/ethyl acetate – 3/1), the product **57** was obtained as a yellow oil in a yield of 70.1 mg (128 µmol, 64 %).

^{1}H NMR (600 MHz, $CDCl_3$) δ = 7.79–7.74 (m, 2H), 7.31 (d, *J* = 8.1 Hz, 2H), 7.21 (d, *J* = 7.8 Hz, 1H), 7.17 (s, 1H), 7.12 (d, *J* = 7.8 Hz, 1H), 4.91 (d, *J* = 12.0 Hz, 1H), 4.66–4.51 (m, 5H), 4.36–4.32 (m, 1H), 4.21 (dd, *J* = 5.6, 2.3 Hz, 1H), 4.13 (dd, *J* = 13.4, 2.7 Hz, 1H), 4.03 (d, *J* = 8.5 Hz, 1H), 3.99 (d, *J* = 13.4 Hz, 1H), 3.87 (d, *J* = 8.5 Hz, 1H), 3.44 (d, *J* = 7.3 Hz, 1H), 2.40 (s, 3H), 1.50 (s, 3H), 1.48 (s, 3H), 1.38 (s, 3H), 1.36 (s, 3H) ppm; **^{13}C NMR** (151 MHz, $CDCl_3$) δ = 143.8, 138.4, 136.4, 135.6, 133.8, 130.0, 127.7, 127.4, 122.6, 122.0, 112.4, 109.2, 104.5, 77.8, 76.5, 74.0, 72.8, 72.1, 60.2, 53.8, 53.7, 28.3, 26.9, 26.3, 26.3, 21.7 ppm; **IR** (ATR) $\tilde{\nu}$ = 667 (s), 731 (s), 813 (m), 909 (m), 973 (m), 1018 (m), 1081 (s), 1163 (s), 1219 (m), 1342 (m), 1372 (m) ,1457 (w), 1595 (w), 1733 (w), 2930 (w), 2986 (w) cm^{-1}; **HRMS** (ESI): calcd. for $C_{28}H_{35}NO_8Na^+$: 568.1976; found: 568.1987; ***R*f** = 0.40 (isohexane/ethyl acetate – 2/1).

4.2 Substrate Synthesis

4.2.1 Preparation of 4-Methyl-*N*-(prop-2-yn-1-yl)benzenesulfonamide **S7**

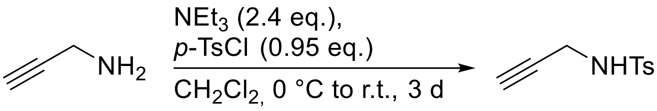

S6 **S7**

Preparation of 4-methyl-*N*-(prop-2-yn-1-yl)benzenesulfonamide **S7**

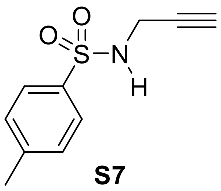

S7

The compound was prepared according to a literature-known procedure.[3] The reaction was set up under air without anhydrous solvents. Propargylamine **S6** (6.4 mL, 101 mmol, 1.0 eq.) was dissolved in dichloromethane (240 mL) at 0 °C in a single-necked round-bottomed flask. Triethylamine (33.3 mL, 240 mmol, 2.4 eq.) and *p*-toluenesulphonyl chloride (18.1 g, 95.0 mmol, 0.95 eq.) were added at 0 °C, then the reaction mixture was warmed to room temperature and stirred for 3 days. After dilution with diethyl ether, the mixture was washed with dilute hydrochloric acid (1 N), a saturated aqueous ammonium chloride solution and a saturated aqueous sodium chloride solution. The organic layer was dried over magnesium sulphate and the solvent was removed under reduced pressure. The title compound was isolated in a yield of 18.2 g (87.4 mmol, 92%, calcd. on *p*-TsCl) as a pale yellow solid.

^{1}H NMR (300 MHz, $CDCl_3$) δ = 7.84–7.72 (m, 2H), 7.31 (d, J = 8.0 Hz, 2H), 4.64 (t, J = 5.4 Hz, 1H), 3.83 (dd, J = 6.0, 2.5 Hz, 2H), 2.43 (s, 3H), 2.10 (t, J = 2.5 Hz, 1H) ppm; **^{13}C NMR** (75 MHz, $CDCl_3$) δ = 144.0, 136.7, 129.9, 127.6, 78.1, 73.2, 33.0, 21.7 ppm; **IR** (ATR) $\tilde{\nu}$ = 663 (s), 868 (m), 1092 (m), 1155 (s), 1319 (s), 1435 (m), 3265 (s) cm^{-1}; **MS** (EI): *m*/*z* (%): 209 (1), 155 (18), 144 (30), 139 (13), 132 (17), 130 (27), 118 (36), 116 (13), 92 (40), 91 (100), 89 (16), 77 (10), 65 (43), 54 (27); $\boldsymbol{R_f}$ = 0.10 (isohexane/ethyl acetate – 5/1).

The analytical data are in good accordance with the literature.[9]

4.2.2 Preparation of Methyl 4-((4-methyl-*N*-(prop-2-yn-1-yl)phenyl)sulfonamido)but-2-ynoate **S8** and *N*-(4-Methoxybut-2-yn-1-yl)-4-methyl-*N*-(prop-2-yn-1-yl)benzenesulfonamide **S9**

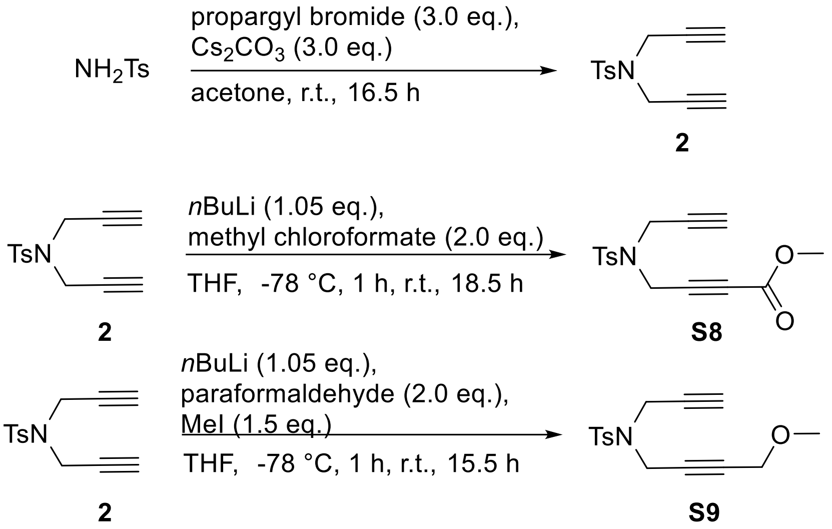

Preparation of 4-methyl-*N*,*N*-di(prop-2-yn-1-yl)benzenesulfonamide **2**

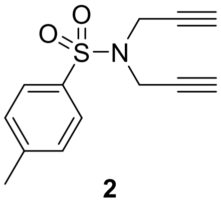

2

The compound was prepared according to a literature-known procedure.[10] To the suspension of 4-toluenesulfonamide (3.42 g, 20.0 mmol, 1.0 eq.) in acetone (50 mL), cesium carbonate (19.5 g, 60.0 mmol, 3.0 eq.) was added and stirred for 20 minutes at room temperature. To this, propargylbromide (80% in toluene, 6.7 mL, 60 mmol, 3.0 eq.) was added, and the mixture was stirred at room temperature for 16.5 hours. The solvent was removed in vacuo. The residual solid was dissolved in water, and dichlormethane was added. The layers were separated, and the aqueous layer was extracted with dichlormethane. The combined organic layers were dried over magnesium sulphate and the solvent was removed under reduced pressure. The title compound **2** was isolated after purification by silica gel column chromatography (isohexane/ethyl acetate – 20/1) in a yield of 3.18 g (12.9 mmol, 64%) as a colourless solid.

^{1}H NMR (300 MHz, $CDCl_3$) δ = 7.77–7.67 (m, 2H), 7.35–7.27 (m, 2H), 4.16 (d, J = 2.4 Hz, 4H), 2.42 (s, 3H), 2.15 (t, J = 2.3 Hz, 2H) ppm; **^{13}C NMR** (75 MHz, $CDCl_3$) δ = 144.1, 135.3, 129.7, 128.0, 76.3, 74.2, 36.3, 21.7 ppm; **IR** (ATR) $\tilde{\nu}$ = 753 (m), 813 (m), 887 (m), 954 (w), 1092 (s), 1159 (s), 1252 (w), 1349 (m), 1435 (w), 1595 (w), 3284 (m), 3284 (m) cm^{-1}; **MS** (EI): *m*/*z* (%):

247 (5), 182 (20), 168 (13), 155 (27), 139 (13), 130(6), 91 (100), 65 (43); $\boldsymbol{R_f}$ = 0.20 (isohexane/ethyl acetate – 20/1).

The analytical data are in good accordance with the literature.[11]

Preparation of methyl 4-((4-methyl-*N*-(prop-2-yn-1-yl)phenyl)sulfonamido)but-2-ynoate **S8**

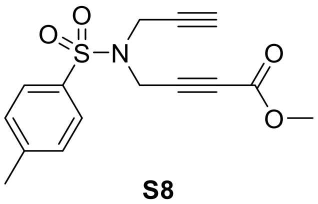

S8

Tosylamid **2** (742 mg, 3.00 mmol, 1.0 eq.) was dissolved in anhydrous tetrahydrofurane (6 mL) the resulting solution was cooled to −78 °C. *n*-butyllithium (2.0 mL, 3.2 mmol, 1.05 eq., 1.6 M in hexanes) was added dropwise. The reaction mixture was was stirred for 1 h at −78 °C. Methyl chloroformate (0.46 mL, 6.0 mmol, 2.0 eq.) was added dropwise. The reaction mixture was warmed to room temperature and was stirred for 18.5 hours. The reaction was quenched by addition of water. The aqueous layer was extracted with ethyl acetat and the combined organic layers were washed with a saturated aqueous sodium chloride solution and dried over magnesium sulphate. The volatiles were removed under reduced pressure and the crude product was purified by silica gel column chromatography (isohexane/ethyl acetate – 5/1). The product was obtained as a yellow solid in a yield of 310 mg (1.01 mmol, 34%).

^{1}H NMR (400 MHz, $CDCl_3$) δ = 7.75–7.67 (m, 2H), 7.34–7.28 (m, 2H), 4.30 (s, 2H), 4.14 (d, J = 2.4 Hz, 2H), 3.72 (s, 3H), 2.42 (s, 3H), 2.19 (t, J = 2.5 Hz, 1H) ppm; **^{13}C NMR** (101 MHz, $CDCl_3$) δ = 153.1, 144.5, 134.8, 129.9, 128.0, 80.2, 77.2, 75.9, 74.8, 52.9, 36.9, 36.3, 21.7 ppm; **IR** (ATR) $\tilde{\nu}$ = 749 (m), 816 (m), 895 (m), 943 (w), 1059 (m), 1096 (m), 1163 (s), 1252 (s), 1349 (m), 1435 (m), 1595 (w), 1715 (s), 2244 (w), 3280 (w) cm^{-1}; **HRMS** (ESI): calcd. for $C_{15}H_{15}NO_4SNa^+$: 328.0614; found: 328.0620; $\boldsymbol{R_f}$ = 0.19 (isohexane/ethyl acetate – 5/1).

Preparation of *N*-(4-methoxybut-2-yn-1-yl)-4-methyl-*N*-(prop-2-yn-1-yl)benzenesulfonamide **S9**

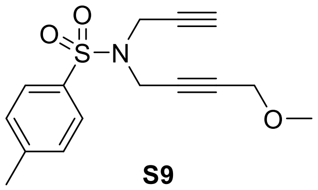

S9

Tosylamid **2** (742 mg, 3.00 mmol, 1.0 eq.) was dissolved in anhydrous tetrahydrofurane (6 mL) the resulting solution was cooled to −78 °C. *n*-butyllithium (2.0 mL, 3.15 mmol, 1.05 eq., 1.6 M in hexanes) was added dropwise. The reaction mixture was was stirred for 1 hour at −78 °C. Paraformaldehyde (180 mg, 6.00 mmol, 2.0 eq.) was added in one portion. The reaction mixture was warmed to room temperature and was stirred for 30 minutes, after this period iodomethane (0.28 mL, 4.5 mmol, 1.5 eq.) was added. The reaction mixture was stirred for 15.0 hours. The reaction was quenched by addition of a saturated aqueous sodium hydrogencarbonate solution. The aqueous layer was extracted with ethyl acetat and the combined organic layers were washed with a saturated aqueous sodium chloride solution and dried over magnesium sulphate. The volatiles were removed under reduced pressure and the crude product was purified by silica gel column chromatography (isohexane/ethyl acetate – 8/1). The product was obtained as a yellow liquid in a yield of 172 mg (591 µmol, 20%).

^{1}H NMR (300 MHz, $CDCl_3$) δ = 7.75–7.65 (m, 2H), 7.35–7.27 (m, 2H), 4.21 (t, J = 1.6 Hz, 2H), 4.15 (d, J = 2.4 Hz, 2H), 3.94 (t, J = 1.9 Hz, 2H), 3.26 (s, 3H), 2.42 (s, 3H), 2.14 (t, J = 2.5 Hz, 1H) ppm; **^{13}C NMR** (75 MHz, $CDCl_3$) δ = 144.1, 135.4, 129.7, 128.1, 81.8, 79.0, 76.4, 74.1, 59.8, 57.7, 36.6, 36.5, 21.7 ppm; **IR** (ATR) $\tilde{\nu}$ = 749 (m), 816 (m), 895 (m), 950 (w), 1092 (s), 1159 (s), 1252 (w), 1349 (m), 1439 (w), 1495 (w), 1595 (w), 2848 (w), 2930 (w), 3276 (w) cm^{-1}; **HRMS** (ESI): calcd. for $C_{15}H_{17}NO_3SNa^+$: 314.0821; found: 314.0826; $\boldsymbol{R}_f$ = 0.23 (isohexane/ethyl acetate – 8/1).

4.2.3 Preparation of 5,5-dimethyl-2,2-di(prop-2-yn-1-yl)cyclohexane-1,3-dione **S11**

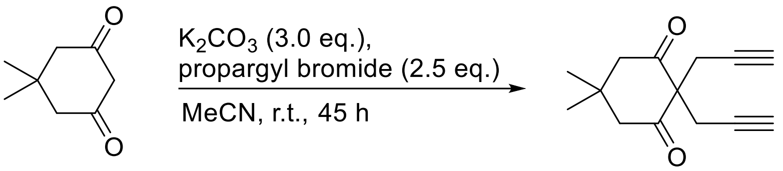

S10 **S11**

Preparation of 5,5-dimethyl-2,2-di(prop-2-yn-1-yl)cyclohexane-1,3-dione **S11**

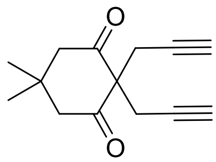

S11

The compound was prepared according to a literature-known procedure.[12] Dimedone **S10** (1.40 g, 10.0 mmol, 1.0 eq.) and potassium carbonate (4.14 g, 30.0 mmol, 3.0 eq.) were suspended in acetonitrile (20 mL). Propargyl bromide (80% in toluene, 2.8 mL, 25 mmol, 2.5 eq.)

was added and the reaction mixture was stirred at room temperature for 45 hours. After filtration (silica), the volatiles were removed under reduced pressure and the crude product was diluted with dichlormethane and washed with water. The aqueous layer was extracted with dichlormethane and the combined organic layers were washed with a saturated aqueous sodium chloride solution and dried over magnesium sulphate. The solvent was removed under reduced pressure. The title compound **S11** was isolated after purification by silica gel column chromatography (isohexane/ethyl acetate – 8/1 to 5/1) in a yield of 1.40 g (6.45 mmol, 65%) as a colourless solid.

^{1}H NMR (300 MHz, $CDCl_3$) δ = 2.72–2.63 (m, 8H), 2.07 (t, J = 2.7 Hz, 2H), 1.06 (s, 6H) ppm; **^{13}C NMR** (75 MHz, $CDCl_3$) δ = 206.2, 79.1, 72.6, 66.0, 52.2, 30.8, 28.9, 24.3 ppm; **IR** (ATR) $\tilde{\nu}$ = 1073 (w), 1204 (m), 1256 (w), 1327 (m), 1416 (m), 1469 (w), 1696 (s), 1729 (m), 2960 (w), 3280 (m) cm^{-1}; **HRMS** (ESI): calcd. for $C_{14}H_{16}O_2Na^+$: 239.1043; found: 239.1049; ***R*f** = 0.30 (isohexane/ethyl acetate – 5/1).

The analytical data are in good accordance with the literature.[11]

4.2.4 Preparation of dimethyl 2,2-di(prop-2-yn-1-yl)malonate **S13**

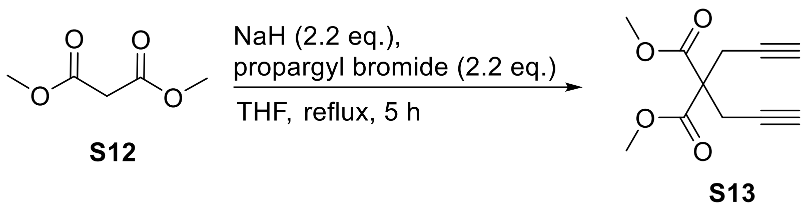

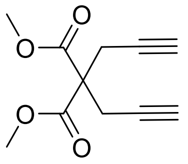

S13

The compound was prepared according to a literature-known procedure.[13] NaH (60 % in mineral oil, 880 mg, 22.0 mmol, 2.2 eq.) was added to anhydrous tetrahydrofurane (10 mL). The suspension was cooled down to 0 °C and dimethyl malonate **S12** (1.1 mL, 9.6 mmol, 1.0 eq.) was added. Stirring was continued for 20 minutes, after that propargyl bromide (80% in toluene, 2.5 mL, 22 mmol, 2.2 eq.) was added dropwise. The reaction mixture was refluxed for 4.5 hours. The reaction was quenched by addition of a saturated aqueous ammonium chloride solution, and extracted with diethyl ether. The combined organic layers were washed with a

saturated aqueous sodium chloride solution and dried over magnesium sulphate and the solvent was removed under reduced pressure. The title compound **S13** was isolated after purification by silica gel column chromatography (isohexane/ethyl acetate – 15/1) in a yield of 1.69 g (8.11 mmol, 81%) as a colourless solid.

^{1}H NMR (400 MHz, $CDCl_3$) δ = 3.74 (s, 6H), 2.97 (d, J = 2.7 Hz, 4H), 2.02 (t, J = 2.6 Hz, 2H) ppm; **^{13}C NMR** (101 MHz, $CDCl_3$) δ = 169.1, 78.4, 71.9, 56.5, 53.2, 22.7 ppm; **IR** (ATR) $\tilde{\nu}$ = 671 (s), 850 (m), 932 (w), 977 (m), 1059 (m), 1200 (s), 1245 (m), 1293 (s), 1319 (m), 1435 (m), 1737 (s), 3276 (m) cm^{-1}; **HRMS** (ESI): calcd. for $C_{11}H_{12}O_4Na^+$: 231.0628; found: 231.0634; $\boldsymbol{R_f}$ = 0.34 (isohexane/ethyl acetate – 10/1).

The analytical data are in good accordance with the literature.[11]

4.2.5 Preparation of 4-methyl-*N*-(4-methylpent-2-yn-1-yl)-*N*-(prop-2-yn-1-yl)benzenesulfonamide **S16**

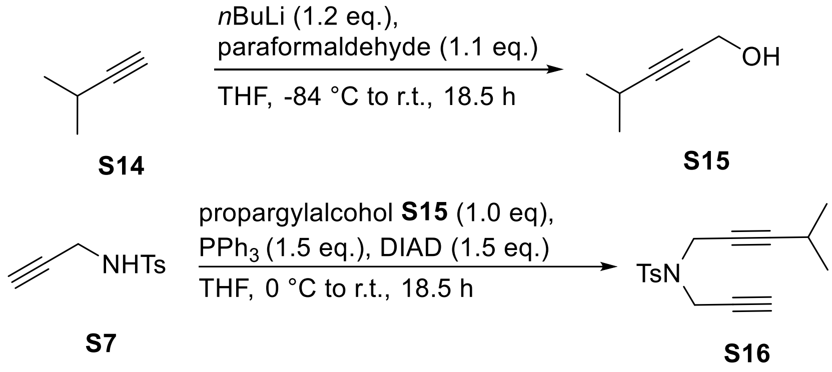

Preparation of 4-methylpent-2-yn-1-ol **S15**

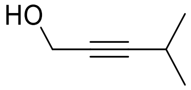

S15

The compound was prepared according to a literature-known procedure.[13] A solution of *n*-butyllithium (6.9 mL, 11 mmol, 1.2 eq., 1.6 M in hexanes) was added dropwise to a solution of 3-methyl-1-butyne **S14** (1.0 mL, 10 mmol, 1.0 eq.) in anhydrous tetrahydrofuran (25 mL) at −84 °C. The mixture was stirred at −84 °C for 30 minutes. Paraformaldehyde (330 mg, 11.0 mmol, 1.1 eq.) was added, the reaction mixture was warmed to room temperature and stirred for 18.5 hours. The reaction was quenched by addition of a saturated aqueous sodium hydrogencarbonate solution and the layers were separated. The aqueous layer was extracted with ethyl acetate. The combined organic layers were washed with a saturated aqueous sodium chloride solution and dried over magnesium sulphate. The solvent was removed under

reduced pressure, the title compound was purified by silica gel column chromatography (isohexane/ethyl acetate – 5/1) and was obtained in a yield of 432 mg (4.40 mmol, 44%) as a yellow liquid.

^{1}H NMR (300 MHz, $CDCl_3$) δ = 4.24 (d, J = 3.0 Hz, 2H), 2.73–2.42 (m, 1H), 1.66 (t, J = 5.7 Hz, 1H), 1.16 (d, J = 6.9 Hz, 6H) ppm; **^{13}C NMR** (75 MHz, $CDCl_3$) δ = 92.1, 51.5, 23.0, 20.6 ppm (one carbon resonance is missing, probably due to overlap with the solvent signal); **IR** (ATR) $\tilde{\nu}$ = 708 (m), 876 (w), 999 (s), 1051 (m), 1126 (m), 1185 (m), 1319 (m), 1364 (m), 1461 (m), 2874 (w), 2933 (w), 2971 (m), 3321 (w) cm^{-1}; **MS** (EI): *m/z* (%): 98 (10), 83 (100), 79 (33), 69 (49), 55 (89); ***R*$_f$** = 0.30 (isohexane/ethyl acetate – 5/1).

The analytical data are in good accordance with the literature.[13]

Preparation of 4-methyl-*N*-(4-methylpent-2-yn-1-yl)-*N*-(prop-2-yn-1-yl)benzenesulfonamide **S16**

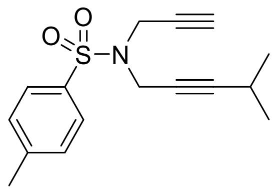

S16

Tosylamide **S7** (419 mg, 2.00 mmol, 1.0 eq.), propargylalcohol **S15** (196 mg, 2.00 mmol, 1.0 eq.) and triphenylphosphine (787 g, 3.00 mmol, 1.5 eq.) were dissolved in anhydrous tetrahydrofuran (5 mL) and the resulting solution was cooled to 0 °C. DIAD (0.59 mL, 3.0 mmol, 1.5 eq.) was added dropwise. The reaction mixture was warmed to room temperature and was stirred for 18.5 hours. The reaction was quenched by addition of a saturated aqueous ammonium chloride solution. The aqueous layer was extracted with ethyl acetate and the combined organic layers were washed with a saturated aqueous sodium chloride solution and dried over magnesium sulphate. The volatiles were removed under reduced pressure and the crude product was purified by silica gel column chromatography (isohexane/ethyl acetate – 10/1). The product was obtained as a colourless liquid in a yield of 486 mg (1.68 mmol, 84%).

^{1}H NMR (300 MHz, $CDCl_3$) δ = 7.78–7.51 (m, 2H), 7.37–7.04 (m, 2H), 4.11 (d, J = 2.1 Hz, 2H), 4.08 (d, J = 2.5 Hz, 2H), 2.38 (s, 3H), 2.35–2.25 (m, 1H), 2.13 (t, J = 2.5 Hz, 1H), 0.95 (d, J = 6.9 Hz, 6H) ppm; **^{13}C NMR** (75 MHz, $CDCl_3$) δ = 143.8, 135.5, 129.5, 127.9, 92.1, 76.6, 73.7, 71.1, 36.8, 36.1, 22.6, 21.5, 20.3 ppm; **IR** (ATR) $\tilde{\nu}$ = 708 (m), 753 (m), 813 (m), 895 (m), 950 (w), 1044 (w), 1092 (s), 1159 (s), 1252 (w), 1349 (m), 1439 (w), 1595 (w), 1707 (w), 2930 (w), 2974 (w), 3280 (w) cm^{-1}; **HRMS** (ESI): calcd. for $C_{16}H_{19}NO_2SNa^+$: 312.1029; found: 312.1023; ***R*$_f$** = 0.34 (isohexane/ethyl acetate – 10/1).

The analytical data are in good accordance with the literature.[14]

4.2.6 Preparation of 4-methyl-*N*-(5-phenylpent-2-yn-1-yl)-*N*-(prop-2-yn-1-yl)benzenesulfonamide **S19**

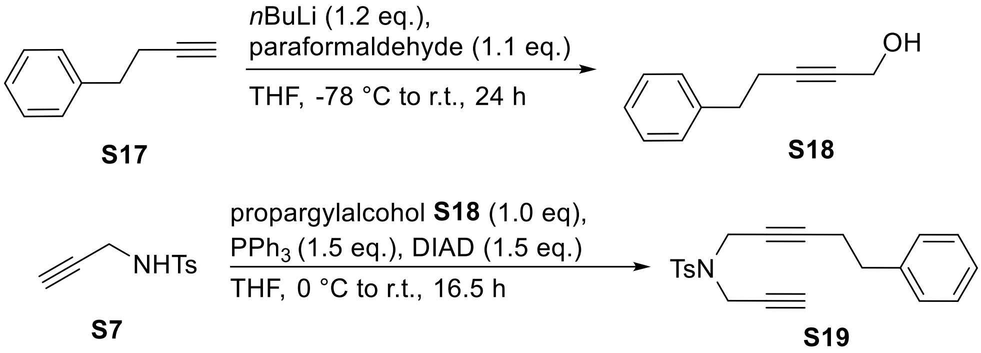

Preparation of 5-phenylpent-2-yn-1-ol **S18**

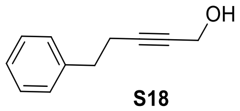

S18

The compound was prepared according to a literature-known procedure.[13] A solution of *n*-butyllithium (3.8 mL, 6.0 mmol, 1.2 eq., 1.6 M in hexanes) was added dropwise to a solution of 3-butyn-1-ylbenzene **S17** (0.70 mL, 5.0 mmol, 1.0 eq.) in anhydrous tetrahydrofuran (25 mL) at −78 °C. The mixture was stirred at −78 °C for 30 minutes. Paraformaldehyde (165 mg, 5.50 mmol, 1.1 eq.) was added, the reaction mixture was warmed to room temperature and stirred for 23.5 hours. The reaction was quenched by addition of a saturated aqueous sodium hydrogencarbonate solution and the layers were separated. The aqueous layer was extracted with ethyl acetate. The combined organic layers were washed with a saturated aqueous sodium chloride solution and dried over magnesium sulphate. The solvent was removed under reduced pressure, the title compound **S18** was purified by silica gel column chromatography (isohexane/ethyl acetate – 5/1) and was obtained in a yield of 635 mg (3.96 mmol, 79%) as a yellow liquid.

^{1}H NMR (300 MHz, $CDCl_3$) δ = 7.36–7.27 (m, 2H), 7.25–7.15 (m, 3H), 4.23 (t, J = 2.2 Hz, 2H), 2.84 (t, J = 7.6 Hz, 2H), 2.59–2.45 (m, 2H) ppm; **^{13}C NMR** (75 MHz, $CDCl_3$) δ = 140.7, 128.6, 128.5, 126.5, 85.9, 79.2, 51.5, 35.1, 21.0 ppm; **IR** (ATR) $\tilde{\nu}$ = 697 (s), 745 (m), 1006 (m), 1133 (m), 1342 (w), 1454 (m), 1495 (w), 2863 (w), 2922 (w), 3325 (w) cm^{-1}; **MS** (EI): *m*/*z* (%): 160

(1), 142 (52), 129 (23), 116 (10), 91 (100), 77 (5), 65 (20); $\boldsymbol{R_f}$ = 0.17 (isohexane/ethyl acetate – 5/1).

The analytical data are in good accordance with the literature.[13]

Preparation of 4-methyl-*N*-(5-phenylpent-2-yn-1-yl)-*N*-(prop-2-yn-1-yl)benzenesulfonamide **S19**

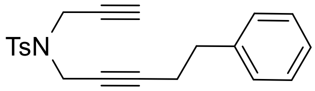

S19

Tosylamide **S7** (845 mg, 4.00 mmol, 1.0 eq.), propargylalcohol **S18** (641 mg, 4.00 mmol, 1.0 eq.) and triphenylphosphine (1.57 g, 6.00 mmol, 1.5 eq.) were dissolved in anhydrous tetrahydrofuran (10 mL) and the resulting solution was cooled to 0 °C. DIAD (1.2 mL, 6.0 mmol, 1.5 eq.) was added dropwise. The reaction mixture was warmed to room temperature and was stirred for 16.5 hours. The reaction was quenched by addition of a saturated solution of a saturated aqueous ammonium chloride solution. The aqueous layer was extracted with ethyl acetate and the combined organic layers were washed with a saturated aqueous sodium chloride solution and dried over magnesium sulphate. The volatiles were removed under reduced pressure and the crude product was purified by silica gel column chromatography (isohexane/ethyl acetate – 10/1). The product **S19** was obtained as a yellow oil in a yield of 1.35 g (3.83 mmol, 96%).

^{1}H NMR (400 MHz, $CDCl_3$) δ = 7.73–7.65 (m, 2H), 7.32–7.24 (m, 4H), 7.25–7.17 (m, 1H), 7.17–7.05 (m, 2H), 4.12 (t, *J* = 2.1 Hz, 2H), 4.02 (d, *J* = 2.4 Hz, 2H), 2.65 (t, *J* = 7.4 Hz, 2H), 2.41 (s, 3H), 2.31 (tt, *J* = 7.5, 2.2 Hz, 2H), 2.13 (t, *J* = 2.5 Hz, 1H) ppm; **^{13}C NMR** (101 MHz, $CDCl_3$) δ = 143.8, 140.4, 135.5, 129.6, 128.5, 128.5, 128.1, 126.5, 85.8, 76.7, 73.8, 73.1, 36.8, 36.1, 34.8, 21.7, 20.8 ppm; **IR** (ATR) $\tilde{\nu}$ = 813 (m), 895 (m), 954 (w), 1092 (m), 1159 (s), 1252 (w), 1349 (m), 1431 (w), 1495 (w), 1595 (w), 2922 (w), 3027 (w), 3280 (w) cm^{-1}; **HRMS** (ESI): calcd. for $C_{21}H_{21}NO_2SNa^+$: 374.1185; found: 374.1178; $\boldsymbol{R_f}$ = 0.21 (isohexane/ethyl acetate – 10/1).

4.2.7 Preparation of *N*-(6-((tert-butyldimethylsilyl)oxy)hex-2-yn-1-yl)-4-methyl-*N*-(prop-2-yn-1-yl)benzenesulfonamide **S24**

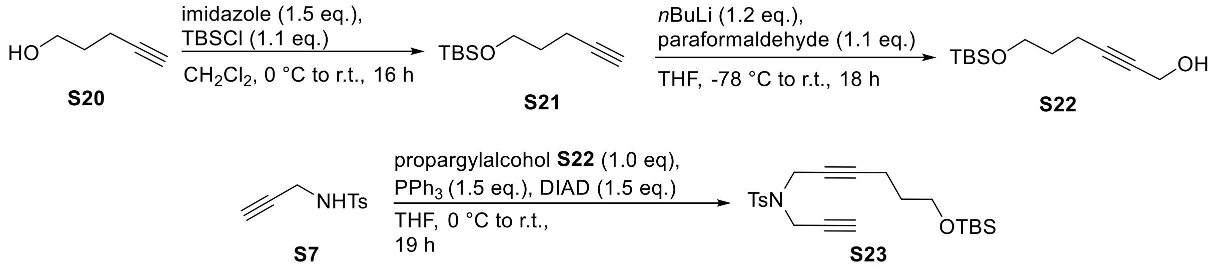

Preparation of tert-butyldimethyl(pent-4-yn-1-yloxy)silane **S21**

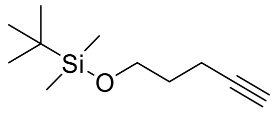

S21

The compound was prepared according to a literature-known procedure.[13] To a solution of imidazole (5.11 g, 75.0 mmol, 1.5 eq.) and 4-pentyn-1-ol **S20** (4.7 mL, 50 mmol, 1.0 eq.) in dichlormethane (50 mL) was added TBSCl (8.23 g, 55.0 mmol, 1.1 eq.) at 0°C. The reaction was warmed to room temperature and stirred for 16 hours. Then the solution was filtered through a plug of silica and washed with ethyl acetate. The solvent was removed under reduced pressure, the title compound was purified by silica gel column chromatography (isohexane/ethyl acetate – 50/1) and was obtained in a yield of 9.27 g (46.7 mmol, 93%) as a colourless liquid.

^{1}H NMR (300 MHz, $CDCl_3$) δ = 3.70 (t, J = 6.0 Hz, 2H), 2.27 (td, J = 7.1 Hz, 2.7 Hz, 2H), 1.93 (t, J = 2.7 Hz, 1H), 1.79–1.63 (m, 2H), 0.89 (s, 9H), 0.06 (s, 6H) ppm; **^{13}C NMR** (75 MHz, $CDCl_3$) δ = 84.4, 68.4, 61.6, 31.7, 26.1, 18.5, 15.0, -5.2 ppm; **IR** (ATR) $\tilde{\nu}$ = 716 (w), 775 (s), 831 (s), 939 (w), 980 (m), 1103 (m), 1193 (w), 1252 (m), 1360 (w), 1387 (w), 1435 (w), 1472 (w), 2 859 (w), 2889 (w), 2952 (m), 3314 (w) cm^{-1}; **MS** (EI): *m*/*z* (%): 199 (1), 155 (3), 141 (100), 111 (30) 99 (8), 83 (9), 75 (75); ***R*$_f$** = 0.17 (isohexane/ethyl acetate – 50/1).

The analytical data are in good accordance with the literature.[13]

Preparation of 6-((tert-butyldimethylsilyl)oxy)hex-2-yn-1-ol **S22**

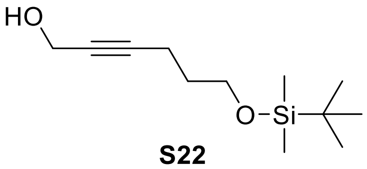

S22

The compound was prepared according to a literature-known procedure.[13] A solution of *n*-butyllithium (30.0 mL, 48.0 mmol, 1.2 eq., 1.6 M in hexanes) was added dropwise to a solution of alkyne **S21** (7.94 g, 40.0 mmol, 1.0 eq.) in anhydrous tetrahydrofuran (100 mL) at −78 °C. The mixture was stirred at −78 °C for 30 minutes. Paraformaldehyde (3.60 g, 120 mmol, 3.0 eq.) was added, the reaction mixture was warmed to room temperature and stirred for 17.5 hours. The reaction was quenched by addition of a saturated aqueous sodium hydrogencarbonate solution and the layers were separated. The aqueous layer was extracted with ethyl acetate. The combined organic layers were washed with a saturated aqueous sodium chloride solution and dried over magnesium sulphate. The solvent was removed under reduced pressure, the title compound was purified by silica gel column chromatography (isohexane/ethyl acetate – 10/1) and was obtained in a yield of 7.38 g (32.3 mmol, 81%) as a yellow liquid.

^{1}H NMR (300 MHz, $CDCl_3$) δ = 4.24 (t, J = 2.2 Hz, 2H), 3.68 (t, J = 6.0 Hz, 2H), 2.30 (tt, J = 7.1 Hz, 2.2 Hz, 2H), 1.77–1.64 (m, 2H), 1.60 (s, 1H), 0.89 (s, 9H), 0.05 (s, 6H) ppm; **^{13}C NMR** (75 MHz, $CDCl_3$) δ = 86.4, 78.6, 61.7, 51.6, 31.8, 26.1, 18.5, 15.3, -5.2 ppm; **IR** (ATR) $\tilde{\nu}$ = 779 (m), 835 (s), 1006 (s), 1051 (s), 1141 (m), 1252 (m), 1360 (m), 1431 (m), 1469 (w), 1711 (w), 2859 (m), 2930 (m), 3306 (m) cm^{-1}; **HRMS** (ESI): calcd for $C_{12}H_{25}O_2Si^+$: 229.1618; found: 229.1620; $\boldsymbol{R_f}$ = 0.11 (isohexane/ethyl acetate – 10/1).

The analytical data are in good accordance with the literature.[13]

Preparation of *N*-(6-((tert-butyldimethylsilyl)oxy)hex-2-yn-1-yl)-4-methyl-*N*-(prop-2-yn-1-yl)benzenesulfonamide **S23**

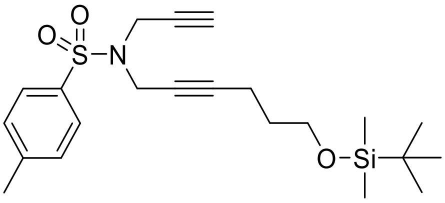

S23

Tosylamide **S7** (2.06 g, 9.84 mmol, 1.0 eq.), propargylalcohol **S22** (2.25 g, 9.84 mmol, 1.0 eq.) and triphenylphosphine (3.87 g, 14.8 mmol, 1.5 eq.) were dissolved in anhydrous tetrahydrofuran (25 mL) and the resulting solution was cooled to 0 °C. DIAD (2.9 mL, 15 mmol, 1.5 eq.) was added dropwise. The reaction mixture was warmed to room temperature and was stirred for 19 hours. The reaction was quenched by addition of a saturated solution of a saturated aqueous ammonium chloride solution. The aqueous layer was extracted with ethyl acetate and the combined organic layers were washed with a saturated aqueous sodium chloride solution and dried over magnesium sulphate. The volatiles were removed under reduced pressure and the crude product was purified by silica gel column chromatography (isohexane/ethyl acetate – 10/1). The product was obtained as a colourless liquid in a yield of 2.62 mg (6.23 mmol, 63%).

^{1}H NMR (300 MHz, $CDCl_3$) δ = 7.75–7.65 (m, 2H), 7.33–7.20 (m, 2H), 4.18–4.06 (m, 4H), 3.56 (t, J = 6.0 Hz, 2H), 2.41 (s, 3H), 2.20–1.96 (m, 3H), 1.61–1.45 (m, 2H), 0.88 (s, 9H), 0.03 (s, 6H) ppm; **^{13}C NMR** (101 MHz, $CDCl_3$) δ = 143.9, 135.5, 129.6, 128.1, 85.8, 76.6, 73.9, 72.8, 61.6, 36.8, 36.3, 31.5, 26.0, 21.6, 18.4, 15.1, –5.3 ppm; **IR** (ATR) $\tilde{\nu}$ = 704 (m), 749 (s), 775 (s), 835 (s), 895 (s), 958 (m), 1006 (w), 1096 (s), 1163 (s), 1252 (m), 1349 (s), 1387 (w), 1435 (w), 1469 (w), 1595 (w), 1774 (w), 2855 (m), 2889 (w), 2952 (m) cm^{-1}; **HRMS** (ESI): calcd. for $C_{22}H_{33}NO_3SSiNa^+$: 442.1843; found: 442.1847; $\boldsymbol{R_f}$ = 0.31 (isohexane/ethyl acetate – 10/1).

4.2.8 Preparation of Ferrocene Diyne **S26**

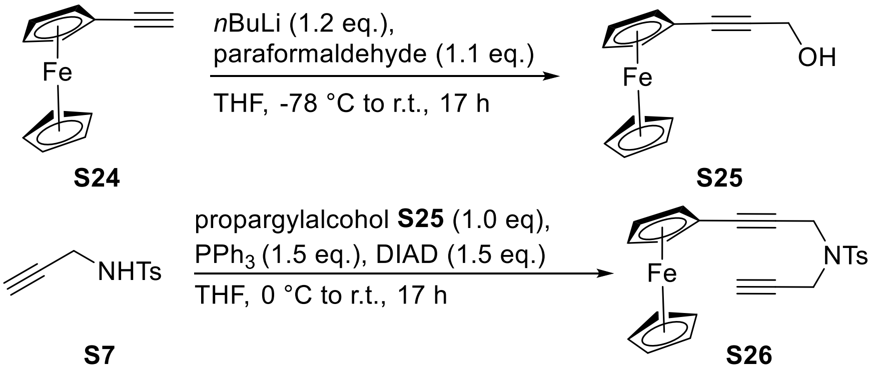

Preparation of (3-Hydroxy-1-propyn-1-yl)ferrocene **S25**

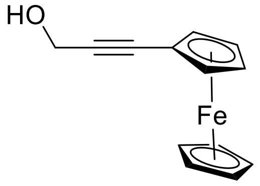

S25

A solution of *n*-butyllithium (2.3 mL, 3.6 mmol, 1.2 eq., 1.6 M in hexanes) was added dropwise to a solution of ethynylferrocene **S24** (630 mg, 3.00 mmol, 1.0 eq.) in anhydrous tetrahydrofuran (15 mL) at −78 °C. The mixture was stirred at −78 °C for 30 minutes. Paraformaldehyde (99.1 mg, 3.30 mmol, 1.1 eq.) was added, the reaction mixture was warmed to room temperature and stirred for 16.5 hours. The reaction was quenched by addition of a saturated aqueous sodium hydrogencarbonate solution and the layers were separated. The aqueous layer was extracted with diethyl ether. The combined organic layers were washed with a saturated aqueous sodium chloride solution and dried over magnesium sulphate. The solvent was removed under reduced pressure, the title compound was purified by silica gel column chromatography (isohexane/ethyl acetate – 5/1) and was obtained in a yield of 619 mg (2.58 mmol, 86%) as a red solid.

^{1}H NMR (300 MHz, $CDCl_3$) δ = 4.46–4.34 (m, 4H), 4.29–4.12 (m, 7H), 1.71 (t, *J* = 6.0 Hz, 1H) ppm; **^{13}C NMR** (75 MHz, $CDCl_3$) δ = 84.7, 83.9, 71.6, 70.1, 68.9, 64.5, 52.0 ppm; **IR** (ATR) $\tilde{\nu}$ = 820 (s), 876 (w), 965 (m), 1003 (s), 1059 (m), 1103 (m), 1260 (m), 1353 (m), 1409 (m), 1461 (m), 2236 (m), 2863 (m), 2919 (m), 3094 (m), 3302 (m) cm^{-1}; **HRMS** (ESI): calcd. for $C_{13}H_{12}FeO^+$: 240.0232; found: 240.0239; $\boldsymbol{R_f}$ = 0.23 (isohexane/ethyl acetate – 5/1).

The analytical data are in good accordance with the literature.[15]

Preparation of Ferrocene Diyne **S26**

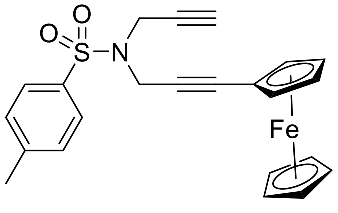

S26

Tosylamide **S7** (419 mg, 2.00 mmol, 1.0 eq.), propargylalcohol **S25** (480 mg, 2.00 mmol, 1.0 eq.) and triphenylphosphine (787 mg, 3.00 mmol, 1.5 eq.) were dissolved in anhydrous tetrahydrofuran (5 mL) and the resulting solution was cooled to 0 °C. DIAD (0.59 mL, 3.0 mmol, 1.5 eq.) was added dropwise. The reaction mixture was warmed to room temperature and was stirred for 17.0 hours. The reaction was quenched by addition of a saturated solution of a saturated aqueous ammonium chloride solution. The aqueous layer was extracted with diethyl ether and the combined organic layers were washed with a saturated aqueous sodium chloride

solution and dried over magnesium sulphate. The volatiles were removed under reduced pressure and the crude product was purified by silica gel column chromatography (isohexane/ethyl acetate – 5/1). The product was obtained as a red solid in a yield of 607 mg (1.41 mmol, 70%).

^{1}H NMR (300 MHz, $CDCl_3$) δ = 7.81–7.68 (m, 2H), 7.37–7.28 (m, 2H), 4.29 (s, 2H), 4.23 (t, *J* = 1.9 Hz, 2H), 4.20 (d, *J* = 2.4 Hz, 2H), 4.17–4.10 (m, 7H), 2.43 (s, 3H), 2.18 (t, *J* = 2.5 Hz, 1H) ppm; **^{13}C NMR** (75 MHz, $CDCl_3$) δ = 143.9, 135.6, 129.7, 128.1, 84.8, 77.7, 76.7, 74.0, 71.5, 70.0, 68.8, 64.11, 37.5, 36.4, 21.7 ppm; **IR** (ATR) $\tilde{\nu}$ = 749 (m), 816 (m), 898 (m), 954 (w), 1021 (w), 1092 (m), 1159 (s), 1260 (w), 1349 (m), 1435 (w), 1495 (w), 1595 (w), 2225 (w), 2922 (w), 2971 (w), 3094 (w), 3280 (w) cm^{-1}; **HRMS** (ESI): calcd. for $C_{23}H_{21}FeNO_2SNa^+$: 454.0535; found: 454.0538; $\boldsymbol{R}_f$ = 0.29 (isohexane/ethyl acetate – 5/1).

4.2.9 Preparation of *N*-(but-2-yn-1-yl)-4-methyl-*N*-(prop-2-yn-1-yl)benzenesulfonamide **S27**

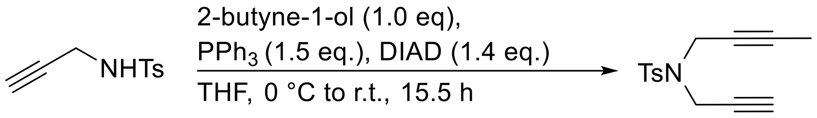

S7 **S27**

Preparation of *N*-(but-2-yn-1-yl)-4-methyl-*N*-(prop-2-yn-1-yl)benzenesulfonamide **S27**

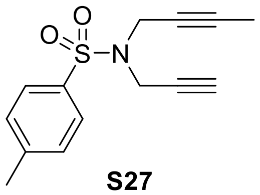

S27

Tosylamide **S7** (628 mg, 3.00 mmol, 1.0 eq.), 2-butyn-1-ol (0.22 mL, 3.0 mmol, 1.0 eq.) and triphenylphosphine (1.18 g, 4.50 mmol, 1.5 eq.) were dissolved in anhydrous tetrahydrofuran (15 mL) and the resulting solution was cooled to 0 °C. DIAD (0.82 mL, 4.2 mmol, 1.4 eq.) was added dropwise. The reaction mixture was warmed to room temperature and was stirred for 15.5 hours. The reaction was quenched by addition of a saturated solution of a saturated aqueous ammonium chloride solution. The aqueous layer was extracted with ethyl acetate and the combined organic layers were washed with a saturated aqueous sodium chloride solution and dried over magnesium sulphate. The volatiles were removed under reduced pressure and the crude product was purified by silica gel column chromatography (isohexane/ethyl acetate – 10/1). The product was obtained as a pale yellow oil in a yield of 538 mg (2.06 mmol, 69%).

^{1}H NMR (400 MHz, $CDCl_3$) δ = 7.75–7.65 (m, 2H), 7.32–7.27 (m, 2H), 4.13 (d, *J* = 2.3 Hz, 2H), 4.10 (q, *J* = 2.2 Hz, 2H), 2.42 (s, 3H), 2.13 (t, *J* = 2.5 Hz, 1H), 1.65 (t, *J* = 2.4 Hz, 3H) ppm; **^{13}C NMR** (101 MHz, $CDCl_3$) δ = 143.8, 135.5, 129.5, 128.1, 82.1, 76.7, 73.8, 71.4, 36.8, 36.3, 21.7,

3.5 ppm; **IR** (ATR) $\tilde{\nu}$ = 704 (m), 745 (m) ,813 (m), 895 (m), 928 (w), 954 (w), 1092 (s), 1159 (s), 1252 (w), 1349 (m), 1439 (w), 1495 (w), 1595 (w) ,1707 (w), 2851 (w), 2922 (w), 2967 (w), 3280 (w), 3280 (w) cm^{-1}; **HRMS** (ESI): calcd. for $C_{14}H_{16}NO_2S^+$: 262.0896; found: 262.0891; $\boldsymbol{R}_f$ = 0.20 (isohexane/ethyl acetate – 10/1).

The analytical data are in good accordance with the literature.[16]

4.2.10 Preparation of 3-phenyl-*N*-(prop-2-yn-1-yl)prop-2-yn-1-amine **S31**

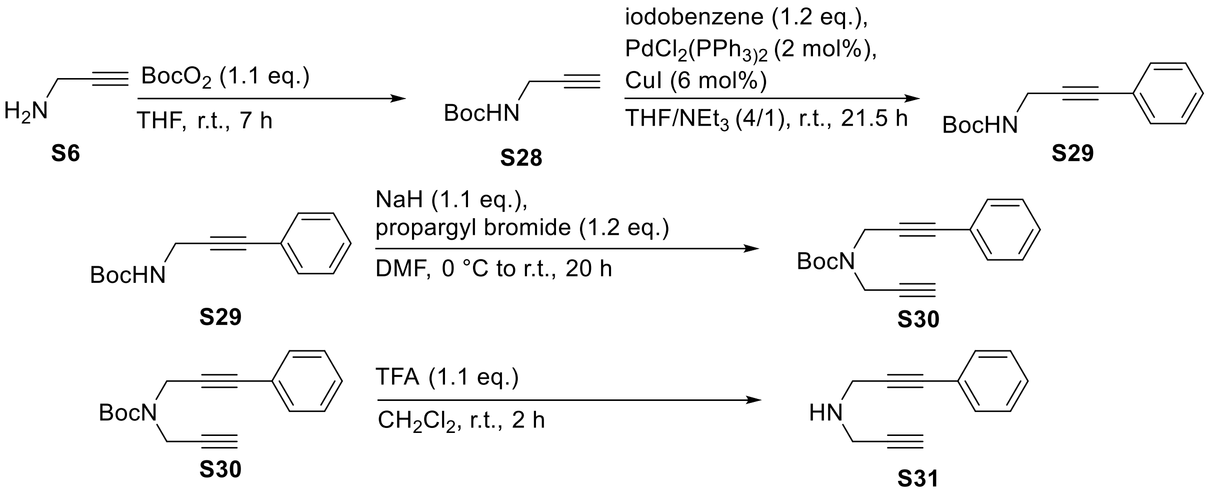

Preparation of tert-butyl prop-2-yn-1-ylcarbamate **S28**

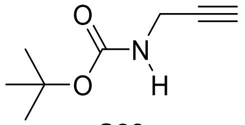

S28

The compound was prepared according to a literature-known procedure.[17] Propargylamine **S6** (1.3 mL, 20 mmol, 1.0 eq.) was dissolved in anhydrous tetrahydrofurane (15 mL) and di-*tert*-butyl dicarbonate (4.80 g, 22.0 mmol, 1.1 eq.) was added in one portion. The solution was stirred at room temperature for 7 hours.The reaction mixture was diluted with ethyl acetate und washed with water. The aqueous layer was extracted with ethyl acetate and the combined organic layers were washed with a saturated aqueous sodium chloride solution and dried over magnesium sulphate. The title compound was isolated after purification by silica gel column chromatography (isohexane/ethyl acetate – 10/1) in a yield of 2.76 mg (17.8 mmol, 89%) as a yellow liquid.

^{1}H NMR (300 MHz, $CDCl_3$) δ = 4.71 (s, 1H), 3.98–3.79 (m, 2H), 2.21 (t, J = 2.5 Hz, 1H), 1.45 (s, 9H) ppm; **^{13}C NMR** (101 MHz, $CDCl_3$) δ = 155.4, 80.2, 80.2, 71.4, 30.5, 28.5 ppm; **IR** (ATR) $\tilde{\nu}$ = 731 (m), 779 (m), 857 (m), 909 (w), 947 (m), 1051 (m), 1118 (m), 1163 (s), 1215 (m), 1249

(s) ,1368 (m), 1454 (m), 1506 (m), 1692 (s), 2933 (w), 2978 (w), 3302 (w) cm^{-1}; **MS** (EI): *m*/*z* (%): 155 (1), 112 (3), 99 (50), 81 (4), 59 (64), 57 (100); $\boldsymbol{R}_f$ = 0.36 (isohexane/ethyl acetate – 10/1).

The analytical data are in good accordance with the literature.[17]

Preparation of tert-butyl (3-phenylprop-2-yn-1-yl)carbamate **S29**

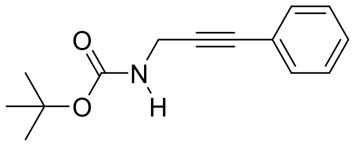

S29

The compound was prepared according to a literature-known procedure. A dried two-necked flask was charged with iodobenzene (0.67 mL, 6.0 mmol, 1.2 eq.), $PdCl_2(PPh_3)_2$ (70.2 mg, 0.10 mmol, 2 mol%) and copper(I)-iodide (57.1 mg, 0.30 mmol, 6 mol%). Anhydrous tetrahydrofurane (12 mL) and anhydrous triethylamine (3.0 mL) was added and the resulting slurry was stirred at room temperature for 5 minutes. Boc-protected propargylamid **S28** (776 mg, 5.00 mmol, 1.0 eq.) was added and the reaction mixture was stirred for 21.5 hours. The raction mixture was filtered over Celite and dried over magnesium sulphate. The solvent was removed under reduced pressure. The title compound was isolated after purification by silica gel column chromatography (isohexane/ethyl acetate – 10/1) in a yield of 879 mg (3.80 mmol, 76%) as a colourless solid.

^{1}H NMR (400 MHz, $CDCl_3$) δ = 7.46–7.37 (m, 2H), 7.34–7.27 (m, 3H), 4.79 (s, 1H), 4.15 (d, *J* = 4.6 Hz, 2H), 1.47 (s, 9H) ppm; **^{13}C NMR** (101 MHz, $CDCl_3$) δ = 155.4, 131.8, 128.5, 128.4, 122.8, 85.5, 83.2, 80.1, 31.4, 28.5 ppm; **IR** (ATR) $\tilde{\nu}$ =753 (s), 857 (m), 913 (m), 969 (w), 1047 (m), 1163 (s), 1245 (s), 1390 (m), 1491 (m), 1692 (s), 2974 (w), 3340 (w) cm^{-1}; **HRMS** (ESI): calcd. for $C_{14}H_{19}NO_2Na^+$: 254.1151; found: 254.1168; $\boldsymbol{R}_f$ = 0.57 (isohexane/ethyl acetate – 5/1).

The analytical data are in good accordance with the literature.[18]

Preparation of tert-butyl (3-phenylprop-2-yn-1-yl)(prop-2-yn-1-yl)carbamate **S30**

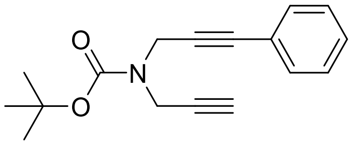

S30

Boc-protected propargylamid **S29** (694 mg, 3.00 mmol, 1.0 eq.) was dissolved in anhydrous dimethylformamide (10 mL). The solution was cooled down to 0 °C and NaH (60 % in mineral oil, 132 mg, 3.30 mmol, 1.1 eq.) was added. Stirring was continued for 30 minutes, subsequently propargyl bromide (80% in toluene, 0.39 mL, 3.6 mmol, 1.2 eq.) was added dropwise. The reaction mixture was warmed to room temperature and was stirred for 19.5 hours. The reaction was quenched by addition of water and extracted with diethyl ether. The combined organic layers were washed with Brine and dried over magnesium sulphate and the solvent was removed under reduced pressure. The title compound was isolated after purification by silica gel column chromatography (isohexane/ethyl acetate – 30/1) in a yield of 531.4 mg (1.97 mmol, 66%) as a yellow liquid.

^{1}H NMR (400 MHz, $CDCl_3$) δ = 7.48–7.37 (m, 2H), 7.34–7.27 (m, 3H), 4.39 (s, 2H), 4.23 (s, 2H), 2.24 (t, *J* = 2.4 Hz, 1H), 1.50 (s, 9H) ppm; **^{13}C NMR** (101 MHz, $CDCl_3$) δ = 154.5, 131.9, 128.5, 128.4, 122.9, 84.3, 81.2, 79.2, 71.9, 36.3, 35.4, 28.5 ppm (one carbon resonance is missing, probably due to overlap with the solvent signal); **IR** (ATR) $\tilde{\nu}$ = 690 (m), 757 (s) ,865 (m), 965 (w), 1029 (w), 1070 (w), 1118 (m) ,1159 (s), 1241 (s), 1401 (m), 1442 (m), 1491 (w), 1599 (w), 1696 (s), 2930 (w), 2974 (w), 3295 (w) cm^{-1}; **HRMS** (ESI): calcd for $C_{17}H_{14}ONa^{+}$: 292.1308; found: 292.1334; $\boldsymbol{R}_f$ = 0.67 (isohexane/ethyl acetate – 5/1).

Preparation of 3-phenyl-N-(prop-2-yn-1-yl)prop-2-yn-1-amine **S31**

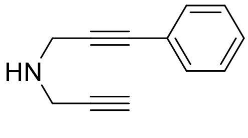

S31

Diyne **S30** (404.0 mg, 1.50 mmol, 1.0 eq.) was dissolved in anhydrous dichlormethane (3.0 mL). To this solution trifluoroacetic acid (0.21 mL, 2.7 mmol, 1.1 eq.) was added dropwise and the resulting mixture was stirred at room temperature for 2 hours. The reaction was quenched by addition of saturated K_2CO_3 solution and extracted with dichlormethane. The combined organic layers were washed with a saturated aqueous sodium chloride solution and dried over magnesium sulphate and the solvent was removed under reduced pressure. The

title compound was isolated after purification by silica gel column chromatography (isohexane/ethyl acetate – 1/3) in a yield of 243 mg (1.43 mmol, 96%) as a orange liquid.

^{1}H NMR (300 MHz, $CDCl_3$) δ = 7.48–7.37 (m, 2H), 7.34–7.27 (m, 3H), 3.75 (s, 2H), 3.59 (d, J = 2.5 Hz, 2H), 2.25 (t, J = 2.5 Hz, 1H) ppm; **^{13}C NMR** (75 MHz, $CDCl_3$) δ = 131.8, 128.4, 128.3, 123.2, 86.7, 84.0, 81.5, 72.0, 38.0, 37.2 ppm; **IR** (ATR) $\tilde{\nu}$ = 693 (m), 757 (s), 913 (w), 1103 (m), 1327 (w), 1353 (w), 1442 (w), 1491 (m), 3291 (m) cm^{-1}; **HRMS** (ESI): calcd. for $C_{12}H_{12}N^{+}$: 170.0964; found: 170.0983; $\boldsymbol{R}_f$ = 0.17 (isohexane/ethyl acetate – 1/2).

4.2.11 Preparation of 4-methyl-*N*-(2-methyl-4-phenylbut-3-yn-2-yl)-*N*-(prop-2-yn-1-yl)benzenesulfonamide **S35**

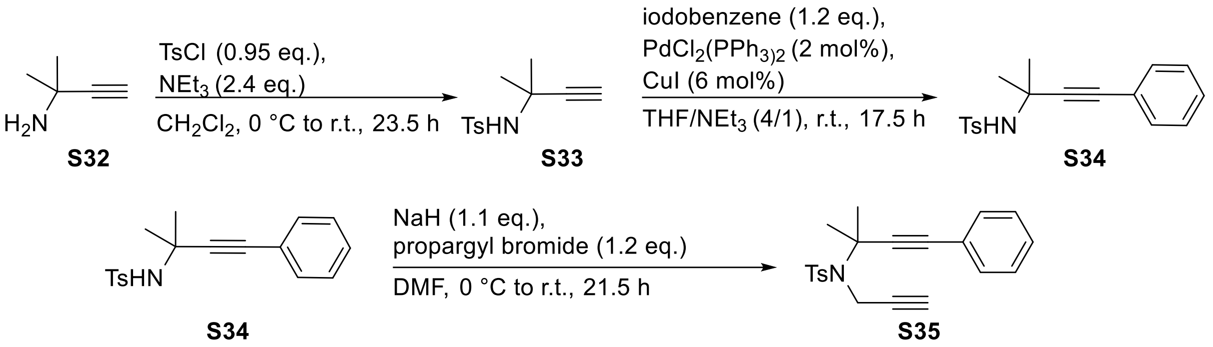

Preparation of 4-methyl-*N*-(2-methylbut-3-yn-2-yl)benzenesulfonamide **S33**

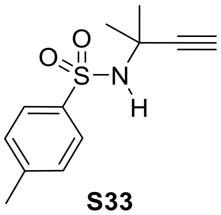

S33

The reaction was set up under air without anhydrous solvents. 1,1-Dimethylpropargylamine **S32** (1.1 mL, 10 mmol, 1.00 eq.) was dissolved in dichloromethane (24 mL) at 0 °C in a single-necked round-bottomed flask. Triethylamine (3.3 mL, 24 mmol, 2.4 eq.) and *p*-toluenesulphonyl chloride (1.81 g, 9.50 mmol, 0.95 eq.) were added at 0 °C, then the reaction mixture was warmed to room temperature and stirred for 23.5 hours. After dilution with diethyl ether, the mixture was washed with dilute hydrochloric acid (1 N), a saturated aqueous ammonium chloride solution and a saturated aqueous sodium chloride solution. The organic layer was dried over magnesium sulphate and the solvent was removed under reduced pressure. The title compound was isolated after purification by silica gel column chromatography (isohexane/ethyl acetate – 6/1 to 2/1) in a yield of 1.15 g (4.84 mmol, 51%, calcd. on *p*-TsCl) as a colourless solid.

^{1}H NMR (300 MHz, $CDCl_3$) δ = 7.88–7.64 (m, 2H), 7.29–7.22 (m, 2H), 4.78 (s, 1H), 2.42 (s, 3H), 2.10 (s, 1H), 1.55 (s, 6H) ppm; **^{13}C NMR** (75 MHz, $CDCl_3$) δ = 143.4, 139.0, 129.4, 127.8, 85.6, 71.3, 50.2, 30.8, 21.7 ppm; **IR** (ATR) $\tilde{\nu}$ = 813 (w), 861 (w), 939 (w), 999 (m), 1096 (m), 1148 (s) ,1219 (w), 1323 (m), 1383 (w), 1424 (w), 1599 (w), 2922 (w), 2986 (w), 3269 (m) cm^{-1}; **HRMS** (ESI): calcd. for $C_{12}H_{15}NO_2SNa^+$: 260.0816; found: 260.0818 ***R*f** = 0.27 (isohexane/ethyl acetate – 5/1).

The analytical data are in good accordance with the literature.[19]

Preparation of 4-methyl-*N*-(2-methyl-4-phenylbut-3-yn-2-yl)benzenesulfonamide **S34**

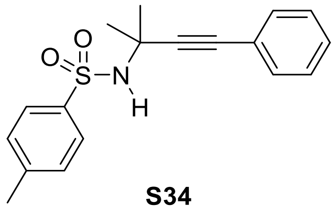

S34

A dried two-necked flask was charged with iodobenzene (0.13 mL, 1.2 mmol, 1.2 eq.), $PdCl_2(PPh_3)_2$ (14.0 mg, 19.9 µmol, 2 mol%) and copper(I)-iodide (11.4 mg, 599 µmol, 6 mol%). Anhydrous tetrahydrofurane (3.0 mL) and anhydrous triethylamine (1.0 mL) was added and the resulting slurry was stirred at room temperature for 5 minutes. Propargylamid **S33** (237 mg, 1.00 mmol, 1.0 eq.) was added and the reaction mixture was stirred for 17.5 hours. The raction mixture was filtered over Celite and dried over magnesium sulphate. The solvent was removed under reduced pressure. The title compound was isolated after purification by silica gel column chromatography (isohexane/ethyl acetate – 6/1) in a yield of 292 mg (931 µmol, 93%) as a colourless solid.

^{1}H NMR (300 MHz, $CDCl_3$) δ = 7.87–7.72 (m, 2H), 7.28–7.17 (m, 3H), 7.17–7.10 (m, 2H), 7.07–7.00 (m, 2H), 5.39 (s, 1H), 2.24 (s, 3H), 1.64 (s, 6H) ppm; **^{13}C NMR** (75 MHz, $CDCl_3$) δ = 143.1, 138.7, 131.7, 129.4, 128.2, 128.0, 127.7, 122.5, 90.6, 83.4, 50.6, 31.2, 21.4 ppm; **IR** (ATR) $\tilde{\nu}$ = 663 (s), 697 (m), 738 (m), 816 (m), 947 (m), 988 (s), 1018 (m), 1088 (s), 1144 (s), 1275 (m), 1383 (m), 1424 (m), 3273 (m) cm^{-1}; **HRMS** (ESI): calcd. for $C_{18}H_{19}NO_2SNa^+$: 336.1029; found: 336.1064; ***R*f** = 0.31 (isohexane/ethyl acetate – 5/1).

The analytical data are in good accordance with the literature.[20]

Preparation of 4-methyl-*N*-(2-methyl-4-phenylbut-3-yn-2-yl)-*N*-(prop-2-yn-1-yl)benzenesulfon-amide **S35**

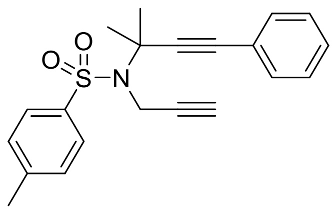

Propargylamid **S34** (313 mg, 1.00 mmol, 1.0 eq.) was dissolved in anhydrous dimethylformamide (5 mL). The solution was cooled down to 0 °C and NaH (60 % in mineral oil, 44.0 mg, 3.30 mmol, 1.1 eq.) was added. Stirring was continued for 10 minutes, subsequently propargyl bromide (80% in toluene, 0.13 mL, 1.2 mmol, 1.2 eq.) was added dropwise. The reaction mixture was warmed to room temperature and was stirred for 21.5 hours. The reaction was quenched by addition of water and extracted with diethyl ether. The combined organic layers were washed with a saturated aqueous sodium chloride solution and dried over magnesium sulphate and the solvent was removed under reduced pressure. The title compound was isolated after purification by silica gel column chromatography (isohexane/ethyl acetate – 15/1) in a yield of 310 mg (882 µmol, 88%) as a colourless liquid.

^{1}H NMR (400 MHz, $CDCl_3$) δ = 7.89–7.79 (m, 2H), 7.36–7.26 (m, 5H), 7.26–7.20 (m, 2H), 4.45 (d, J = 2.4 Hz, 2H), 2.37 (s, 3H), 2.34 (t, J = 2.4 Hz, 1H), 1.81 (s, 6H) ppm; **^{13}C NMR** (101 MHz, $CDCl_3$) δ = 143.2, 139.6, 131.7, 129.5, 128.5, 128.3, 127.5, 122.5, 91.2, 84.3, 81.4, 72.4, 57.4, 37.2, 30.5, 21.6 ppm; **IR** (ATR) $\tilde{\nu}$ = 727 (m), 757 (m), 813 (m), 880 (m), 921 (w), 947 (w), 1044 (m), 1088 (m), 1148 (s), 1196 (m), 1327 (m), 1442 (w), 1491 (w), 1595 (w), 2989 (w), 3288 (w) cm^{-1}; **HRMS** (ESI): calcd. for $C_{21}H_{21}NO_2SNa^+$: 374.1185; found: 374.1218; ***R*f** = 0.63 (isohexane/ethyl acetate – 5/1).

4.2.12 Preparation of 4-methyl-*N*-(pent-4-en-2-yn-1-yl)-*N*-(prop-2-yn-1-yl)benzenesulfonamide **S37**

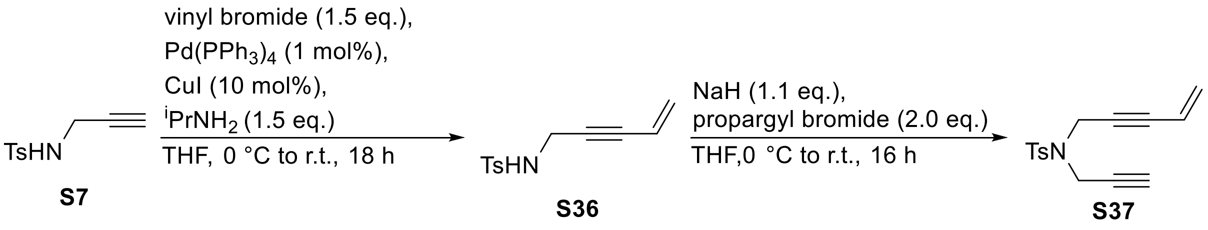

Preparation of 4-methyl-*N*-(pent-4-en-2-yn-1-yl)benzenesulfonamide **S36**

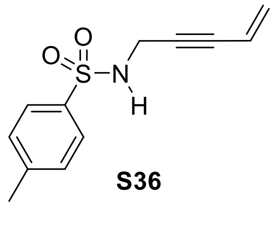

S36

The compound was prepared according to a literature-known procedure.[21] Tosylamide **S7** (1.05 g, 5.00 mmol, 1.0 eq.), $Pd(PPh_3)_4$ (57.8 mg, 50.0 µmol, 1 mol%) and copper(I)-iodide (95.0 mg, 499 µmol, 10 mol%) were dissolved in anhydrous tetrahydrofuran (5 mL) and the resulting solution was cooled to 0 °C. Anhydrous diisopropylamine (0.77 mL, 7.5 mmol, 1.5 eq.) and vinyl bromide (7.5 mL, 7.5 mmol, 1.5 eq., 1 M in THF) was added and the resulting slurry was stirred at room temperature for 18 hours. After filtration (celite), the filtrate was concentrated to dryness under reduced pressure. The title compound was isolated after purification by silica gel column chromatography (isohexane/ethyl acetate – 5/1) in a yield of 742 mg (3.15 mmol, 63%) as a brown solid.

^{1}H NMR (300 MHz, $CDCl_3$) δ = 7.83–7.72 (m, 2H), 7.39–7.27 (m, 2H), 5.65–5.48 (m, 1H), 5.46–5.33 (m, 2H), 4.61 (t, *J* = 5.6 Hz, 1H), 3.95 (dd, *J* = 6.1, 1.8 Hz, 2H), 2.42 (s, 3H) ppm; **^{13}C NMR** (75 MHz, $CDCl_3$) δ = 143.9, 136.9, 129.8, 127.8, 127.6, 116.3, 84.0, 83.5, 33.8, 21.7 ppm; **IR** (ATR) $\tilde{\nu}$ = 671 (m), 749 (w), 813 (s), 842 (m), 939 (m), 977 (w), 1021 (m) ,1062 (m), 1088 (m), 1152 (s) ,1237 (w), 1319 (s), 1431 (m), 1495 (w), 1595 (m), 2851 (w), 2922 (m), 2974 (w), 3269 (m) cm^{-1}; **HRMS** (ESI): calcd. for $C_{12}H_{13}NO_2SNa^+$: 258.0559; found: 258.0571; $\boldsymbol{R_f}$ = 0.24 (isohexane/ethyl acetate – 5/1).

The analytical data are in good accordance with the literature.[13]

Preparation of 4-methyl-*N*-(pent-4-en-2-yn-1-yl)-*N*-(prop-2-yn-1-yl)benzenesulfonamide **S37**

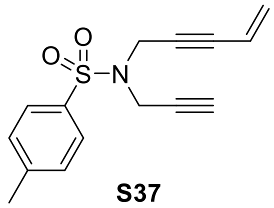

S37

NaH (60 % in mineral oil, 88.0 mg, 2.20 mmol, 1.1 eq.) was added to dry tetrahydrofurane (5 mL). The suspension was cooled down to 0 °C and tosylamide **S36** (471 mg, 2.00 mmol, 1.0 eq.) was added. Stirring was continued for 20 minutes, after that propargyl bromide (80% in toluene, 0.45 mL, 4.0 mmol, 2.0 eq.) was added dropwise. The reaction mixture was warmed to room temperature and was stirred for 16 hours. The reaction was quenched by addition of water, and extracted with diethyl ether. The combined organic layers were washed with a saturated aqueous sodium chloride solution and dried over magnesium sulphate and the solvent was removed under reduced pressure. The title compound was isolated after purification by silica gel column chromatography (isohexane/ethyl acetate – 10/1) in a yield of 456 mg (1.67 mmol, 83%) as a yellow liquid.

^{1}H NMR (300 MHz, $CDCl_3$) δ = 7.79–7.67 (m, 2H), 7.34–7.27 (m, 2H), 5.68–5.51 (m, 1H), 5.50–5.35 (m, 2H), 4.29 (d, J = 1.4 Hz, 2H), 4.14 (d, J = 2.5 Hz, 2H), 2.42 (s, 3H), 2.16 (t, J = 2.5 Hz, 1H) ppm; **^{13}C NMR** (75 MHz, $CDCl_3$) δ = 144.0, 135.4, 129.7, 128.1, 127.9, 116.4, 84.6, 82.2, 76.6, 74.1, 37.1, 36.5, 21.7 ppm; **IR** (ATR) $\tilde{\nu}$ = 745 (m), 816 (w), 895 (m), 928 (w), 973 (w), 1096 (m), 1122 (w), 1163 (s), 1349 (m), 1435 (w), 1599 (w), 3284 (w) cm^{-1}; **HRMS** (ESI): calcd. for $C_{15}H_{15}NO_2SNa^+$: 296.0716; found: 296.0725; $\boldsymbol{R_f}$ = 0.34 (isohexane/ethyl acetate – 10/1).

The analytical data are in good accordance with the literature.[22]

4.2.13 Preparation of *N*-(6-bromohex-2-yn-1-yl)-4-methyl-*N*-(prop-2-yn-1-yl)benzenesulfonamide **S39**

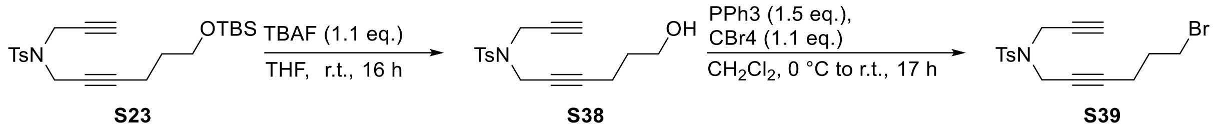

Preparation of *N*-(6-hydroxyhex-2-yn-1-yl)-4-methyl-*N*-(prop-2-yn-1-yl)benzenesulfonamide **S38**

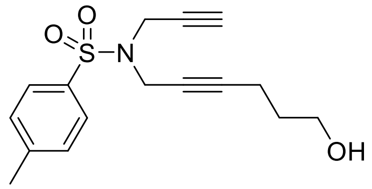

S38

Tosylamid **S23** (2.52 g, 6.00 mmol, 1.0 eq.) was dissolved in anhydrous tetrahydrofurane (5 mL) and TBAF (1 M in THF, 6.6 mL, 6.6 mmol, 1.1 eq.) was added dropwise. The reaction mixture was was stirred for 16.0 hours at rt. The reaction mixture was diluted with diethyl ether and washed with water. The aqueous layer was extracted with diethyl ether and the combined organic layers were washed with a saturated aqueous sodium chloride solution and dried over magnesium sulphate. The volatiles were removed under reduced pressure and the crude product was purified by silica gel column chromatography (isohexane/ethyl acetate – 1/1). The product was obtained as a yellow oil in a yield of 1.57 g (5.12 mmol, 85%).

^{1}H NMR (400 MHz, $CDCl_3$) δ = 7.75–7.66 (m, 2H), 7.33–7.27 (m, 2H), 4.14–4.11 (m, 4H), 3.62 (t, J = 6.2 Hz, 2H), 2.42 (s, 3H), 2.19–2.10 (m, 3H), 1.65–1.55 (m, 2H) ppm; **^{13}C NMR** (101 MHz, $CDCl_3$) δ = 143.9, 135.5, 129.6, 128.1, 85.8, 76.6, 73.9, 72.8, 61.6, 36.8, 36.3, 31.1, 21.7, 15.2 ppm; **IR** (ATR) $\tilde{\nu}$ = 704 (w), 753 (m), 816 (m), 895 (m), 928 (w), 954 (w), 1059 (m), 1092 (m), 1159 (s), 1252 (w), 1346 (m), 1435 (w), 1595 (w), 2922 (w), 3284 (w) cm^{-1}; **HRMS** (ESI): calcd. for $C_{16}H_{19}NO_3SNa^+$: 328.0978; found: 328.0984; $\boldsymbol{R_f}$ = 0.14 (isohexane/ethyl acetate – 2/1).

The analytical data are in good accordance with the literature.[23]

Preparation of *N*-(6-bromohex-2-yn-1-yl)-4-methyl-*N*-(prop-2-yn-1-yl)benzenesulfonamide **S39**

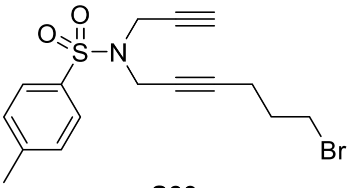

S39

Diyne alcohol **S38** (672 mg, 2.20 mmol, 1.0 eq.) was dissolved in anhydrous dichloromethane (5 mL). This solution was cooled to 0 °C, and triphenylphosphine (867 mg, 3.30 mmol, 1.5 eq.) and tetrabromomethane (803 mg, 2.42 mmol, 1.1 eq.) were added. The reaction mixture was warmed to room temperature and stirred for 17 hours. After filtration (cotton plug), the filtrate was concentrated to dryness under reduced pressure. The crude product was purified by silica gel column chromatography (petroleum ether/ethyl acetate – 10/1 to 5/1) to yield 576 mg (1.56 mmol, 71%) of the title compound as a yellow liquid.

^{1}H NMR (400 MHz, $CDCl_3$) δ = 7.75–7.69 (m, 2H), 7.33–7.28 (m, 2H), 4.19–4.07 (m, 4H), 3.36 (t, J = 6.5 Hz, 2H), 2.43 (s, 3H), 2.22 (tt, J = 6.8 Hz, 2.2 Hz, 2H), 2.15 (t, J = 2.5 Hz, 1H), 1.87 (quint, J = 6.6 Hz, 2H) ppm; **^{13}C NMR** (101 MHz, $CDCl_3$) δ = 144.0, 135.5, 129.7, 128.1, 84.5, 76.6, 73.9, 73.5, 36.8, 36.4, 32.3, 31.2, 21.7, 17.4 ppm; **IR** (ATR) $\tilde{\nu}$ = 749 (m), 813 (m), 854 (w), 895 (m), 954 (w), 1092 (m), 1159 (s), 1249 (m), 1349 (m), 1431 (w), 1495 (w), 1595 (w), 2922 (w), 3284 (w) cm^{-1}; **HRMS** (ESI): calcd. for $C_{16}H_{18}BrNO_2SNa^+$: 390.0134; found: 390.0134; $\boldsymbol{R_f}$ = 0.10 (isohexane/ethyl acetate – 10/1).

4.2.14 Preparation of (3-((5-phenylpent-2-yn-1-yl)oxy)prop-1-yn-1-yl)benzene **S41**

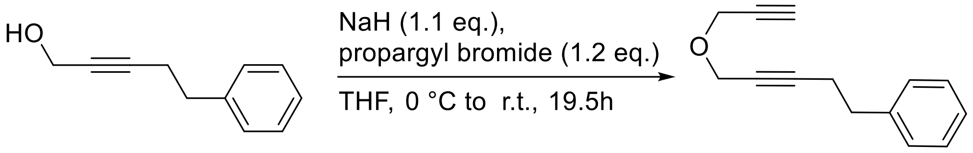

S18 **S40**

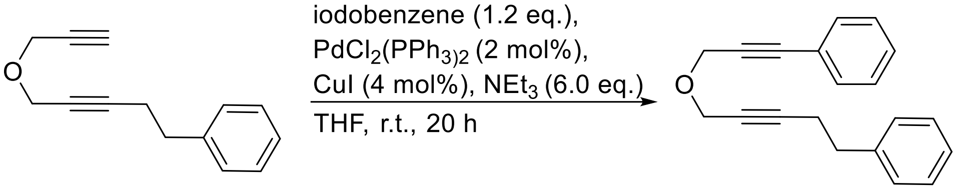

S40 **S41**

Preparation of (5-(prop-2-yn-1-yloxy)pent-3-yn-1-yl)benzene **S40**

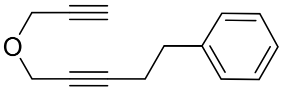

S40

Propargylalcohol **S18** (256 mg, 1.60 mmol, 1.0 eq.) was dissolved in anhydrous tetrahydrofurane (8 mL). The solution was cooled down to 0 °C and NaH (60 % in mineral oil, 70.4 mg, 1.76 mmol, 1.1 eq.) was added. Stirring was continued for 30 minutes, subsequently propargyl bromide (80% in toluene, 0.21 mL, 1.9 mmol, 1.2 eq.) was added dropwise. The reaction mixture was warmed to room temperature and was stirred for 19 hours. The reaction was quenched by addition of a saturated aqueous sodium hydrogencarbonate solution and extracted with diethyl ether. The combined organic layers were washed with a saturated aqueous sodium chloride solution and dried over magnesium sulphate and the solvent was removed under reduced pressure. The title compound was isolated after purification by silica gel column chromatography (isohexane/ethyl acetate – 30/1) in a yield of 299 mg (1.51 mmol, 94%) as a yellow liquid.

^{1}H NMR (300 MHz, $CDCl_3$) δ = 7.36–7.27 (m, 2H), 7.24–7.16 (m, 3H), 4.23 (t, J = 2.1 Hz, 2H), 4.18 (d, J = 2.4 Hz, 2H), 2.84 (t, J = 7.5 Hz, 2H), 2.53 (tt, J = 7.4, 2.1 Hz, 2H), 2.43 (t, J = 2.4 Hz, 1H) ppm; **^{13}C NMR** (75 MHz, $CDCl_3$) δ = 140.6, 128.6, 128.5, 126.5, 87.1, 79.3, 75.9, 74.8, 57.2, 56.3, 35.1, 21.1 ppm; **IR** (ATR) $\tilde{\nu}$ = 701 (m), 745 (m), 887 (w), 932 (w), 1003 (w), 1073 (s), 1129 (m), 1245 (w), 1342 (m), 1454 (w), 1495 (w), 2855 (w), 2907 (w), 3027 (w), 3288 (w) cm^{-1}; **HRMS** (ESI): calcd. for $C_{14}H_{14}ONa^+$: 221.0937; found: 221.0940; $\boldsymbol{R_f}$ = 0.40 (isohexane/ethyl acetate – 20/1).

Preparation of (3-((5-phenylpent-2-yn-1-yl)oxy)prop-1-yn-1-yl)benzene **S41**

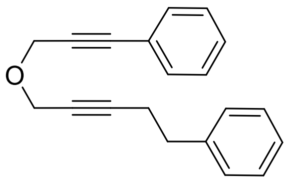

S41

A dried two-necked flask was charged with Diyne **S40** (198 mg, 1.00 mmol, 1.0 eq.), $PdCl_2(PPh_3)_2$ (14.0 mg, 20.0 µmol, 2 mol%) and copper(I)-iodide (7.6 mg, 0.04 mmol, 4 mol%). Anhydrous tetrahydrofurane (2.0 mL) and anhydrous triethylamine (0.83 mL, 6.0 mmol, 6.0 eq.) was added and the resulting slurry was stirred at room temperature for 5 minutes. The iodobenzene (0.13 mL, 1.2 mmol, 1.2 eq.) was added and the reaction mixture was stirred for 17.5 hours. The reaction was diluted with ethyl acetate and washed with dilute hydrochloric acid (1 N). The aqueous layer was extracted with ethyl acetate and the combined organic layers were washed with a saturated aqueous sodium chloride solution and dried over magnesium sulphate. The solvent was removed under reduced pressure. The title compound was isolated after purification by silica gel column chromatography (isohexane/ethyl acetate – 30/1) in a yield of 138 mg (500 µmol, 50%) as a yellow liquid.

^{1}H NMR (300 MHz, $CDCl_3$) δ = 7.50–7.39 (m, 2H), 7.35–7.26 (m, 5H), 7.24–7.19 (m, 3H), 4.41 (s, 2H), 4.29 (t, J = 2.1 Hz, 2H), 2.85 (t, J = 7.5 Hz, 2H), 2.54 (tt, J = 7.5, 2.0 Hz, 2H) ppm; **^{13}C NMR** (75 MHz, $CDCl_3$) δ = 140.7, 131.9, 128.6, 128.6, 128.6, 128.4, 126.5, 122.7, 87.0, 86.7, 84.7, 76.1, 57.3, 57.2, 35.1, 21.1 ppm; **IR** (ATR) $\tilde{v}$ = 693 (m), 757 (s), 891 (w), 917 (w), 1029 (w), 1073 (s), 1129 (w), 1260 (w), 1349 (m), 1442 (w), 1491 (m), 1599 (w), 2851 (w), 2926 (w), 3027 (w), 3064 (w) cm^{-1}; **HRMS** (ESI): calcd. for $C_{20}H_{18}NONa^+$: 297.1250; found: 297.1252; ***R*$_f$** = 0.43 (isohexane/ethyl acetate – 20/1).

4.2.15 Preparation of 4-methyl-*N*-(5-phenylpent-2-yn-1-yl)-*N*-(3-phenylprop-2-yn-1-yl)benzenesulfonamide **S42**

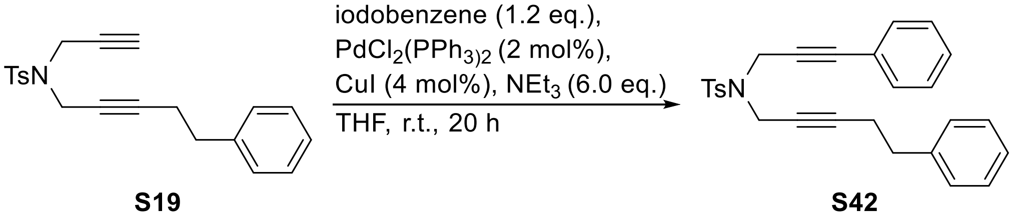

S19 **S42**

Preparation of 4-methyl-*N*-(5-phenylpent-2-yn-1-yl)-*N*-(3-phenylprop-2-yn-1-yl)benzenesulfonamide **S42**

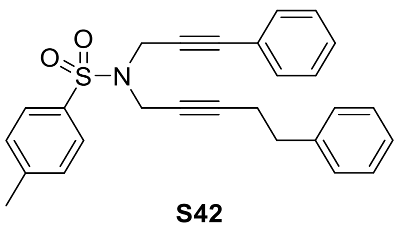

S42

A dried two-necked flask was charged with tosylamide **S19** (527 mg, 1.50 mmol, 1.0 eq.), $PdCl_2(PPh_3)_2$ (21.1 mg, 30.1 µmol, 2 mol%) and copper(I)-iodide (11.4 mg, 58.6 µmol, 4 mol%). Anhydrous tetrahydrofurane (2.5 mL) and anhydrous triethylamine (1.3 mL, 9.4 mmol, 6.3 eq.) was added and the resulting slurry was stirred at room temperature for 5 minutes. The iodobenzene (0.20 mL, 1.8 mmol, 1.2 eq.) was added and the reaction mixture was stirred for 20 hours. The reaction was diluted with ethyl acetate and washed with dilute hydrochloric acid (1 N). The aqueous layer was extracted with ethyl acetate and the combined organic layers were washed with a saturated aqueous sodium chloride solution and dried over magnesium sulphate. The solvent was removed under reduced pressure. The title compound was isolated after purification by silica gel column chromatography (isohexane/ethyl acetate – 10/1) in a yield of 437 mg (1.02 mmol, 68%) as a orange oil.

^{1}H NMR (300 MHz, $CDCl_3$) δ = 7.90–7.51 (m, 2H), 7.37–6.94 (m, 12H), 4.27 (s, 2H), 4.15 (t, J = 2.1 Hz, 2H), 2.69 (t, J = 7.4 Hz, 2H), 2.41–2.29 (m, 5H) ppm; **^{13}C NMR** (75 MHz, $CDCl_3$) δ = 143.8, 140.5, 135.6, 131.8, 129.6, 128.6, 128.5, 128.5, 128.3, 128.1, 126.5, 122.5, 85.8, 85.7, 81.9, 73.4, 37.1, 34.8, 21.6, 20.9 ppm (due to coincidental chemical equivalence of two carbon resonances one signal is missing); **IR** (ATR) $\tilde{\nu}$ = 693 (s), 753 (s), 813 (m), 898 (s), 947 (w), 1092 (s), 1159 (s), 1252 (w), 1349 (s), 1401 (w), 1439 (m), 1491 (m), 1595 (w), 2848 (w), 2922 (w), 3027 (w), 3060 (w) cm^{-1};**HRMS** (ESI): calcd. for $C_{27}H_{25}NO_2SNa^+$: 450.1498; found: 450.1505; $\boldsymbol{R}_f$ = 0.20 (isohexane/ethyl acetate – 10/1).

4.2.16 Preparation of phenylethynylbenzene derivatives **S43** – **S48**

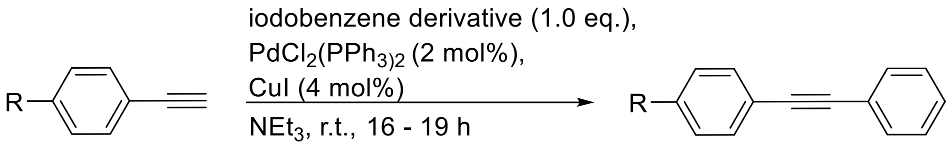

Preparation of 1-methoxy-4-(phenylethynyl)benzene **S43**

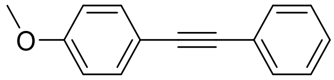

S43

A dried two-necked flask was charged with 4-iodoanisole (2.34 g, 10.0 mmol, 1.0 eq.), $PdCl_2(PPh_3)_2$ (140 mg, 200 µmol, 2 mol%) and copper(I)-iodide (76.2 mg, 400 µmol, 4 mol%). Anhydrous triethylamine (30 mL) was added and the resulting slurry was stirred at room temperature for 5 minutes. The phenylacetylene (1.3 mL, 12 mmol, 1.2 eq.) was added and the reaction mixture was stirred for 16 hours. After filtration (celite), the filtrate was diluted with ethyl acetate and washed with dilute hydrochloric acid (1 N). The aqueous layer was extracted with ethyl acetate and the combined organic layers were washed with a saturated aqueous sodium chloride solution and dried over magnesium sulphate. The solvent was removed under reduced pressure. The title compound was isolated after purification by silica gel column chromatography (isohexane/ethyl acetate – 1/0) in a yield of 1.98 g (9.51 mmol, 95%) as a yellow solid.

^{1}H NMR (300 MHz, $CDCl_3$) δ = 7.57–7.43 (m, 4H), 7.38–7.28 (m, 3H), 6.91–6.83 (m, 2H), 3.83 (s, 3H) ppm; **^{13}C NMR** (75 MHz, $CDCl_3$) δ = 159.8, 133.2, 131.6, 128.5, 128.1, 123.8, 115.5, 114.1, 89.5, 88.2, 55.5 ppm; **IR** (ATR) $\tilde{\nu}$ = 690 (s), 779 (m), 827 (s), 913 (w), 1029 (s), 1070 (w), 1103 (m), 1141 (m), 1174 (m), 1245 (s), 1282 (m), 1461 (m), 1506 (s), 1595 (m), 2837 (w), 2956 (w), 3001 (w), 3053 (w) cm^{-1}; **MS** (EI): *m*/*z* (%): 208 (100), 193 (62), 165 (55), 139 (15), 115 (7), 104 (5), 87 (4); $\boldsymbol{R_f}$ = 0.09 (isohexane/ethyl acetate – 1/0).

The analytical data are in good accordance with the literature.[24]

Preparation of 1-fluoro-4-(phenylethynyl)benzene **S44**

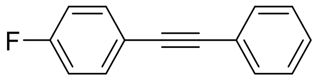

S44

A dried two-necked flask was charged with 1-fluoro-4-iodobenzene (2.00 g, 9.00 mmol, 1.0 eq.), $PdCl_2(PPh_3)_2$ (126 mg, 180 µmol, 2 mol%) and copper(I)-iodide (68.6 mg, 360 µmol, 4 mol%). Anhydrous triethylamine (27 mL) was added and the resulting slurry was stirred at room temperature for 5 minutes. The phenylacetylene (0.99 mL, 9.0 mmol, 1.0 eq.) was added and the reaction mixture was stirred for 19 hours. After filtration (celite), the filtrate was diluted with ethyl acetate and washed with dilute hydrochloric acid (1 N). The aqueous layer was extracted with ethyl acetate and the combined organic layers were washed with a saturated

aqueous sodium chloride solution and dried over magnesium sulphate. The solvent was removed under reduced pressure. The title compound was isolated after purification by silica gel column chromatography (isohexane/ethyl acetate – 1/0) in a yield of 1.65 g (8.41 mmol, 93%) as a colourless solid.

^{1}H NMR (400 MHz, $CDCl_3$) δ = 7.59–7.45 (m, 4H), 7.42–7.31 (m, 3H), 7.15–7.01 (m, 2H) ppm; **^{13}C NMR** (101 MHz, $CDCl_3$) δ = 162.8 (d, J = 249.6 Hz), 133.6 (d, J = 8.4 Hz), 131.7, 128.5, 128.5, 123.2, 119.5 (d, J = 3.5 Hz), 115.8 (d, J = 22.2 Hz), 89.2 (d, J = 1.8 Hz), 88.4 ppm; **IR** (ATR) $\tilde{\nu}$ = 753 (s), 794 (m), 831 (m), 839 (s), 1155 (m), 1223 (s), 1442 (w), 1506 (s), 1595 (m), 2922 (w), 3049 (w) cm^{-1}; **MS** (EI): *m*/*z* (%): 196 (100), 175 (8), 170 (11), 157 (3), 144 (5), 120 (1), 98 (7), 85 (4), 74 (2); $\boldsymbol{R_f}$ = 0.51 (isohexane/ethyl acetate – 1/0).

The analytical data are in good accordance with the literature.[39]

Preparation of 1-chloro-4-(phenylethynyl)benzene **S45**

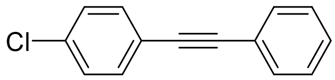

S45

A dried two-necked flask was charged with 1-chloro-4-iodobenzene (2.39 g, 10.0 mmol, 1.0 eq.), $PdCl_2(PPh_3)_2$ (140 mg, 200 µmol, 2 mol%) and copper(I)-iodide (76.2 mg, 400 µmol, 4 mol%). Anhydrous triethylamine (30 mL) was added and the resulting slurry was stirred at room temperature for 5 minutes. The phenylacetylene (1.1 mL, 10 mmol, 1.0 eq.) was added and the reaction mixture was stirred for 17 hours. After filtration (celite), the filtrate was diluted with ethyl acetate and washed with dilute hydrochloric acid (1 N) The aqueous layer was extracted with ethyl acetate and the combined organic layers were washed with a saturated aqueous sodium chloride solution and dried over magnesium sulphate. The solvent was removed under reduced pressure. The title compound was isolated after purification by silica gel column chromatography (isohexane/ethyl acetate – 1/0) in a yield of 1.69 g (7.94 mmol, 79%) as a colourless solid.

^{1}H NMR (300 MHz, $CDCl_3$) δ = 7.58–7.50 (m, 2H), 7.48–7.43 (m, 2H), 7.40–7.29 (m, 5H) ppm; **^{13}C NMR** (75 MHz, $CDCl_3$) δ = 134.4, 133.0, 131.8, 128.8, 128.6, 128.5, 123.1, 121.9, 90.5, 88.4 ppm; **IR** (ATR) $\tilde{\nu}$ = 690 (m) ,753 (m), 831 (s), 917 (w), 1018 (w), 1092 (m), 1394 (w), 1442 (w), 1491 (m) cm^{-1}; **MS** (EI): *m*/*z* (%): 212 (100), 176 (42), 151(13), 137 (2), 126 (4), 106 (6), 98 (3), 88 (6), 75 (5); $\boldsymbol{R_f}$ = 0.63 (isohexane/ethyl acetate – 1/0).

The analytical data are in good accordance with the literature.[24]

Preparation of 1-methyl-4-(phenylethynyl)benzene **S46**

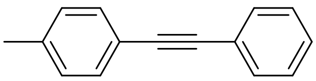

S46

A dried two-necked flask was charged with iodobenzene (1.1 mL, 10 mmol, 1.0 eq.), $PdCl_2(PPh_3)_2$ (140 mg, 200 µmol, 2 mol%) and copper(I)-iodide (76.2 mg, 400 µmol, 4 mol%). Anhydrous triethylamine (30 mL) was added and the resulting slurry was stirred at room temperature for 5 minutes. The 4-methylphenylacetylene (1.16 g, 10.0 mmol, 1.0 eq.) was added and the reaction mixture was stirred for 18.5 hours. After filtration (celite), the filtrate was diluted with ethyl acetate and washed with dilute hydrochloric acid (1 N). The aqueous layer was extracted with ethyl acetate and the combined organic layers were washed with a saturated aqueous sodium chloride solution and dried over magnesium sulphate. The solvent was removed under reduced pressure. The title compound was isolated after purification by silica gel

column chromatography (isohexane/ethyl acetate – 1/0) in a yield of 1.19 g (6.21 mmol, 62%) as a colourless solid.

^{1}H NMR (400 MHz, $CDCl_3$) δ = 7.56–7.48 (m, 2H), 7.46–7.38 (m, 2H), 7.37–7.28 (m, 3H), 7.19–7.11 (m, 2H), 2.37 (s, 3H) ppm; **^{13}C NMR** (101 MHz, $CDCl_3$) δ = 138.5, 131.7, 131.6, 129.3, 128.5, 128.2, 123.6, 120.3, 89.7, 88.9, 21.7 ppm; **IR** (ATR) $\tilde{\nu}$ = 690 (s), 757 (s), 820 (s), 1510 (m), 2855 (m), 2922 (m), 3053 (m) cm^{-1}; **MS** (EI): *m/z* (%): 192 (100), 176 (3), 165 (15), 152 (2), 139 (4), 126 (2), 115 (5), 95 (4), 82 (3); ***R*$_f$** = 0.34 (isohexane/ethyl acetate – 1/0).

The analytical data are in good accordance with the literature.[26]

Preparation of 1-(phenylethynyl)-4-(trifluoromethyl)benzene **S47**

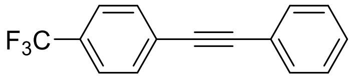

S47

A dried two-necked flask was charged with iodobenzene (1.1 mL, 10 mmol, 1.0 eq.), $PdCl_2(PPh_3)_2$ (140 mg, 200 µmol, 2 mol%) and copper(I)-iodide (76.2 mg, 400 µmol, 4 mol%). Anhydrous triethylamine (30 mL) was added and the resulting slurry was stirred at room temperature for 5 minutes. The 4-(trifluoromethyl)phenylacetylene (1.70 g, 10.0 mmol, 1.0 eq.) was added and the reaction mixture was stirred for 18.5 hours. After filtration (celite), the filtrate was diluted with ethyl acetate and washed with dilute hydrochloric acid (1 N). The aqueous layer was extracted with ethyl acetate and the combined organic layers were washed with with a saturated aqueous sodium chloride solution and dried over magnesium sulphate. The solvent was removed under reduced pressure. The title compound was isolated after purification by silica gel column chromatography (isohexane/ethyl acetate – 1/0) in a yield of 2.25 g (9.13 mmol, 91%) as a pale yellow solid.

^{1}H NMR (400 MHz, $CDCl_3$) δ = 7.66–7.59 (m, 4H), 7.58–7.52 (m, 2H), 7.42–7.33 (m, 3H) ppm; **^{13}C NMR** (101 MHz, $CDCl_3$) δ = 132.0, 131.9, 130.1 (q, *J* = 32.7 Hz), 129.0, 128.6, 127.3 (q, *J* = 1.6 Hz), 125.4 (q, *J* = 3.9 Hz), 124.1 (q, *J* = 271.2 Hz), 122.7, 91.9, 88.1 ppm; **IR** (ATR) $\tilde{\nu}$ = 690 (m), 760 (m), 842 (m), 1021 (w), 1066 (m), 1107 (s), 1129 (m), 1167 (m), 1331 (m), 1614 (w) cm^{-1}; **MS** (EI): *m/z* 246 (100), 227 (23), 207 (5), 196 (16), 176 (20), 151 (7), 123 (6), 98 (12), 85 (3), 75 (3); ***R*$_f$** = 0.49 (isohexane/ethyl acetate – 1/0).

Preparation of methyl 4-(phenylethynyl)benzoate **S48**

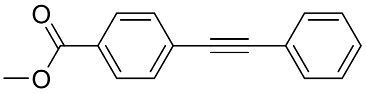

S48

A dried two-necked flask was charged with iodobenzene (1.1 mL, 10 mmol, 1.0 eq.), $PdCl_2(PPh_3)_2$ (140 mg, 200 µmol, 2 mol%) and copper(I)-iodide (76.2 mg, 400 µmol, 4 mol%). Anhydrous triethylamine (30 mL) was added and the resulting slurry was stirred at room temperature for 5 minutes. Methyl 4-ethynylbenzoate (1.60 g, 10.0 mmol, 1.0 eq.) was added and the reaction mixture was stirred for 19.5 hours. After filtration (celite), the filtrate was diluted with ethyl acetate and washed with dilute hydrochloric acid (1 N). The aqueous layer was extracted with ethyl acetate and the combined organic layers were washed with a saturated aqueous sodium chloride solution and dried over magnesium sulphate. The solvent was removed under reduced pressure. The title compound was isolated after purification by silica gel column chromatography (isohexane/ethyl acetate – 50/1 to 30/1) in a yield of 2.18 g (9.23 mmol, 92%) as a colourless solid.

^{1}H NMR (400 MHz, $CDCl_3$) δ = 8.07–8.00 (m, 2H), 7.63–7.57 (m, 2H), 7.57–7.50 (m, 2H), 7.41–7.33 (m, 3H), 3.93 (s, 3H) ppm; **^{13}C NMR** (101 MHz, $CDCl_3$) δ = 166.7, 131.9, 131.6, 129.7, 129.6, 128.9, 128.6, 128.1, 122.8, 92.5, 88.8, 52.4 ppm; **IR** (ATR) $\tilde{\nu}$ = 693 (s), 731 (m), 760 (s), 816 (m), 857 (m), 909 (m), 958 (m), 1103 (s), 1174 (m), 1275 (s), 1405 (m), 1435 (m), 1606 (m), 1707(s) cm^{-1}; **MS** (EI): *m*/*z* 236 (100), 205 (97), 176 (68), 151 (28), 126 (7), 103 (11), 88 (20), 75 (6), 63 (2); ***R*$_f$** = 0.10 (isohexane/ethyl acetate – 1/0).

The analytical data are in good accordance with the literature.[27]

4.2.17 Preparation of *N*-ethynyl-4-methyl-*N*-(2-(phenylethynyl)phenyl)benzenesulfonamide **S52**

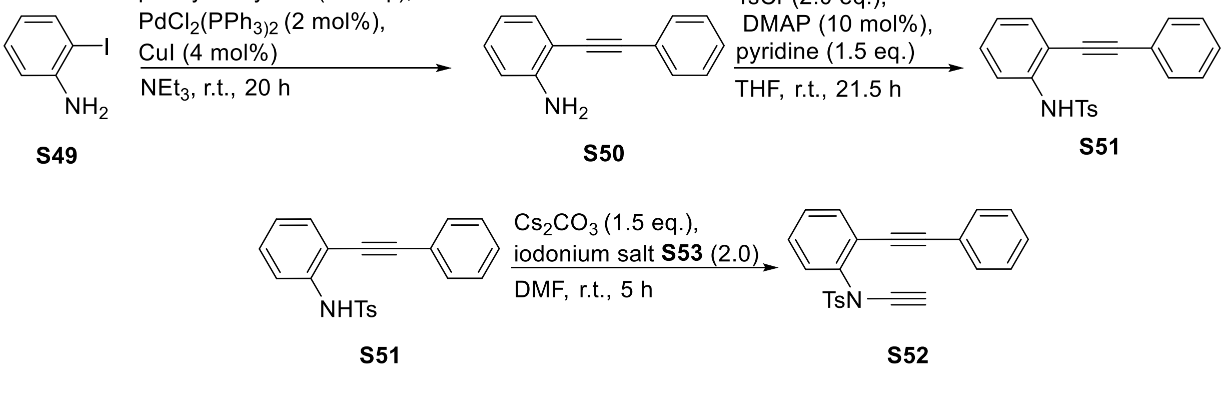

Preparation of methyl 4-(phenylethynyl)benzoate trifluoromethanesulfonate **S53**

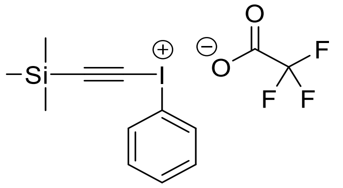

S53

The compound was prepared according to a literature-known procedure.[28] Phenyliodonium diacetate (6.44 g, 20.0 mmol, 1.0 equiv) was diluted with dichlormethane (20 mL) and the mixture was colled to 0 °C. Tf_2O (1.7 mL, 10 mmol, 0.50 eq.) was added dropwise at 0 °C and the resulting mixture was stirred 1 hour. Bis(trimethylsilyl)acetylene (4.5 mL, 20 mmol, 1.0 eq.) was added. The mixture was then stirred 3 hours and diethyl ether was added to precipitate the product. Filtration afforded the product **S53** (5.30 g, 11.8 mmol, 59%) as a colourless solid.

^{1}H NMR (300 MHz, $CDCl_3$) δ = 8.13–7.98 (m, 2H), 7.70–7.60 (m, 1H), 7.59–7.45 (m, 2H), 0.24 (s, 9H) ppm; **^{13}C NMR** (75 MHz, $CDCl_3$) δ = 134.1, 132.8, 132.7, 120.0 (d, J = 319.5 Hz), 120.0, 117.0, 44.2, –0.7 ppm; **IR** (ATR) $\tilde{\nu}$ = 675 (m), 708 (s), 760 (m), 842 (s), 988 (m), 1021 (s), 1155 (s), 1223 (s), 1282 (m), 3388 (w) cm^{-1}; **HRMS** (ESI): calcd. for $C_{11}H_{14}ISi^+$: 300.9904; found: 300.9900.

The analytical data are in good accordance with the literature.[28]

Preparation of 2-(phenylethynyl)aniline **S50**

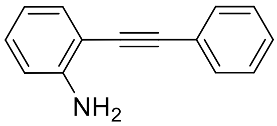

S50

A dried two-necked flask was charged with 2-iodoaniline **S49** (1.10 g, 5.00 mmol, 1.0 eq.), $PdCl_2(PPh_3)_2$ (70.2 mg, 100 µmol, 2 mol%) and copper(I)-iodide (38.1 mg, 200 µmol, 4 mol%). Anhydrous triethylamine (15 mL) was added and the resulting slurry was stirred at room temperature for 5 minutes. The phenylacetylene (0.55 mL, 5.0 mmol, 1.0 eq.) was added and the reaction mixture was stirred for 20 hours. After filtration (celite), the filtrate was diluted with ethyl acetate and washed with dilute hydrochloric acid (1 N). The aqueous layer was extracted with ethyl acetate and the combined organic layers were washed with a saturated aqueous sodium chloride solution and dried over magnesium sulphate. The solvent was removed under

reduced pressure. The title compound was isolated after purification by silica gel column chromatography (isohexane/ethyl acetate – 5/1) in a yield of 753 mg (3.90 mmol, 78%) as a orange solid.

^{1}H NMR (400 MHz, $CDCl_3$) *δ* = 7.58–7.47 (m, 2H), 7.41–7.29 (m, 4H), 7.20–7.08 (m, 1H), 6.79–6.67 (m, 2H), 4.28 (s, 2H) ppm; **^{13}C NMR** (101 MHz, $CDCl_3$) *δ* = 147.9, 132.3, 131.6, 129.9, 128.5, 128.4, 123.4, 118.1, 114.5, 108.1, 94.8, 86.0 ppm; **IR** (ATR) $\tilde{\nu}$ = 690 (s), 749 (s), 1155 (w), 1264 (m), 1312 (m), 1454 (m), 1483 (m), 1495 (m), 1565 (m), 1610 (s), 3030 (w), 3056 (w), 3370 (m), 3466 (m) cm^{-1}; **HRMS** (ESI): calcd. for $C_{14}H_{12}N^{+}$: 194.0964; found: 194.0972; ***R*f** = 0.51 (isohexane/ethyl acetate – 1/0).

The analytical data are in good accordance with the literature.[24]

Preparation of 4-methyl-*N*-(2-(phenylethynyl)phenyl)benzenesulfonamide **S51**

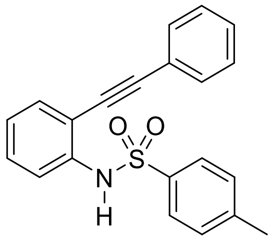

S51

The compound was prepared according to a literature-known procedure.[43] The 2-(2’-phenylethynyl)aniline **S50** (580 mg, 3.00 mmol, 1.0 eq.), pyridine (3.5 mL, 4.5 mmol, 1.5 eq.) and catalytic amount of DMAP (37.0 mg, 30.3 µmol, 10 mol%) was dissolved in tetrahydrofurane (20 mL), and 4-toluenesulfonyl chloride (1.14 g, 6.00 mmol, 2.0 eq.) dissolved in tetrahydrofurane (10 mL) was added dropwise with stirring into reaction. The mixture was stirred for 21.5 hours at room temperature. Then the reaction system was acidified with dilute hydrochloric acid (2 N). The mixture was extracted with ethyl acetate and washed with a saturated aqueous sodium chloride solution. The organic layer was dried over magnesium sulphate and concentrated under vacuum. The title compound was isolated after purification by silica gel column chromatography (isohexane/ethyl acetate – 10/1) in a yield of 554 mg (1.59 mmol, 53%) as a colourless solid.

^{1}H NMR (300 MHz, $CDCl_3$) *δ* = 7.72–7.66 (m, 2H), 7.64 (dd, *J* = 8.2, 0.7 Hz, 1H), 7.50–7.43 (m, 2H), 7.43–7.34 (m, 4H), 7.33–7.23 (m, 2H), 7.16 (d, *J* = 8.0 Hz, 2H), 7.06 (td, *J* = 7.6, 1.1 Hz, 1H), 2.32 (s, 3H) ppm; **^{13}C NMR** (75 MHz, $CDCl_3$) *δ* = 144.1, 137.6, 136.1, 132.1, 131.6,

129.7, 129.1, 128.6, 127.3, 124.7, 122.1, 120.5, 114.8, 96.2, 83.8, 21.6 ppm (due to coincidental chemical equivalence of two carbon resonances one signal is missing); **IR** (ATR) $\tilde{\nu}$ = 663 (s), 727 (s), 753 (s), 813 (m) ,842 (m), 906 (s), 1040 (w), 1088 (m), 1159 (s), 1215 (w), 1275 (m), 1334 (m), 1398 (m), 1454 (w), 1495 (m), 1599 (w), 3060 (w), 3265 (w), 3317 (w) cm^{-1};**MS** (EI): *m*/*z* (%): 347 (73), 183 (6), 267 (5), 208 (5), 192 (52), 165 (100), 139 (10), 115 (5), 91 (18), 89 (6), 77 (2), 65 (8); $\boldsymbol{R}_f$ = 0.20 (isohexane/ethyl acetate – 10/1).

The analytical data are in good accordance with the literature.[29]

Preparation of *N*-ethynyl-4-methyl-*N*-(2-(phenylethynyl)phenyl)benzenesulfonamide **S52**

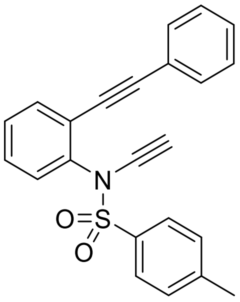

S52

The compound was prepared according to a literature-known procedure.[30] To a stirred solution of *N*-tosylaniline **S51** (452 mg, 1.30 mmol, 1.0 eq.) in anhydrous dimethylformamide (27 mL) was added Cs_2CO_3 (635 mg, 1.95 mmol, 1.5 eq.) at room temperature. After 10 minutes, a solution of iodonium salt **S53** (1.08 g, 2.60 mmol, 2.0 eq.) in anhydrous dichlormethane (11 mL) was added dropwise. Stirring was continued 5 hours. Then, diethyl ether was added and the combined organic layers were washed with water and with a saturated aqueous sodium chloride solution, dried over anhydrous magnesium sulphate and concentrated to dryness. The title compound was isolated after purification by silica gel column chromatography (isohexane/ethyl acetate – 10/1) in a yield of 426 mg (1.15 mmol, 88%) as a orange oil.

^{1}H NMR (400 MHz, $CDCl_3$) δ = 7.72–7.68 (m, 2H), 7.53–7.49 (m, 1H), 7.45–7.40 (m, 1H), 7.40–7.28 (m, 7H), 7.12–7.06 (m, 2H), 2.93 (s, 1H), 2.19 (s, 3H) ppm; **^{13}C NMR** (101 MHz, $CDCl_3$) δ = 144.1, 137.4, 133.6, 132.5, 130.7, 129.1, 128.8, 128.3, 128.2, 127.7, 127.5, 127.2, 122.0, 121.9, 94.6, 83.9, 75.0, 58.3, 20.6 ppm; **IR** (ATR) $\tilde{\nu}$ = 671 (s), 757 (s), 813 (m), 872 (w), 917 (m), 1021 (m), 1088 (m), 1118 (m), 1170 (s), 1260 (w), 1372 (m), 1446 (m), 1495 (m), 1536 (w), 1670 (w), 2132 (w), 3060 (w), 3295 (w) cm^{-1}; **HRMS** (ESI): calcd. for $C_{23}H_{17}NO_2SNa^+$: 394.0872; found: 394.0872 $\boldsymbol{R}_f$ = 0.20 (isohexane/ethyl acetate – 10/1).

The analytical data are in good accordance with the literature.[30]

4.2.18 Preparation of 1,8-bis(phenylethynyl)naphthalene **S54**

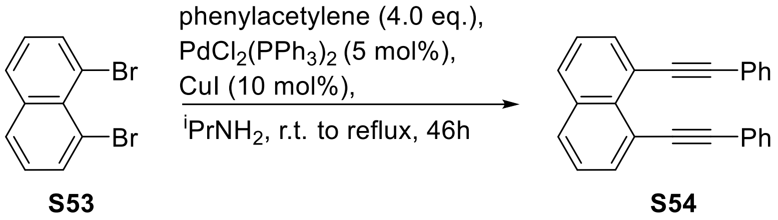

Preparation of 1,8-bis(phenylethynyl)naphthalene **S54**

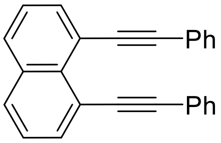

S54

The compound was prepared according to a literature-known procedure.[31] A dried two-necked flask was charged with 1,8-dibromonaphthalene **S53** (572 mg, 2.00 mmol, 1.0 eq.), $PdCl_2(PPh_3)_2$ (70.2 mg, 100 µmol, 5 mol%) and copper(I)-iodide (38.1 mg, 200 µmol, 10 mol%). Anhydrous diisopropylamine (10 mL, degassed) was added and the resulting slurry was stirred at room temperature for 5 minutes. Phenylacetylene (0.88 mL, 8.0 mmol, 4.0 eq.) was added and the reaction mixture was refluxed for 46 hours. After filtration (celite), the solvent was removed under reduced pressure. The title compound was isolated after purification by silica gel column chromatography (isohexane/ethyl acetate – 1/0) in a yield of 396 mg (1.21 mmol, 60%) as brown solid.

^{1}H NMR (400 MHz, $CDCl_3$) δ = 7.87 (dd, J = 7.2, 1.3 Hz, 2H), 7.84 (dd, J = 8.4, 1.2 Hz, 2H), 7.47 (dd, J = 8.2, 7.2 Hz, 2H), 7.38–7.32 (m, 4H), 7.23–7.16 (m, 2H), 7.15–7.06 (m, 4H) ppm; **^{13}C NMR** (101 MHz, $CDCl_3$) δ = 135.1, 134.3, 131.7, 131.6, 129.8, 128.1, 128.0, 125.7, 123.9, 121.0, 96.8, 89.9 ppm; **IR** (ATR) $\tilde{\nu}$ = 690 (m), 753 (s), 827 (m), 1245 (w), 1375 (m), 1491 (m), 1733 (m), 2851 (m), 2922 (s) cm^{-1}; **HRMS** (ESI): calcd. for $C_{26}H_{17}^{+}$: 329.1325; found: 329.1320; $\boldsymbol{R_f}$ = 0.09 (isohexane/ethyl acetate – 1/0).

The analytical data are in good accordance with the literature.[31]

4.2.19 Preparation of 3,4,5,6,11,12,13,14-octaphenyldiindeno[1,2,3-cd:1',2',3'-lm]perylene **59**

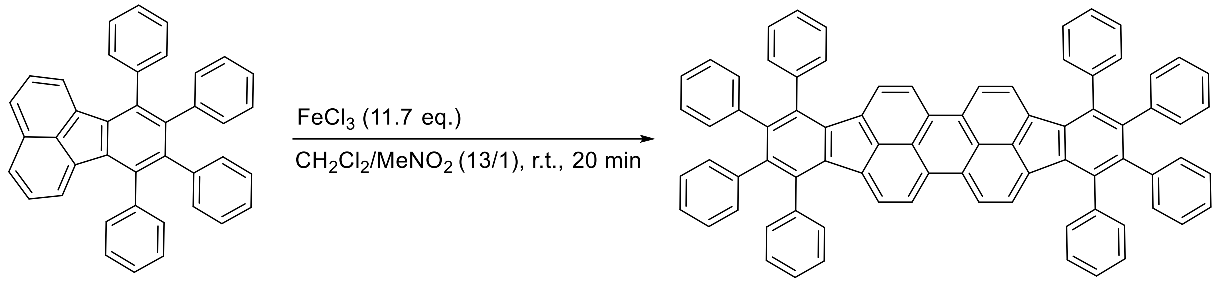

50 **59**

Preparation of 3,4,5,6,11,12,13,14-octaphenyldiindeno[1,2,3-cd:1',2',3'-lm]perylene **59**

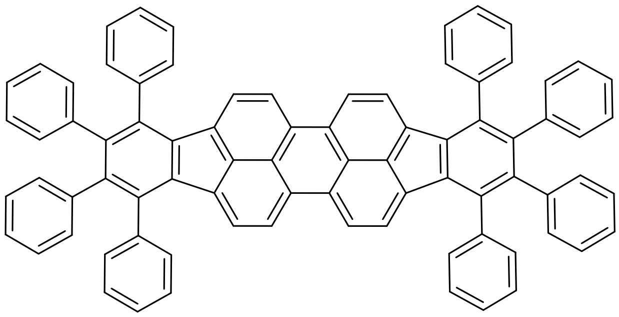

59

The compound was prepared according to a literature-known procedure.[32] A solution of $FeCl_3$ (380 mg, 2.34 mmol, 11.7 eq.) in nitromethane (0.60 mL) was added dropwise to a stirred solution of 7,8,9,10-tetraphenylfluoranthene **50** (101 mg, 200 µmol, 1.0 eq.) in anhydrous dichlormethane (8 mL, degassed). An argon stream was bubbled through the reaction mixture throughout the entire reaction. After stirring for another 20 minutes, the reaction was quenched with methanol. The precipitate was filtered, washed with methanol, and dried under reduced pressure (product). The remaining product in the filtrate was purified by columnchromatography on silica gel with (isohexane/dichloromethane 3/1 to 1/1) to afford the product **59** in a yield of 47.3 mg (46.9 µmol, 47%) as a red solid.

^{1}H NMR (600 MHz, CD_2Cl_2) δ = 7.82 (d, J = 7.8 Hz, 4H), 7.39–7.27 (m, 20H), 7.09–6.81 (m, 20H), 6.46 (d, J = 7.8 Hz, 4H) ppm; **^{13}C NMR** (151 MHz, CD_2Cl_2) δ = 141.1, 140.3, 140.1, 137.7, 136.9, 136.8, 134.7, 131.6, 130.7, 130.3, 128.5, 127.3, 126.9, 125.8, 125.3, 124.4, 122.2 ppm; **IR** (ATR) $\tilde{\nu}$ = 693 (s), 727 (m), 764 (m), 831 (m), 1029 (m), 1070 (m), 1118 (m), 1178 (m), 1316 (m), 1420 (m) cm^{-1}; **HRMS** (ESI): calcd. for $C_{80}H_{48}Na^+$: 1031.3648; found: 1031.3668; $\boldsymbol{R_f}$ = 0.51 (isohexane/dichlormethane – 1/1).

The analytical data are in good accordance with the literature.[32]

4.2.20 Preparation of 1-(ethynyloxy)-2-(phenylethynyl)benzene **S58**

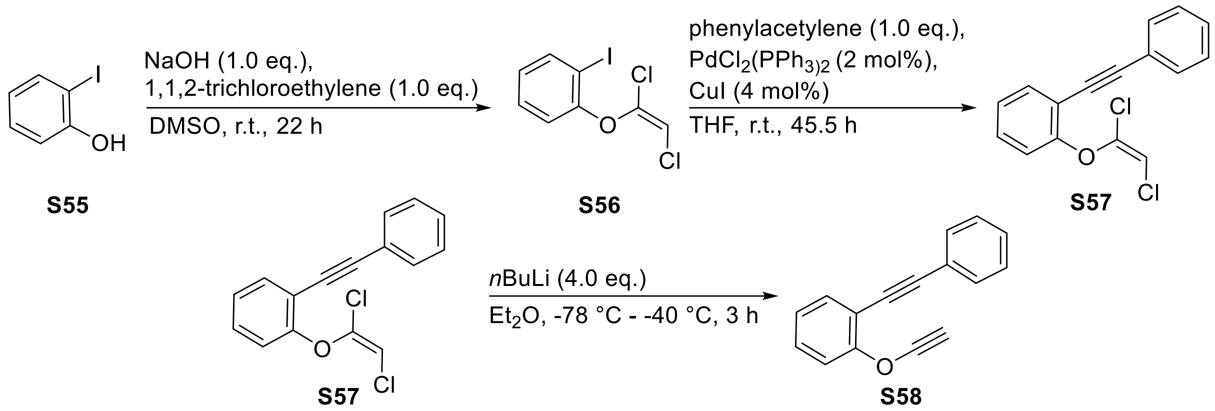

Preparation of (*E*)-1-((1,2-dichlorovinyl)oxy)-2-iodobenzene **S56**

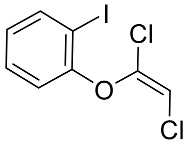

S56

The compound was prepared according to a literature-known procedure.[33] A round bottom flask was charged with a solution of 2-iodophenol **S55** (1.10 g, 5.00 mmol, 1.0 eq.) in anhydrous dimethylsulfoxide (10 mL). To this solution sodium hydroxid (200 mg, 5.00 mmol, 1.0 eq.) was added and the resulting mixture was stirred at room temperature for 2 hours. Afterwards 1,1,2-trichloroethylene (0.45 mL, 5.0 mmol, 1.0 eq.) was slowly added and the resulting mixture was stirred for 20 hours. The reaction was quenched by addition of water, and extracted with dichlormethane. The combined organic layers were washed with a saturated aqueous sodium chloride solution and dried over magnesium sulphate and the solvent was removed under reduced pressure. The title compound was isolated after purification by silica gel column chromatography (isohexane/ethyl acetate – 1/0) in a yield of 1.48 g (4.70 mmol, 94%) as a colourless liquid.

^{1}H NMR (300 MHz, $CDCl_3$) δ = 7.84 (dd, *J* = 7.9, 1.5 Hz, 1H), 7.41–7.29 (m, 1H), 7.00 (dd, *J* = 8.2, 1.2 Hz, 1H), 6.95–6.85 (m, 1H), 5.99 (s, 1H) ppm; **^{13}C NMR** (75 MHz, $CDCl_3$) δ = 153.3, 140.2, 140.0, 129.7, 126.4, 116.6, 104.3, 86.6 ppm; **IR** (ATR) $\tilde{\nu}$ = 682 (w), 745 (m), 827 (m), 1070 (s), 1118 (m), 1163 (m), 1208 (s), 1260 (m), 1353 (w), 1465 (s), 1577 (m), 1629 (m), 3097 (w) cm^{-1}; **MS** (EI): *m*/*z* (%): 316 (58), 314 (90), 251 (64), 238 (20), 203 (100), 159 (41), 152 (52), 127 (10), 111 (19), 92 (16), 76 (80), 63 (22); $\boldsymbol{R_f}$ = 0.57 (isohexane/ethyl acetate – 1/0).

The analytical data are in good accordance with the literature.[33]

Preparation of (*E*)-1-((1,2-dichlorovinyl)oxy)-2-(phenylethynyl)benzene **S57**

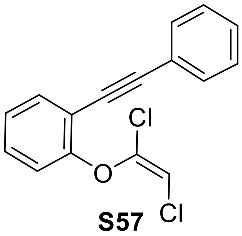

S57

The compound was prepared according to a literature-known procedure.[33] A dried two-necked flask was charged with iodobenzene **S56** (1.42 g, 4.50 mmol, 1.0 eq.), $PdCl_2(PPh_3)_2$ (62.2 mg, 90.0 µmol, 2 mol%) and copper(I)-iodide (34.3 mg, 180 µmol, 4 mol%). Anhydrous triethylamine (15 mL) was added and the resulting slurry was stirred at room temperature for 5 minutes. Phenylacetylene (0.49 mL, 4.5 mmol, 1.0 eq.) was added and the reaction mixture was stirred for 45.5 hours. The raction mixture was filtered over Celite and dried over magnesium sulphate. The solvent was removed underc reduced pressure. The title compound was isolated after purification by silica gel column chromatography (isohexane/ethyl acetate – 1/0) in a yield of 1.09 g (3.78 mmol, 84%) as a yellow liqiud.

^{1}H NMR (300 MHz, $CDCl_3$) δ = 7.62–7.50 (m, 3H), 7.41–7.29 (m, 4H), 7.18 (td, *J* = 7.6, 1.1 Hz, 1H), 7.08 (dd, *J* = 8.2, 1.0 Hz, 1H), 5.92 (s, 1H) ppm; **^{13}C NMR** (75 MHz, $CDCl_3$) δ = 154.0, 140.3, 133.9, 131.9, 129.6, 128.7, 128.5, 124.8, 123.2, 117.5, 115.5, 102.5, 94.8, 84.0 ppm; **IR** (ATR) $\tilde{v}$ =690 (m), 753 (s), 827 (m), 1066 (s), 1103 (m), 1159 (w), 1200 (s), 1260 (m), 1446 (m), 1495 (m), 1573 (w), 1595 (w), 1629 (m), 3101 (w) cm^{-1}; **MS** (EI): *m*/*z* (%): 288 (46), 253 (60), 225 (84), 218 (100), 189 (73), 176 (42), 165 (16), 150 (24), 139 (9), 126 (14), 109 (9), 95 (11), 87 (6), 75 (6), 63 (5); $\boldsymbol{R_f}$ = 0.37 (isohexane/ethyl acetate – 1/0).

The analytical data are in good accordance with the literature.[33]

Preparation of 1-(ethynyloxy)-2-(phenylethynyl)benzene **S58**

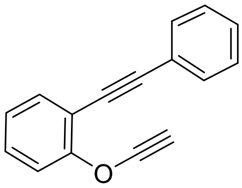

S58

The compound was prepared according to a literature-known procedure.[33] A dried two-necked flask was charged with a solution of the 1,2-dichlorovinyl ether **S57** (868 mg, 3.00 mmol, 1.0 eq.) in dry diethyl ether and cooled to −78 °C. At this temperature *n*-BuLi (7.5 mL, 12 mmol,

4.0 eq.) was dropwise added. The resulting solution was stirred for 1 hour before it was allowed to warm to −40 °C. The mixture was stirred at this temperature for another 2 hour before being quenched with water. The phases were separated and the aqueous layer was extracted with diethyl ether. The combined organic layers were washed with a saturated ammonium chloride solution and with a saturated aqueous sodium chloride solution, dried over magnesium sulphate and concentrated under reduced pressure. The title compound was isolated after purification by silica gel column chromatography (isohexane/ethyl acetate – 1/0) in a yield of 330 mg (1.51 mmol, 50%) as a red oil.

^{1}H NMR (400 MHz, $CDCl_3$) δ = 7.63–7.50 (m, 4H), 7.43–7.32 (m, 4H), 7.16 (td, J = 7.6, 1.1 Hz, 1H), 2.18 (s, 1H) ppm; **^{13}C NMR** (101 MHz, $CDCl_3$) δ = 155.6, 133.6, 131.9, 129.8, 128.8, 128.5, 124.6, 123.0, 114.1, 112.7, 95.2, 84.1, 83.3, 34.7 ppm; **IR** (ATR) $\tilde{\nu}$ = 693 (m), 753 (s), 891 (w), 939 (w), 973 (w), 1025 (w), 1073 (w), 1103 (m), 1155 (m), 1208 (m), 1241 (m), 1301 (m), 1375 (w), 1446 (m), 1476 (w), 1595 (m), 1677 (m), 1733 (m), 2926 (w), 2978 (w) cm^{-1}; **MS** (EI): *m*/*z* (%): 218 (100), 189 (44), 176 (15), 163 (9), 150 (9), 109 (11), 94 (12), 87 (3), 75 (3); ***R*$_f$** = 0.46 (isohexane/ethyl acetate – 1/0).

The analytical data are in good accordance with the literature.[33]

4.2.21 Preparation of 4-methyl-*N*,*N*-bis(3-phenylprop-2-yn-1-yl)benzenesulfonamide **S59**

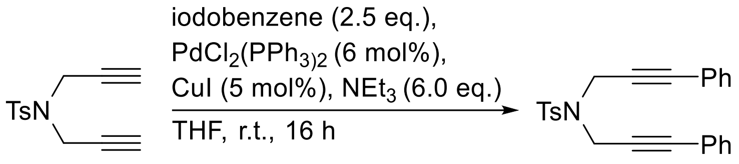

2 **S59**

Preparation of 4-methyl-*N*,*N*-bis(3-phenylprop-2-yn-1-yl)benzenesulfonamide **S59**

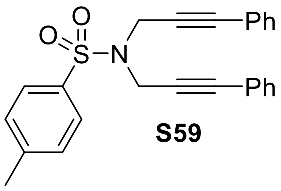

S59

The compound was prepared according to a literature-known procedure.[34] A dried two-necked flask was charged with tosylamide **2** (495 mg, 2.00 mmol, 1.0 eq.), $PdCl_2(PPh_3)_2$ (84.2 mg, 120 µmol, 6 mol%) and copper(I)-iodide (19.0 mg, 99.8 µmol, 5 mol%). Anhydrous tetrahydrofurane (5.0 mL) and anhydrous triethylamine (1.7 mL, 12 mmol, 6.0 eq.) was added and the resulting slurry was stirred at room temperature for 5 minutes. The iodobenzene (0.56 mL, 5.0 mmol, 2.5 eq.) was added and the reaction mixture was stirred for 16 hours. The reaction

was quenched by addition of a dilute hydrochloric acid (1 N) and ethyl acetate the layers were separated. The aqueous layer was extracted with ethyl acetate and the combined organic layers were washed with a saturated aqueous sodium chloride solution and dried over magnesium sulphate. The solvent was removed under reduced pressure. The title compound was isolated after purification by silica gel column chromatography (isohexane/ethyl acetate – 10/1) in a yield of 493 mg (1.23 mmol, 62%) as a brown solid.

^{1}H NMR (400 MHz, $CDCl_3$) δ = 7.83–7.73 (m, 2H), 7.37–7.14 (m, 12H), 4.45 (s, 4H), 2.32 (s, 3H) ppm; **^{13}C NMR** (101 MHz, $CDCl_3$) δ = 144.0, 135.6, 131.8, 129.7, 128.6, 128.3, 128.1, 122.4, 85.9, 81.8, 37.7, 21.6 ppm; **IR** (ATR) $\tilde{\nu}$ = 693 (m), 753 (s), 813 (m), 895 (m), 969 (w), 1092 (s), 1118 (m), 1159 (s), 1256 (w), 1349 (s), 1401 (w), 1442 (w), 1491 (m), 1595 (w), 2922 (w), 3056 (w) cm^{-1}; **HRMS** (ESI): calcd. for $C_{25}H_{21}NO_2SNa^+$: 422.1185; found: 422.1181; $\boldsymbol{R_f}$ = 0.40 (isohexane/ethyl acetate – 5/1).

The analytical data are in good accordance with the literature.[35]

4.2.22 Preparation of 3-phenylprop-2-yn-1-yl 3-phenylpropiolate **S61**

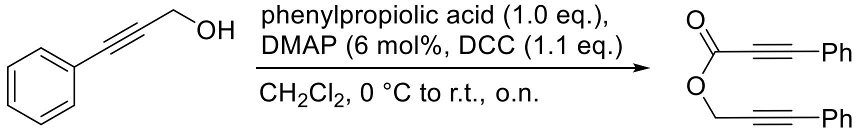

S60 **S61**

Preparation of 3-phenylprop-2-yn-1-yl 3-phenylpropiolate **S61**

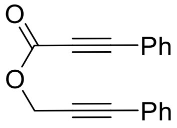

S61

To a solution of the phenylpropiolic acid (731 mg, 5.00 mmol, 1.0 eq.) and 3-phenyl-2-propyn-1-ol **S60** (0.62 mL, 5.0 mmol, 1.0 eq.) in anhydrous dichlormethane (50 mL) DMAP (36.7 mg, 300 µmol, 6 mol%) was added and cooled to 0 °C. To the resulting solution a solution of DCC (1.13 g, 5.50 mmol, 1.1 eq.) in anhydrous dichlormethane (50 mL) was dropped in slowly. After this the mixture was allowed to come to room temperature and stirred over night. The resulting suspension was filtered over celite. The organic layer was washed with dilute hydrochloric acid (0.25 N) and a saturated aqueous sodium hydrogencarbonate solution, dried over magnesium sulphate. The solvent was removed under reduced pressure. The title compound was isolated after purification by silica gel column chromatography (isohexane/ethyl acetate – 20/1) in a yield of 1.08 g (4.15 mmol, 83%) as a orange oil.

^{1}**H NMR** (400 MHz, $CDCl_3$) δ = 7.63–7.57 (m, 2H), 7.51–7.43 (m, 3H), 7.41–7.38 (m, 2H), 7.38–7.29 (m, 3H), 5.07 (s, 2H) ppm. ^{13}**C NMR** (101 MHz, $CDCl_3$) δ = 153.5, 133.3, 132.1, 131.0, 129.1, 128.8, 128.5, 122.1, 119.5, 87.6, 87.5, 82.1, 80.2, 54.4 ppm; **IR** (ATR) $\tilde{\nu}$ = 686 (s) ,753 (s), 865 (m), 921 (m), 954 (m), 988 (m), 1029 (m), 1070 (m),1155 (s),1182 (m), 1278 (s),1368 (m), 1442 (m), 1491 (m), 1595 (w), 1707 (s), 1767 (w), 2218 (m), 3056 (w) cm^{-1}; **HRMS** (ESI): calcd. for $C_{18}H_{12}O_2Na^+$: 283.0730; found: 283.0731; $\boldsymbol{R}_f$ = 0.27 (isohexane/ethyl acetate – 20/1).

4.2.23 Preparation of 3-phenyl-*N*-(prop-2-yn-1-yl)-*N*-tosylpropiolamide **S62**

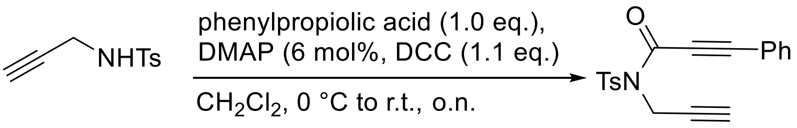

S7 **S62**

Preparation of 3-phenyl-*N*-(prop-2-yn-1-yl)-*N*-tosylpropiolamide **S61**

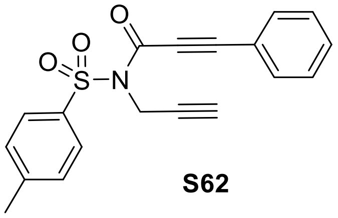

S62

To a solution of the tosylamide **S7** (1.26 g, 6.00 mmol, 1.0 eq.) and phenylpropiolic acid (877 mg, 6.00 mmol, 1.0 eq.) in anhydrous dichlormethane (60 mL) DMAP (44.0 mg, 0.36 mmol, 6 mol%) was added and cooled to 0 °C. To the resulting solution a solution of DCC (1.36 g, 6.60 mmol, 1.1 eq.) in anhydrous dichlormethane (60 mL) was dropped in slowly. After this the mixture was allowed to come to room temperature and stirred over night. The resulting suspension was filtered over celite. The organic layer was washed with dilute hydrochloric acid (0.25 N) and a saturated aqueous sodium hydrogencarbonate solution, dried over magnesium sulphate. The solvent was removed under reduced pressure. The title compound was isolated after purification by silica gel column chromatography (isohexane/ethyl acetate – 5/1) in a yield of 1.07 g (3.17 mmol, 53%) as a yellow solid.

^{1}**H NMR** (300 MHz, $CDCl_3$) δ = 8.05 – 7.97 (m, 2H), 7.60 – 7.53 (m, 2H), 7.52 – 7.45 (m, 1H), 7.44 – 7.35 (m, 2H), 7.31 (d, *J*=8.1, 2H), 4.86 (d, *J*=2.4, 2H), 2.43 (s, 3H), 2.35 (t, *J*=2.4, 1H) ppm. ^{13}**C NMR** (101 MHz, $CDCl_3$) δ = 152.1, 145.5, 135.5, 133.0, 131.3, 129.6, 129.0, 128.9, 119.4, 94.2, 81.3, 78.1, 73.1, 36.3, 21.9 ppm; **IR** (ATR) $\tilde{\nu}$ = 656 (s), 690 (s), 727 (s), 760 (m), 813 (m), 868 (m), 943 (m), 1088 (m), 1122 (s), 1185 (m), 1275 (m), 1327 (s), 1353 (s), 1416 (w), 1442 (w), 1491 (w), 1595 (m), 1670 (s), 2210 (m) ,3284 (w), 3284 (w) cm^{-1}; **HRMS** (ESI):

calcd. for $C_{19}H_{15}NO_3SNa^+$: 360.0665; found: 360.0674; $\boldsymbol{R_f}$ = 0.36 (isohexane/ethyl acetate – 5/1).

4.2.24 Preparation of But-2-yn-1-yl 3-phenylpropiolate **S64**

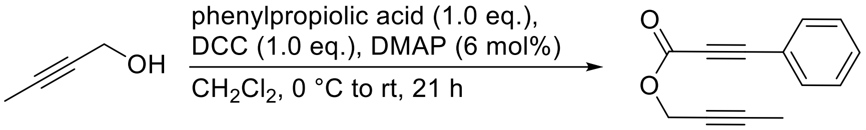

S63 **S64**

Preparation of But-2-yn-1-yl 3-phenylpropiolate **S64**

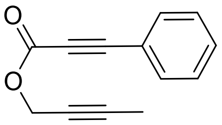

S64

The compound was prepared according to a literature-known procedure.[36] To a solution of the phenylpropiolic acid (438 mg, 3.00 mmol, 1.0 eq.) in anhydrous dichlormethane (30 mL) at 0 °C 2-butyn-1-ol **S63** (0.22 ml, 3.0 mmol, 1.0 eq.), DMAP (22.0 mg, 180 µmol, 6 mol%) and DCC (619 mg, 3.00 mmol, 1.0 eq.) were added. The mixture was allowed to come to room temperature and stirred for 21 hours. The resulting suspension was filtered over silica and washed with dilute hydrochloric acid (1 N) and a saturated aqueous sodium hydrogencar-bonate solution. The combined aqueous layers were extracted with dichlormethane and the combined organic layers were washed with a saturated aqueous sodium chloride solution and dried over magnesium sulphate. The solvent was removed under reduced pressure. The title compound was isolated after purification by silica gel column chromatography (isohexane/ethyl acetate – 1/0 to 50/1) in a yield of 416 mg (2.10 mmol, 70%) as a colourless liquide.

^{1}H NMR (300 MHz, $CDCl_3$) δ = 7.62–7.55 (m, 2H), 7.51–7.41 (m, 1H), 7.41–7.32 (m, 2H), 4.80 (q, J = 2.4 Hz, 2H), 1.88 (t, J = 2.4 Hz, 3H) ppm; **^{13}C NMR** (75 MHz, $CDCl_3$) δ = 153.5, 133.2, 130.9, 128.7, 119.6, 87.3, 84.4, 80.2, 72.4, 54.4, 3.8 ppm; **IR** (ATR) $\tilde{\nu}$ = 690 (m), 760 (m), 969 (m), 1159 (s), 1278 (s), 1372 (w), 1442 (w), 1491 (w), 1707 (s), 2221 (m) cm^{-1}; **MS** (EI): *m*/*z* (%): 198 (4), 169 (19), 153 (54), 140 (45), 129 (100), 115 (9), 102 (41), 75 (32), 51 (17); $\boldsymbol{R_f}$ = 0.61 (isohexane/ethyl acetate – 9/1).

The analytical data are in good accordance with the literature.[36]

4.2.25 Preparation of 1,1'-(1,2-Phenylene)bis(prop-2-yn-1-ol) **S66**

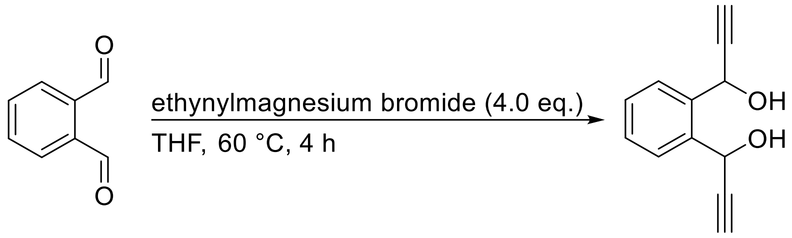

S65 **S66**

Preparation of 1,1'-(1,2-Phenylene)bis(prop-2-yn-1-ol) **S66**

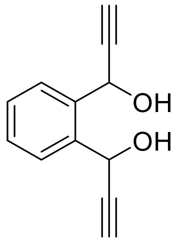

S66

The compound was prepared according to a literature-known procedure.[37] A dried two-necked flask was charged with ethynylmagnesium bromide (0.5 M in THF, 24.0 mL, 12.0 mmol, 4.0 Äq.). Phthaldialdehyde **S65** (402 mg, 3.00 mmol, 1.0 eq.) was added and the resulting mixture was stirred at 60 °C for 4 hours. The reaction was quenched by addition of saturated ammonium-chloride solution and extracted with ethyl acetate. The combined organic layers were washed with a saturated aqueous sodium chloride solution and dried over magnesium sulphate and the solvent was removed under reduced pressure. The title compound was isolated after purification by silica gel column chromatography (isohexane/ethyl acetate – 2/1) in a yield of 525 mg (2.82 mmol, 94 %) as an orange oil (mixture of two diastereomers 1.0/0.6).

^{1}H NMR (300 MHz, $CDCl_3$) δ = 7.89–7.80 (m, 1H), 7.72–7.61 (m, 0.6H), 7.46–7.34 (m, 1.6H), 6.01 (s, 0.6H), 5.88 (s, 1H), 3.62 (s, 0.6H), 3.19 (d, J = 3.8 Hz, 1H), 2.74 (d, J = 2.3 Hz, 1H), 2.72 (d, J = 2.3 Hz, 0.6H) ppm; **^{13}C NMR** (101 MHz, $CDCl_3$) δ = 137.7, 137.4, 129.5, 129.4, 129.3, 128.1, 82.8, 82.5, 76.2, 75.9, 63.3, 61.9 ppm; **IR** (ATR) $\tilde{\nu}$ = 760 (s), 809 (m), 947 (s), 1010 (s), 1204 (m) ,1252 (m), 1401 (m), 1454 (m), 3284 (m) cm^{-1}; **HRMS** (ESI): calcd. for $C_{12}H_{10}O_2Na^+$: 209.0573; found: 209.584; $\boldsymbol{R_f}$ = 0.29 (isohexane/ethyl acetate – 5/1).

The analytical data are in good accordance with the literature.[37]

4.2.26 Preparation of (3*S*,9*S*,10*R*,13*R*,14*S*,17*R*)-10,13-Dimethyl-17-((*R*)-6-methylheptan-2-yl)-3-(prop-2-yn-1-yloxy)-2,3,4,7,8,9,10,11,12,13,14,15,16,17-tetradecahydro-1*H*-cyclopenta[*a*]phenanthrene **S68**

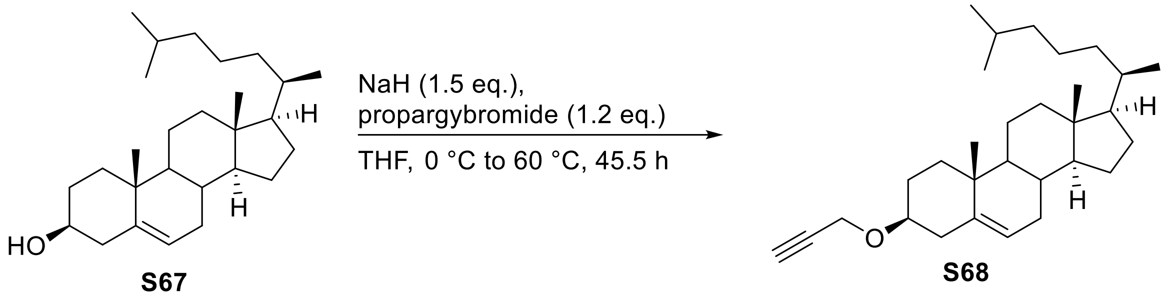

Preparation of (3*S*,9*S*,10*R*,13*R*,14*S*,17*R*)-10,13-Dimethyl-17-((*R*)-6-methylheptan-2-yl)-3-(prop-2-yn-1-yloxy)-2,3,4,7,8,9,10,11,12,13,14,15,16,17-tetradecahydro-1*H*-cyclopenta[*a*]phenanthrene **S68**

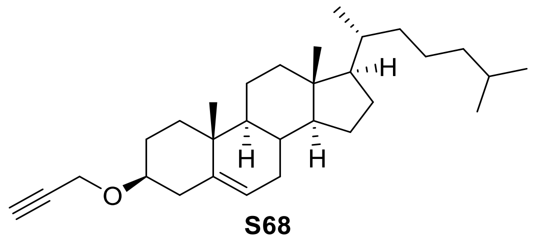

The compound was prepared according to a literature-known procedure.[38] Cholesterol **S67** (1.92 g, 5.00 mmol, 1.0 eq.) was dissolved in anhydrous THF (15 mL). The solution was cooled down to 0 °C and NaH (60 % in mineral oil, 300 mg, 7.50 mmol, 1.5 eq.) was added. Stirring was continued for 10 minutes, subsequently propargyl bromide (80% in toluene, 0.67 mL, 6.0 mmol, 1.2 eq.) was added dropwise. The reaction mixture was warmed to 60 °C and was stirred for 45.5 hours. The reaction was quenched by addition of water and extracted with diethyl ether. The combined organic layers were washed with a saturated aqueous sodium chloride solution and dried over magnesium sulphate and the solvent was removed under reduced pressure. The title compound was isolated after purification by silica gel column chromatography (isohexane/ethyl acetate – 1/0 to 100/1) in a yield of 924 mg (2.18 mmol, 44%) as a yellow liquid.

^{1}**H NMR** (400 MHz, $CDCl_3$) δ = 5.41–5.29 (m, 1H), 4.19 (d, *J* = 2.4 Hz, 2H), 3.38 (tt, *J* = 11.3, 4.5 Hz, 1H), 2.44–2.34 (m, 2H), 2.28–2.14 (m, 1H), 2.08–1.77 (m, 5H), 1.66–0.81 (m, 33H), 0.67 (s, 3H) ppm; ^{13}**C NMR** (101 MHz, $CDCl_3$) δ = 140.7, 122.0, 80.6, 78.3, 73.9, 56.9, 56.3, 55.2, 50.3, 42.5, 39.9, 39.7, 38.9, 37.3, 37.0, 36.3, 35.9, 32.1, 32.0, 28.4, 28.2, 28.2, 24.4, 24.0, 23.0, 22.7, 21.2, 19.5, 18.9, 12.0 ppm; **IR** (ATR) $\tilde{\nu}$ = 663 (m), 734 (m), 801 (w), 909 (m),

943 (w), 1021 (m), 1085 (s), 1379 (m), 1465 (m), 2866 (m), 2933 (s) cm^{-1}; **HRMS** (ESI): calcd. for $C_{27}H_{45}^{+}$: 369.3516; found: 369.3513; ***R*$_f$** = 0.09 (isohexane/ethyl acetate – 1/0).

The analytical data are in good accordance with the literature.[38]

4.2.27 Preparation of (*S*)-6-Chloro-4-(cyclopropylethynyl)-1-methyl-4-(trifluoromethyl)-1,4-dihydro-2*H*-benzo[*d*][1,3]oxazin-2-one **S70**

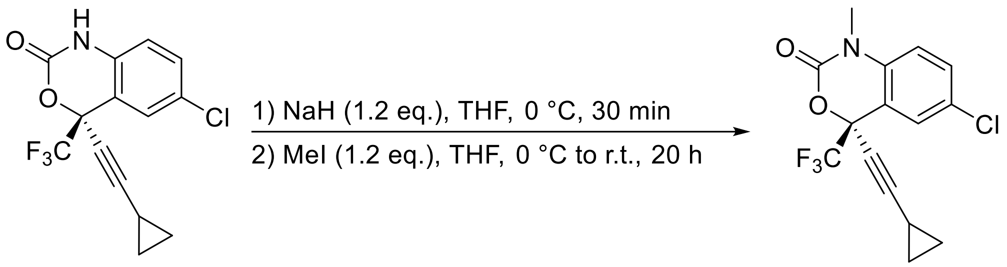

S69 **S70**

Preparation of (*S*)-6-Chloro-4-(cyclopropylethynyl)-1-methyl-4-(trifluoromethyl)-1,4-dihydro-2*H*-benzo[*d*][1,3]oxazin-2-one **S70**

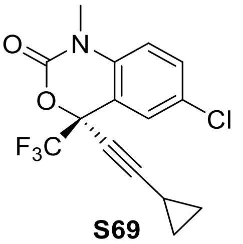

S69

The compound was prepared according to a literature-known procedure.[X] Efavirenz **S69** (947 mg, 3.00 mmol, 1.0 eq.) was dissolved in anhydrous THF (15 mL). The solution was cooled down to 0 °C and NaH (60 % in mineral oil, 144 mg, 3.60 mmol, 1.2 eq.) was added. Stirring was continued for 30 minutes, subsequently iodomethane (0.22 mL, 3.5 mmol, 1.2 eq.) was added dropwise. The reaction mixture was warmed to room temperature and was stirred for 20 hours. The reaction was quenched by addition of water and extracted with ethyl acetate. The combined organic layers were washed with a saturated aqueous sodium chloride solution and dried over magnesium sulphate and the solvent was removed under reduced pressure. The title compound was isolated after purification by silica gel column chromatography (isohexane/ethyl acetate – 8/1) in a yield of 944 mg (2.86 mmol, 95%) as a colourless oil.

^{1}H NMR (300 MHz, $CDCl_3$) δ = 7.54 (d, J = 2.1 Hz, 1H), 7.43 (dd, J = 8.7, 2.4 Hz, 1H), 6.90 (d, J = 8.8 Hz, 1H), 3.39 (s, 3H), 1.44–1.33 (m, 1H), 0.96–0.87 (m, 2H), 0.87–0.80 (m, 2H) ppm; **^{13}C NMR** (75 MHz, $CDCl_3$) δ = 148.3, 136.2, 131.6, 129.0, 128.1, 122.4, 117.6, 115.0, 95.7, 66.4, 31.9, 8.9, 8.9, –0.5 ppm; **IR** (ATR) $\tilde{\nu}$ = 682 (m), 716 (m), 742 (m), 813 (m), 883 (m), 924 (s) 962 (m), 1036 (m), 1073 (m), 1107 (m), 1137 (s), 1185 (s), 1260 (s), 1342 (m), 1424 (m), 1495 (s), 1603 (m), 1733 (s), 2248 (w) cm^{-1}; **HRMS** (ESI): calcd. for $C_{15}H_{11}ClF_3NO_2Na^+$: 352.0323; found: 352.0325; $\boldsymbol{R}_\mathbf{f}$ = 0.20 (isohexane/ethyl acetate – 8/1).

4.2.28 Preparation of (2*S*,5*R*)-2-((*R*)-(6-Methoxyquinolin-4-yl)(prop-2-yn-1-yloxy)methyl)-5-vinylquinuclidine **S72**

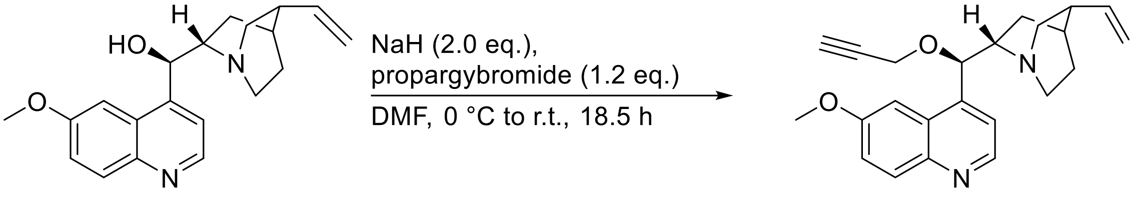

Preparation of (2*S*,5*R*)-2-((*R*)-(6-Methoxyquinolin-4-yl)(prop-2-yn-1-yloxy)methyl)-5-vinylquinuclidine **S72**

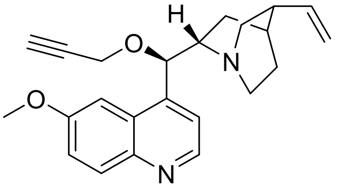

S72

The compound was prepared according to a literature-known procedure.[39] NaH (60 % in mineral oil, 300 mg, 7.50 mmol, 1.5 eq.) was suspended in anhydrous dimethylformamide (7.5 mL). The solution was cooled down to 0 °C and a solution of quinine **S71** (1.62 g, 5.00 mmol, 1.0 eq.) in dimethylformamide (7.5 mL) was added. Stirring was continued for 10 minutes, subsequently propargyl bromide (80% in toluene, 0.67 mL, 6.0 mmol, 1.2 eq.) was added dropwise. The reaction mixture was warmed to room temperature and was stirred for 19.5 hours. The reaction was quenched by addition of water and extracted with diethyl ether. The combined organic layers were washed with a saturated aqueous sodium chloride solution and dried over magnesium sulphate and the solvent was removed under reduced pressure. The title compound was isolated after purification by silica gel column chromatography (isohexane/ethyl acetate – 1/2 + 2% Et_3N) in a yield of 938 mg (2.59 mmol, 52 %) as a yellow liquid.

^{1}H NMR (300 MHz, $CDCl_3$) δ = 8.75 (d, *J* = 4.5 Hz, 1H), 8.04 (d, *J* = 9.8 Hz, 1H), 7.45–7.33 (m, 3H), 5.75 (ddd, *J* = 17.6, 10.3, 7.6 Hz, 1H), 5.35 (s, 1H), 5.00–4.86 (m, 2H), 4.22 (dd, *J* = 15.8, 2.4 Hz, 1H), 4.00–3.85 (m, 4H), 3.51–3.36 (m, 1H), 3.22–3.01 (m, 2H), 2.77–2.55 (m, 2H), 2.45 (t, *J* = 2.4 Hz, 1H), 2.33–2.20 (m, 1H), 1.86–1.61 (m, 4H), 1.60–1.45 (m, 1H) ppm; **^{13}C NMR** (75 MHz, $CDCl_3$) δ = 158.0, 147.7, 144.9, 144.0, 142.0, 132.0, 127.7, 121.9, 119.3, 114.4, 101.4, 79.5, 75.1, 60.2, 57.2, 56.4, 55.9, 43.2, 40.1, 27.9, 27.9, 23.1 ppm (due to coincidental chemical equivalence of two carbon resonances one signal is missing); **IR** (ATR) $\tilde{\nu}$ = 667 (m), 716 (m), 760 (m), 854 (s), 913 (m), 958 (w), 1029 (s), 1073 (s), 1133 (m), 1170 (m), 1226 (s), 1301 (m), 1357 (m), 1472 (m), 1506 (s), 1618 (s), 2863 (m), 2930 (m), 3291 (w) cm^{-1}; **HRMS** (ESI): calcd. for $C_{23}H_{27}N_2O_2^+$: 363.2067 found: 363.2090; ***R*$_f$** = 0.24 (isohexane/ethyl acetate – 1/2 + 5 % Et_3N).

The analytical data are in good accordance with the literature.[39]

4.2.29 Preparation of Prop-2-yn-1-yl 2-(1-(4-chlorobenzoyl)-5-methoxy-2-methyl-1*H*-indol-3-yl)acetate **S74**

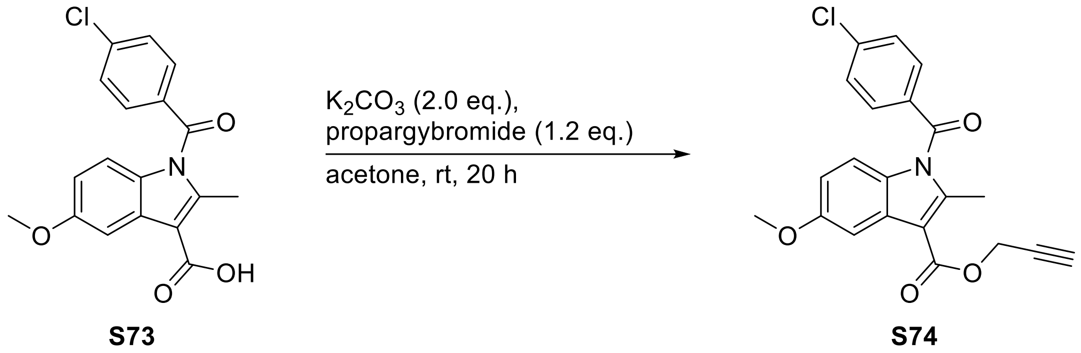

Preparation of Prop-2-yn-1-yl 2-(1-(4-chlorobenzoyl)-5-methoxy-2-methyl-1*H*-indol-3-yl)acetate **S74**

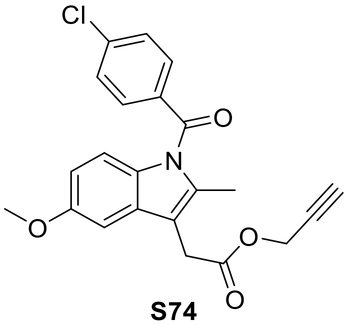

S74

The compound was prepared according to a literature-known procedure.[40] Indometacin **S73** (1.72 g, 5.00 mmol, 1.0 eq.) and potassium carbonate (1.38 g, 10.0 mmol, 2.0 eq.) were suspended in acetone (50 mL). Propargyl bromide (80% in toluene, 1.1 ml, 10 mmol, 2.0 eq.) was added and the reaction mixture was stirred at room temperature for 20 hours. After filtration (silica), the volatiles were removed under reduced pressure and the crude product was diluted with ethyl acetate and washed with water. The aqueous layer was extracted with ethyl acetate and the combined organic layers were washed with a saturated aqueous sodium chloride solution and dried over magnesium sulphate. The solvent was removed under reduced pressure. The title compound was isolated after purification by silica gel column chromatography (isohexane/ethyl acetate – 8/1) in a yield of 734 mg (1.92 mmol, 38 %) as a yellow oil.

^{1}H NMR (400 MHz, $CDCl_3$) δ = 7.71–7.63 (m, 2H), 7.52–7.42 (m, 2H), 6.96 (d, *J* = 2.5 Hz, 1H), 6.87 (d, *J* = 9.0 Hz, 1H), 6.67 (dd, *J* = 9.0, 2.5 Hz, 1H), 4.71 (d, *J* = 2.5 Hz, 3H), 3.84 (s, 2H), 3.72 (s, 2H), 2.48 (t, *J* = 2.5 Hz, 1H), 2.39 (s, 3H) ppm; **^{13}C NMR** (101 MHz, $CDCl_3$) δ = 170.2, 168.4, 156.2, 139.5, 136.3, 134.0, 131.3, 130.9, 130.6, 129.3, 115.1, 112.1, 112.0, 101.3, 77.6, 75.3, 55.9, 52.6, 30.2, 13.5; **IR** (ATR) $\tilde{\nu}$ = 671 (m), 745 (m), 813 (m), 854 (m), 891 (m), 924

(m), 958 (m), 1029 (s), 1088 (m) 1148, (s) 1215, (m) 1264 (m), 1323 (m), 1390 (m), 1454 (m), 1506 (m), 1603 (m), 1633 (m), 1737 (s), 2937 (w), 2974 (w), 3284 (w) cm^{-1}; **HRMS** (ESI): calcd. for $C_{22}H_{18}ClNO_4Na^+$: 418.0817 found: 418.0810; ***R*f** = 0.29 (isohexane/ethyl acetate – 8/1).

The analytical data are in good accordance with the literature.[40]

4.2.30 Preparation of Prop-2-yn-1-yl (*S*)-2-(6-methoxynaphthalen-2-yl)propanoate **S76**

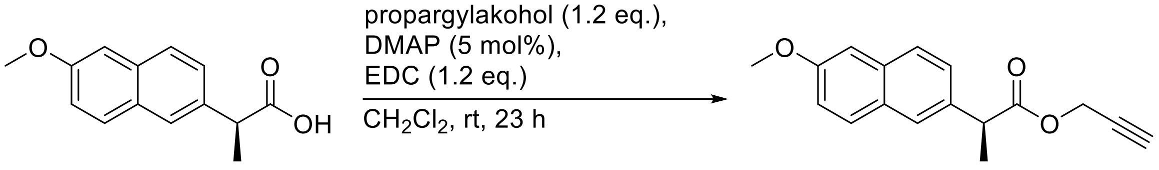

S75 **S76**

Preparation of Prop-2-yn-1-yl (*S*)-2-(6-methoxynaphthalen-2-yl)propanoate **S76**

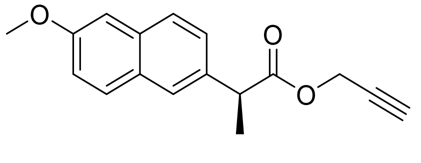

S75

The compound was prepared according to a literature-known procedure.[40] To a solution of naproxen **S75** (691 mg, 3.00 mmol, 1.0 eq.) and 2-propyn-1-ol (0.21 ml, 3.6 mmol, 1.2 eq.) in anhydrous dichlormethane (30 mL) DMAP (18.3 mg, 150 µmol, 5 mol%) and EDCl (690 mg, 3.60 mmol, 1.2 eq.) was added. The reaction mixture was stirred at room temperature for 23 hours. The reaction was quenched by addition of water and extracted with dichlormethane. The combined organic layers were washed with a saturated aqueous sodium chloride solution and dried over magnesium sulphate and the solvent was removed under reduced pressure. The title compound was isolated after purification by silica gel column chromatography (iso-hexane/ethyl acetate – 10/1) in a yield of 694 mg (2.59 mmol, 86 %) as a yellow liquid.

^{1}H NMR (400 MHz, $CDCl_3$) δ = 7.76–7.65 (m, 3H), 7.41 (dd, J = 8.5, 1.8 Hz, 1H), 7.18–7.08 (m, 2H), 4.67 (ddd, J = 46.0, 15.6, 2.5 Hz, 2H), 3.92 (s, 4H), 2.44 (t, J = 2.5 Hz, 1H), 1.60 (d, J = 7.2 Hz, 3H) ppm; **^{13}C NMR** (101 MHz, $CDCl_3$) δ = 174.0, 157.8, 135.2, 133.9, 129.4, 129.0, 127.4, 126.3, 126.2, 119.2, 105.7, 77.7, 75.0, 55.5, 52.4, 45.3, 18.7; **IR** (ATR) $\tilde{\nu}$ = 690 (m), 753 (m), 835 (m), 924 (m), 1014 (m), 1088 (m), 1141 (s), 1219 (s), 1260 (m), 1312, (s) 1357 (s), 1401 (m), 1476 (s), 1592 (m), 1677 (s), 1737 (m), 2833 (w), 2930 (w), 3280 (w) cm^{-1}; **HRMS**

(ESI): calcd. for $C_{17}H_{16}O_3Na^+$: 291.0992 found: 291.1001; $\boldsymbol{R_f}$ = 0.40 (isohexane/ethyl acetate – 8/1).

The analytical data are in good accordance with the literature.[40]

4.2.31 Preparation of (3a'*R*,4*S*,7'*S*,7a'*R*)-2,2,2',2'-Tetramethyl-7'-(prop-2-yn-1-yloxy)tetrahydrospiro[[1,3]dioxolane-4,6'-[1,3]dioxolo[4,5-*c*]pyran] **S78**

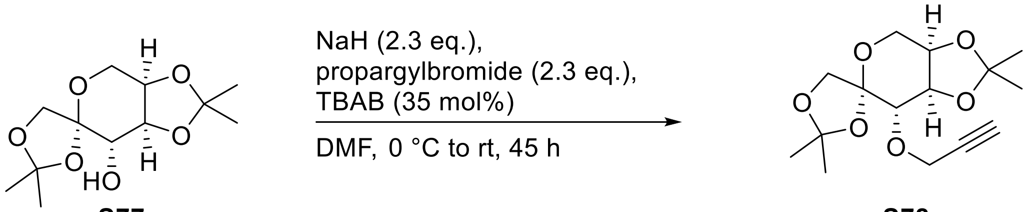

S77 **S78**

Preparation of (3a'*R*,4*S*,7'*S*,7a'*R*)-2,2,2',2'-Tetramethyl-7'-(prop-2-yn-1-yloxy)tetrahydrospiro[[1,3]dioxolane-4,6'-[1,3]dioxolo[4,5-*c*]pyran] **S78**

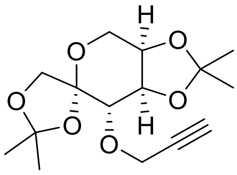

S78

The compound was prepared according to a literature-known procedure.[41] Fructopyranose **S75** (1.30 g, 4.37 mmol, 1.0 eq.) was dissolved in anhydrous diemthylformamide (50 mL). The solution was cooled down to 0 °C and NaH (60 % in mineral oil, 400 mg, 10.0 mmol, 2.3 eq.) was added. Stirring was continued for 30 minutes, subsequently propargyl bromide (80% in toluene, 1.1 ml, 10 mmol, 2.3 eq.) and TBAB (500 mg, 1.55 mmol, 35 mol%) was added. The reaction mixture was warmed to room temperature and was stirred for 45 hours. The reaction was quenched by addition of water and extracted with ethyl acetate. The combined organic layers were washed with a saturated aqueous sodium chloride solution and dried over magnesium sulphate and the solvent was removed under reduced pressure. The title compound was isolated after purification by silica gel column chromatography (isohexane/ethyl acetate – 8/1) in a yield of 1.17 g (3.94 mmol, 90 %) as a yellow oil.

^{1}H NMR (300 MHz, $CDCl_3$) δ = 4.49 (qd, *J* = 16.0, 2.4 Hz, 2H), 4.31 (dd, *J* = 7.5, 5.5, 1H), 4.25 (d, *J* = 8.6 Hz, 1H), 4.22 – 4.17 (m, 1H), 4.16 – 4.07 (m, 1H), 4.02 (d, *J* = 13.4 Hz, 1H), 3.95 (d, *J* = 8.6 Hz, 1H), 3.75 (d, *J* = 7.5 Hz, 1H), 2.42 (t, *J* = 2.4 Hz, 1H), 1.58 (s, 3H), 1.49 (s, 3H),

1.41 (s, 3H), 1.36 (s, 3H) ppm; **^{13}C NMR** (75 MHz, $CDCl_3$) δ = 112.3, 109.3, 104.4, 79.8, 77.8, 74.5, 74.3, 74.1, 72.0, 60.3, 58.5, 28.3, 26.9, 26.5, 26.2 ppm; **IR** (ATR) $\tilde{\nu}$ = 671 (w), 772 (w), 805 (m), 850 (m), 883 (m), 977 (m), 1018 (m), 1081 (s), 1114 (m), 1185 (m), 1219 (m), 1327 (w), 1372 (m), 1454 (w), 2889 (w), 2933 (w), 2989 (w), 3265 (w) cm^{-1}; **HRMS** (ESI): calcd. for $C_{15}H_{22}O_6Na^+$: 321.1309; found: 321.1319; $\boldsymbol{R_f}$ = 0.21 (isohexane/ethyl acetate – 8/1).

The analytical data are in good accordance with the literature.[42]

4.2.32 Preparation of 4'-(Hydroxymethyl)-5'-methyl-6'-phenyl-[1,1':2',1''-terphenyl]-3'-carboxylic acid **58**

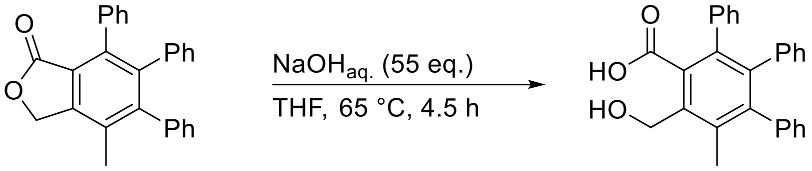

51 **58**

Preparation of 4'-(Hydroxymethyl)-5'-methyl-6'-phenyl-[1,1':2',1''-terphenyl]-3'-carboxylic acid **58**

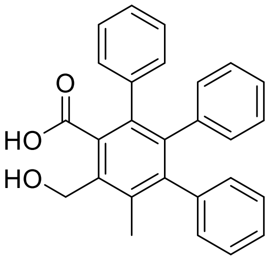

58

To a solution of lactone **51** (113 mg, 300 µmol, 1.0 eq.) in anhydrous THF (10 mL) NaOH (10% in water, 6.0 mL, 17 mmol, 55 eq.) was added. The reaction mixture was warmed to 65 °C and was stirred for 4.5 hours. The reaction was quenched by addition of saturated potassium bisulfate solution (acidify to pH 3). The mixture was diluted with water and extracted with ethyl acetate. The combined organic layers were dried over magnesium sulphate and the solvent was removed under reduced pressure. The title compound was isolated in a yield of 93.4 mg (237 µmol, 79 %) as a colourless foam.

5. References

1. F. Rami, B. Klinnert, J. Nowak, F. Ullwer, M. Zheng, W. Frey, B. Plietker, *Org. Lett*., **26**, 6370−6374 (2024).

2. S. Rommel, L. Hettmanczyk, J. E. M. N. Klein, B. Plietker, *Chem. Asian J.*, **9**, 2140 – 2147 (2014).

3. V. A. Rassadin, E. Nicolasb, Y. Six, *Chem. Commun*., **60,** 7666-7669 (2014).

4. M. Féo, N. Bakas, A. Radović, W. Parisot, A. Clisson, L.-M. Chamoreau, M. Haddad, V. Ratovelomanana-Vidal, M. L. Neidig, G. Lefèvre, *ACS Catal*., **13**, 4882−4893 (2023).

5. J. Jacquet, A.-L. Auvinet, A. K. Mandadapu, M. Haddad, V. Ratovelomanana-Vidal, V. Michelet, *Adv. Synth. Catal*., **357**, 1387-1392 (2015).

6. I. González, A. Pla-Quintana, A. Roglans, *Synlett*, **17**, 2844–2848 (2009).

7. Q. Huang, M.-Y. Hu, S.-F. Zhu, *Org. Lett*., **21**, 7883−7887 (2019).

8. R. Abe, Y. Nagashima, J. Tanaka, K. Tanaka, *ACS Catal*., **13**, 1604−1613 (2023).

9. F. Kramm, F. Ullwer, B. Klinnert, M. Zheng, B. Plietker, *Angew. Chem. Int. Ed*., **61**, e202205169 (2022).

10. S. Kotha, G. Sreevani, *ACS Omega*, **3**, 1850−1855 (2018).

11. A. Stephen K. Hashmi, T. Häffner, M. Rudolph, F. Rominger, *Chem. Eur. J.*, **17**, 8195 – 8201 (2011).

12. V. Richard, M. Ipouck, D. S. Mérel, S. Gaillard, R. J. Whitby, B. Witulski, J.-L. Renaud, Iron(ii)-catalysed [2+2+2] cycloaddition for pyridine ring construction, *Chemical Communications*, **50**, 593–595 (2014).

13. S. Moulin, H. Dentel, A.a Pagnoux-Ozherelyeva, S. Gaillard, A. Poater, L. Cavallo, J.-F. Lohier, J.-L. Renaud, *Chem. Eur. J*., **19**, 17881–17890 (2013).

14. I. Manjón-Mata, M. T. Quirós, E. Velasco-Juárez, E. Buñuel, D. J. Cárdenas, *Adv. Synth. Catal.*, **364**, 1716–1723 (2022).

15. V. P. Dyadchenko, M. A. Dyadchenko, V. N. Okulov, D. A. Lemenovskii, *Journal of Organometallic Chemistry,* **696**, 468–472 (2011).

16. S.-H. Kim-Lee, I. Alonso, P. Mauleón, R. G. Arrayás, J. C. Carretero, *ACS Catal*., **8**, 8993−9005 (2018).

17. A. Arlegui, P. Torres, V. Cuesta, J. Crusats, A. Moyano, *Eur. J. Org. Chem*., 4399–4407 (2020).

18. H. Rao, H. Fu, Y. Jiang, Y. Zhao, *Adv. Synth. Catal*., **352**, 458–462 (2010).

19. R. Liu, Z. Ni, L. Giordano, A. Tenaglia, *Org. Lett*., **18**, 4040−4043 (2016).

20. H.-H. Lin, T.-C. C., R.-X. Wu, Y.-M. Chang, H.-W. Wang, S.-T. Liu, M.-Ch. P. Yeh, *Adv. Synth. Catal*., **361**, 1277 – 1282 (2019).

21. H.-M. Huang, P. Bellotti, C. G. Daniliuc, F. Glorius, *Angew. Chem. Int. Ed*., **60**, 2464 – 2471 (2021).

22. F. Ye, F. Boukattaya, M. Haddad, V. Ratovelomanana-Vidal, V. Michelet, *New J. Chem*., **42**, 3222–3235 (2018).

23. A. L. Jones, J. K. Snyder, *J. Org. Chem*., **74**, 2907–2910 (2009).

24. G. Fabrizi, A. Goggiamani, A. Sferrazza, S. Cacchi, *Angew. Chem. Int. Ed.*, **4***9*, 4067–4070 (2010).

25. G. Jin, X. Zhang, S. Cao, *Org. Lett*., **15**, 3114-3117 (2013).

26. R. E. Whittaker, G. Dong, *Org. Lett*., **17**, 5504−5507 (2015).

27. R. Cai, M. Lu, E. Y. Aguilera, Y. Xi, N. G. Akhmedov, J. L. Petersen, H. Chen, X. Shi, *Angew. Chem. Int. Ed*., **54**, 8772–8776 (2015).

28. S. Nicolai, S. Erard, D. F. González, J. Waser, *Org. Lett*., **12**, 384-387 (2010).

29. Z. Shen, X. Lu, *Adv. Synth. Catal*., **351**, 3107–3112 (2009).

30. M. F. Martínez-Esperón, D. Rodríguez, L. Castedo, C. Saá, *Org. Lett*., **7**, 2213–2216 (2005).

31. S. T. Fard, K. Sekine, K. Farshadfar, F. Rominger M. Rudolph, A. Ariafard, A. Stephen, K. Hashmi, *Chem. Eur. J.*, **27**, 3552–3559 (2021).

32. M. Wehmeier, M. Wagner, K. Müllen, *Chem. Eur. J*., **7**, 2197-2205 (2001).

33. K. Graf, C. L. Rühl, M. Rudolph, F. Rominger, A. Stephen, K. Hashmi, *Angew. Chem. Int. Ed.*, **52**, 12727–12731 (2013).

34. M. R. Mutra, Y.-T. Chen, J.-J. Wang, *Adv. Synth. Catal.*, **365**, 1012–1019 (2023).

35. K. R. Strom, A. C. Impastato, K. J. Moy, A. J. Landreth, J. K. Snyder, *Org. Lett.*, **17**, 2126−2129 (2015).

36. M. K. Smith, J. A. Tunge, Palladium-Catalyzed Synthesis of Conjugated Allenynes via Decarboxylative Coupling, *Org. Lett.*, **19**, 5497–5500 (2017).

37. J. Miguel-Ávila, M. Tomás-Gamasa, J. L. Mascareñas, Intracellular Ruthenium-Promoted (2+2+2) Cycloadditions, *Angewandte Chemie International Edition*, **59**, 17628–17633 (2020).

38. J. Rull-Barrull, M. d´Halluin, E. Le Grognec, and F.-X. Felpin, Harnessing the Dual Properties of Thiol-Grafted Cellulose Paper for Click Reactions: A Powerful Reducing Agent and Adsorbent for Cu, *Angew. Chem. Int. Ed.*, **55**, 13549–13552 (2016).

39. J.-S. Poh, D. N. Tran, C. Battilocchio, J. M. Hawkins and S. V. Ley, A Versatile Room-Temperature Route to Di- and Trisubstituted Allenes Using Flow-Generated Diazo Compounds, *Angew. Chem*., **127**, 8031–8034 (2015).

40. X. Li, M. D. Wodrich and J. Waser, Accessing elusive σ-type cyclopropenium cation equivalents through redox gold catalysis, *Nature Chemistry* **16**, 901–912 (2024).

41. A. Mishra, B. B. Mishra, V. K. Tiwari, Regioselective facile synthesis of novel isoxazole-linked glycoconjugates, *RSC Adv.*, **5**, 41520–41535 (2015).

42. D. K. R. Vennam, R. K. Thatipamula, S. Babu Haridasyam, S. K. Koppula, Design, synthesis, and biological evaluation of new 1-aryl-4-(β-D-fructopyranos-3-O-yl)methyl-1H-1,2,3-triazole derivatives, Chemistry of Heterocyclic Compounds, **54**, 630–637, (2018).

6. Copies of ^{1}H, ^{13}C and ^{31}P NMR

Copies of ^{1}H and ^{13}C NMR spectra of **4**

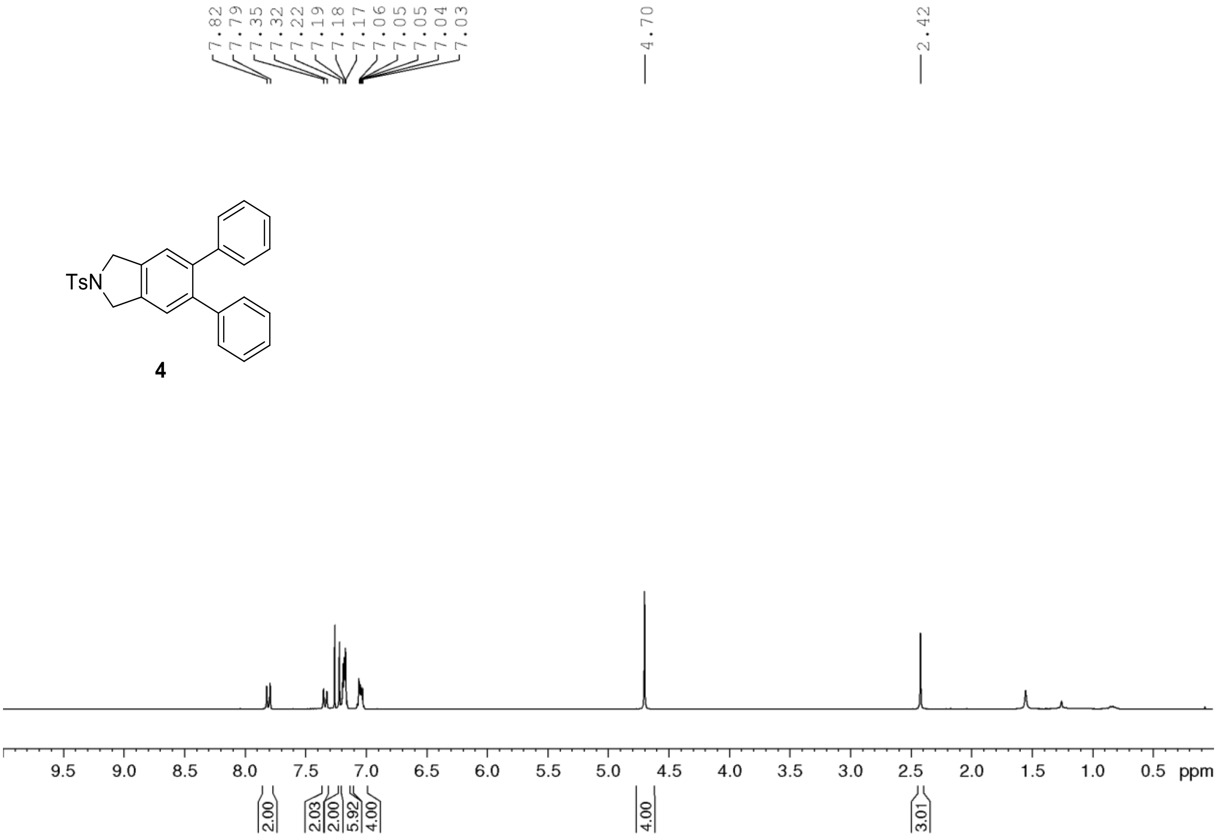

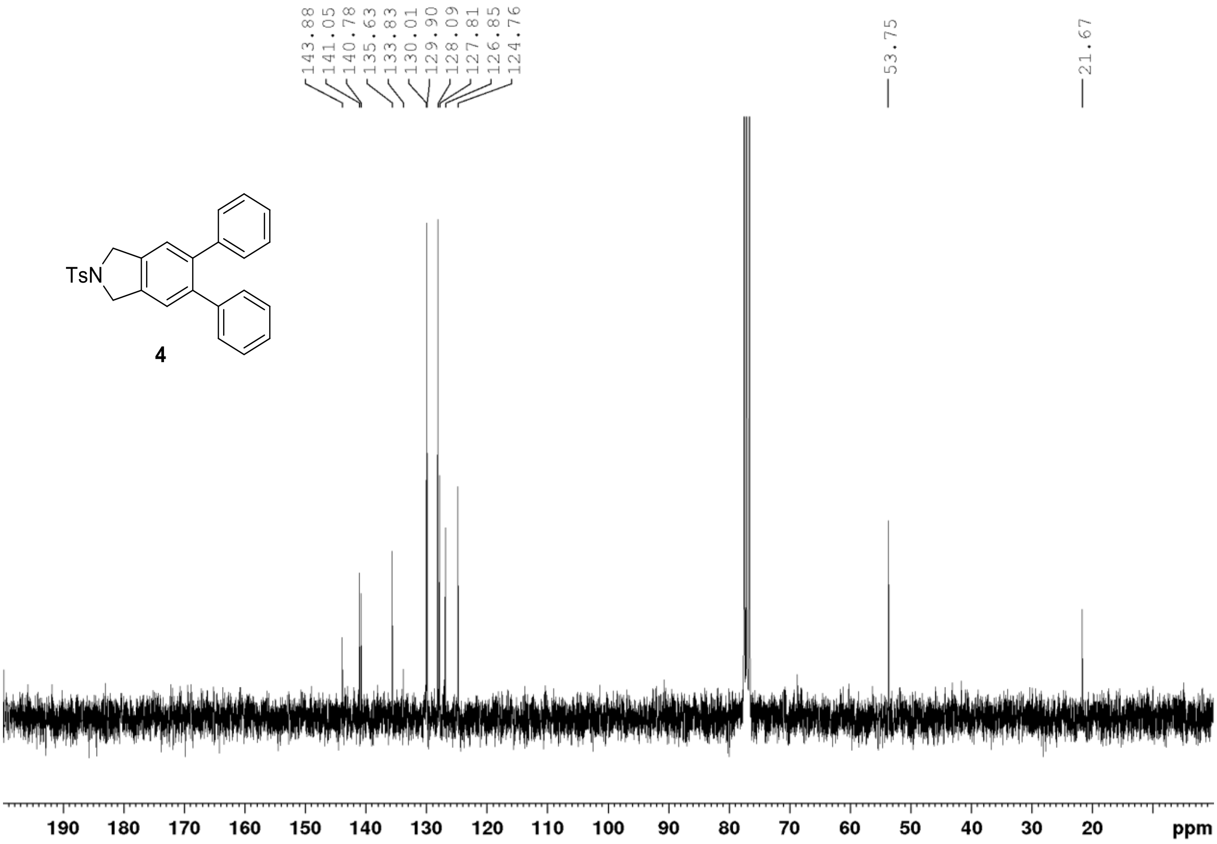

Copies of ^{1}H and ^{13}C NMR spectra of **7**

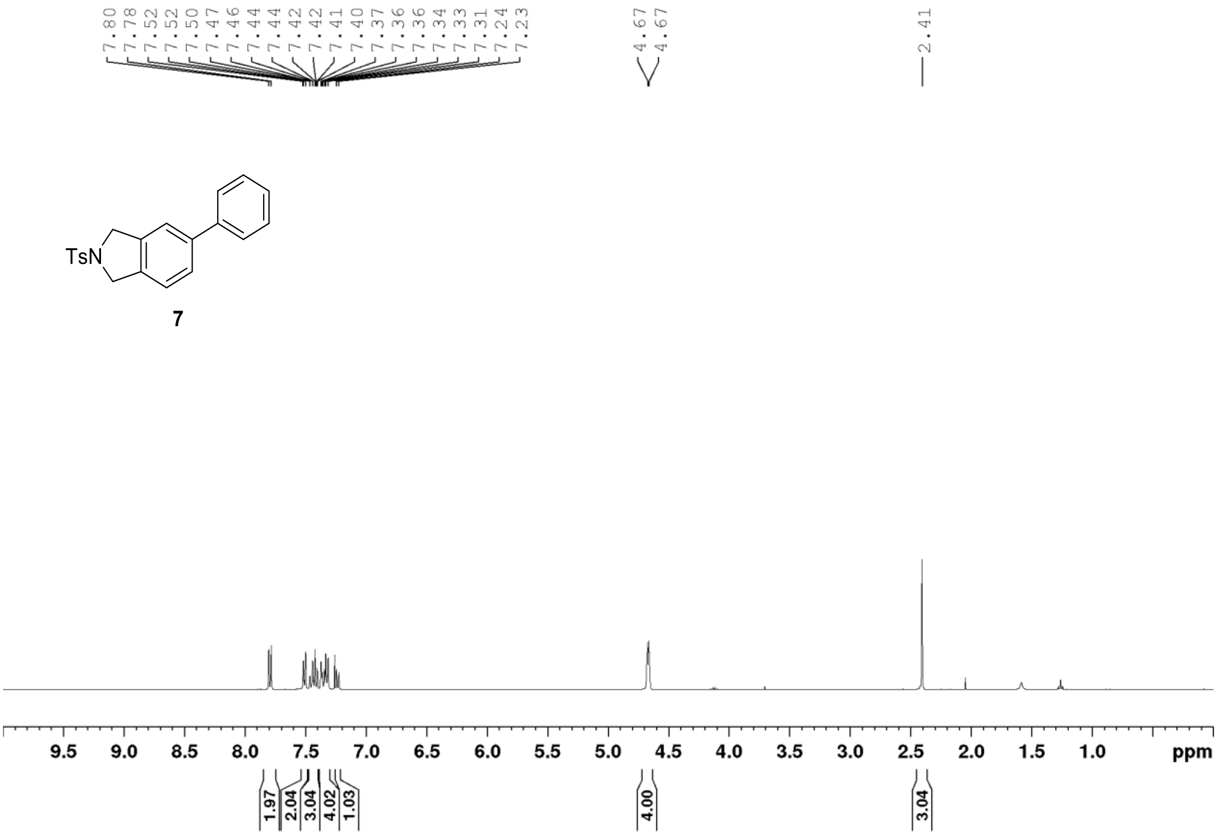

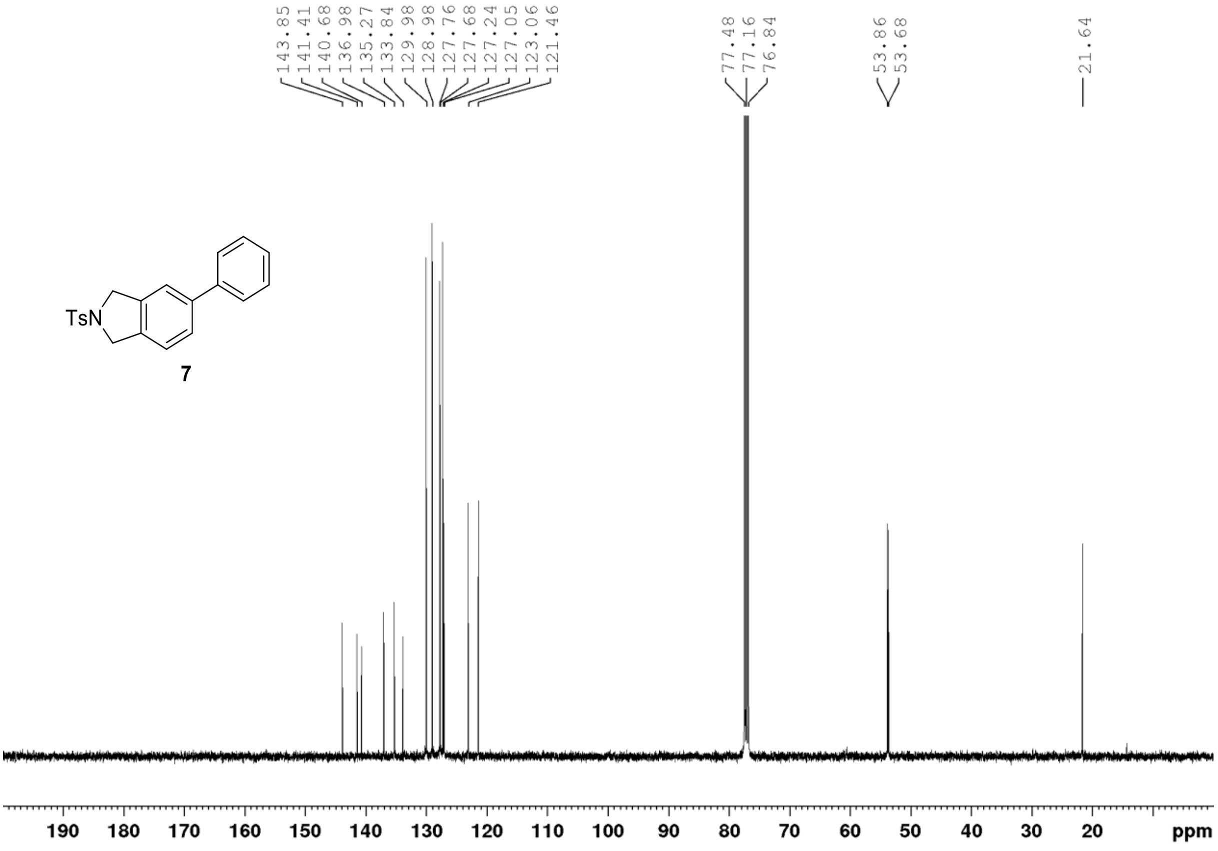

Copies of ^{1}H and ^{13}C NMR spectra of **8**

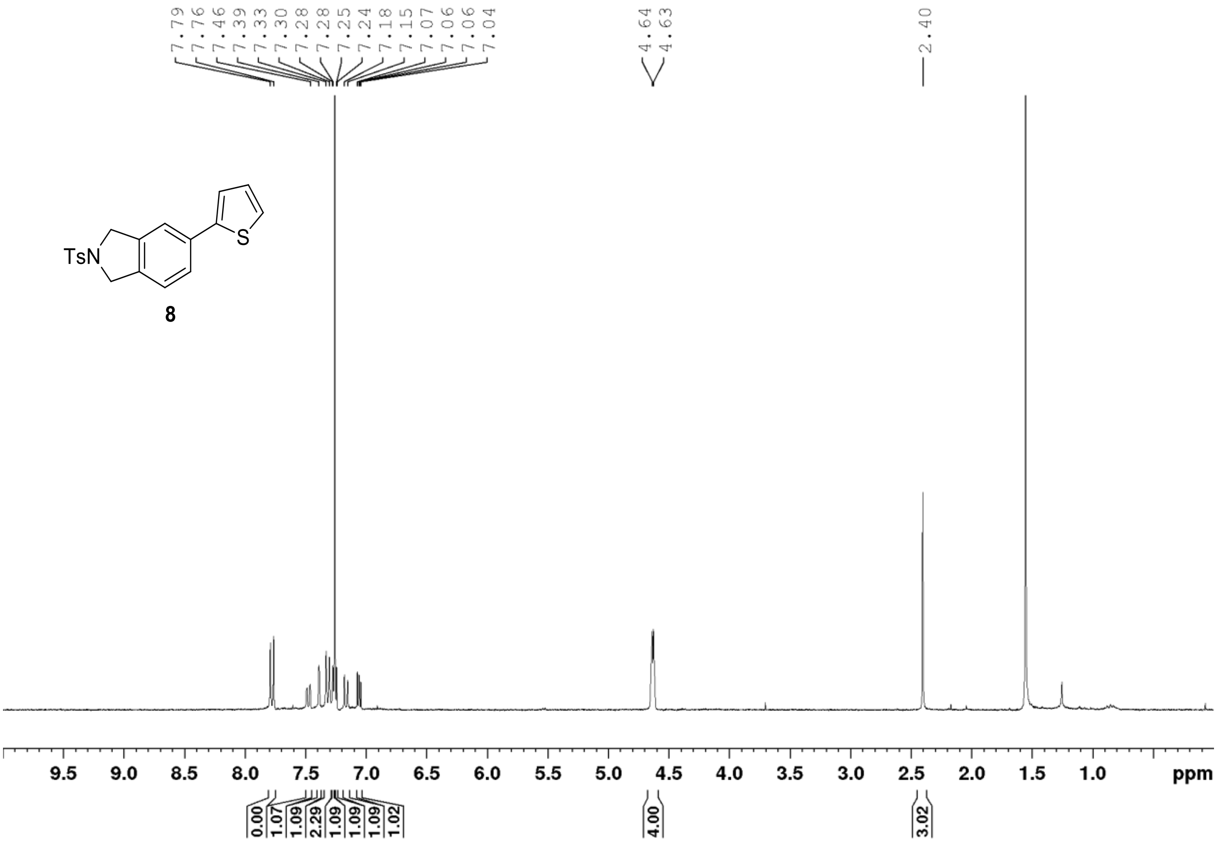

8

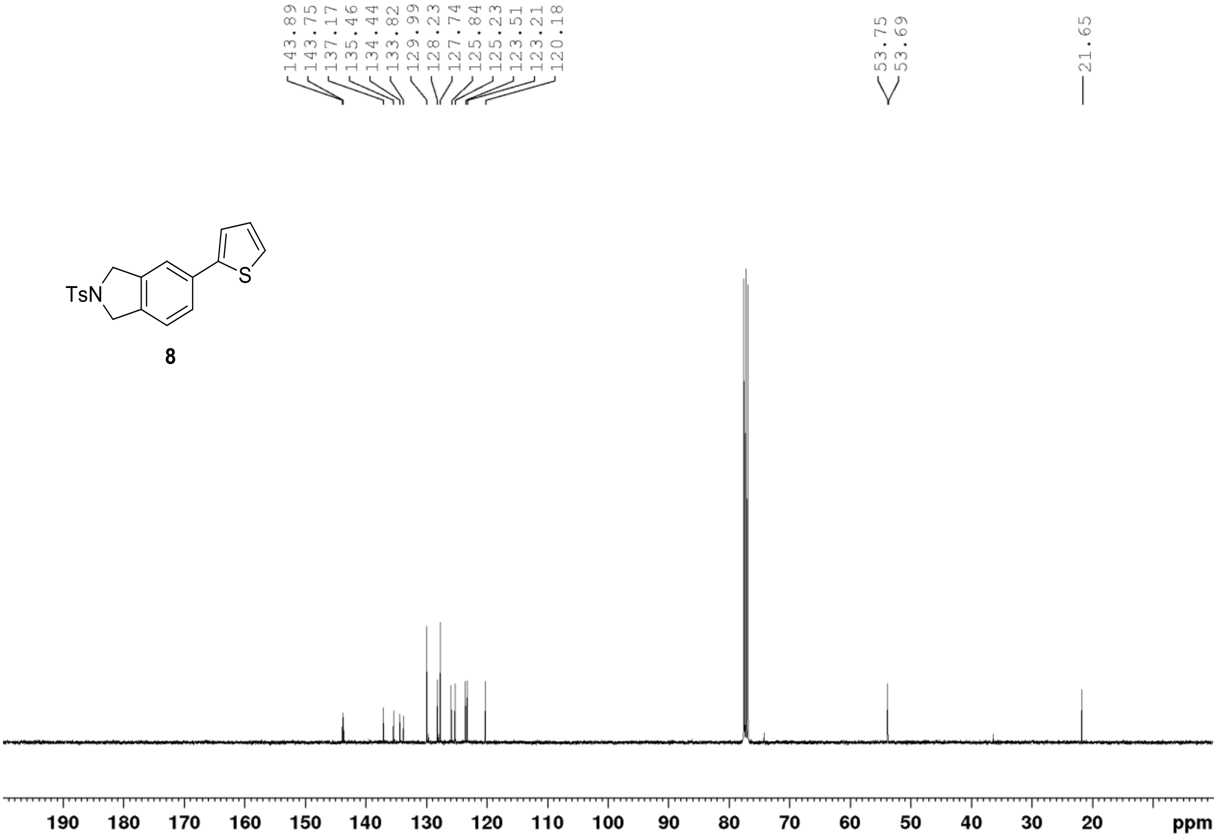

8

Copies of ^{1}H and ^{13}C NMR spectra of **9**

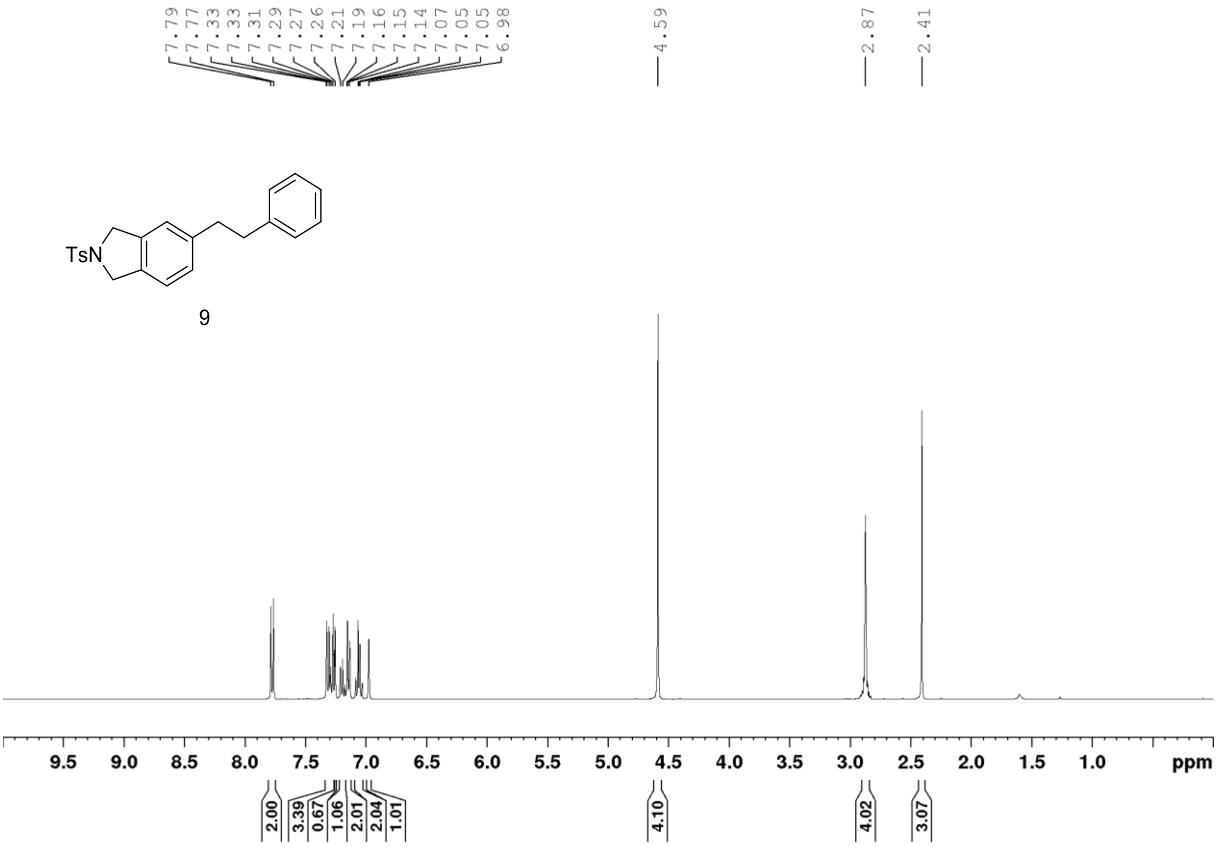

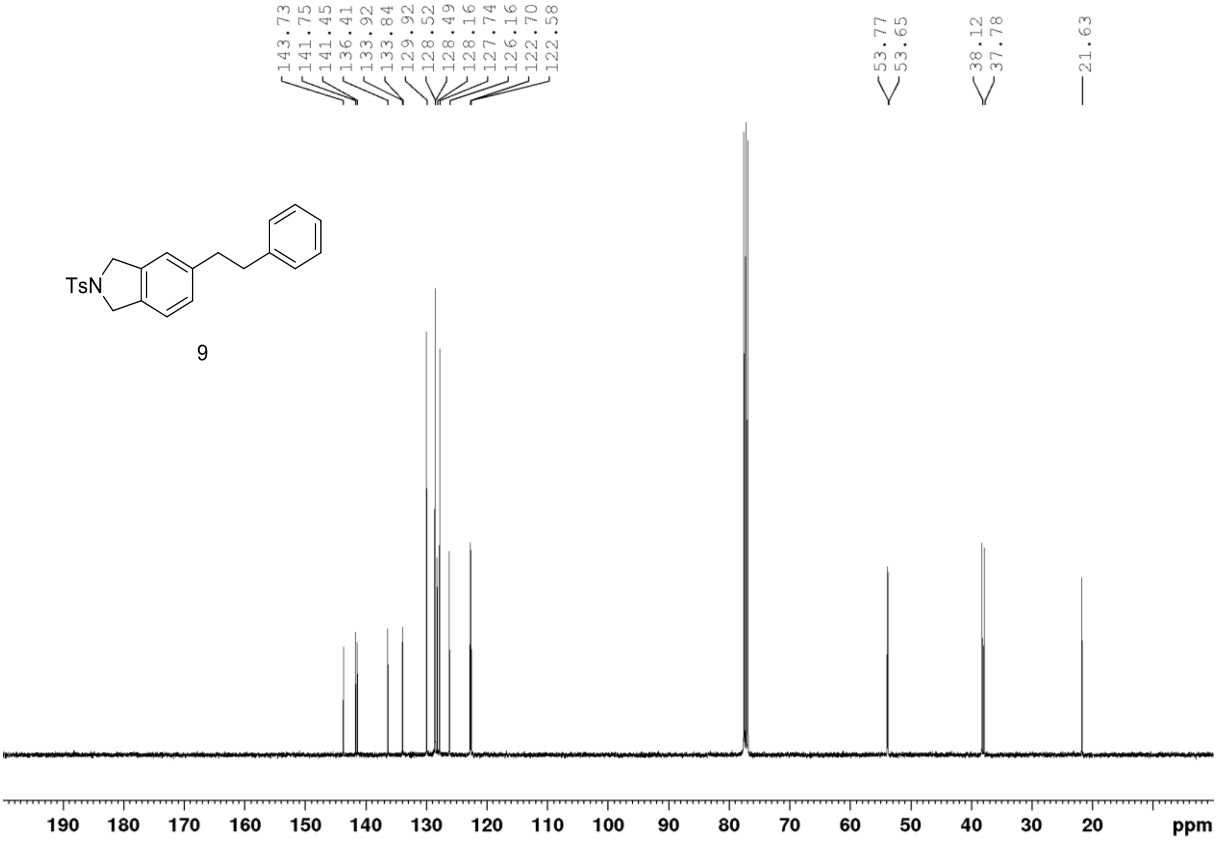

Copies of ^{1}H and ^{13}C NMR spectra of **10**

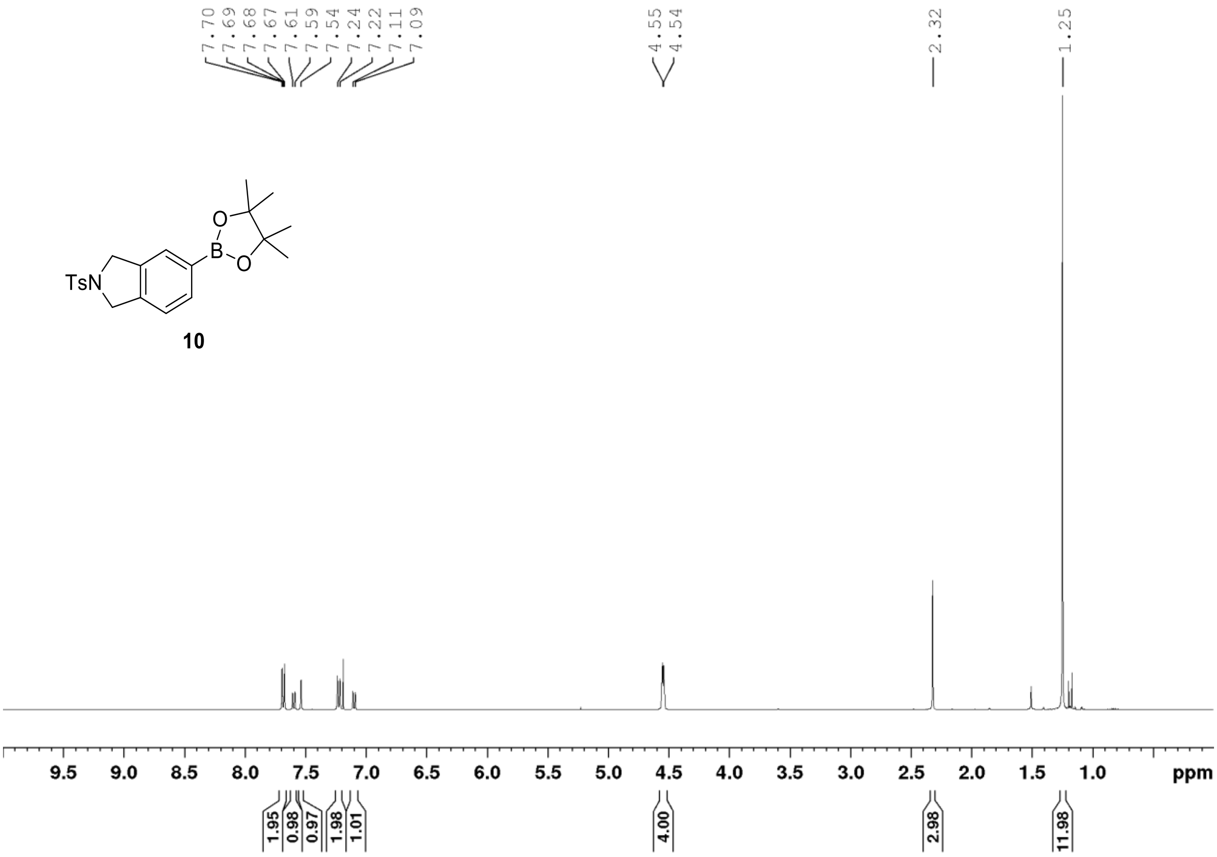

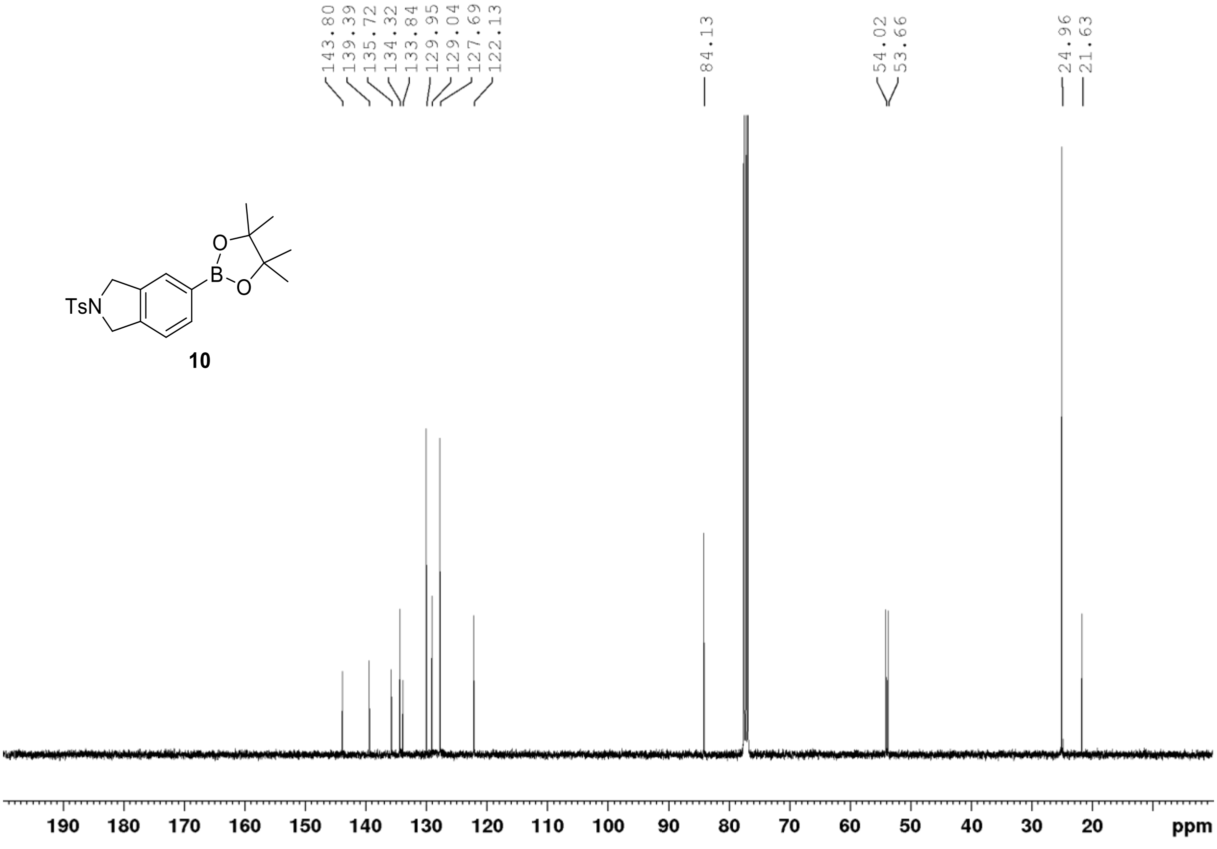

Copies of ^{1}H and ^{13}C NMR spectra of **11**

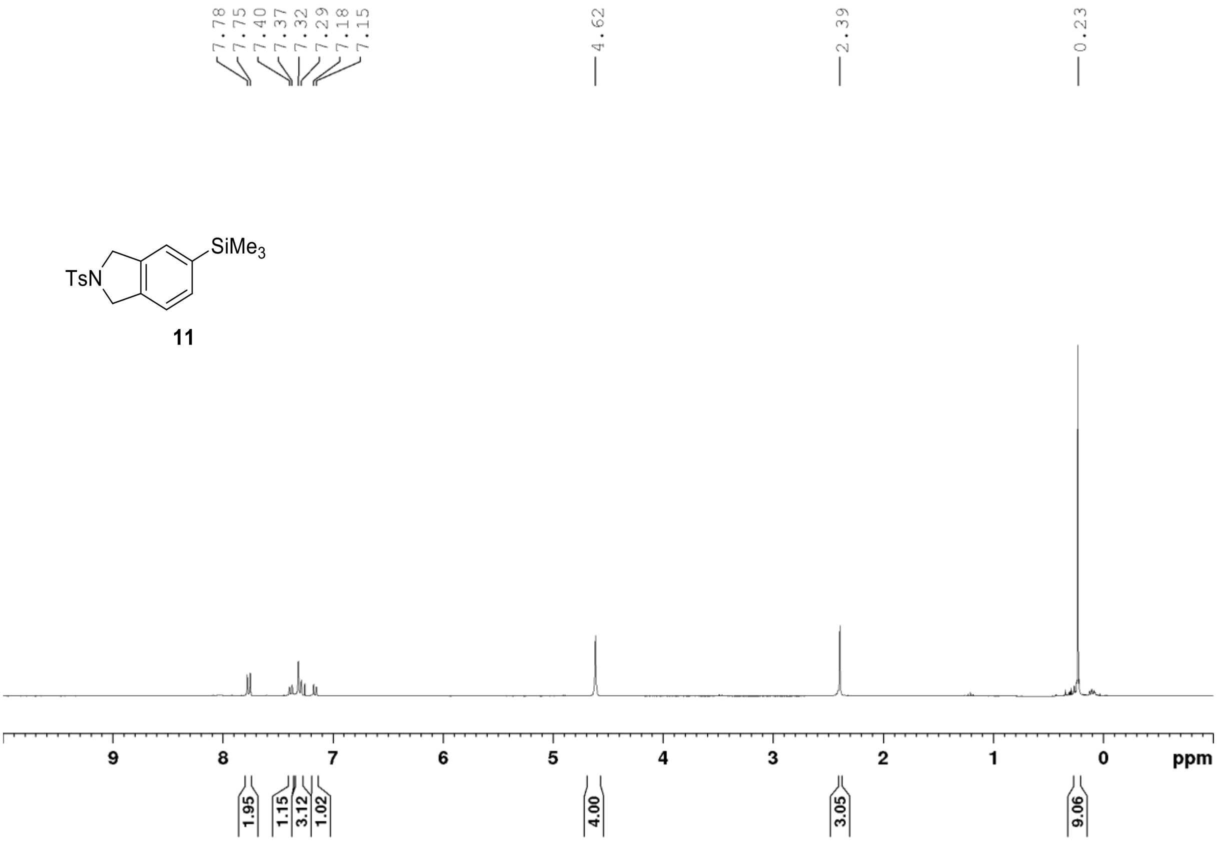

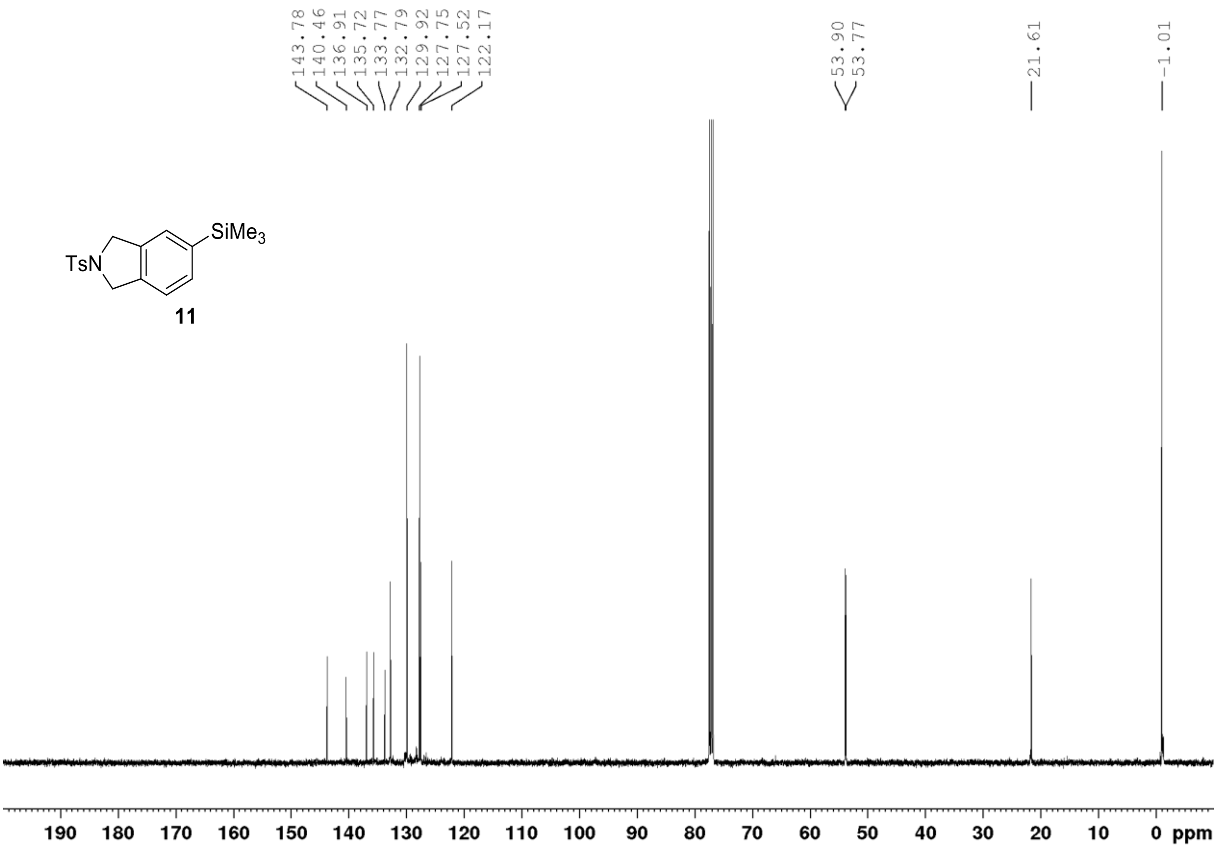

Copies of ^{1}H and ^{13}C NMR spectra of **12**

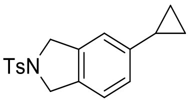

12

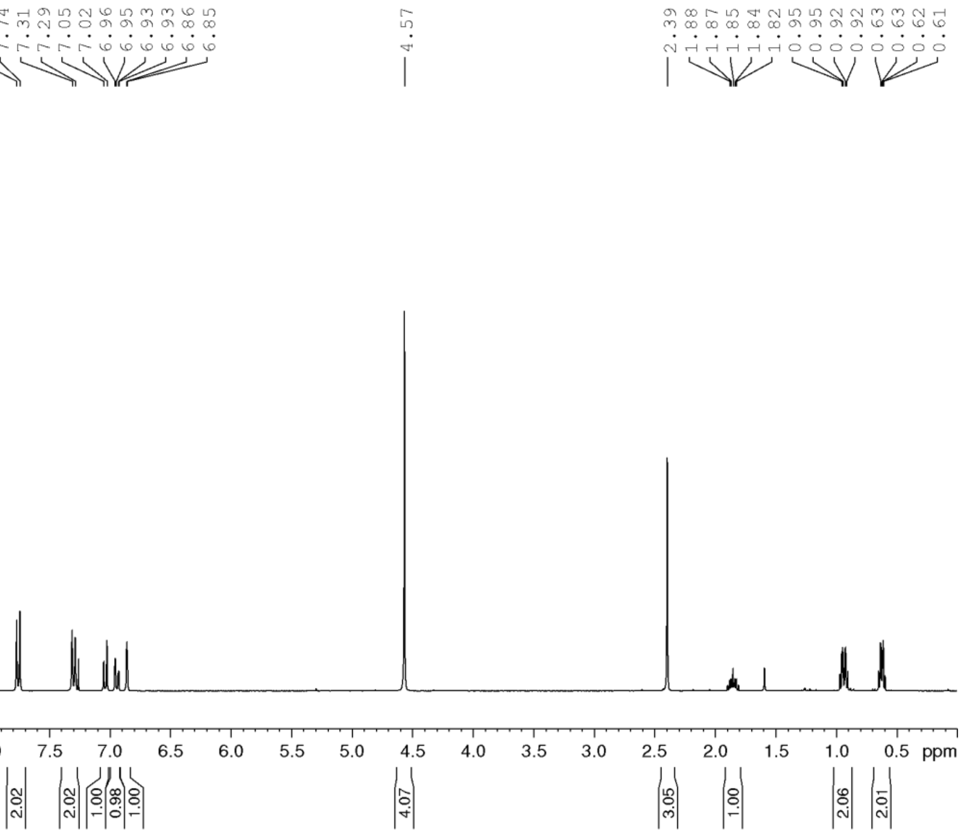

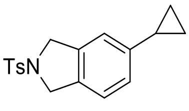

12

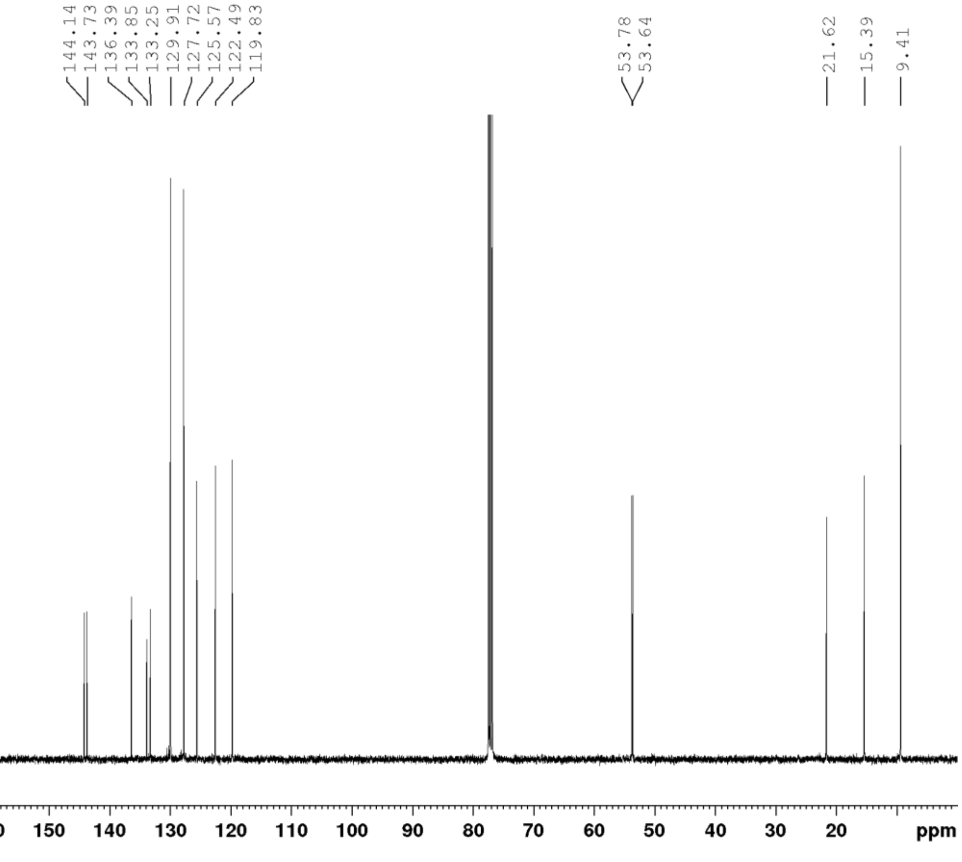

Copies of ^{1}H and ^{13}C NMR spectra of **13**

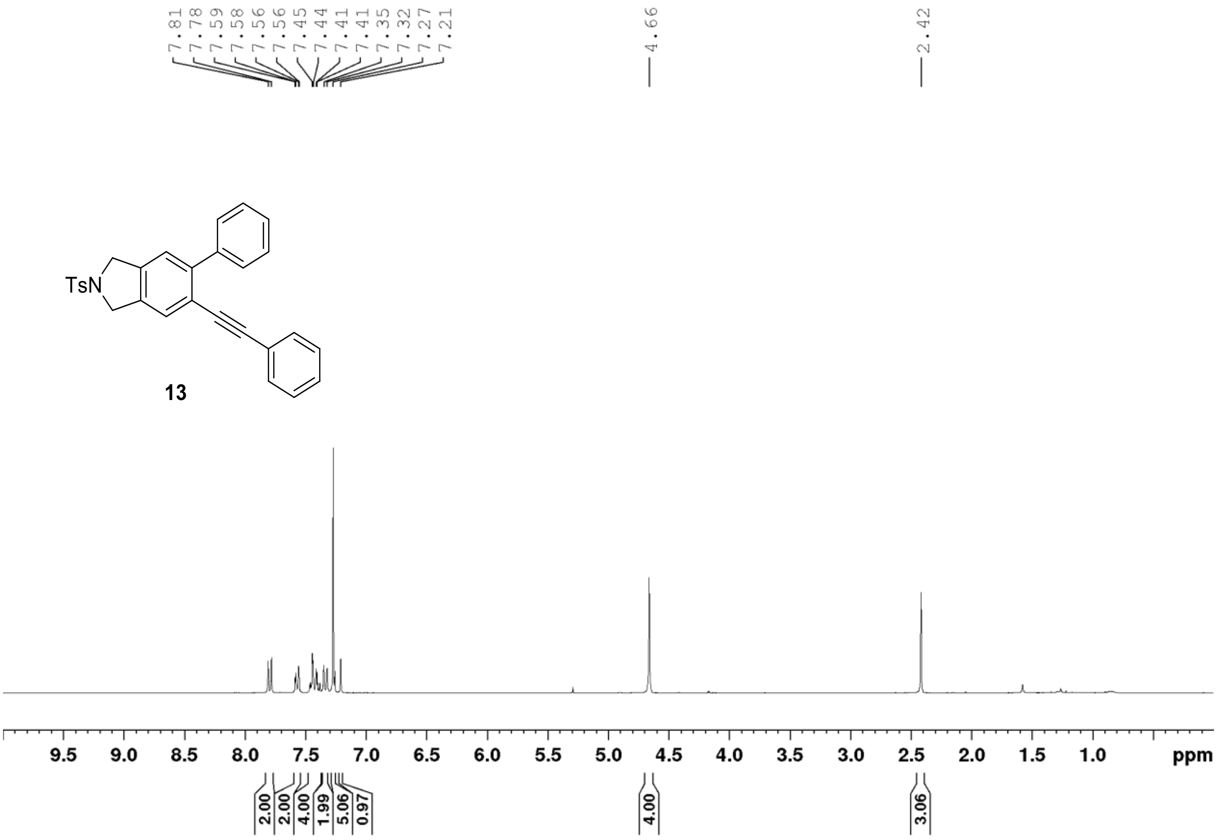

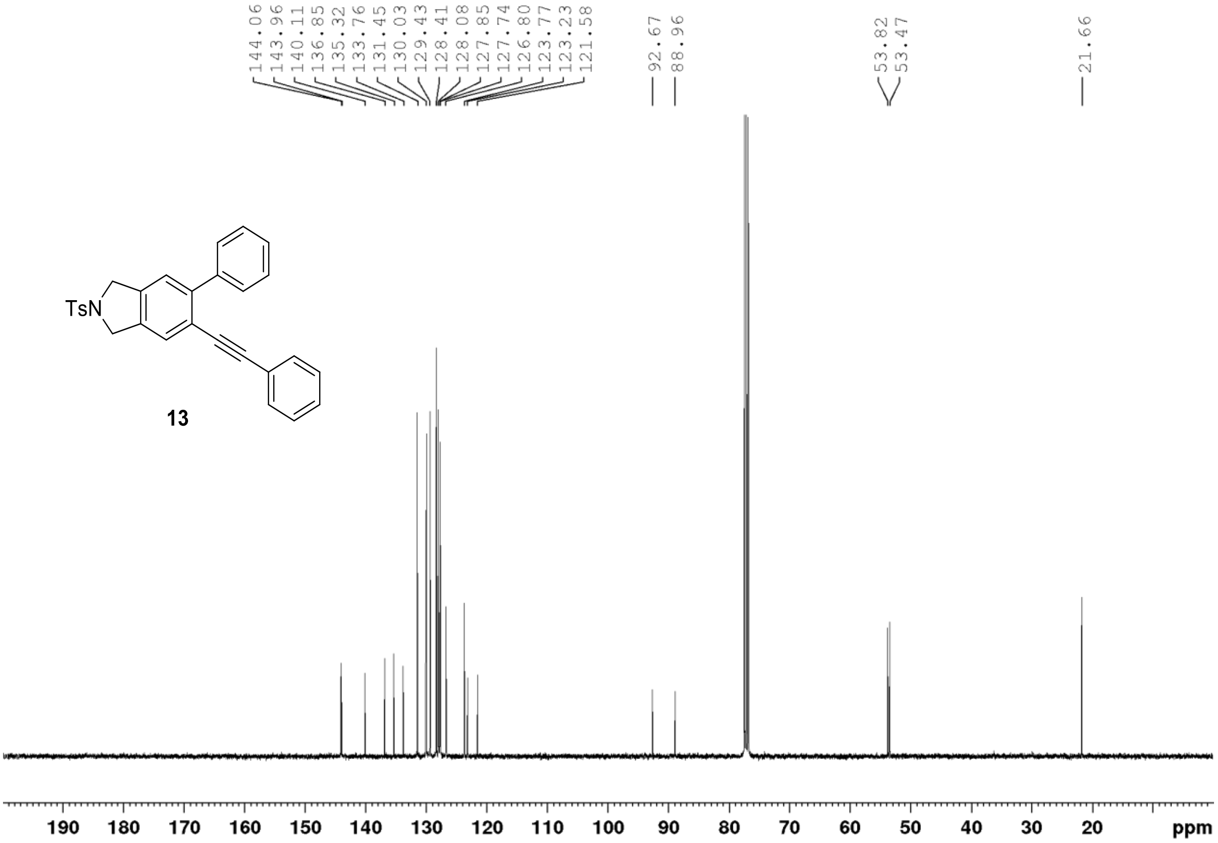

Copies of ^{1}H and ^{13}C NMR spectra of **14**

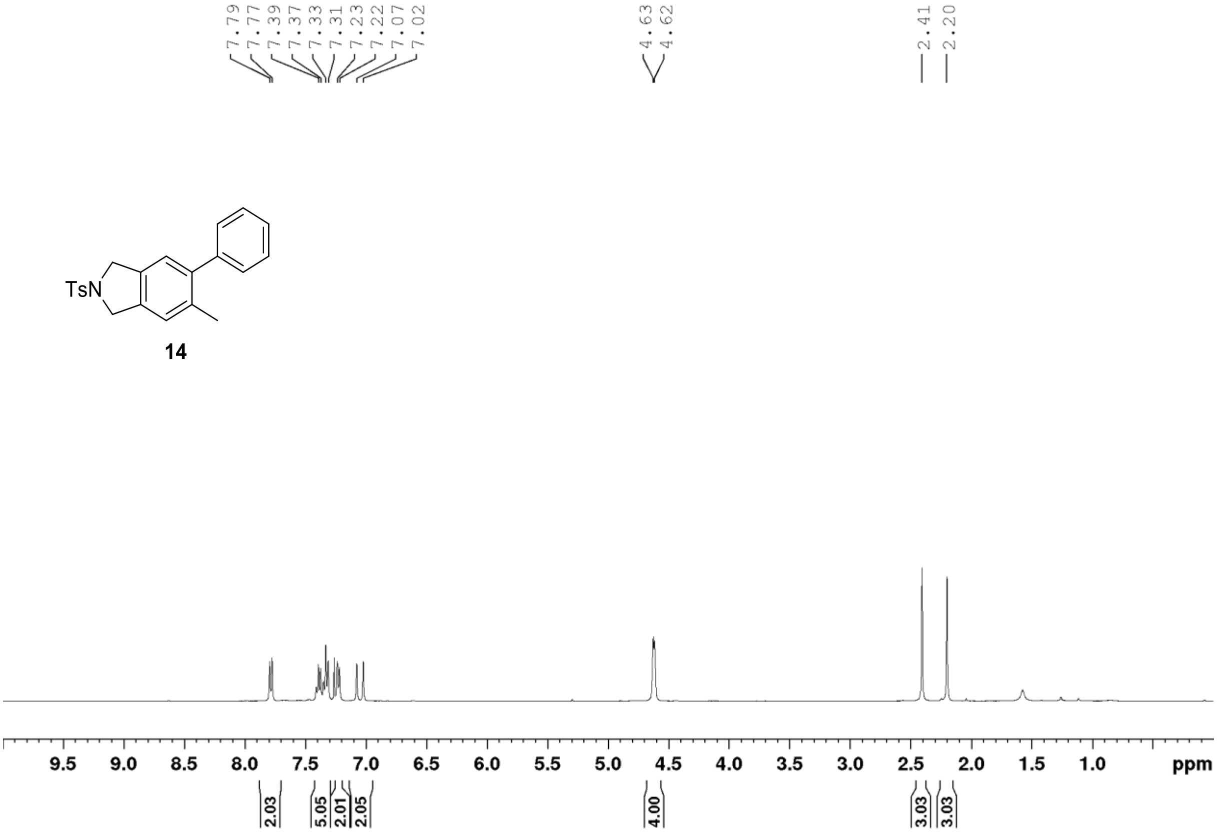

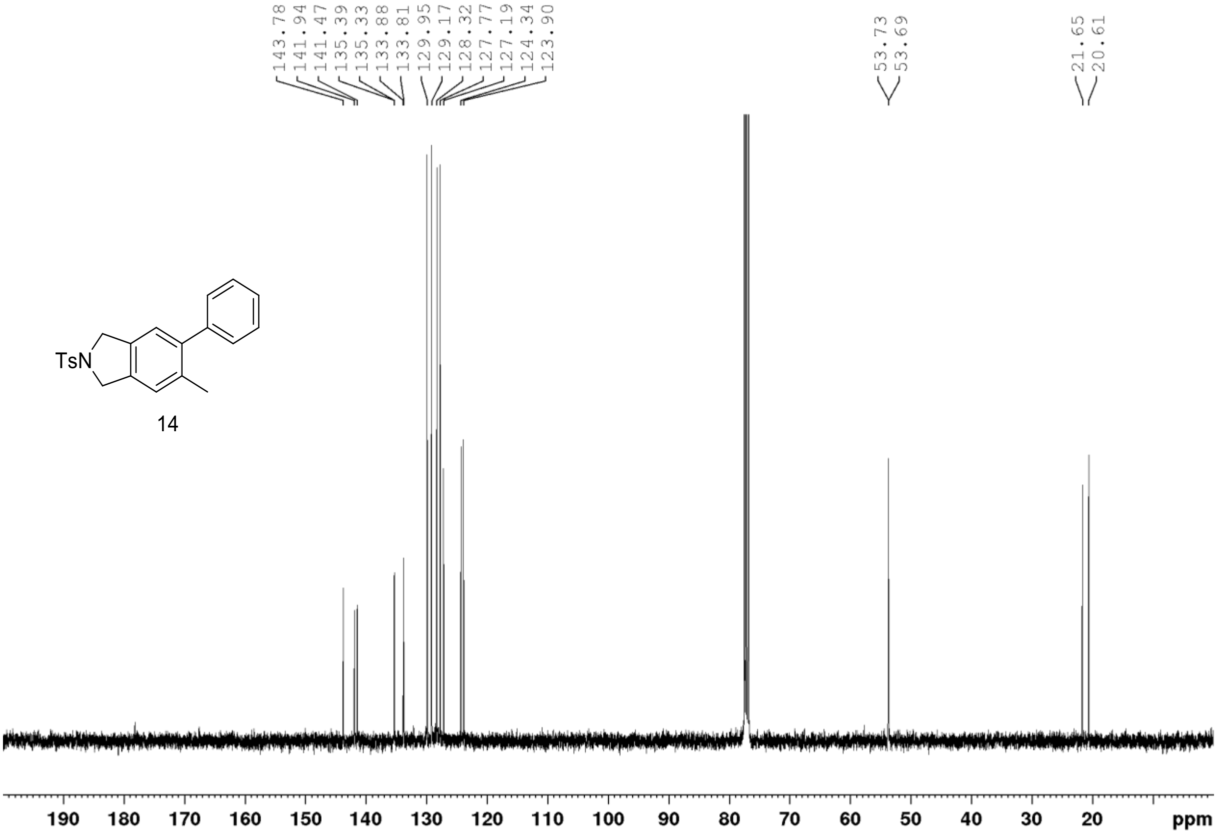

Copies of ^{1}H and ^{13}C NMR spectra of **15**

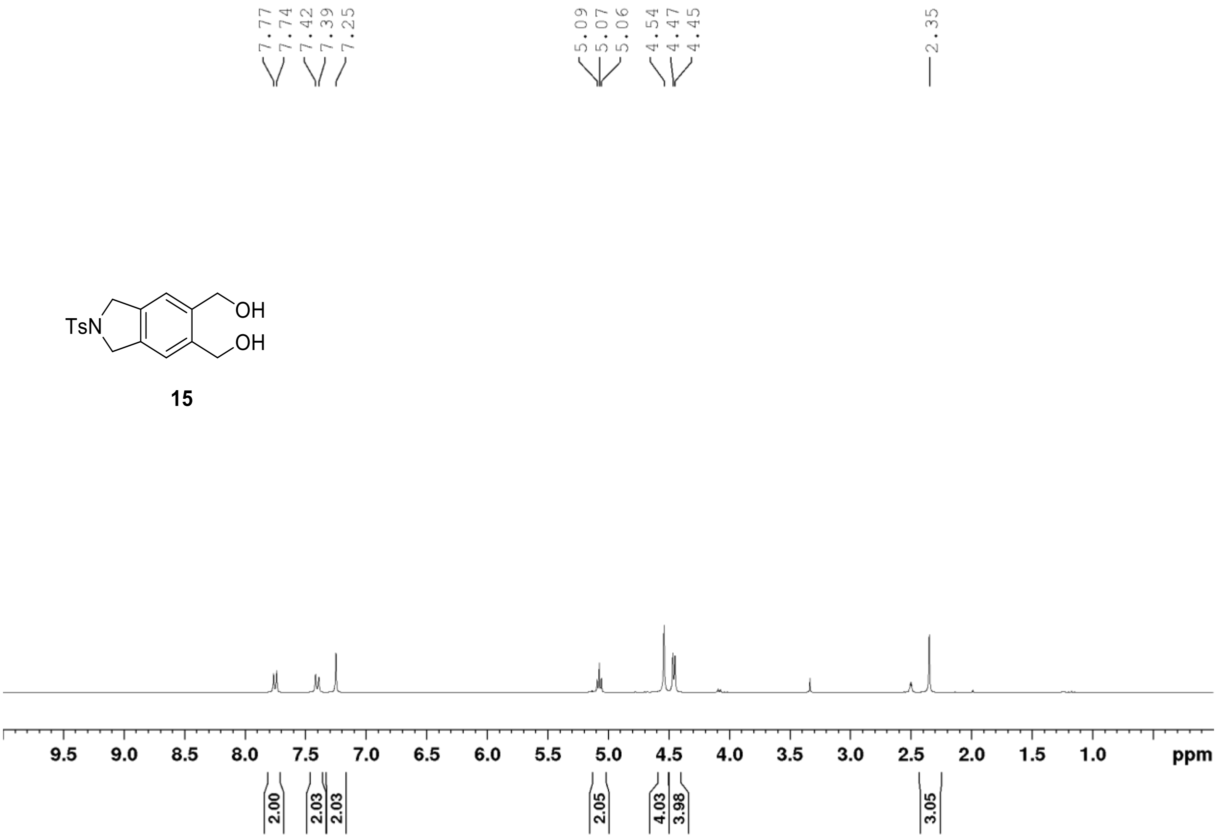

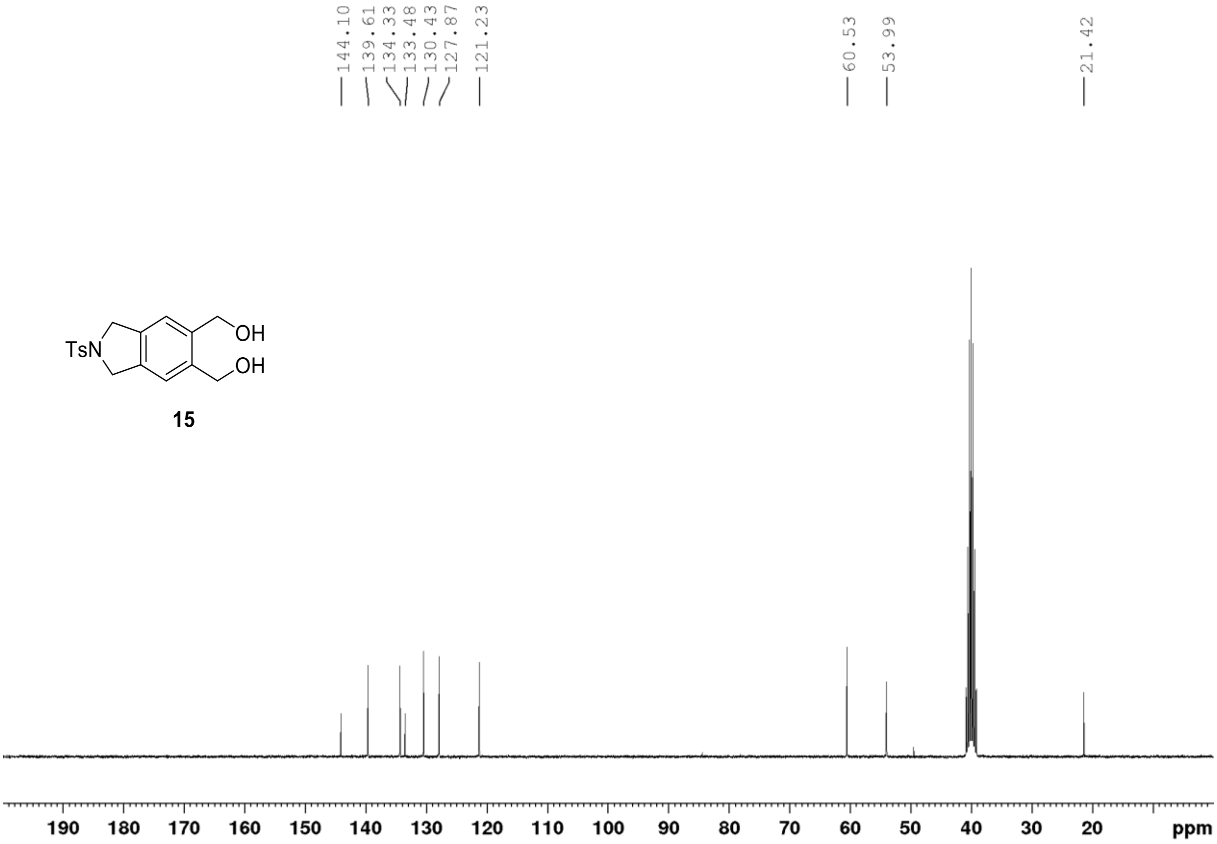

Copies of ^{1}H and ^{13}C NMR spectra of **16**

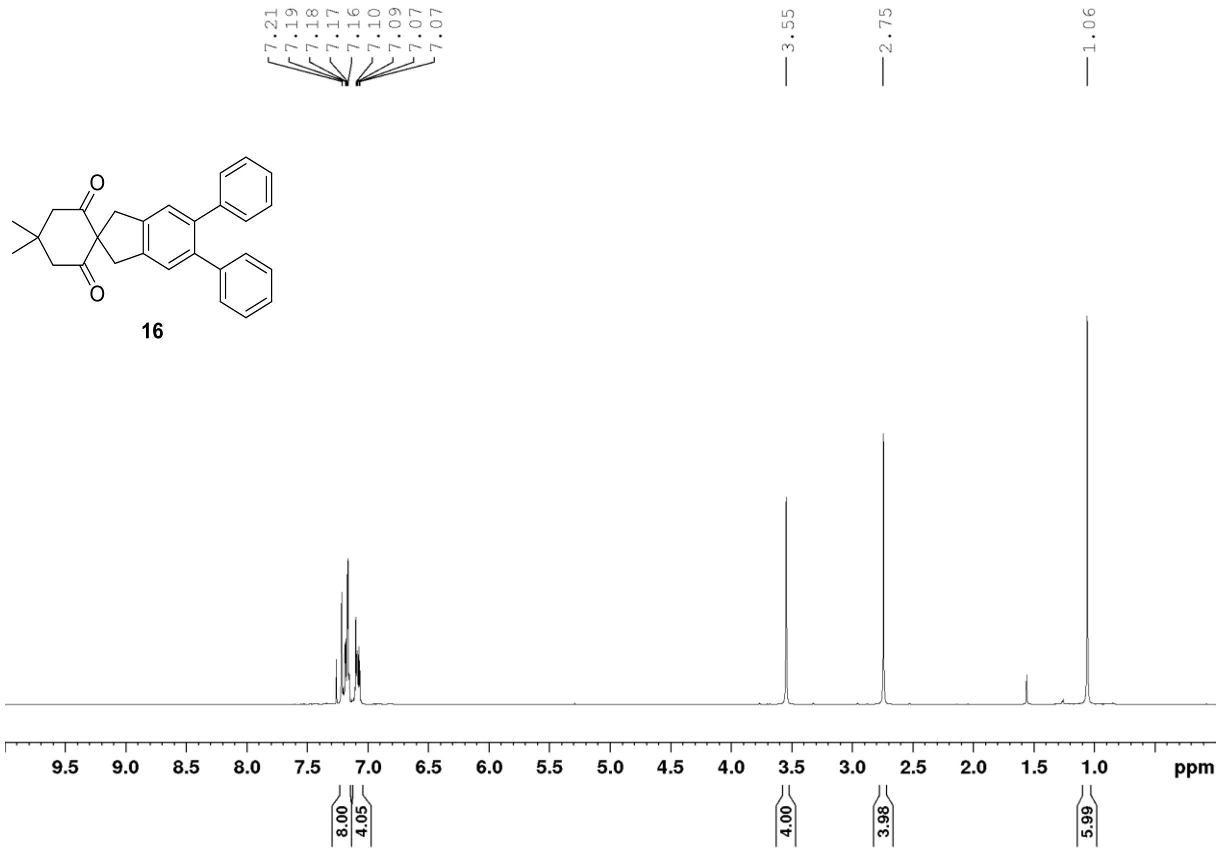

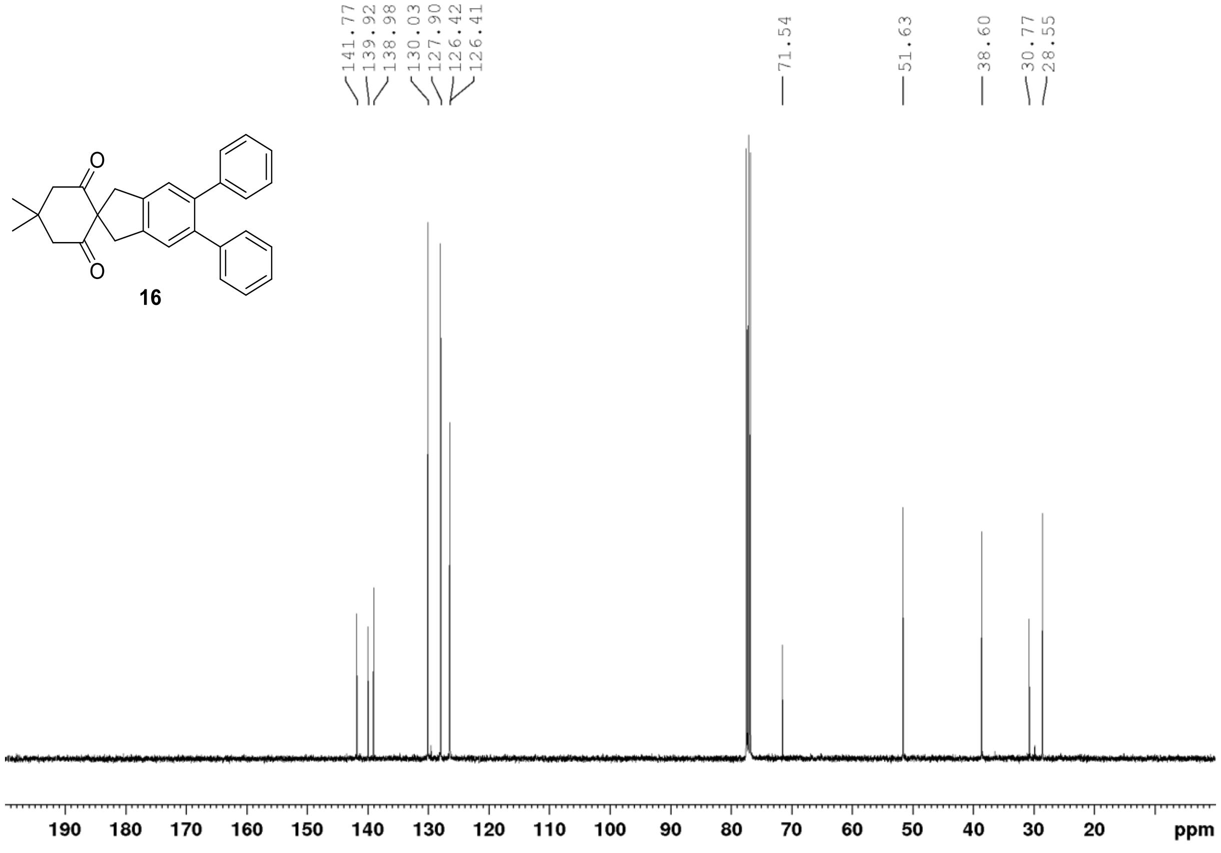

Copies of ^{1}H and ^{13}C NMR spectra of **17**

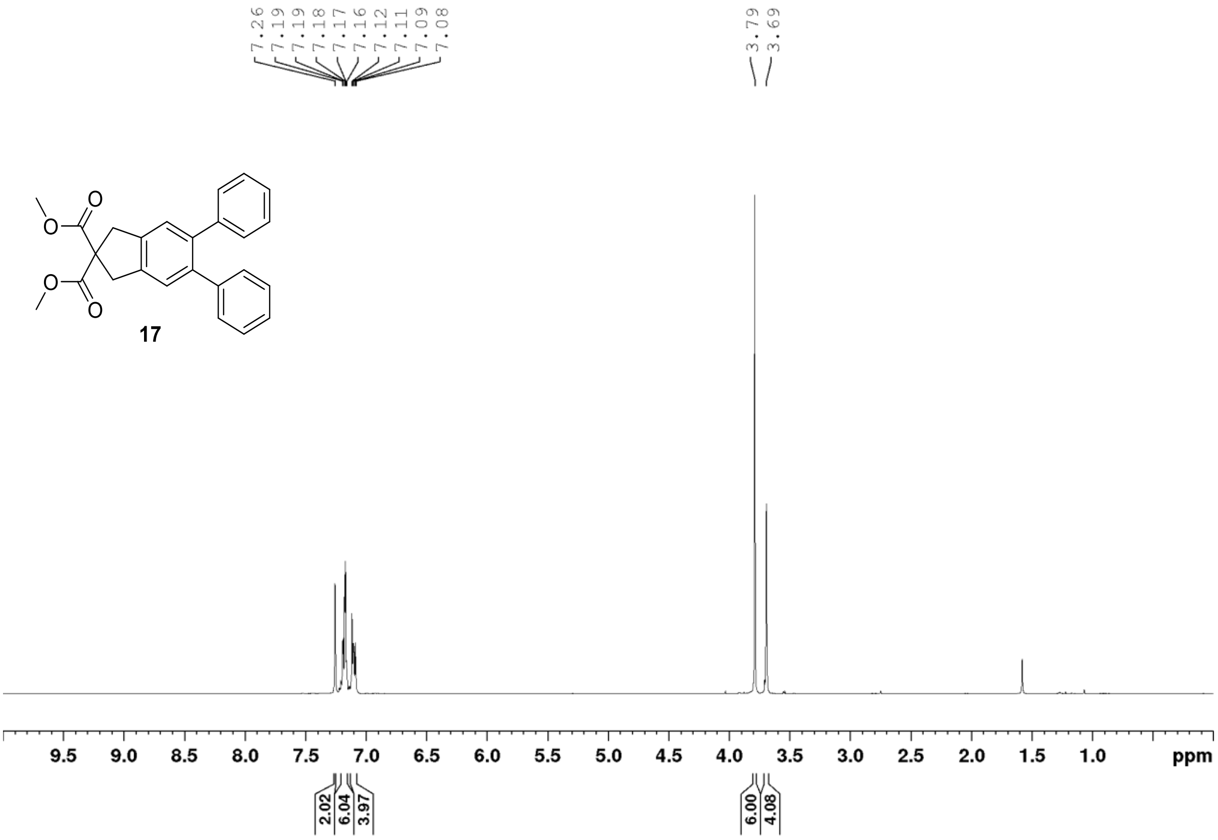

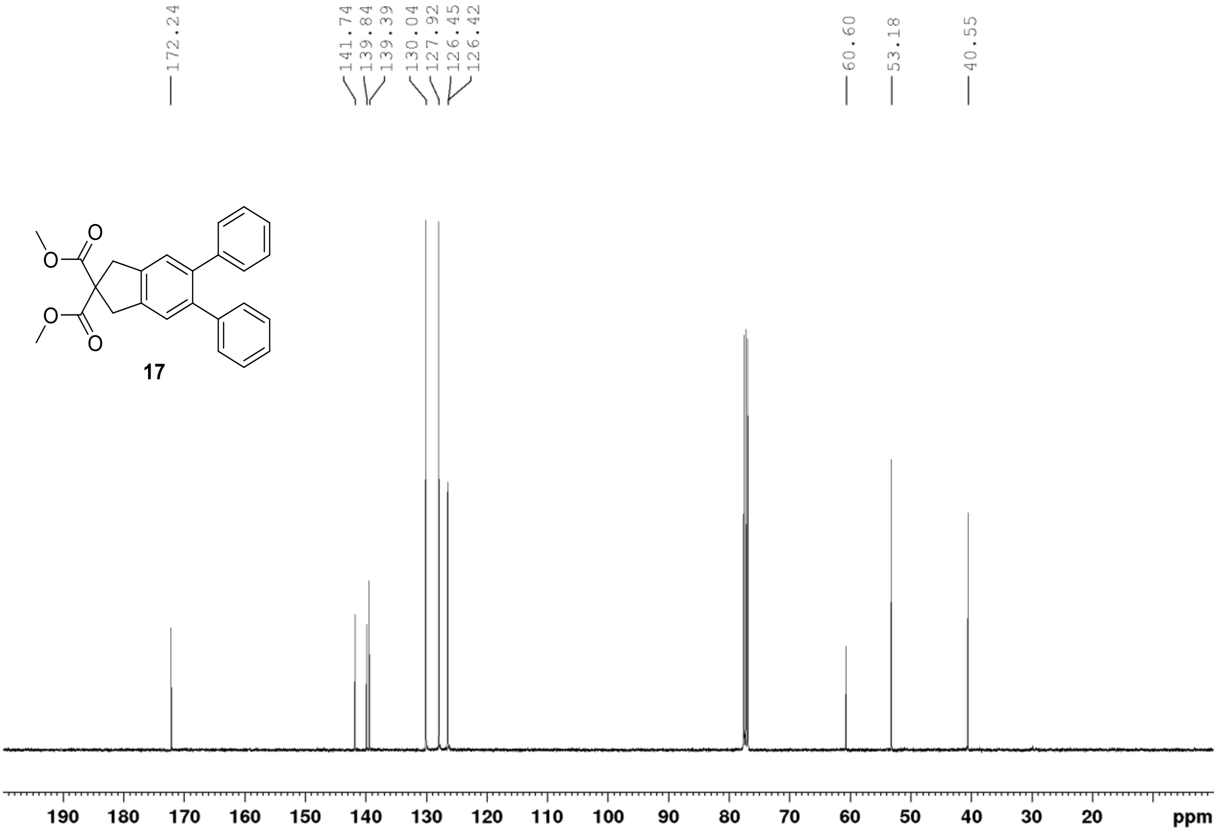

Copies of ^{1}H and ^{13}C NMR spectra of **18** diastereomer 1

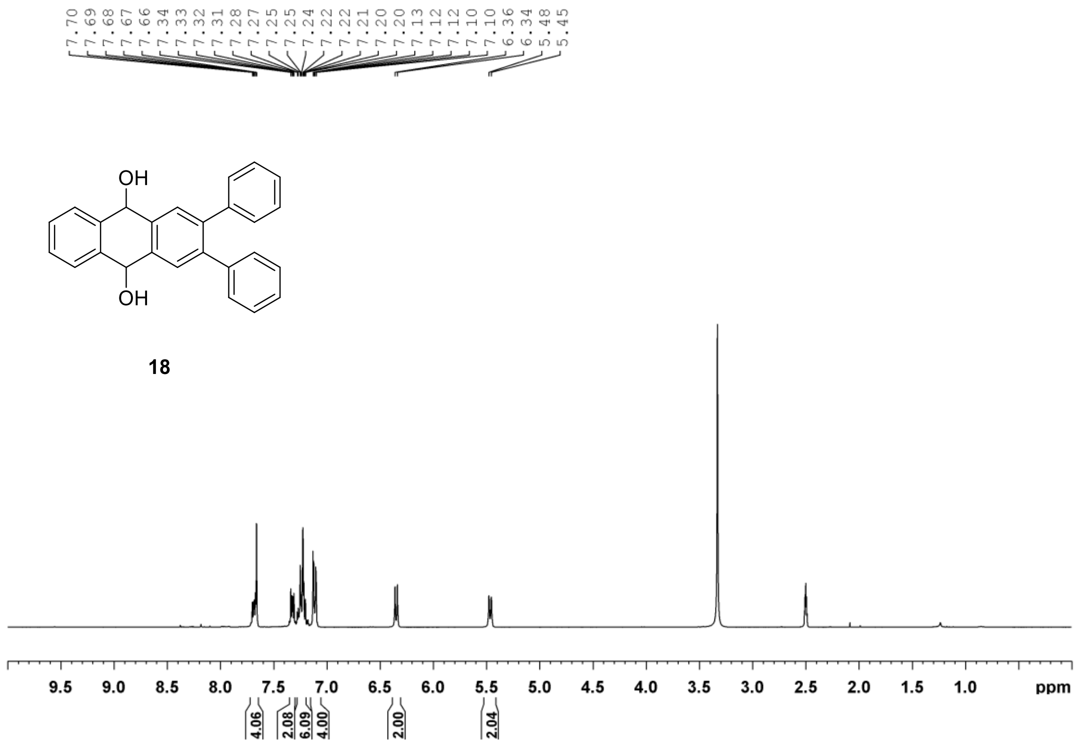

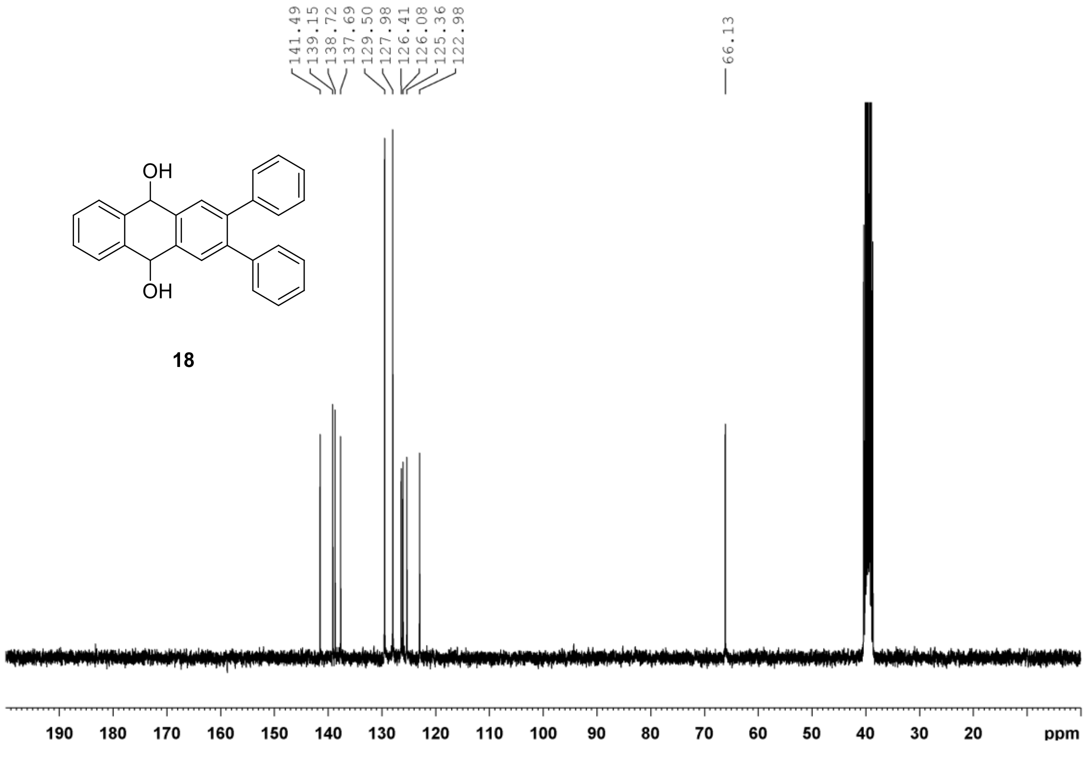

Copies of ^{1}H and ^{13}C NMR spectra of **18** diastereomer 2

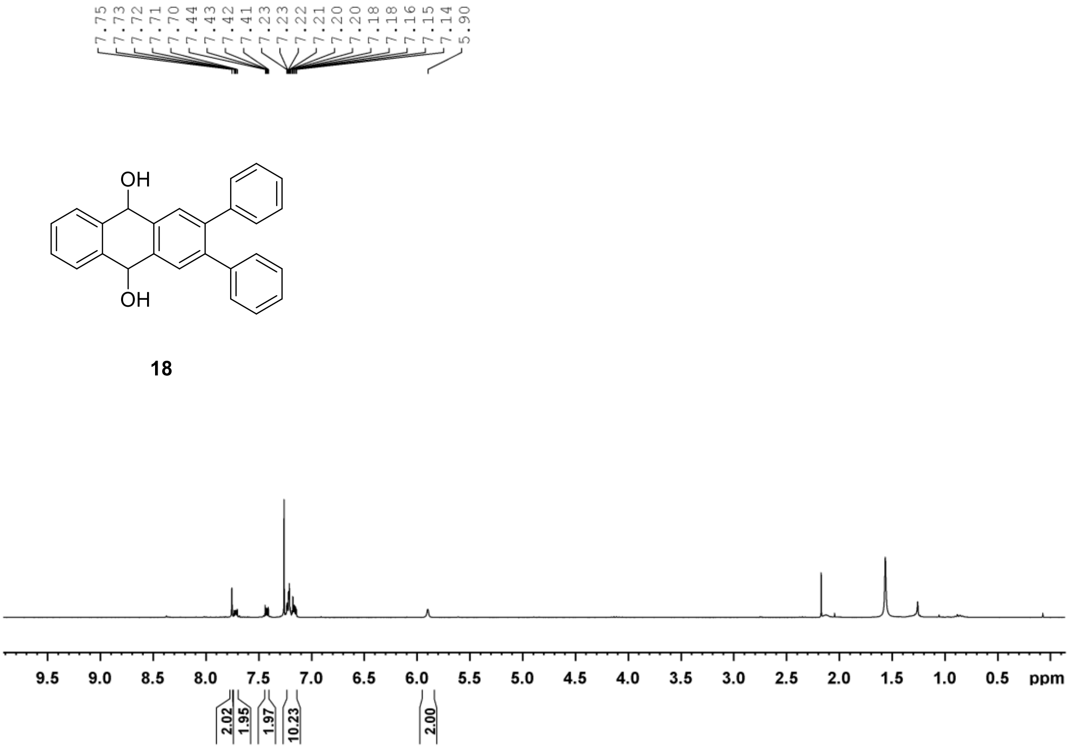

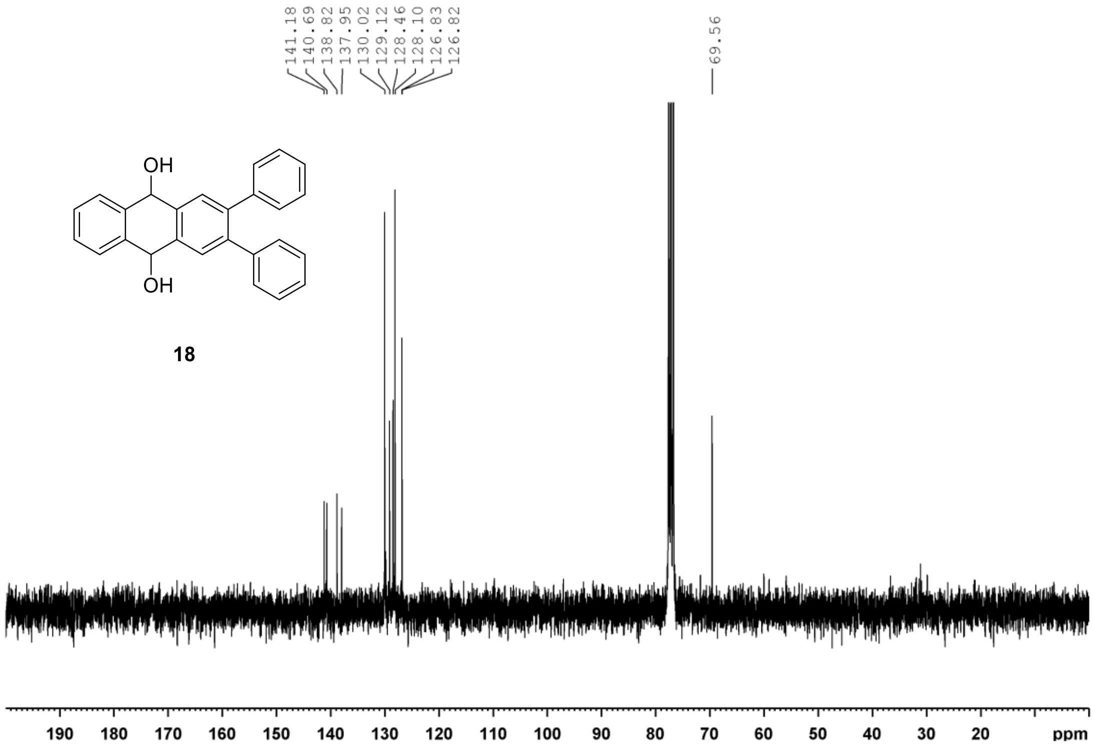

Copies of ^{1}H and ^{13}C NMR spectra of **19**

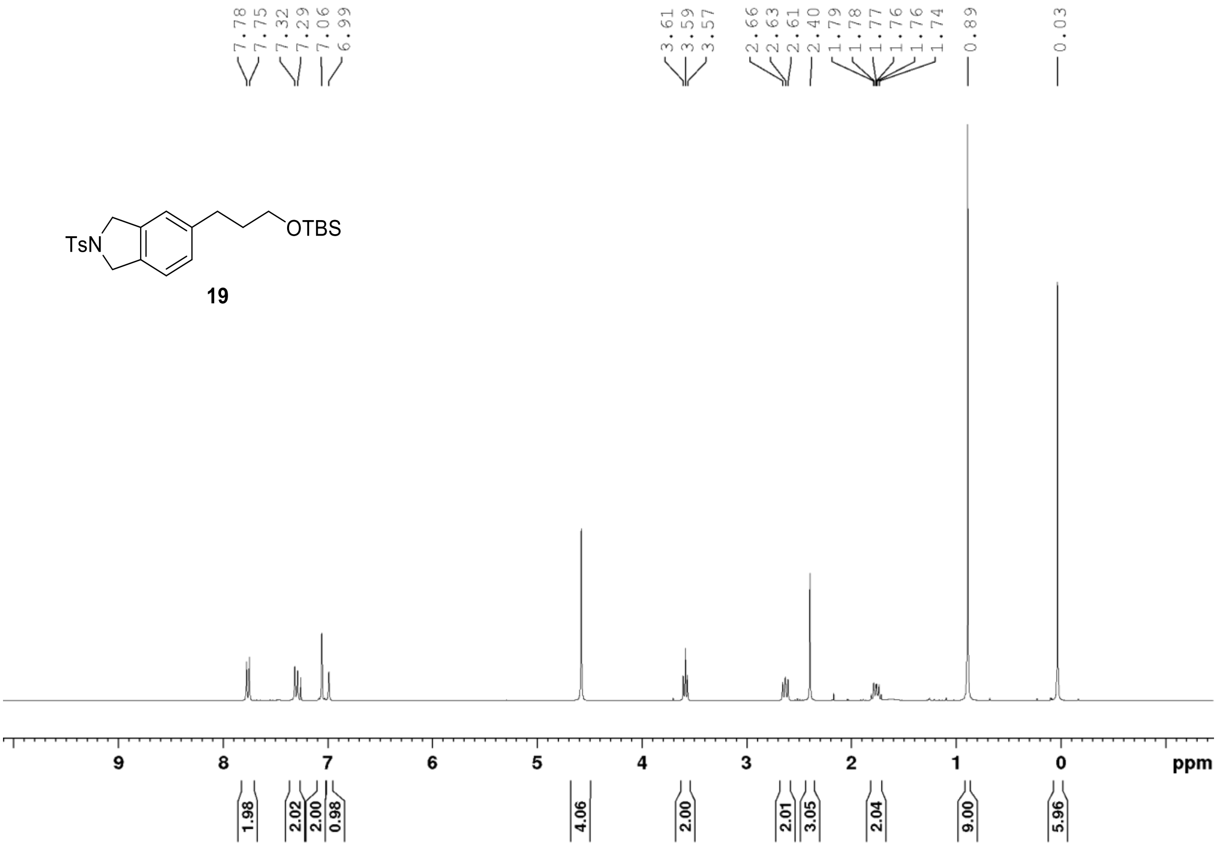

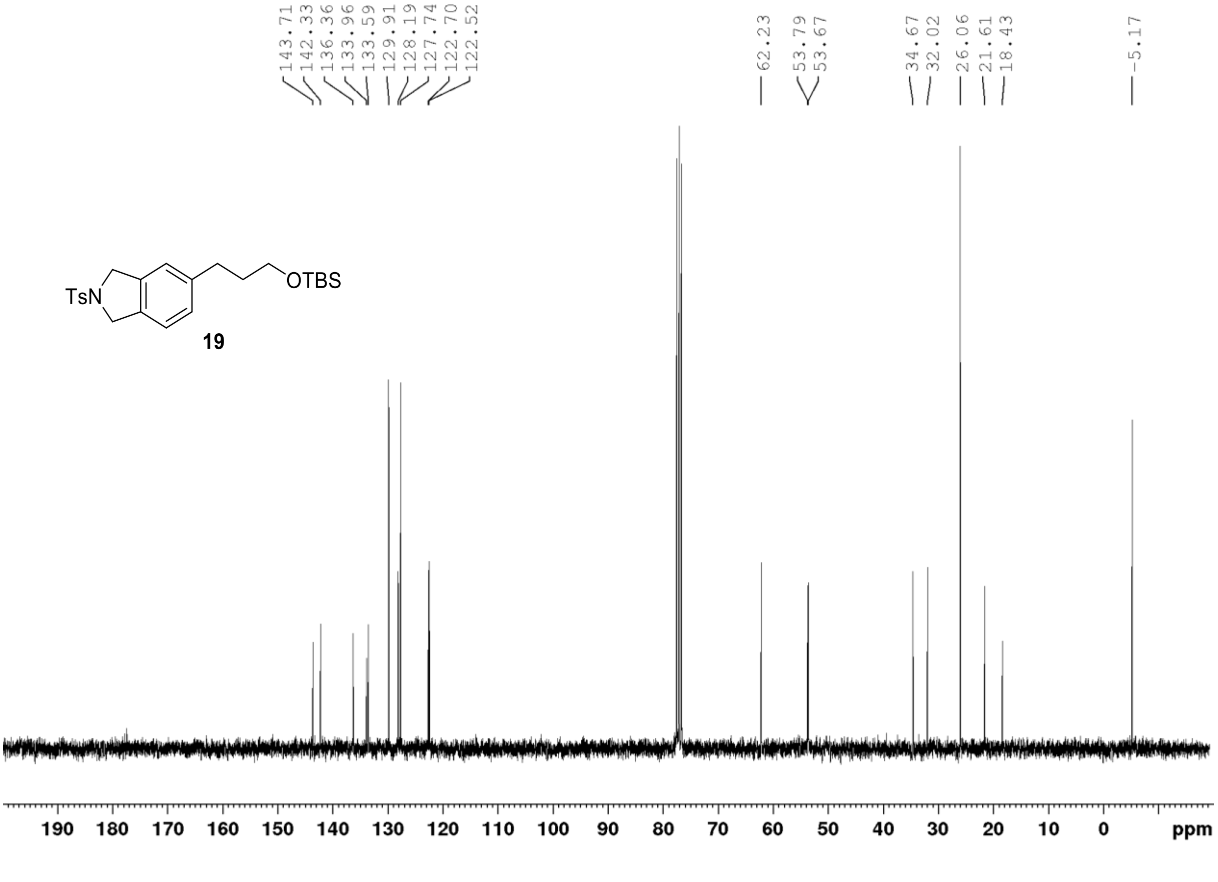

Copies of ^{1}H and ^{13}C NMR spectra of **20**

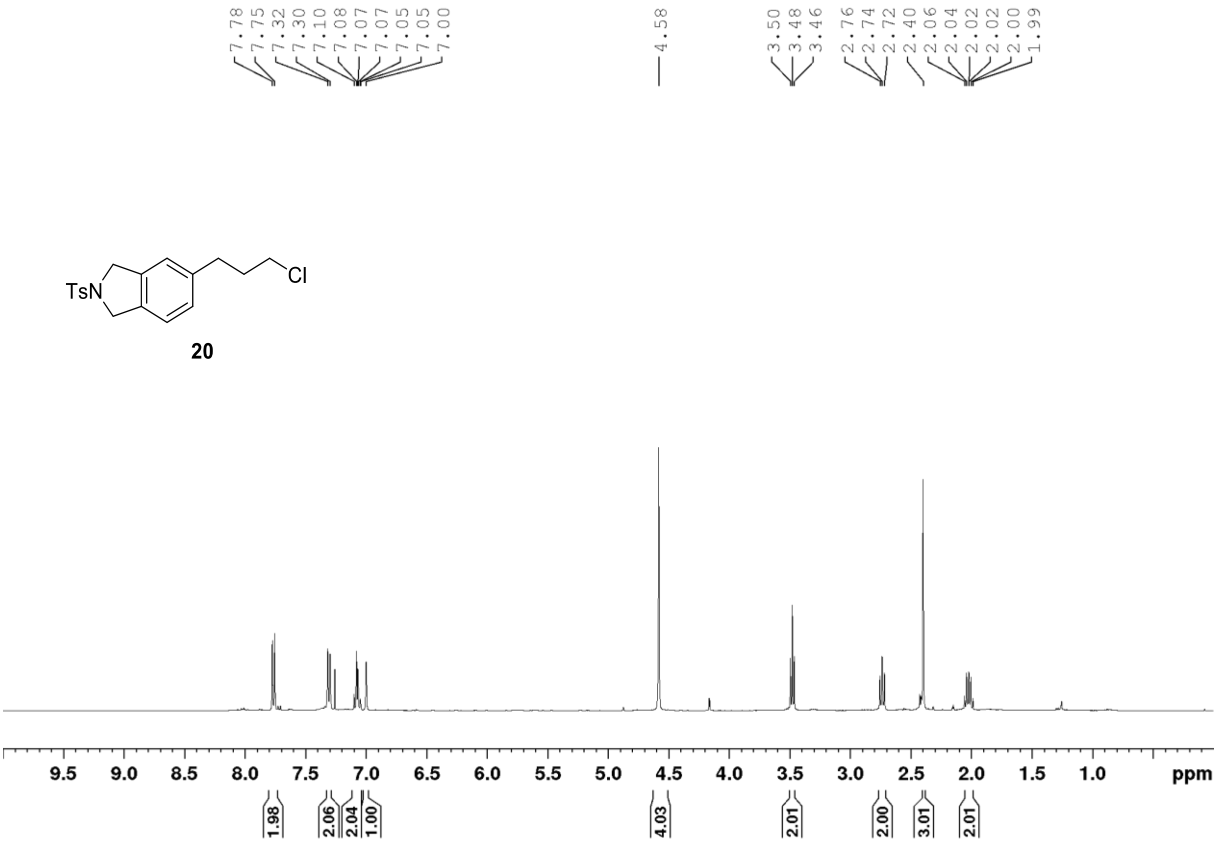

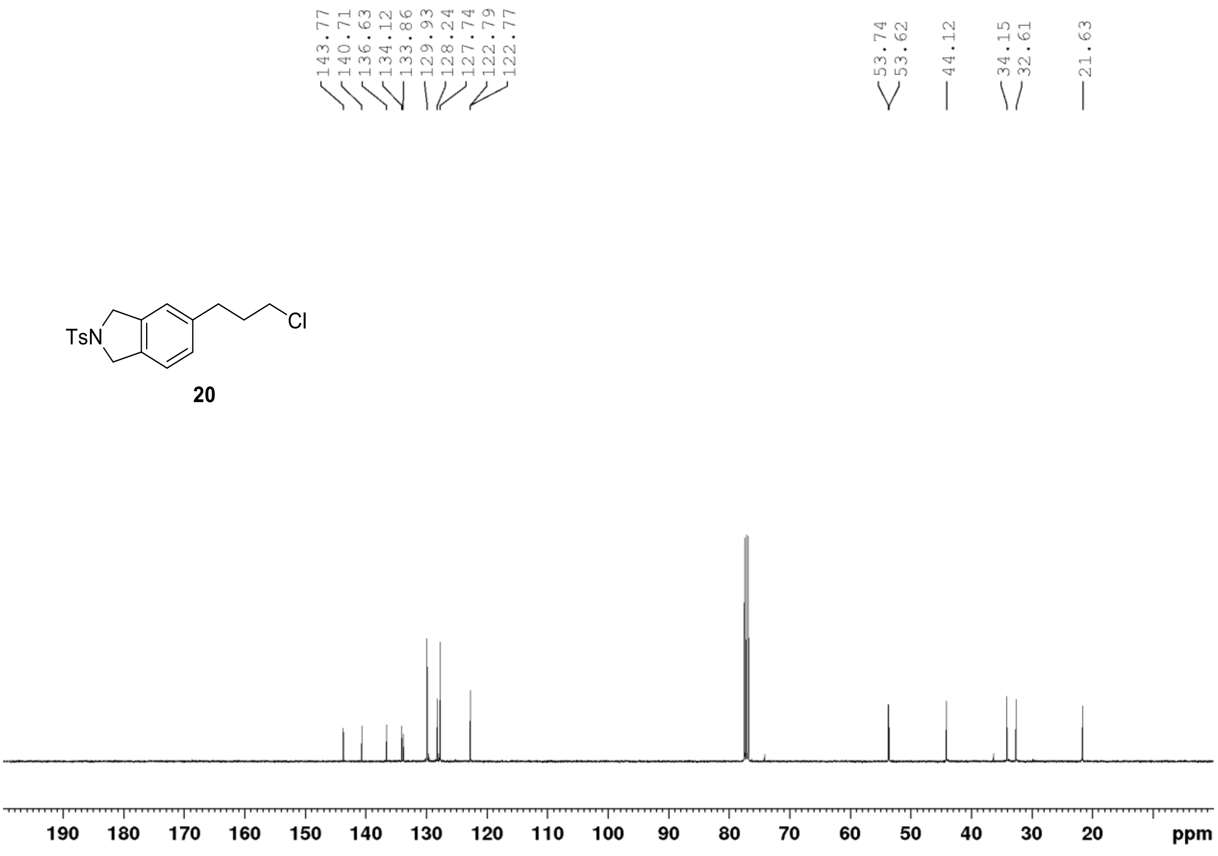

Copies of ^{1}H and ^{13}C NMR spectra of **21**

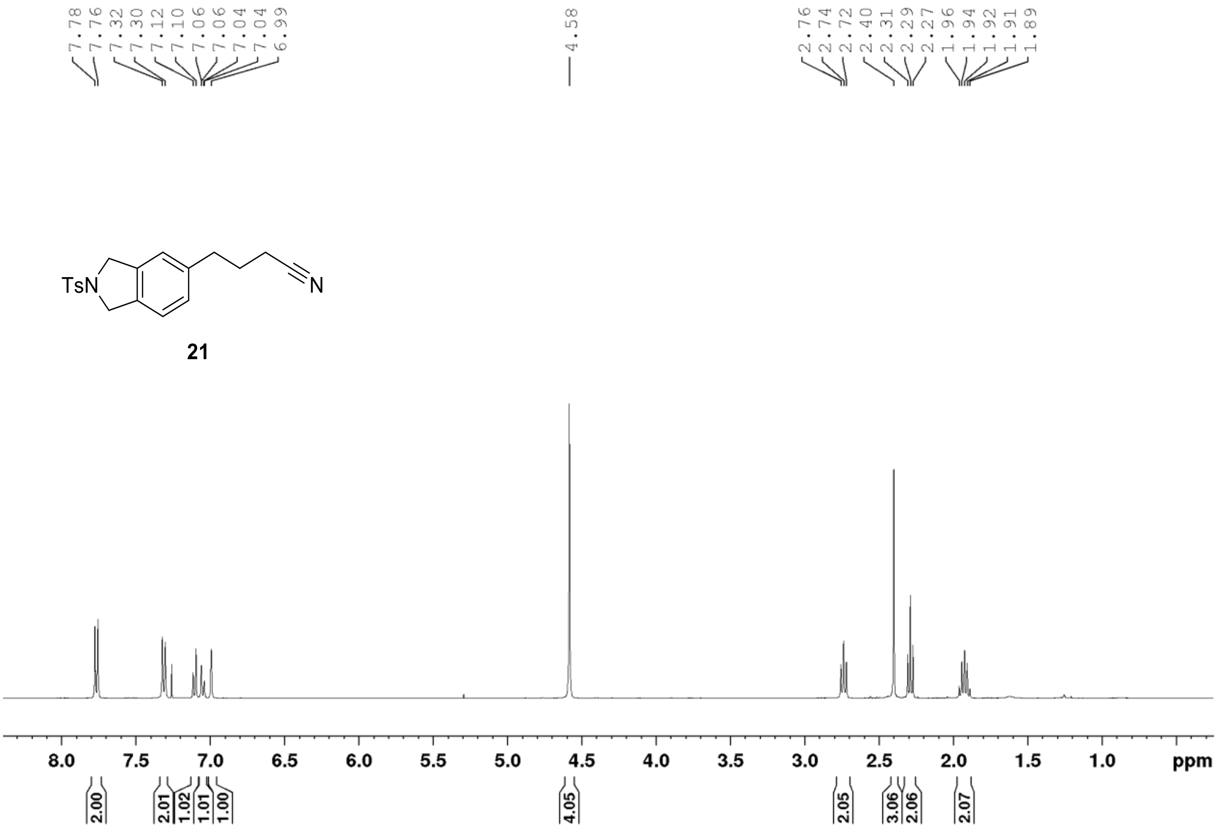

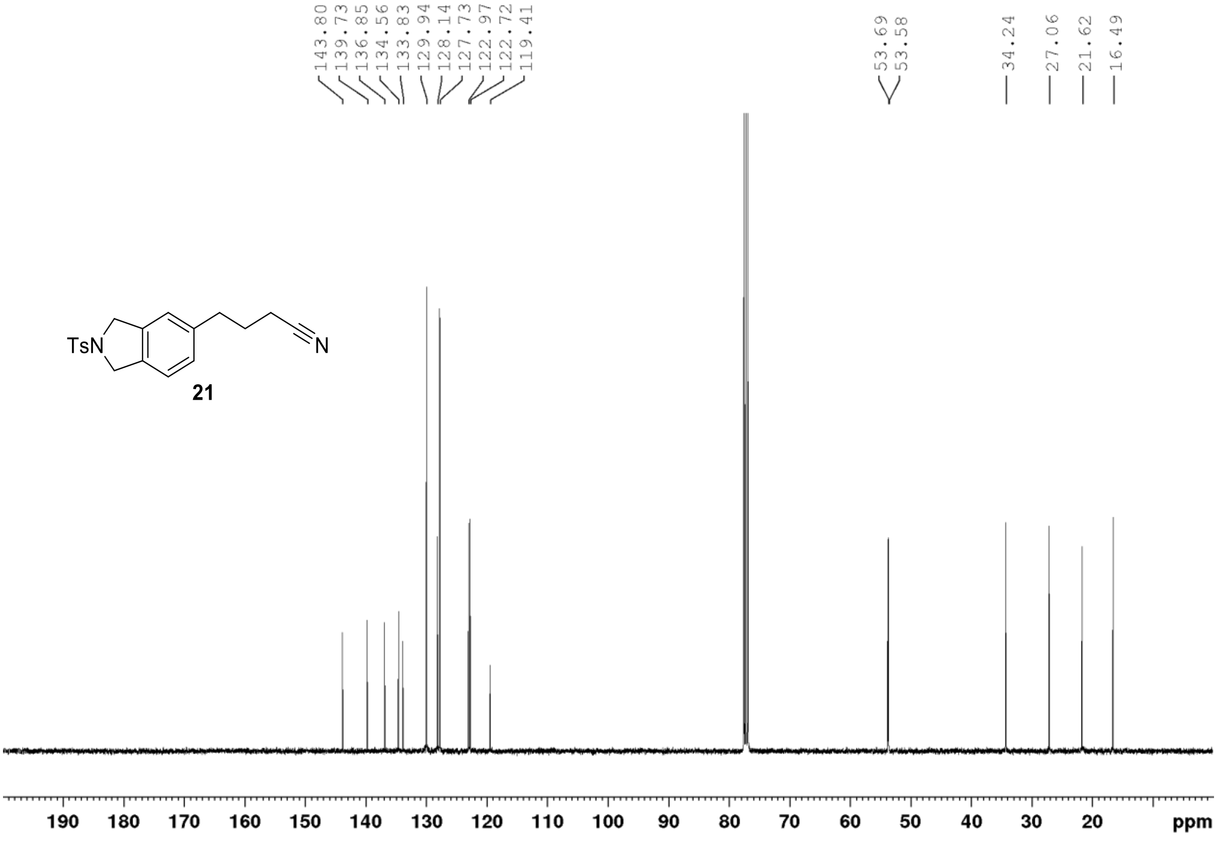

Copies of ^{1}H and ^{13}C NMR spectra of **22**

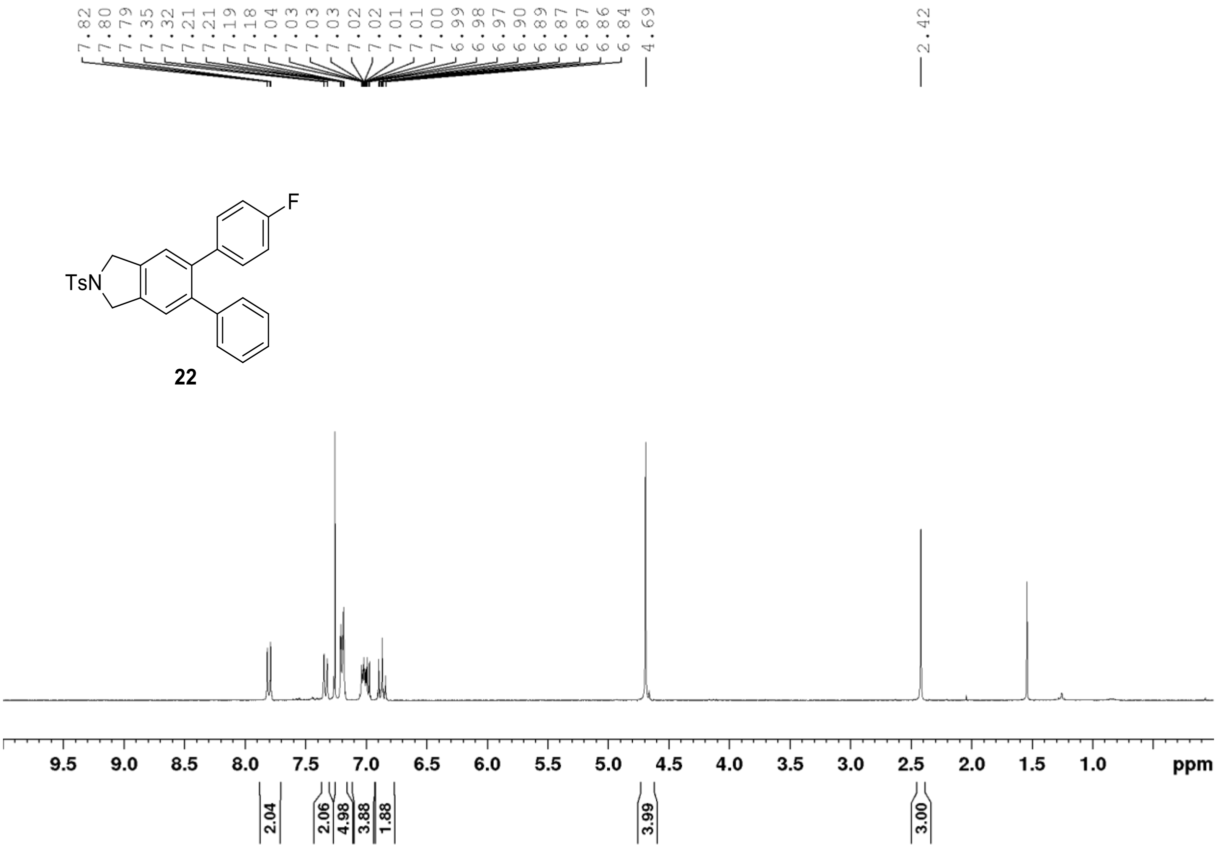

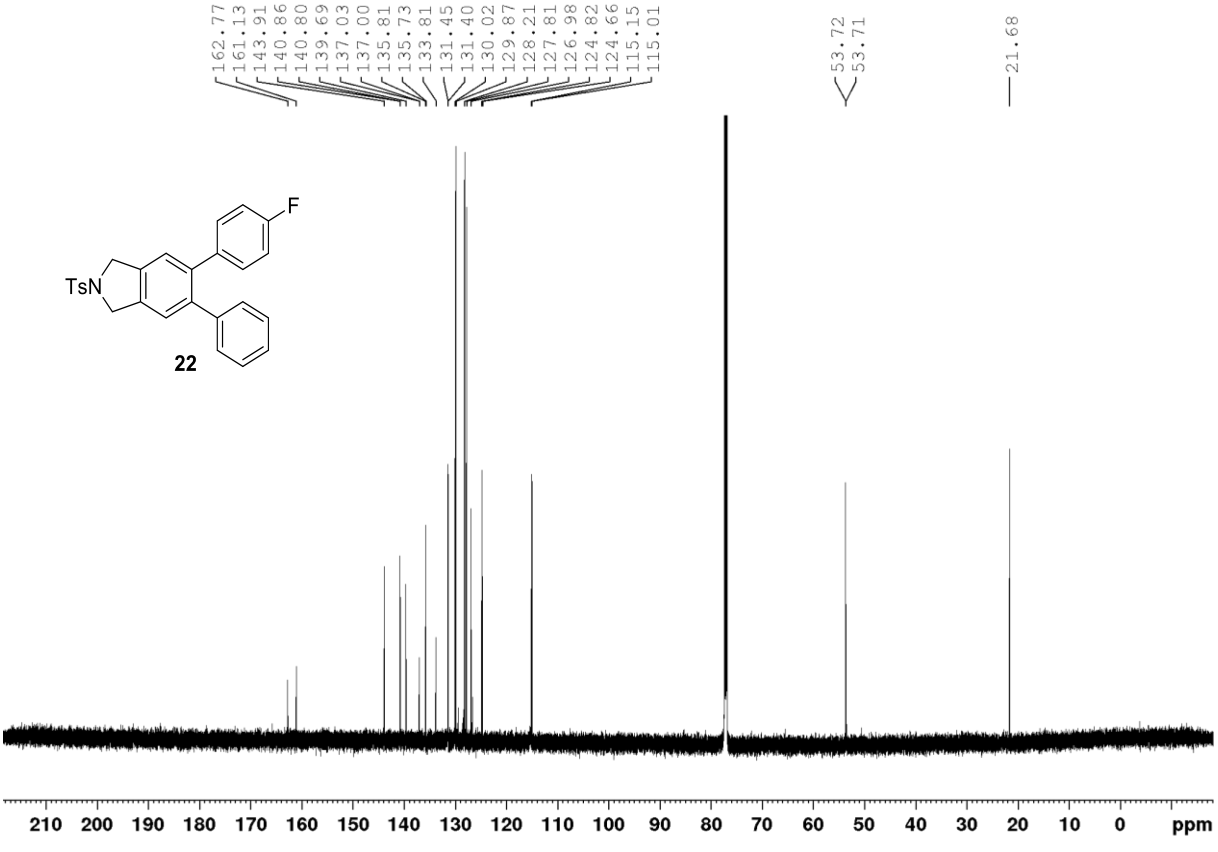

Copies of ^{1}H and ^{13}C NMR spectra of **23**

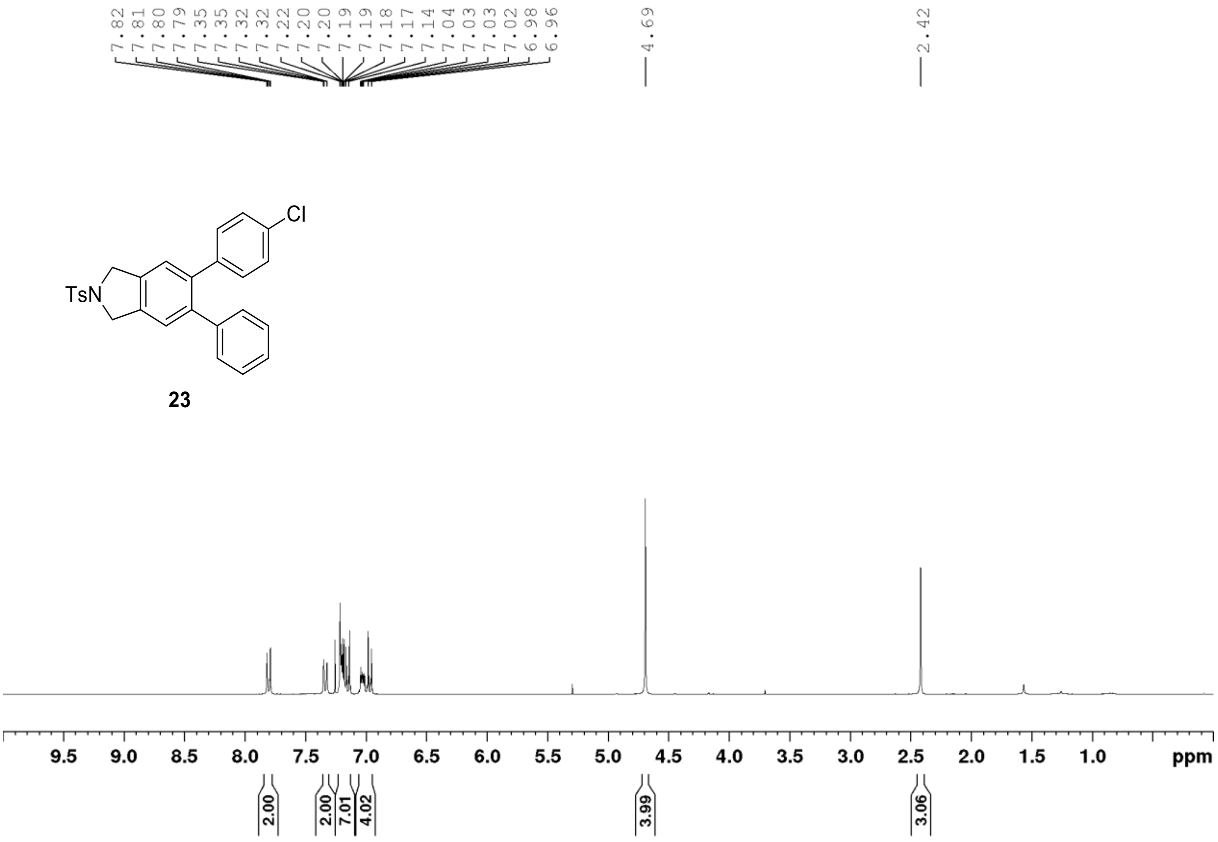

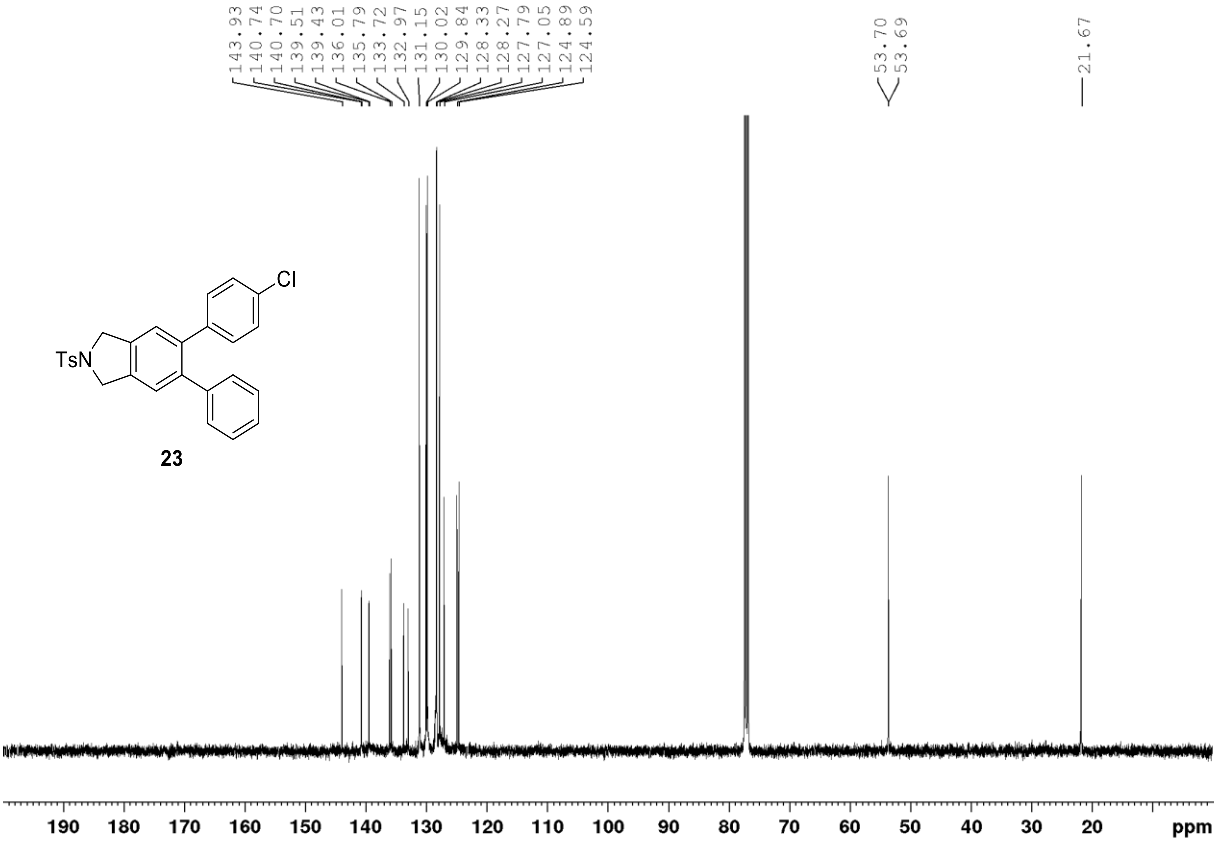

Copies of ^{1}H and ^{13}C NMR spectra of **24**

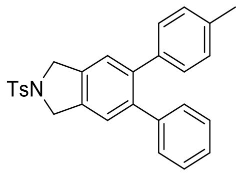

24

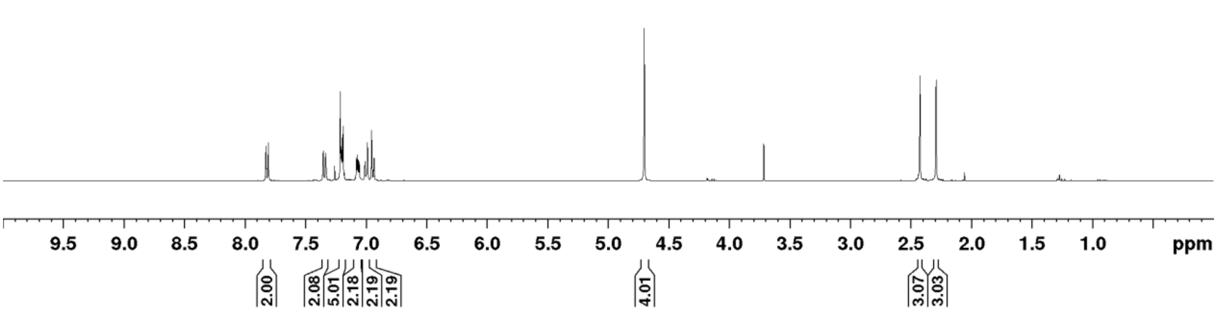

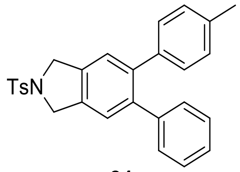

24

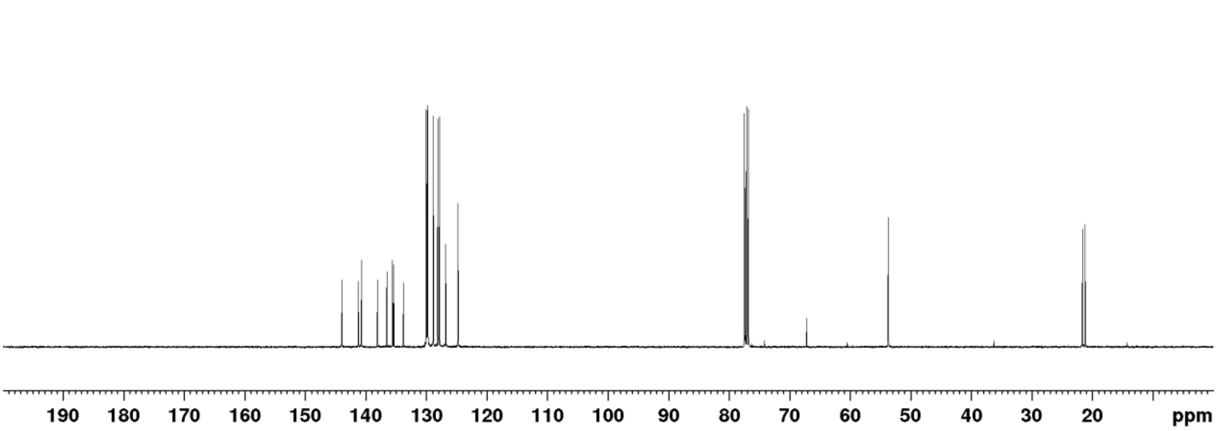

Copies of ^{1}H and ^{13}C NMR spectra of **25**

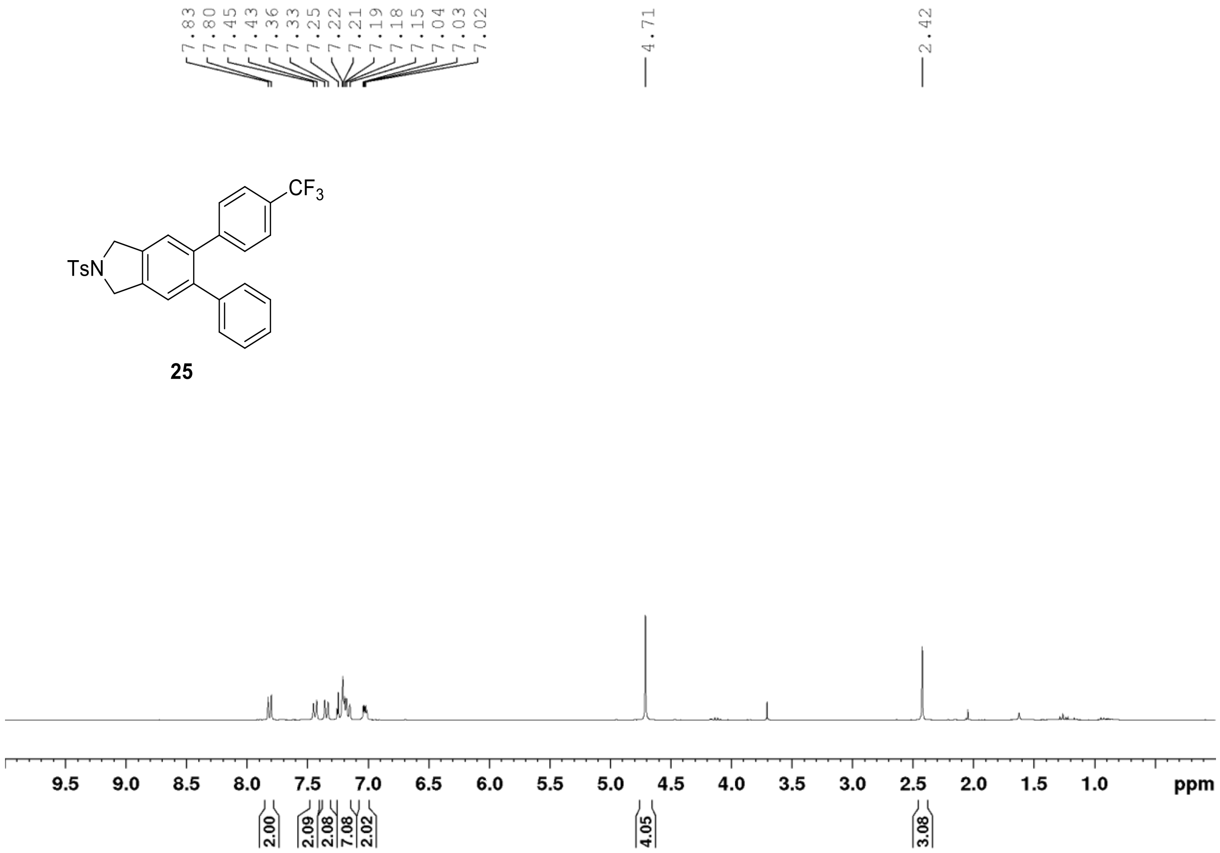

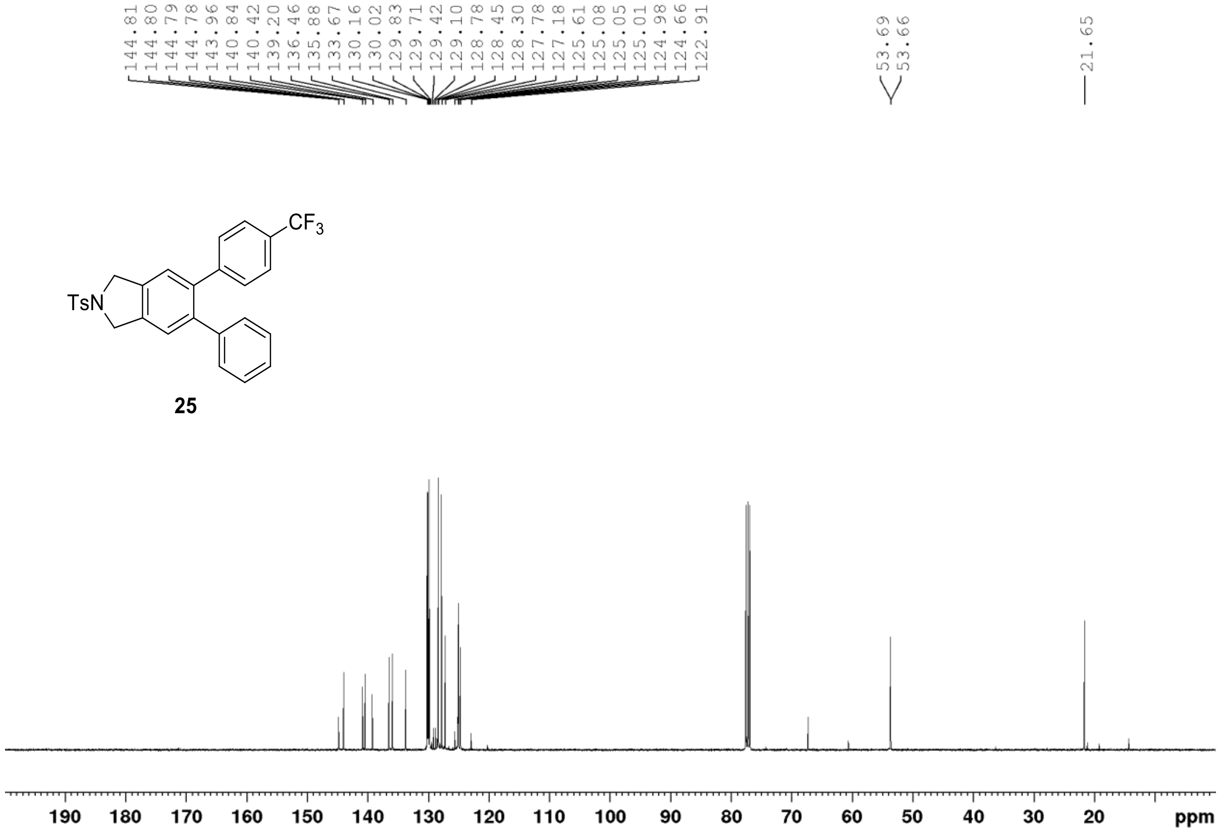

Copies of ^{1}H and ^{13}C NMR spectra of **26**

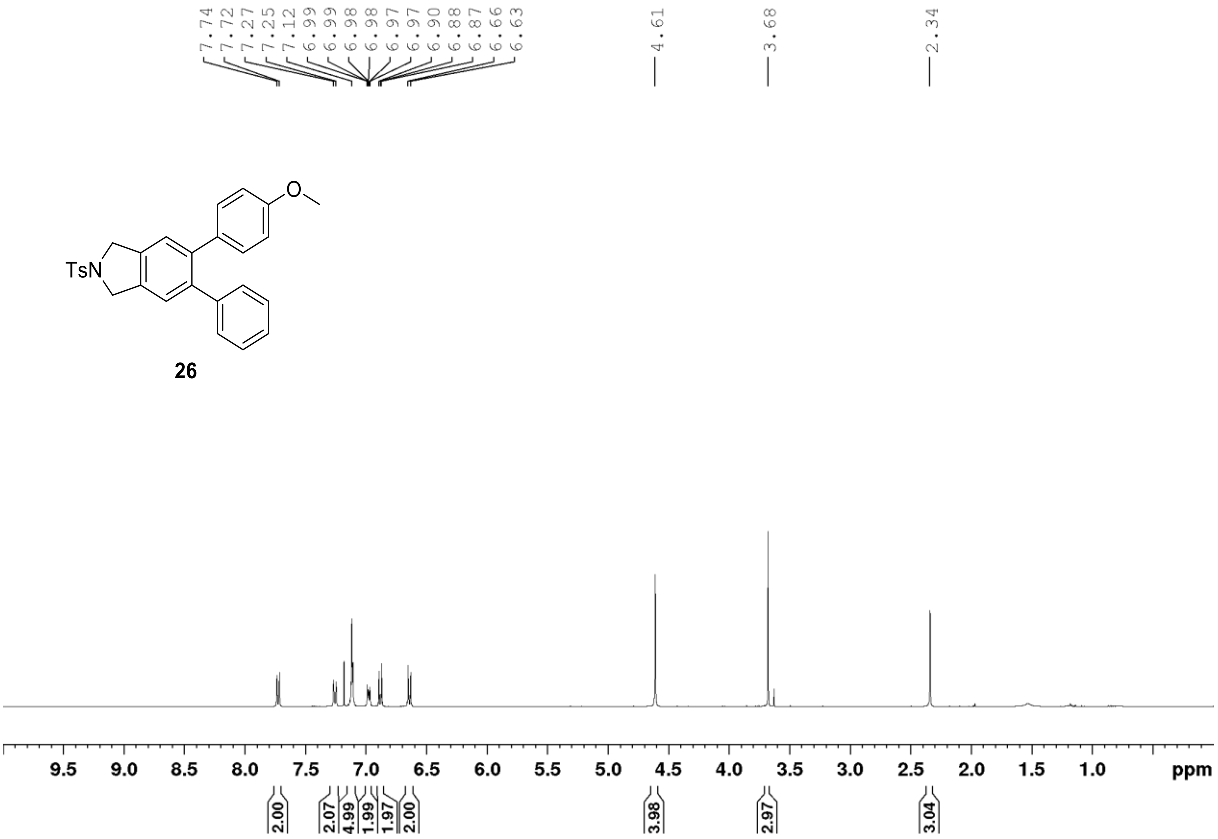

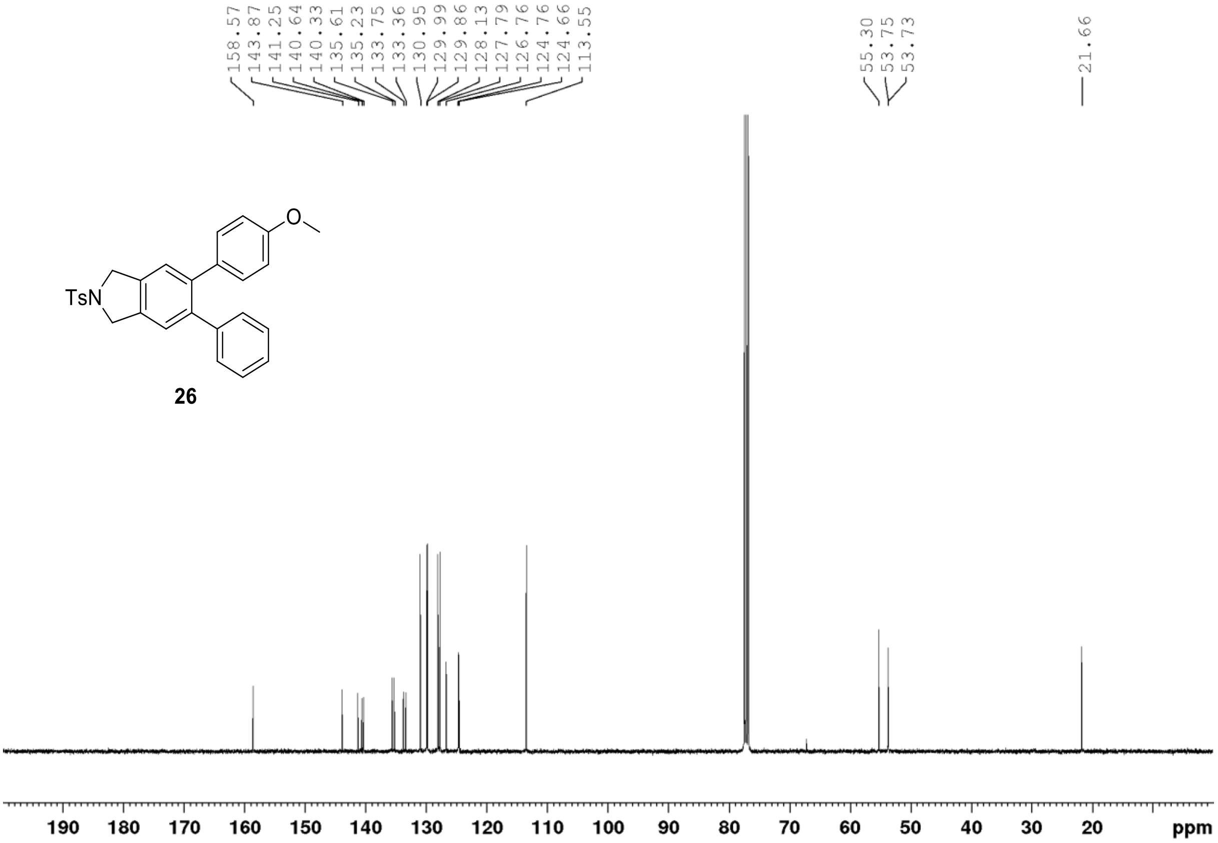

Copies of ^{1}H and ^{13}C NMR spectra of **27**

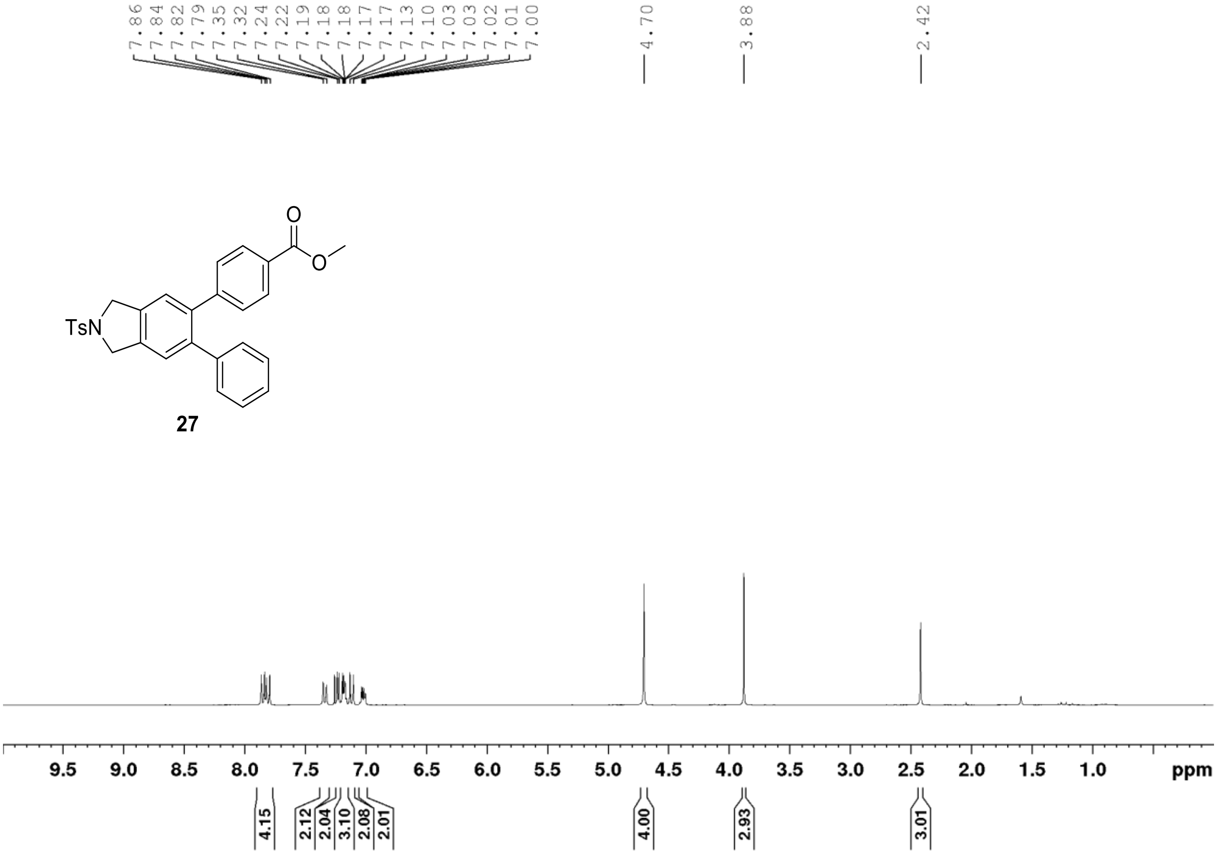

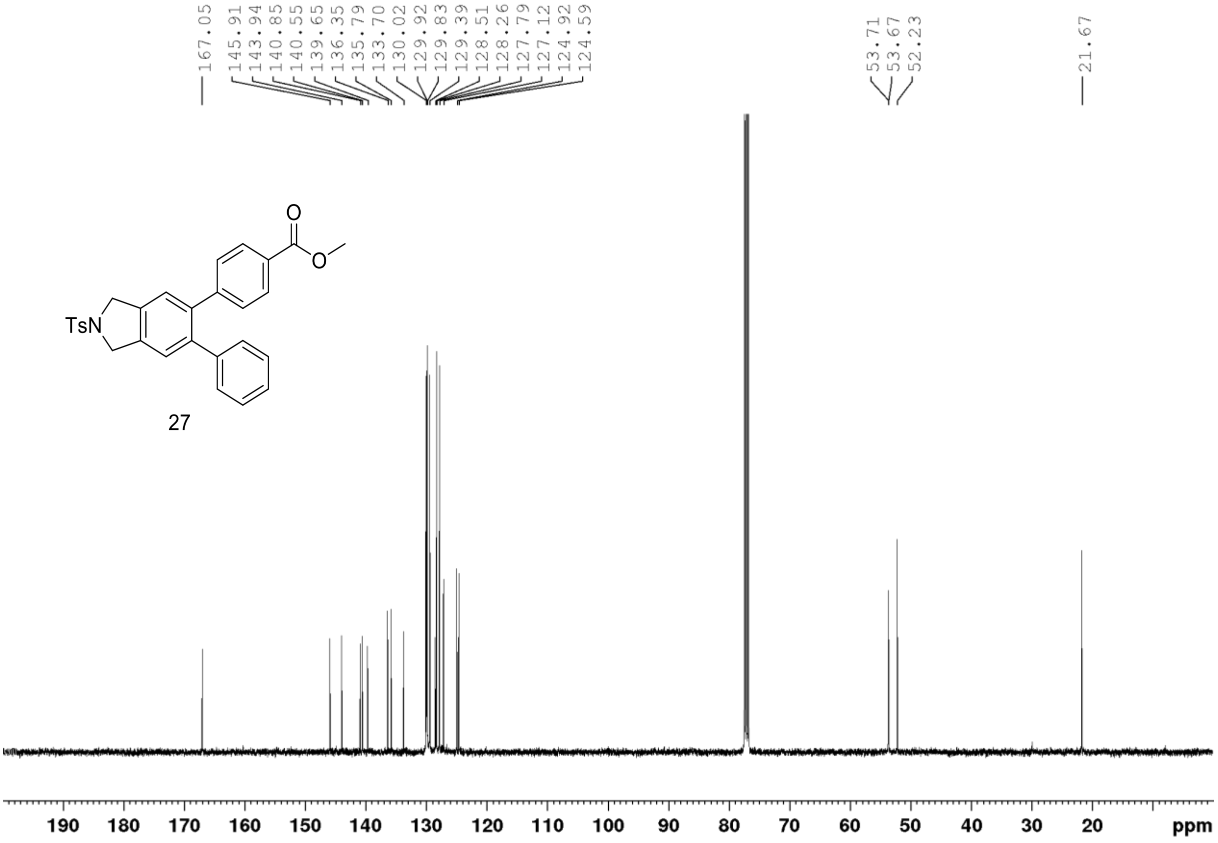

Copies of ^{1}H and ^{13}C NMR spectra of **28**

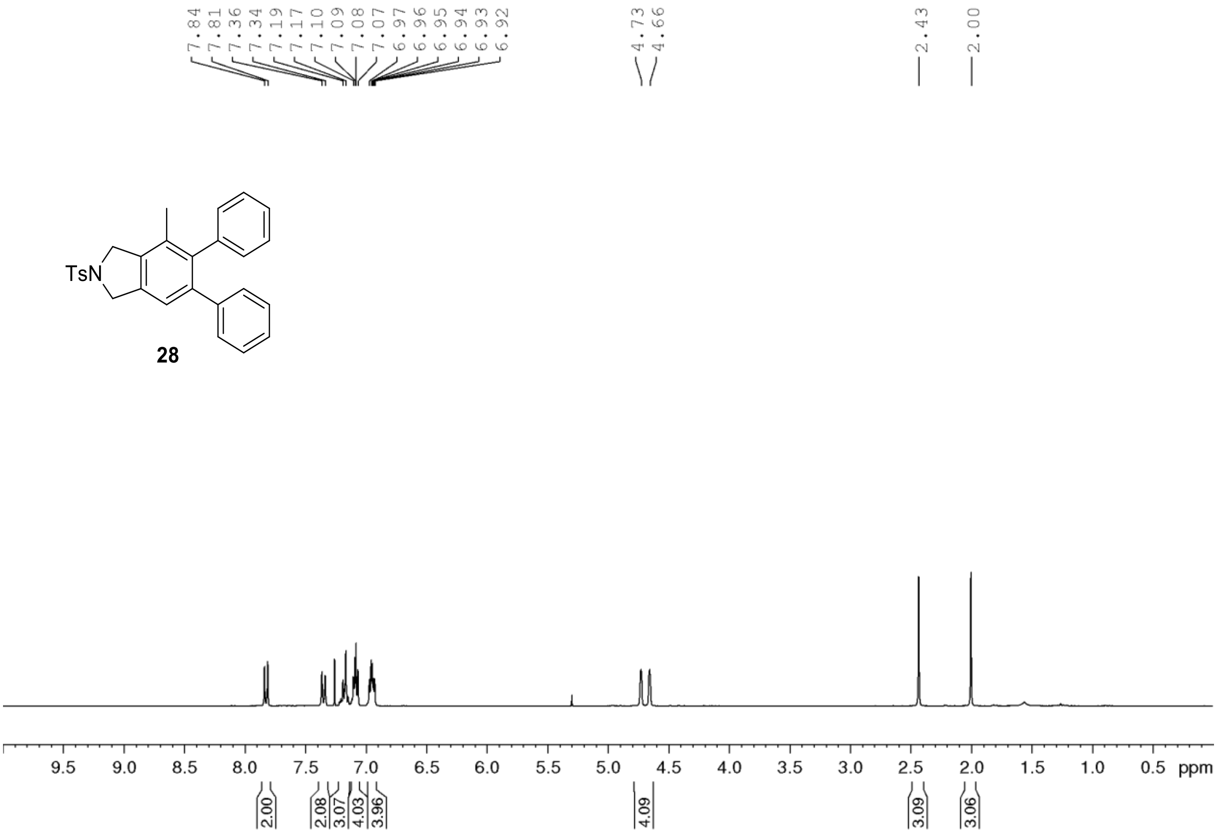

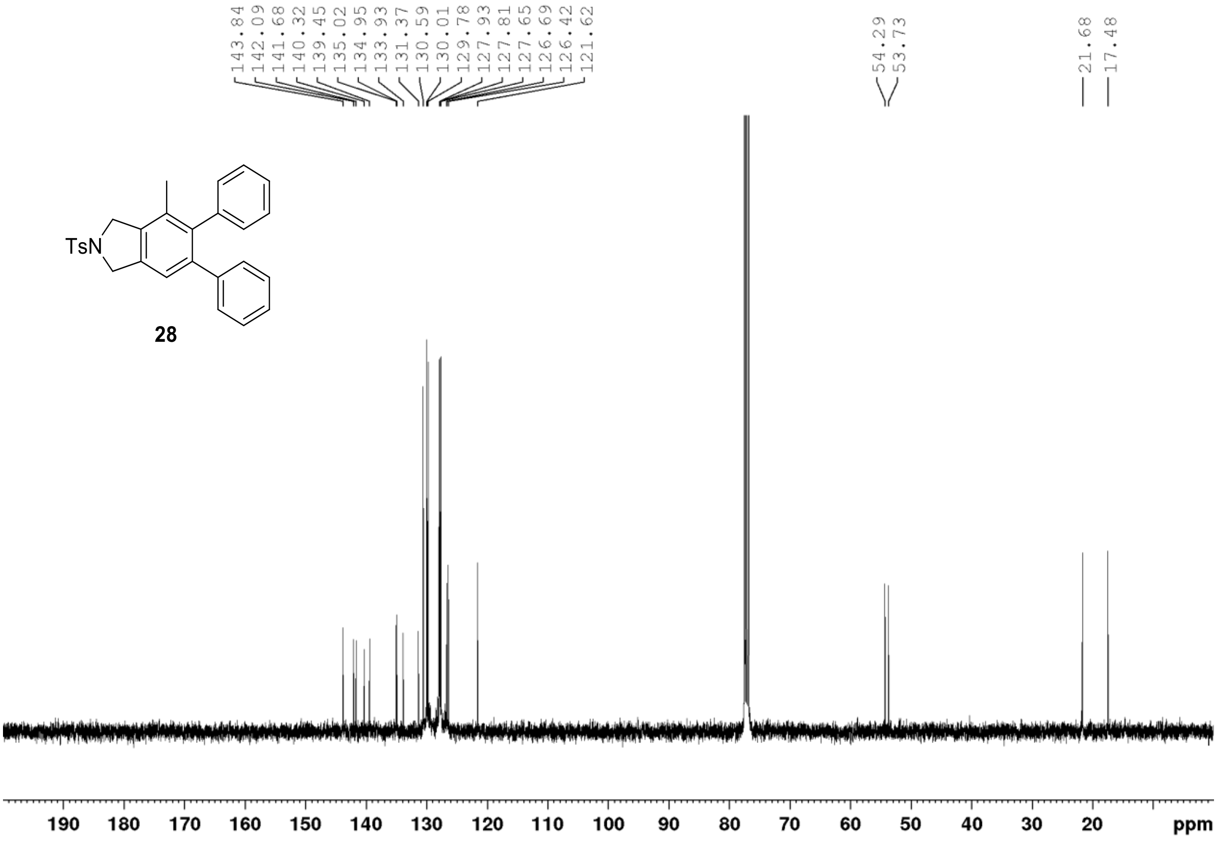

Copies of ^{1}H and ^{13}C NMR spectra of **29**

29

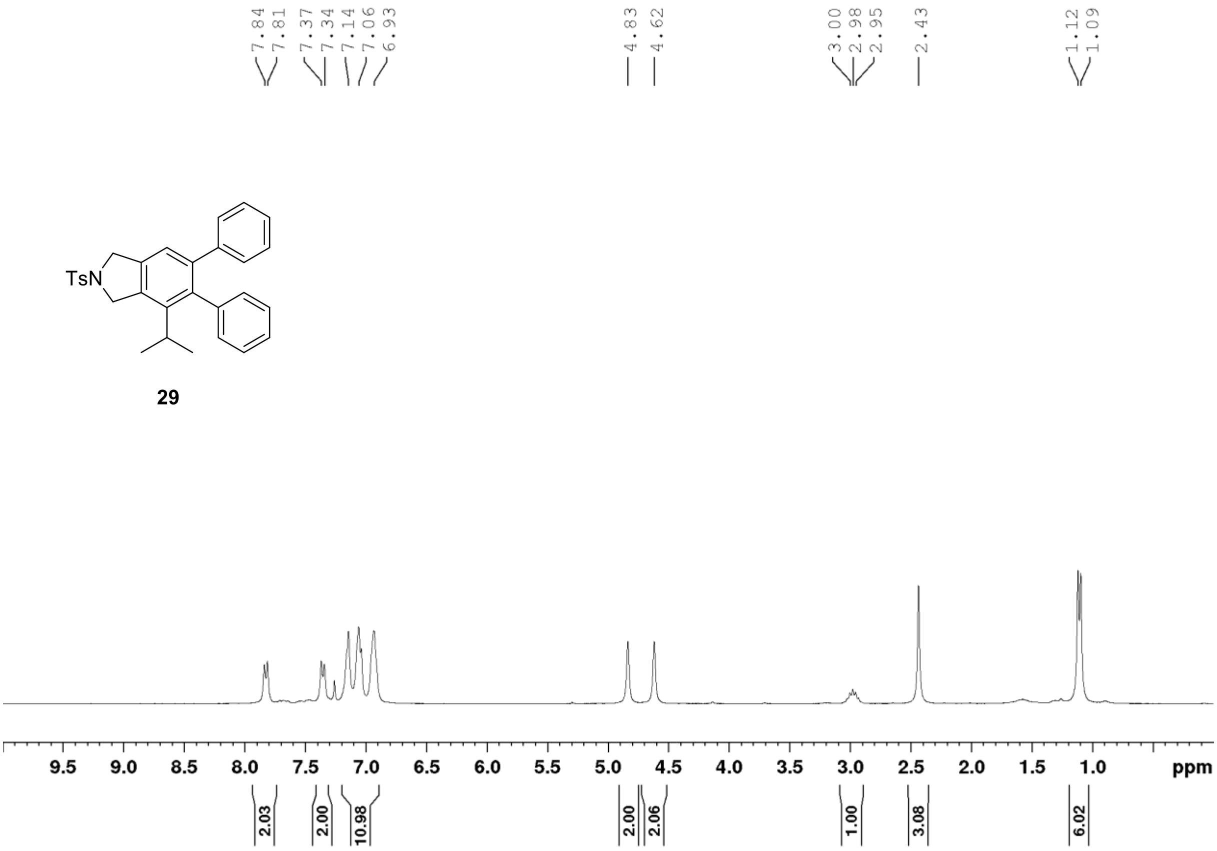

29

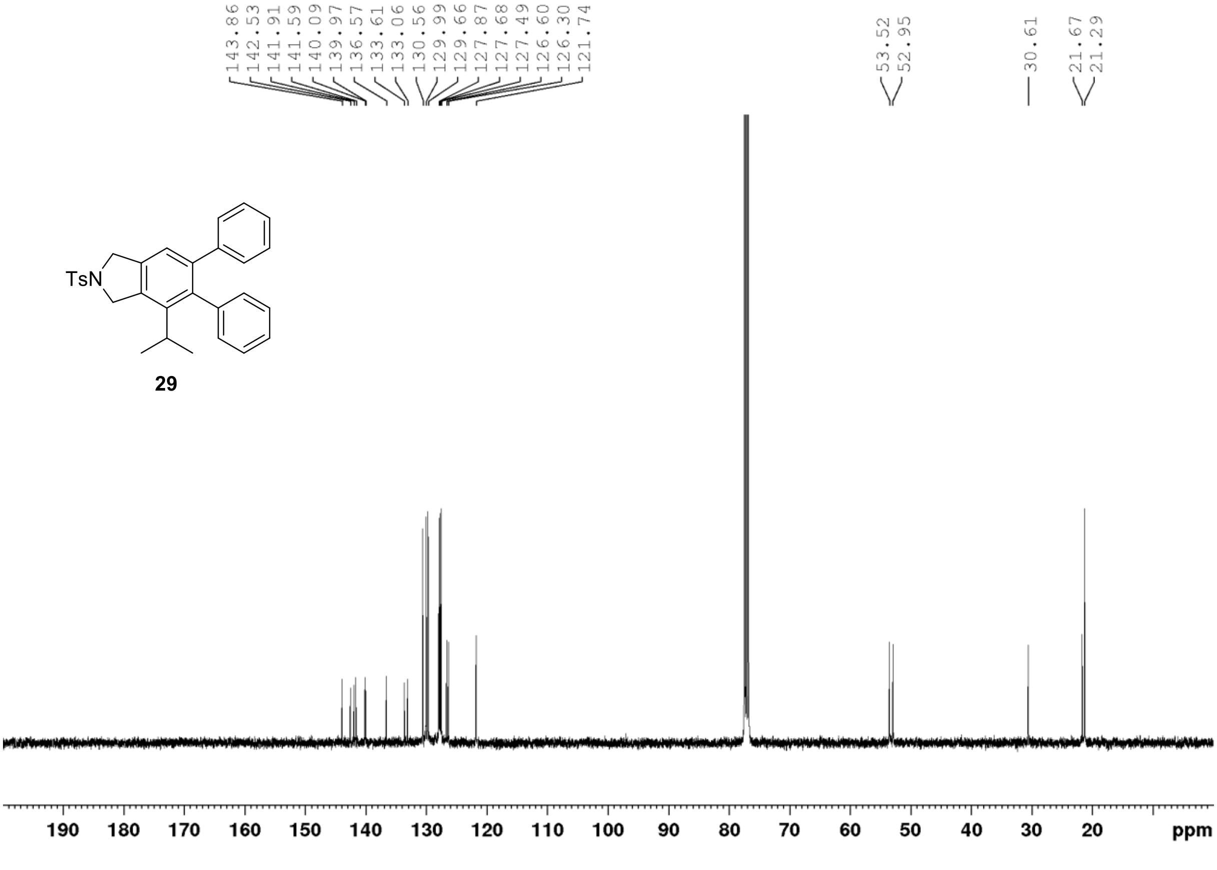

Copies of ^{1}H and ^{13}C NMR spectra of **30**

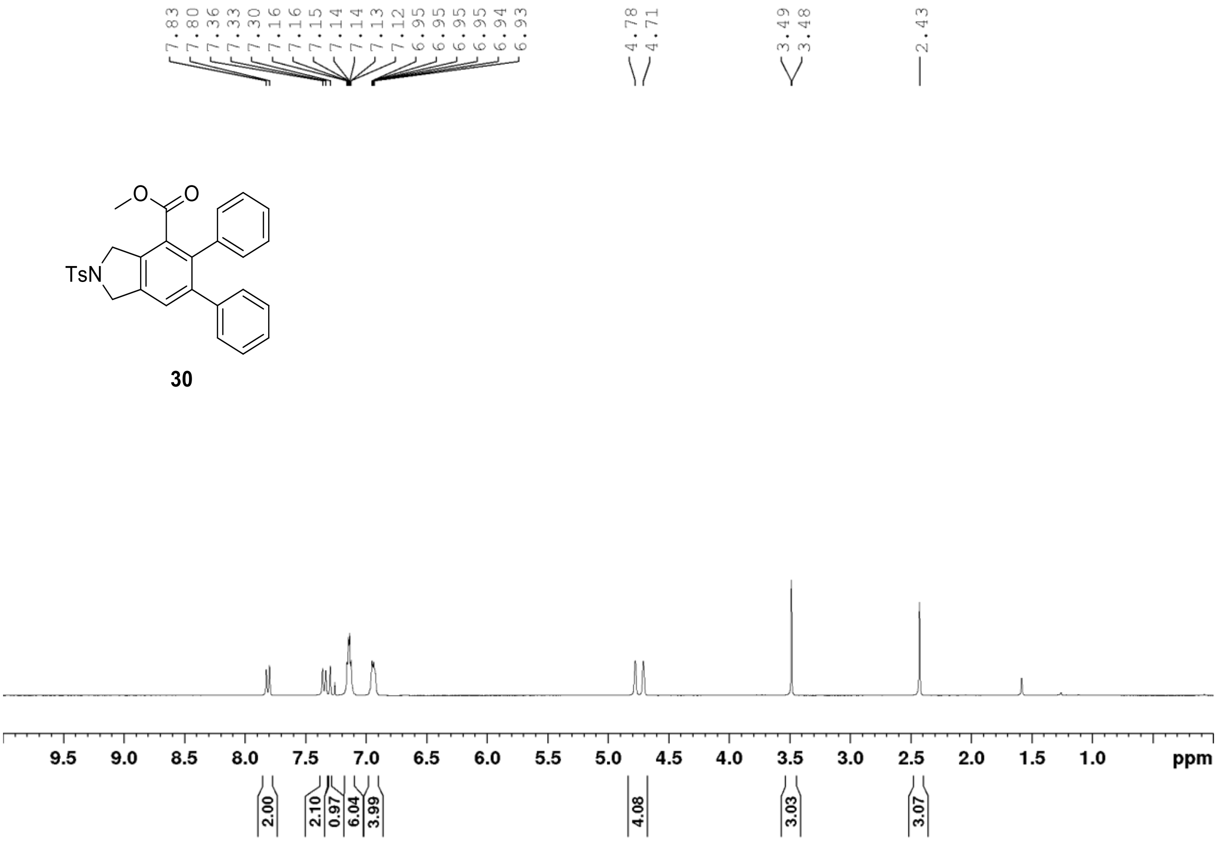

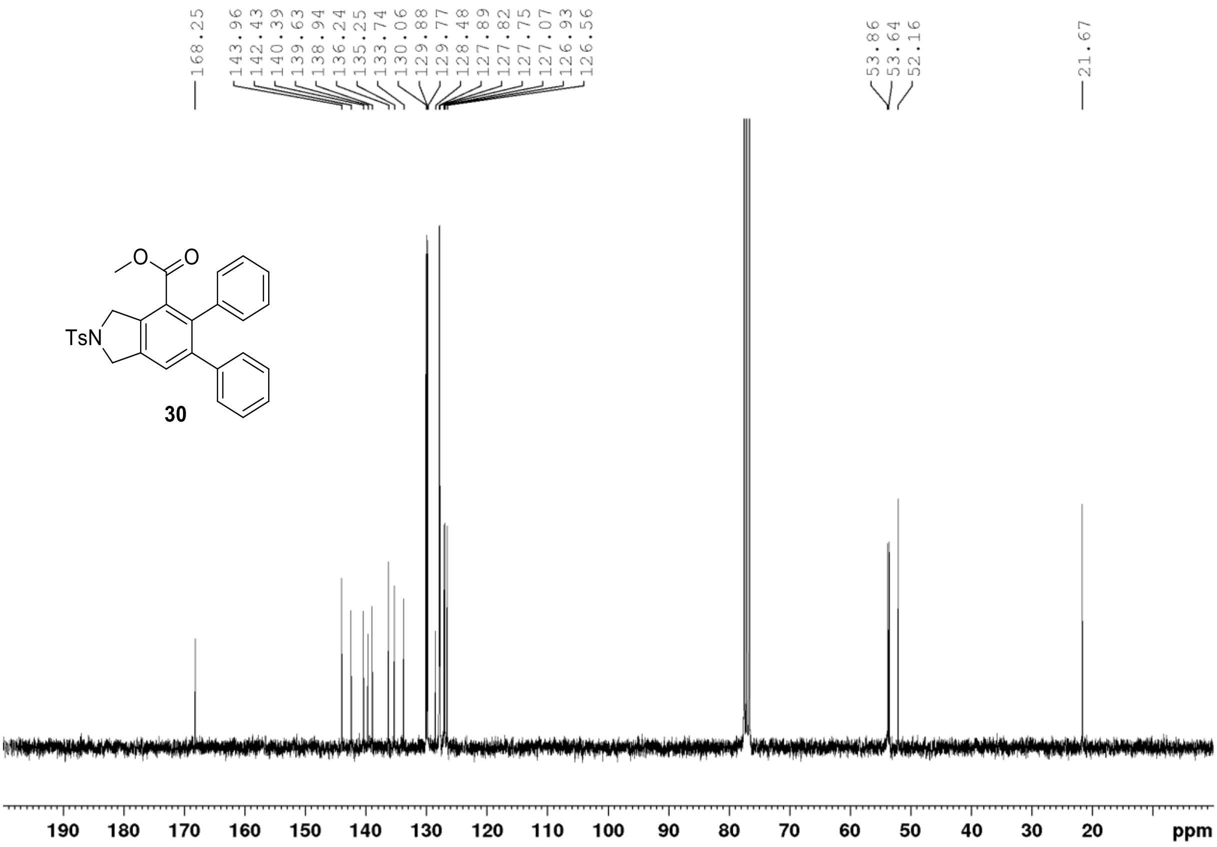

Copies of ^{1}H and ^{13}C NMR spectra of **31**

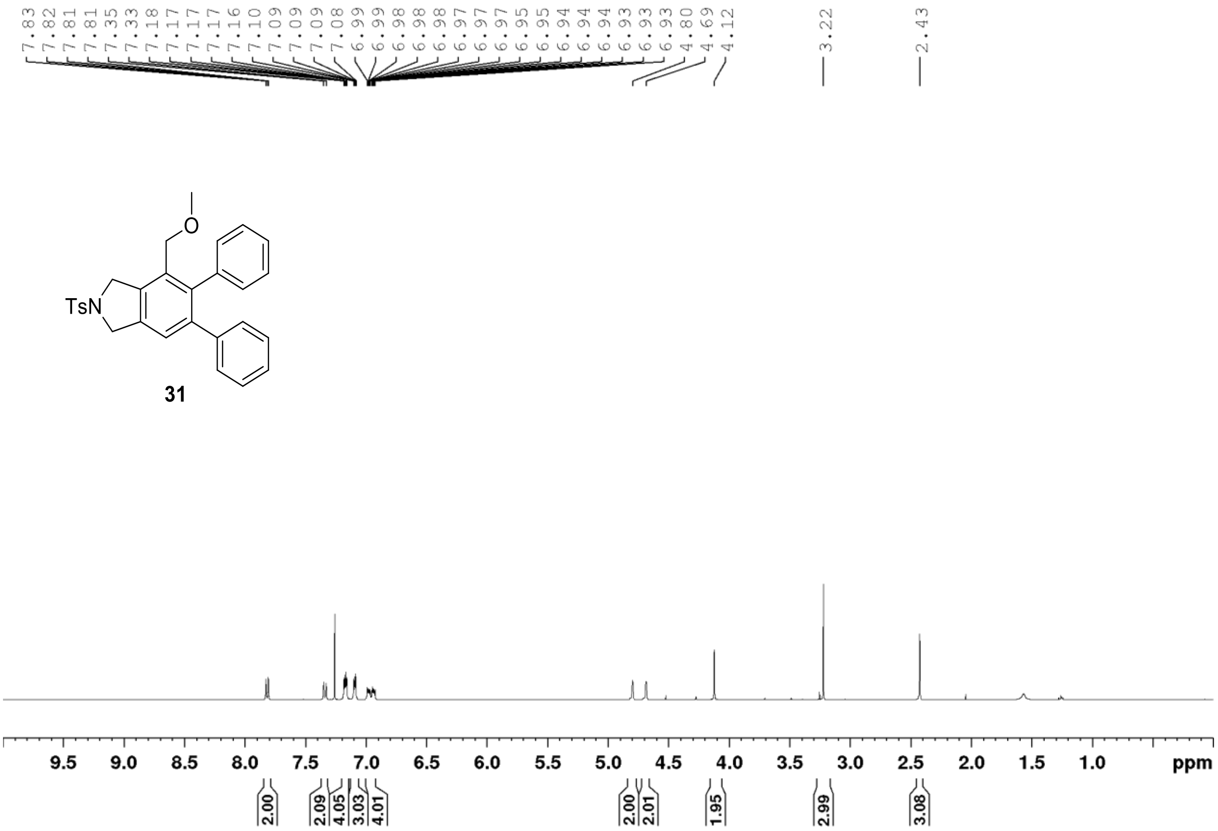

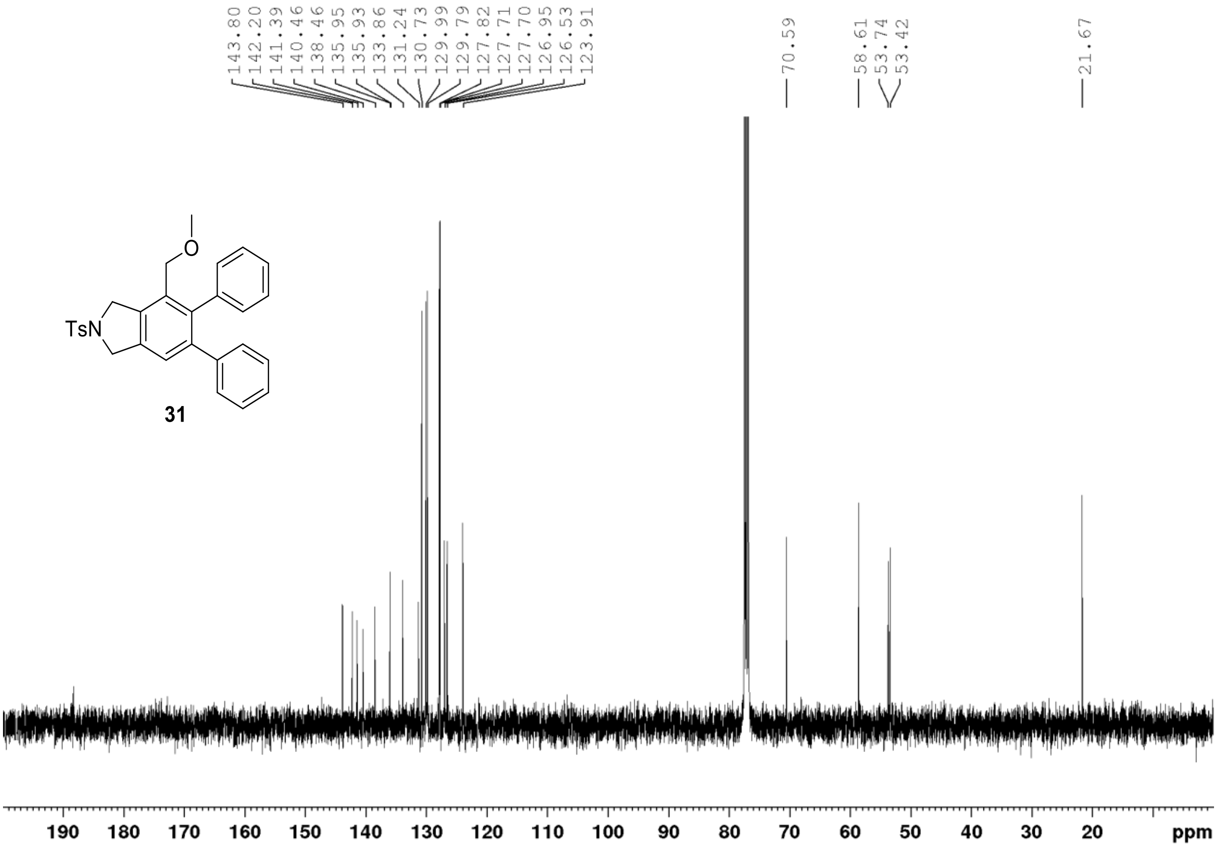

Copies of ^{1}H and ^{13}C NMR spectra of **32**

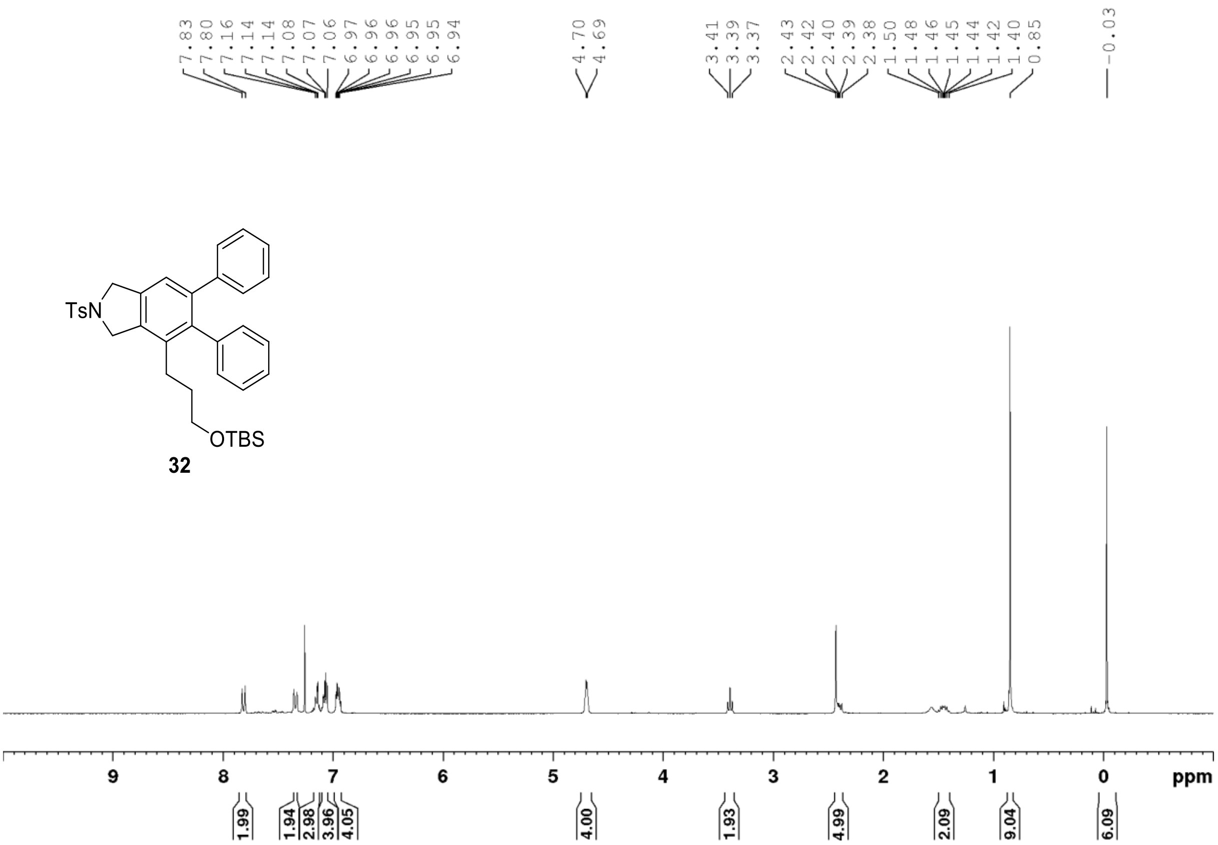

32

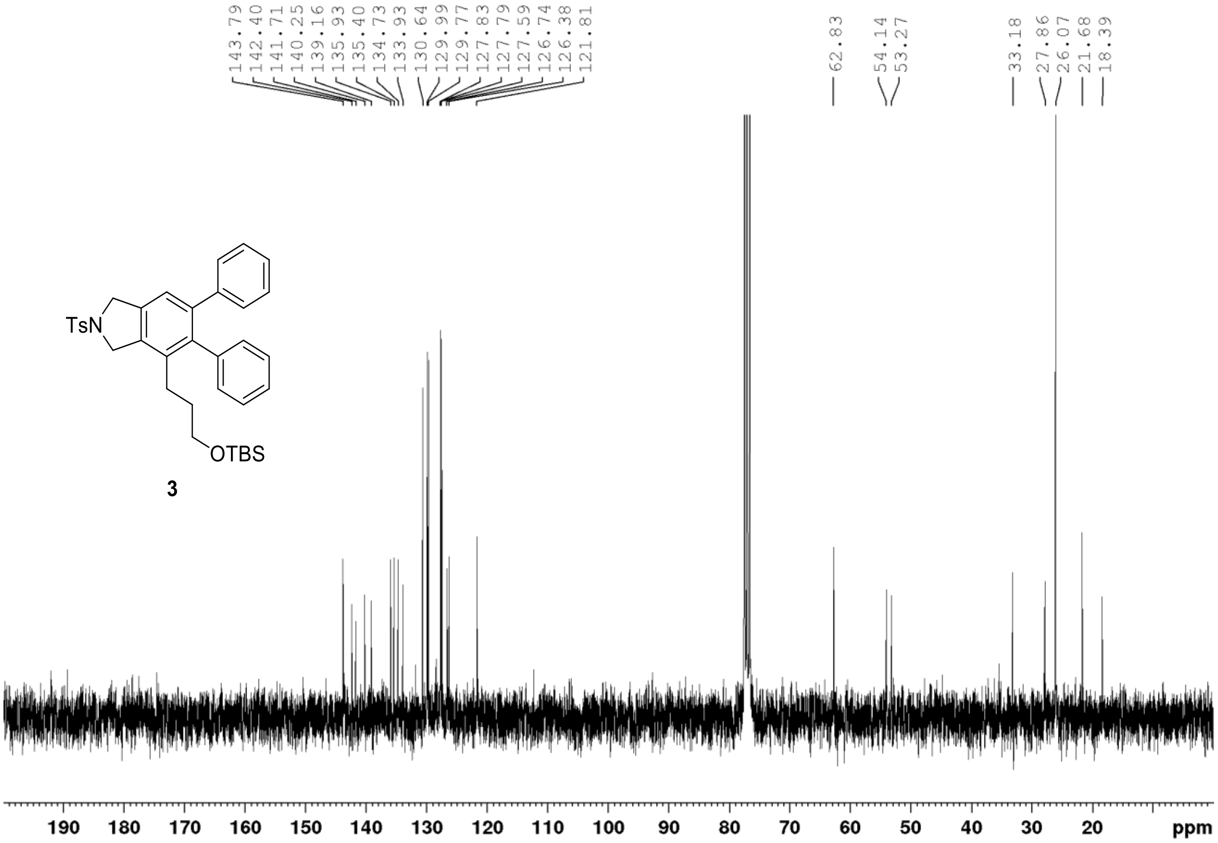

3

Copies of ^{1}H and ^{13}C NMR spectra of **33**

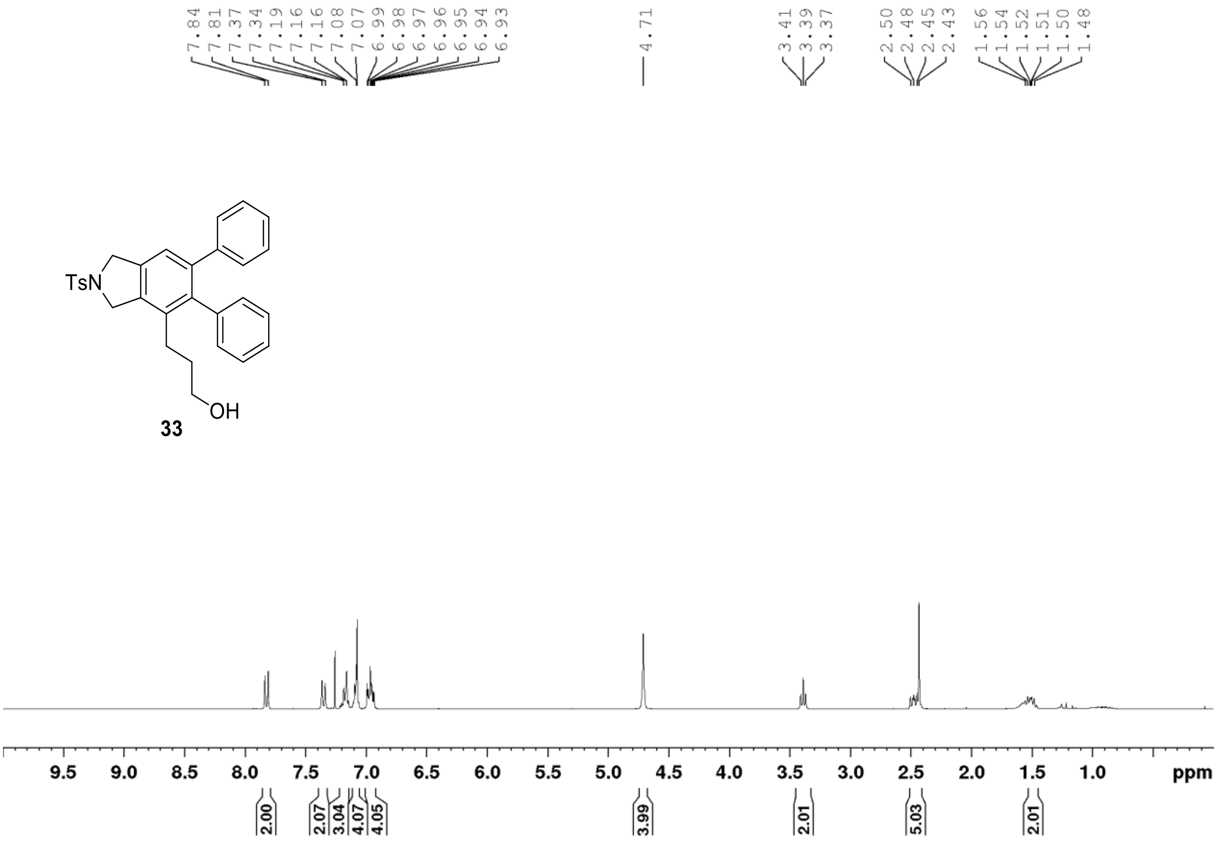

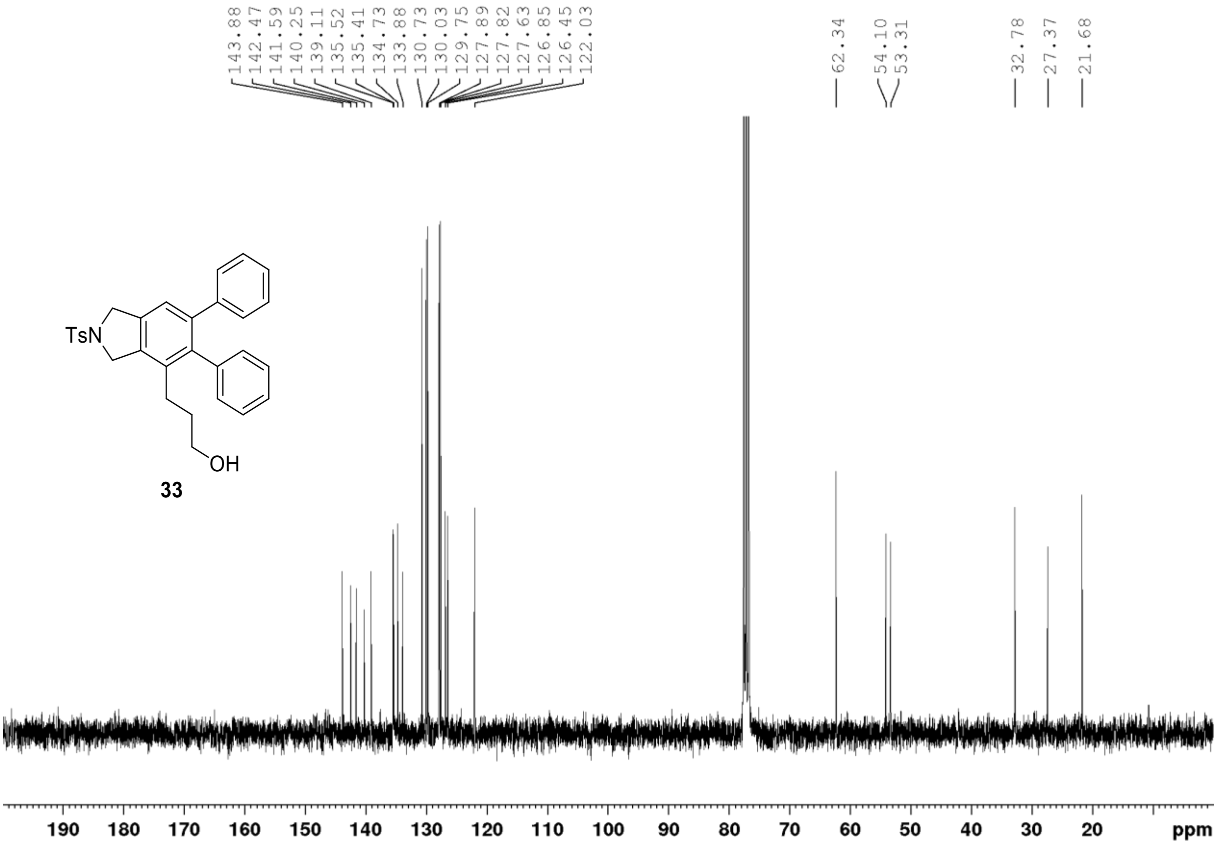

Copies of ^{1}H and ^{13}C NMR spectra of **34**

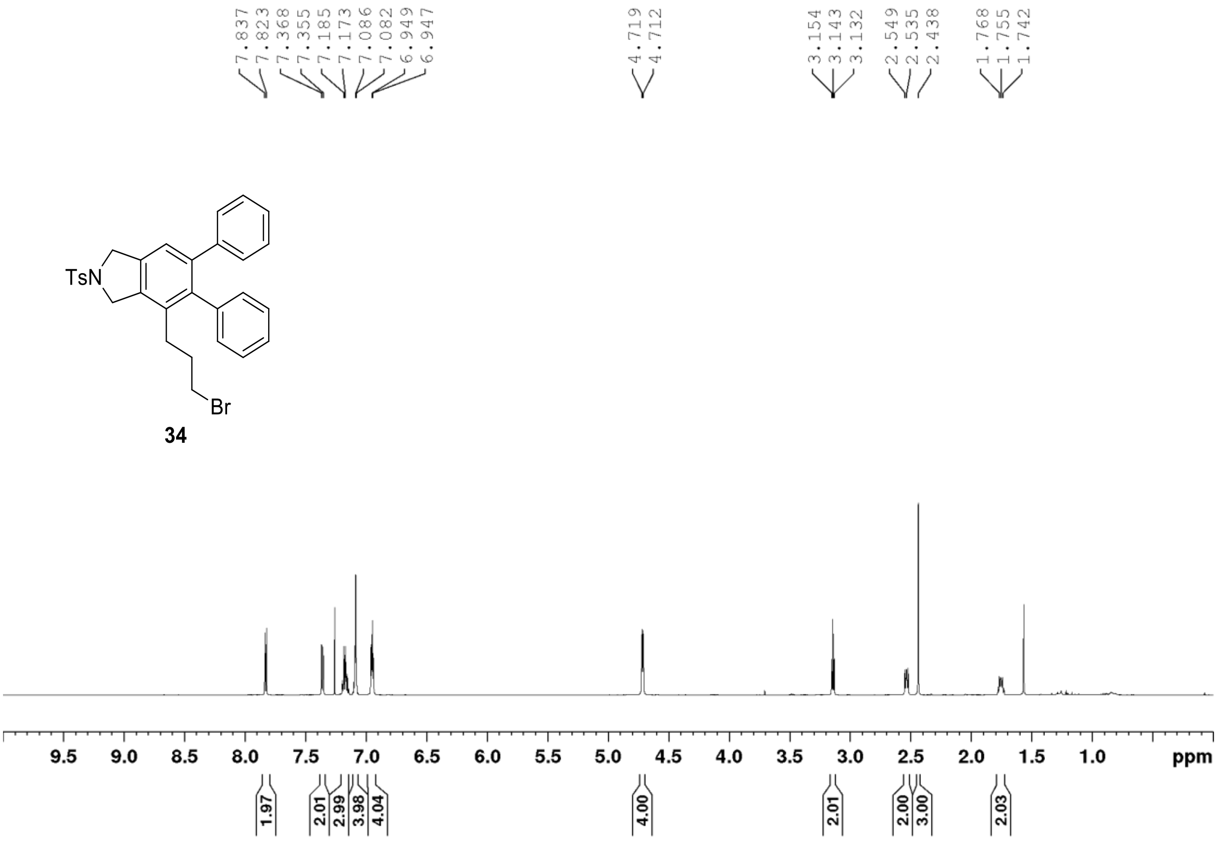

34

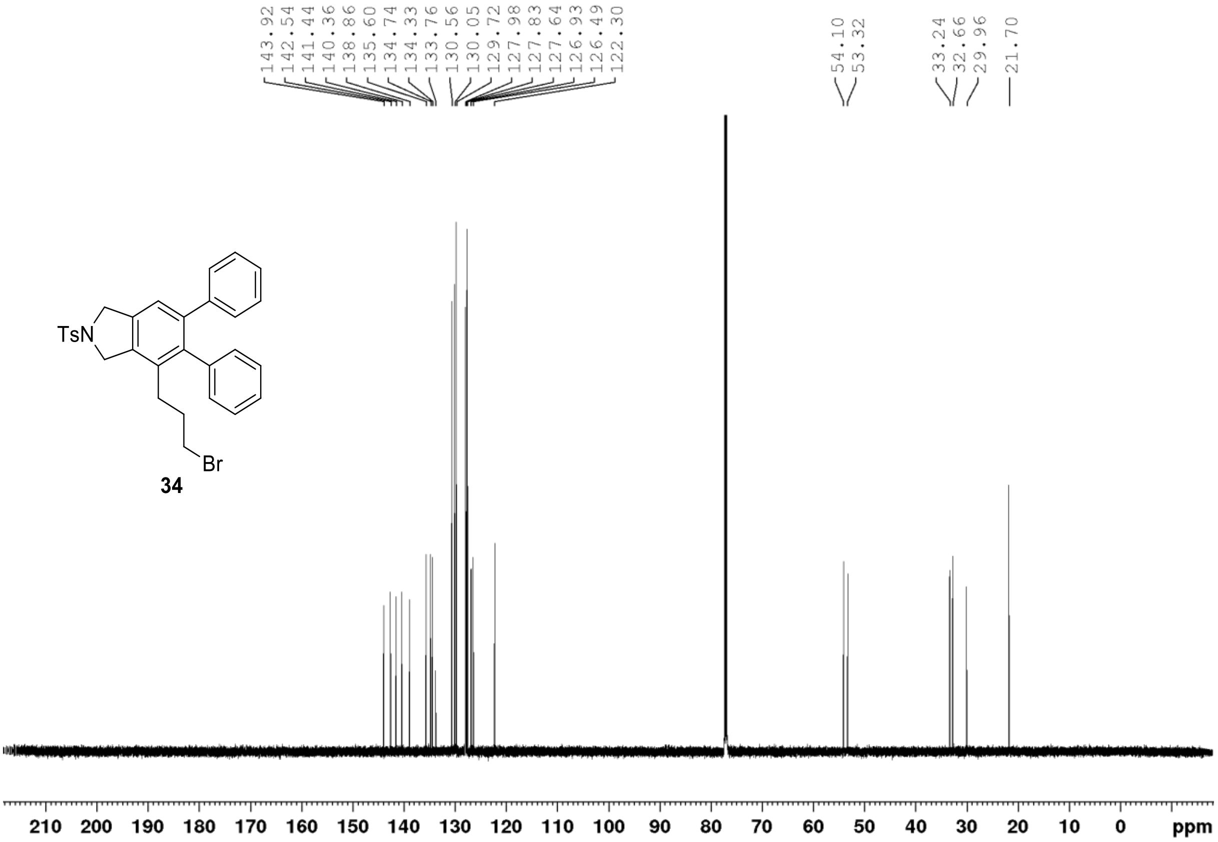

34

Copies of ^{1}H and ^{13}C NMR spectra of **35**

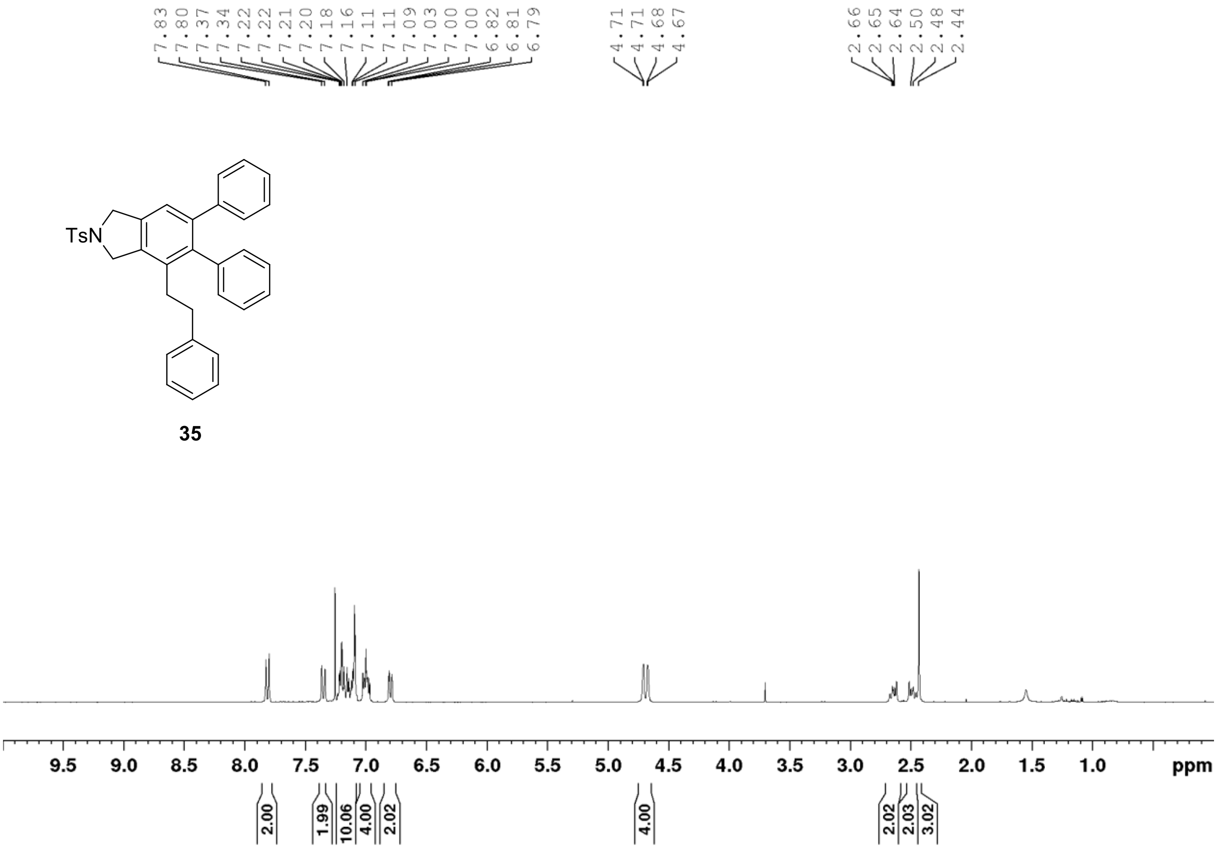

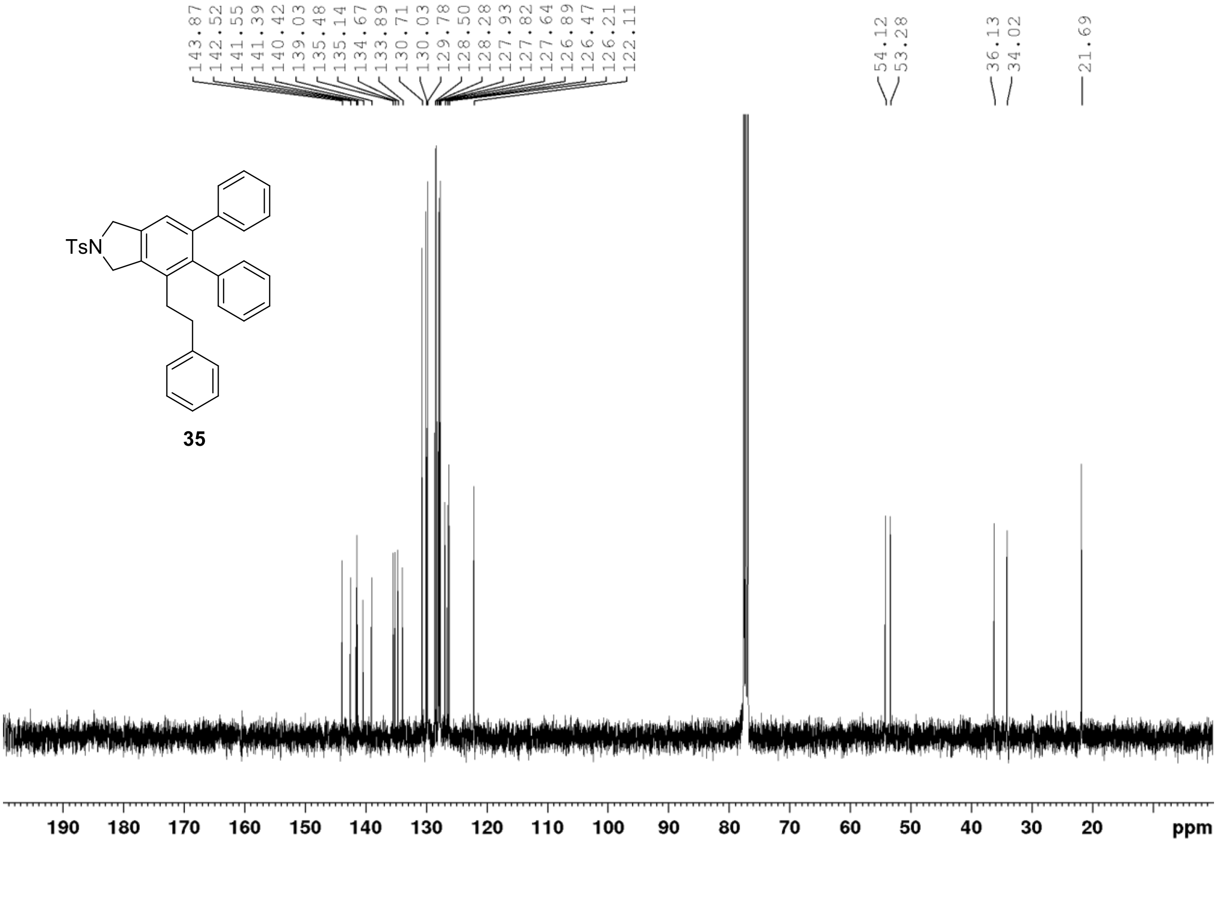

Copies of ^{1}H and ^{13}C NMR spectra of **36**

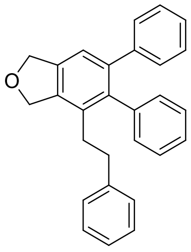

36

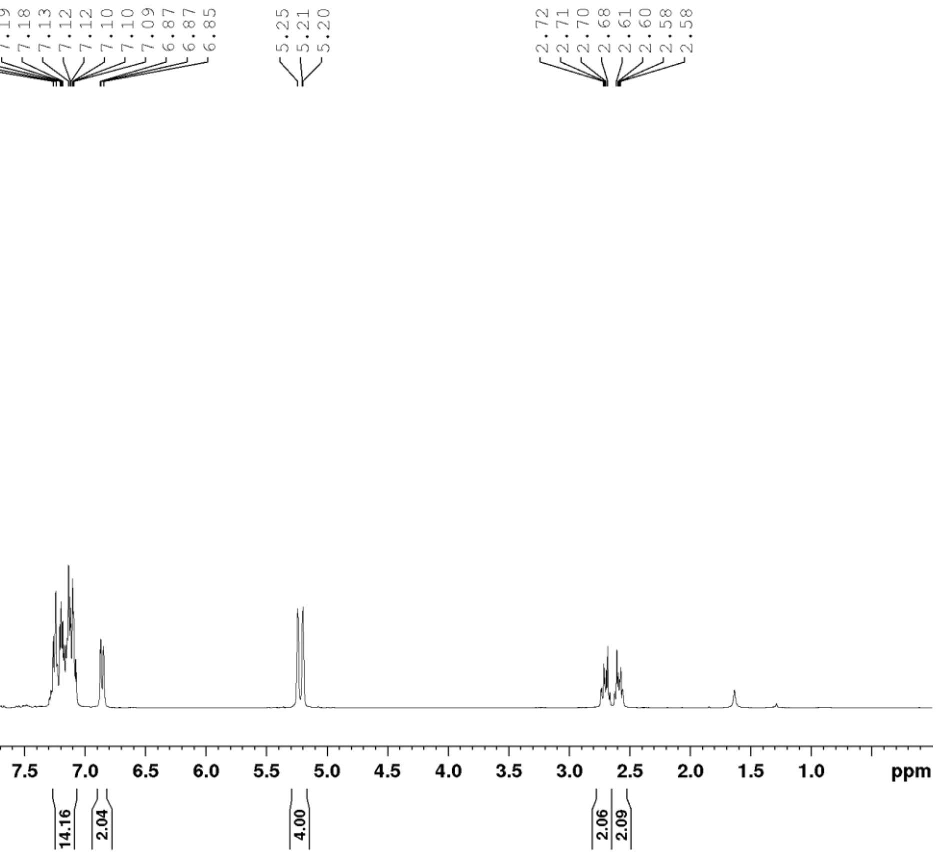

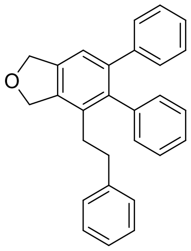

36

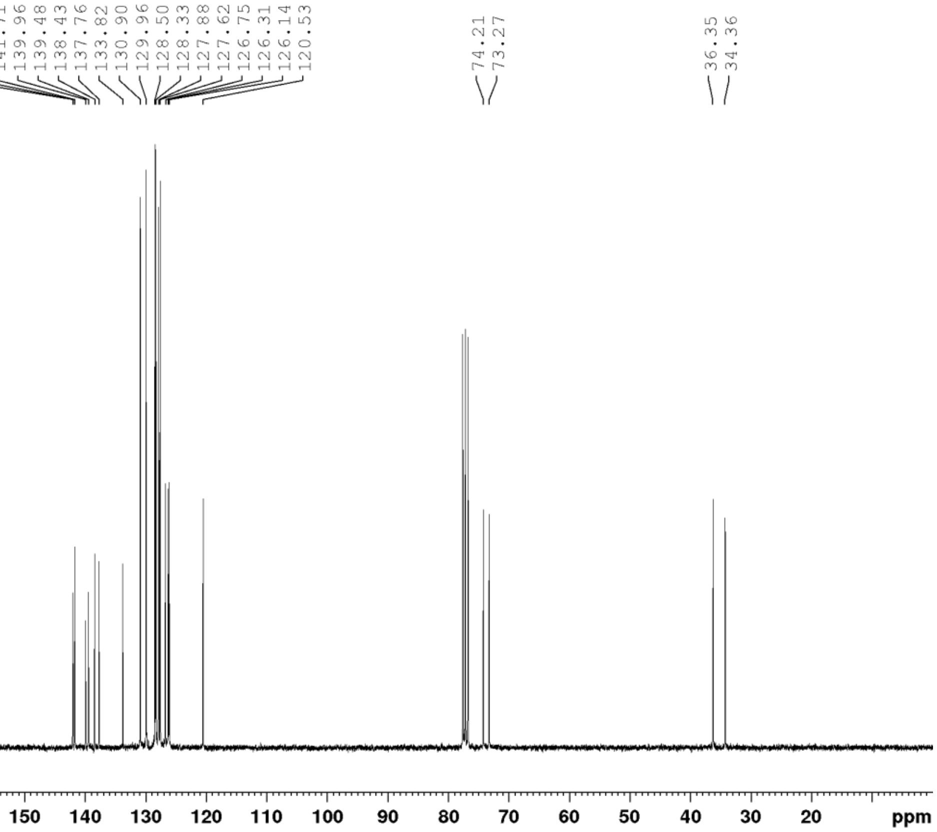

Copies of ^{1}H and ^{13}C NMR spectra of **37**

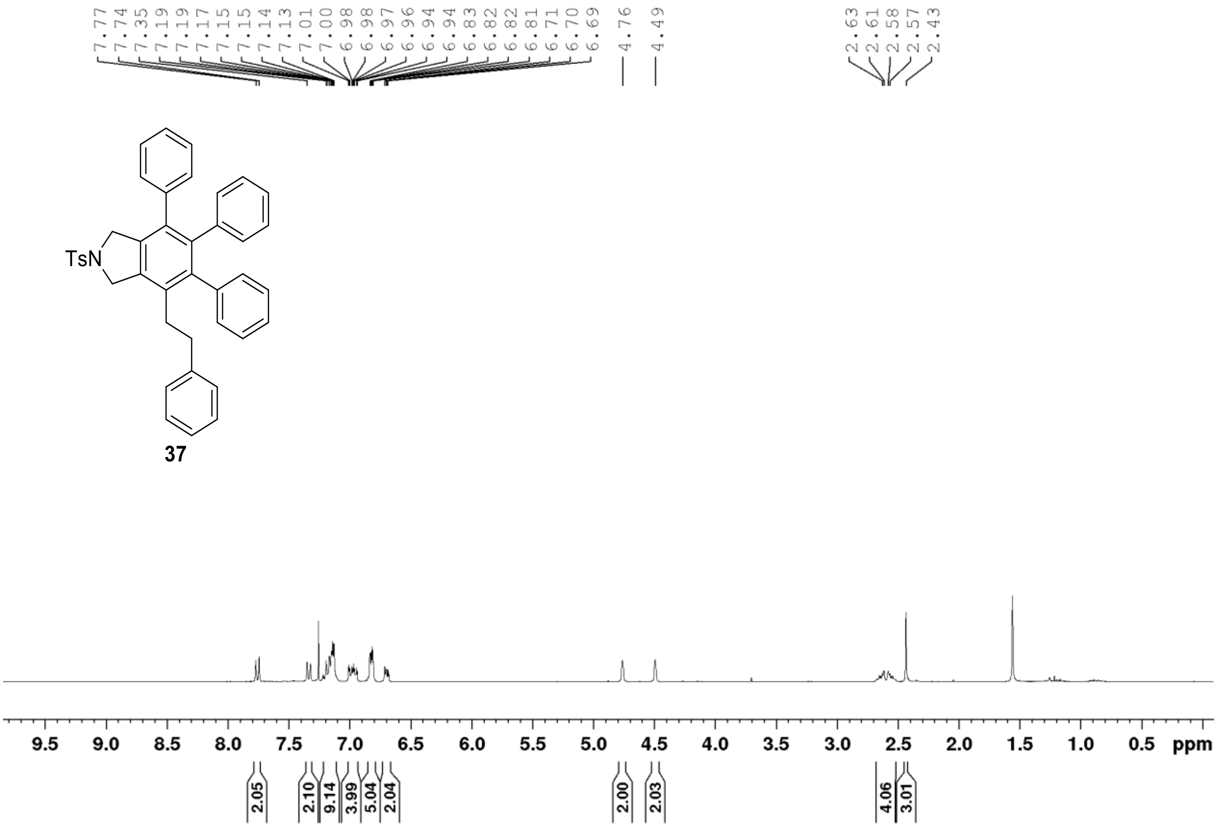

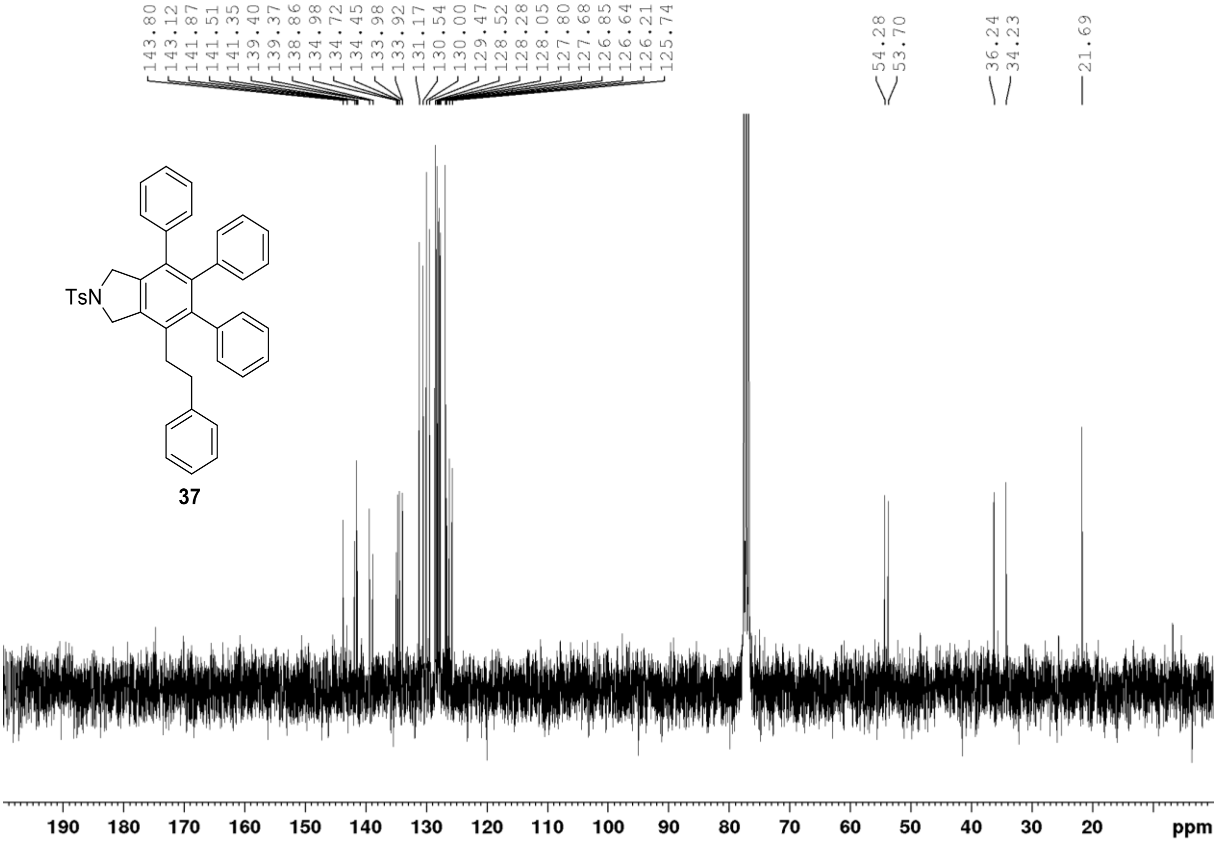

Copies of ^{1}H and ^{13}C NMR spectra of **38**

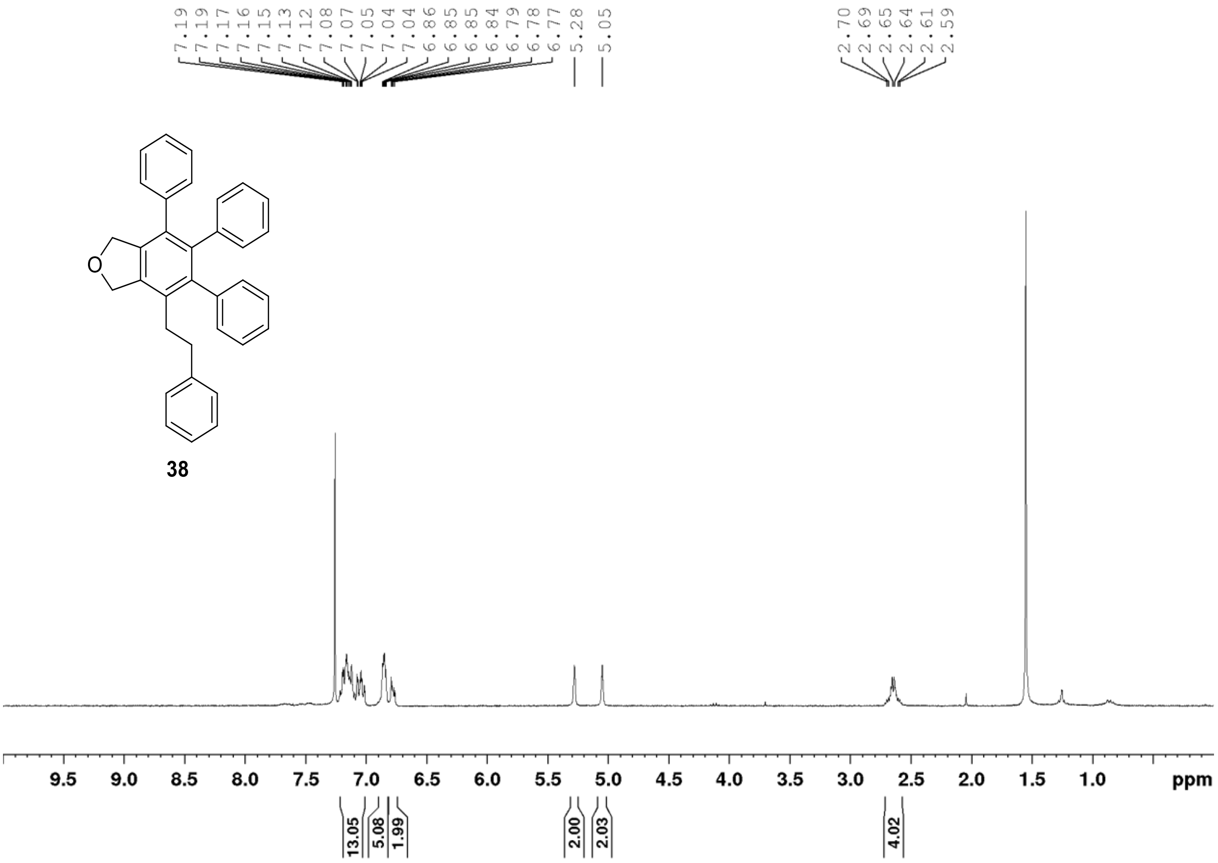

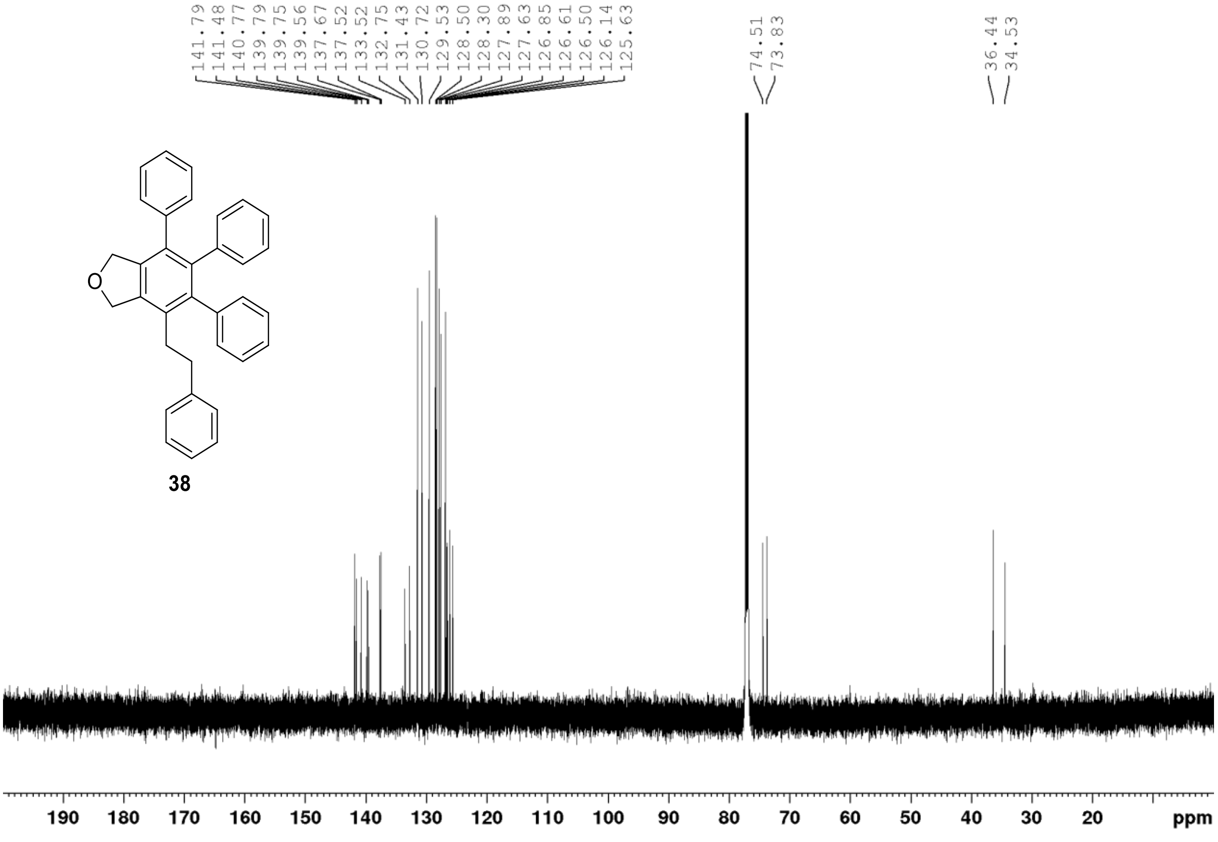

Copies of ^{1}H and ^{13}C NMR spectra of **39**

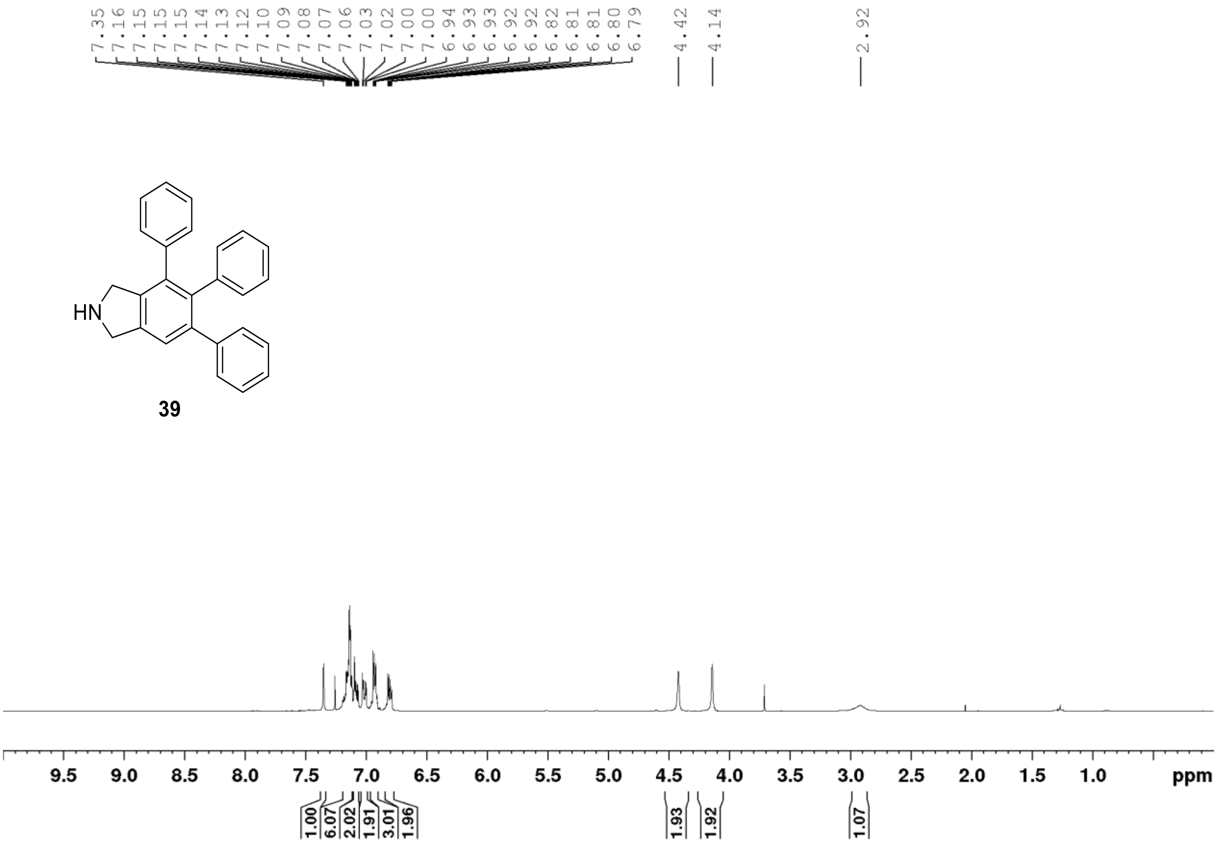

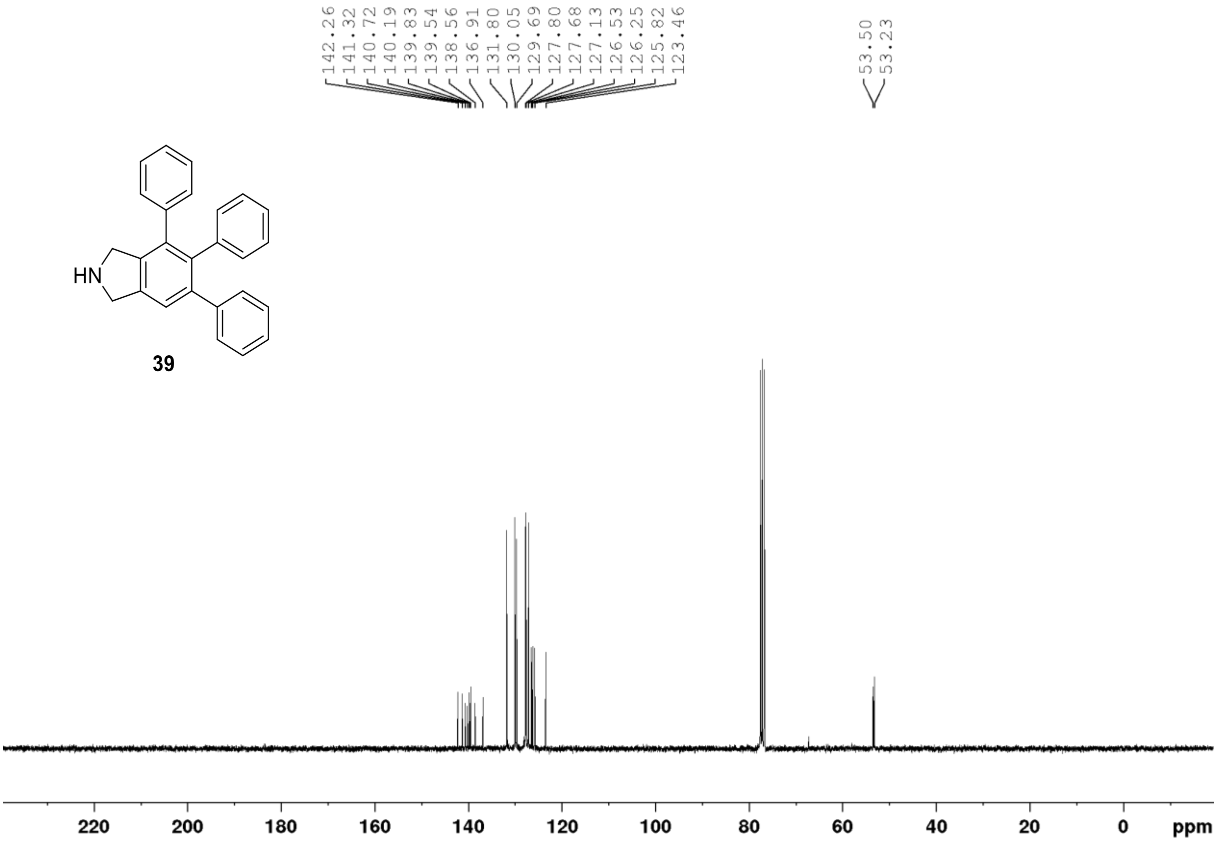

Copies of ^{1}H and ^{13}C NMR spectra of **40**

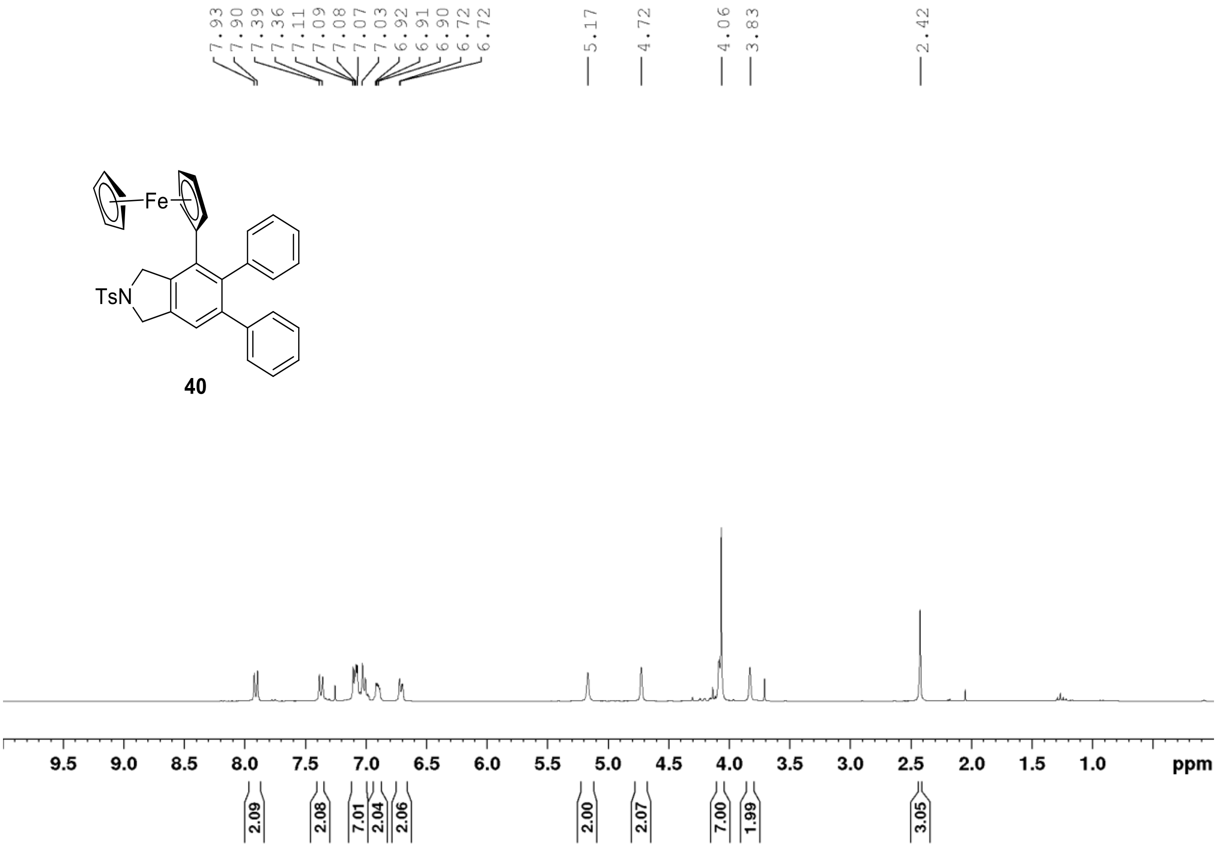

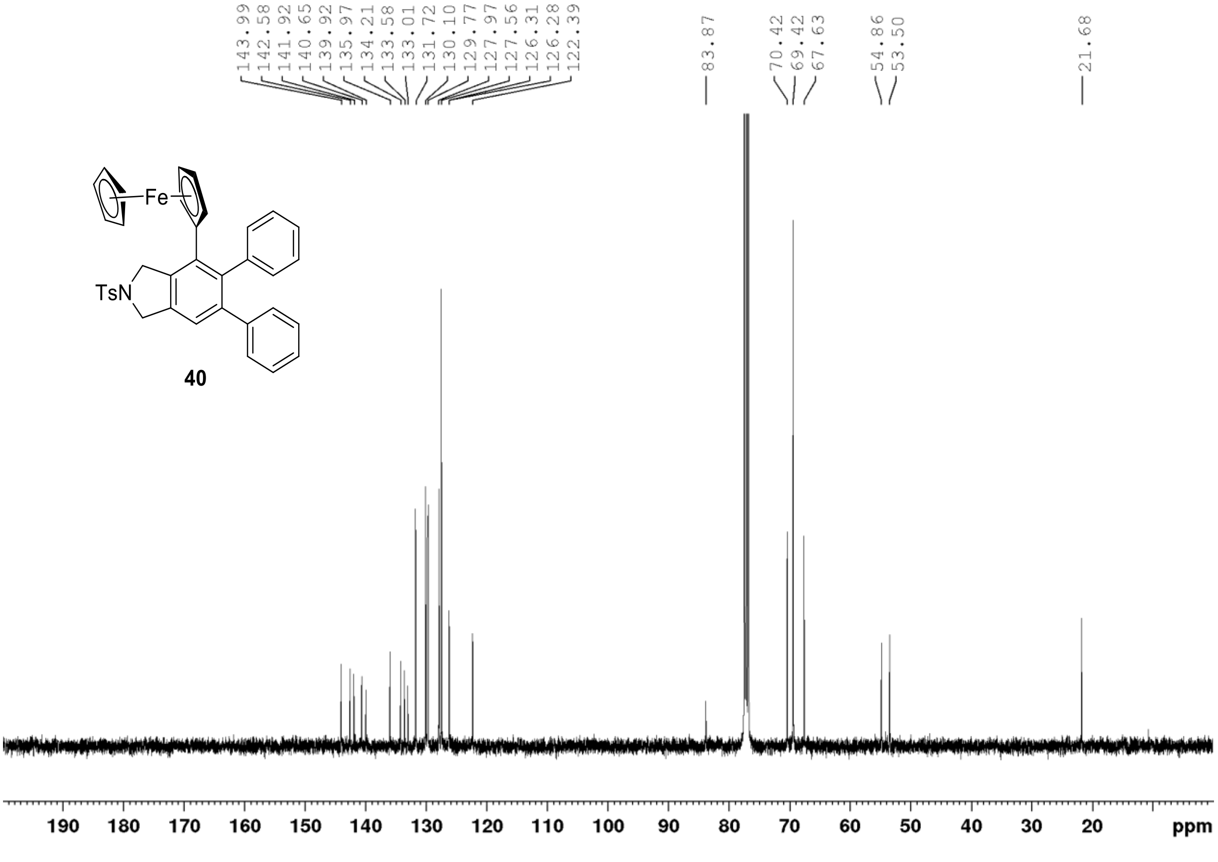

Copies of ^{1}H and ^{13}C NMR spectra of **41**

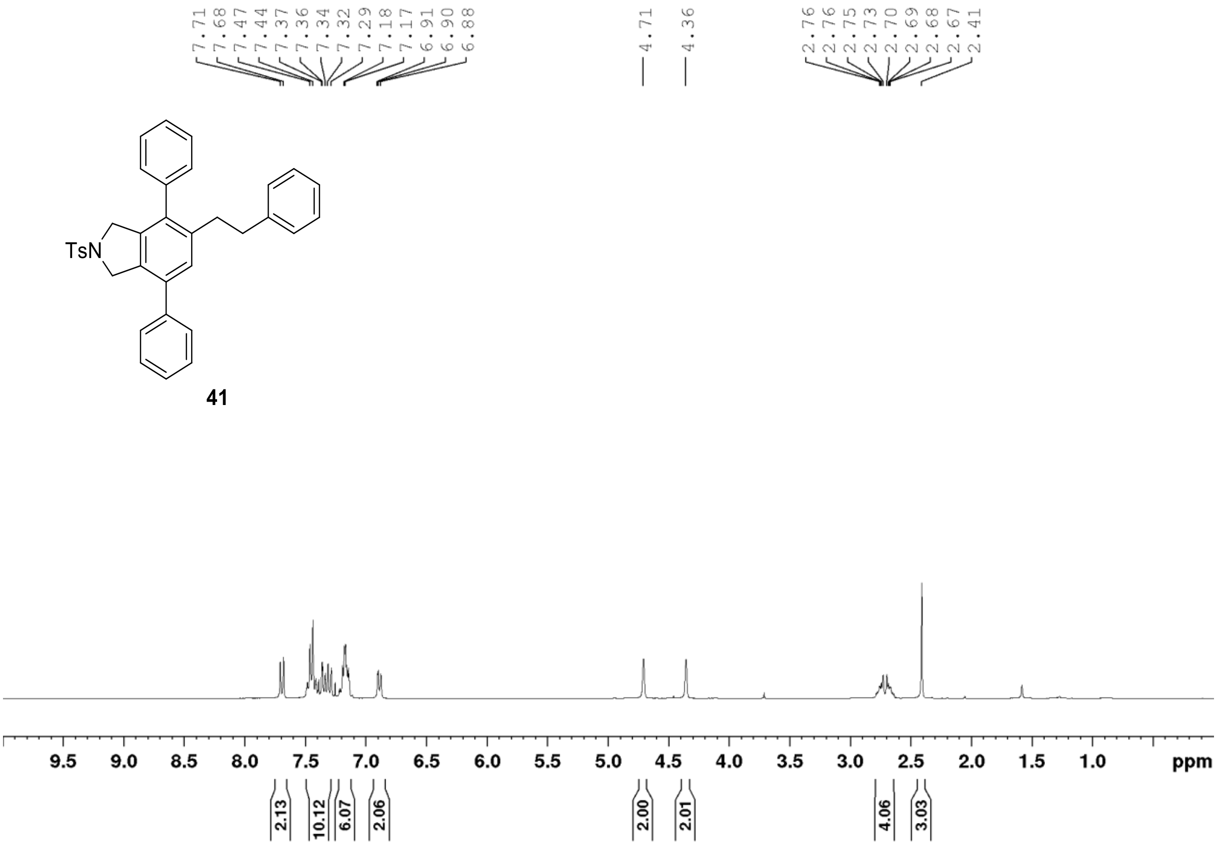

41

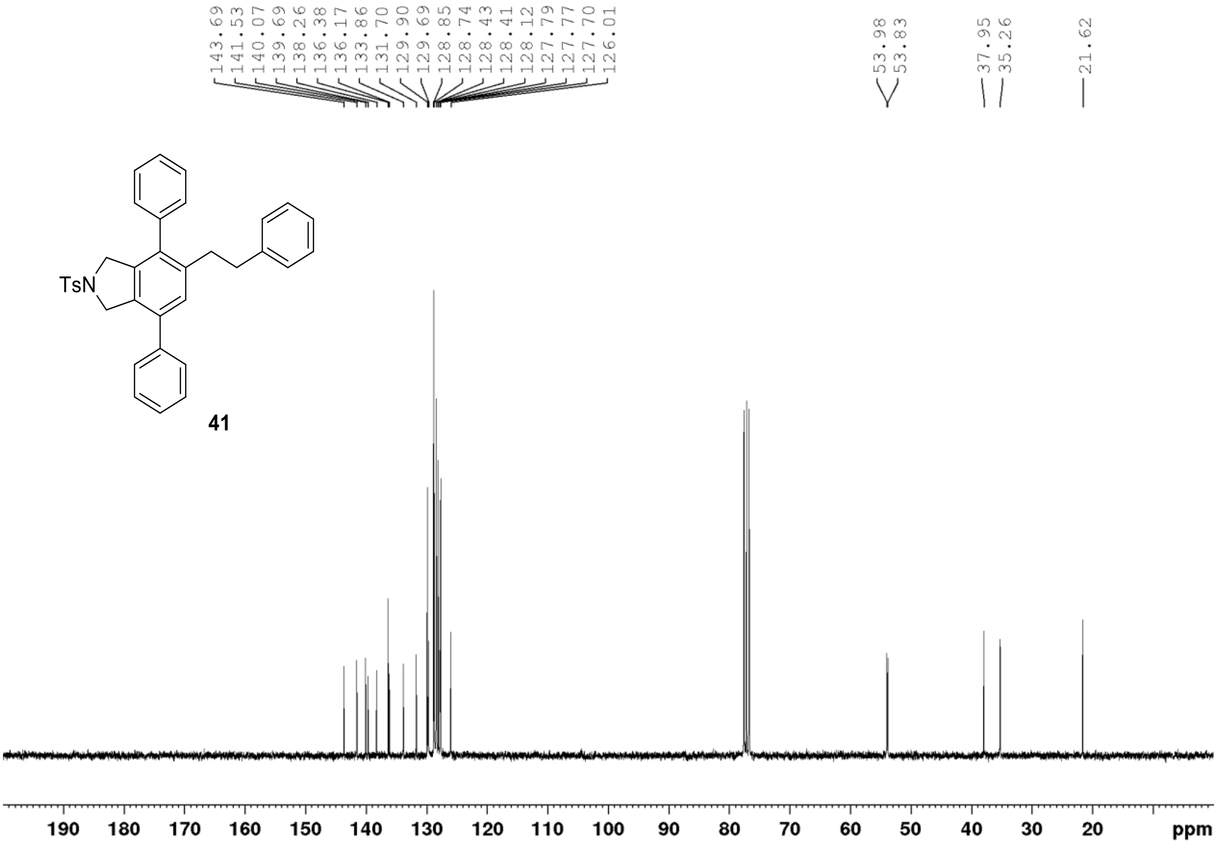

41

Copies of ^{1}H and ^{13}C NMR spectra of **42**

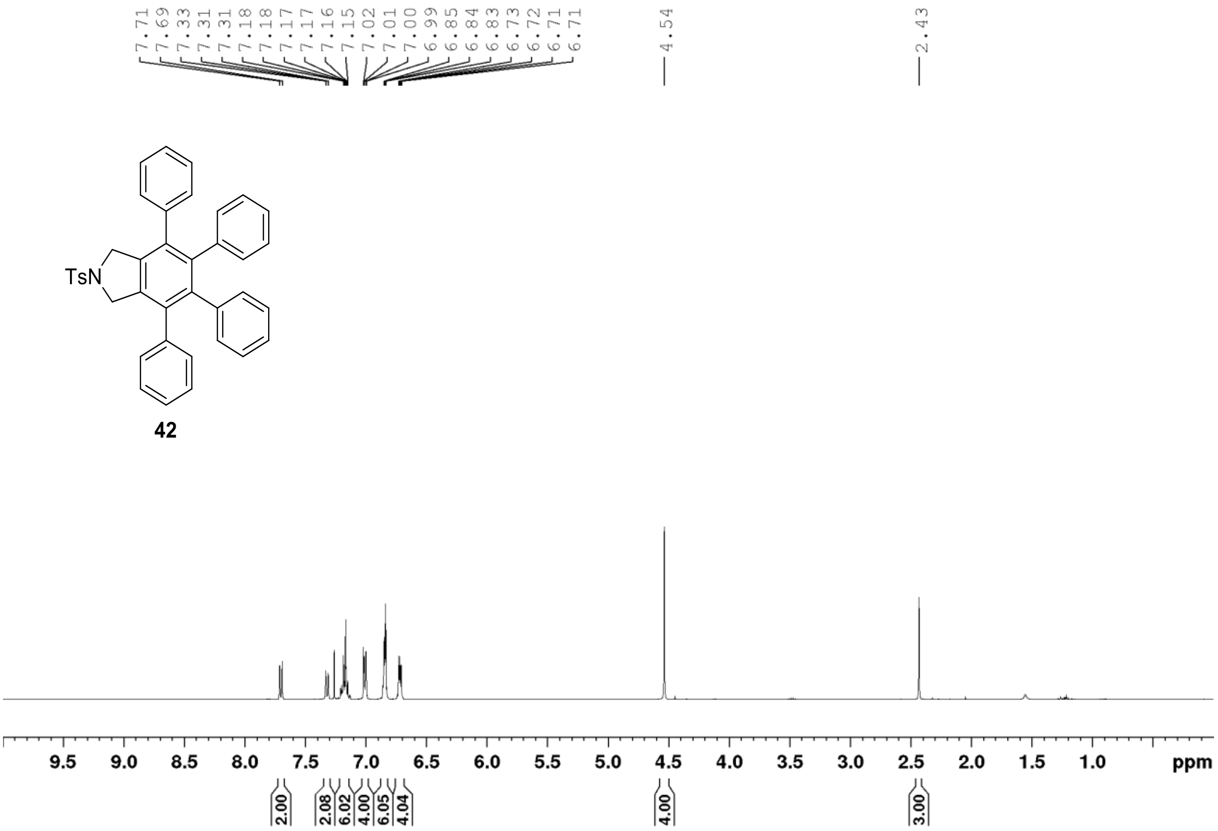

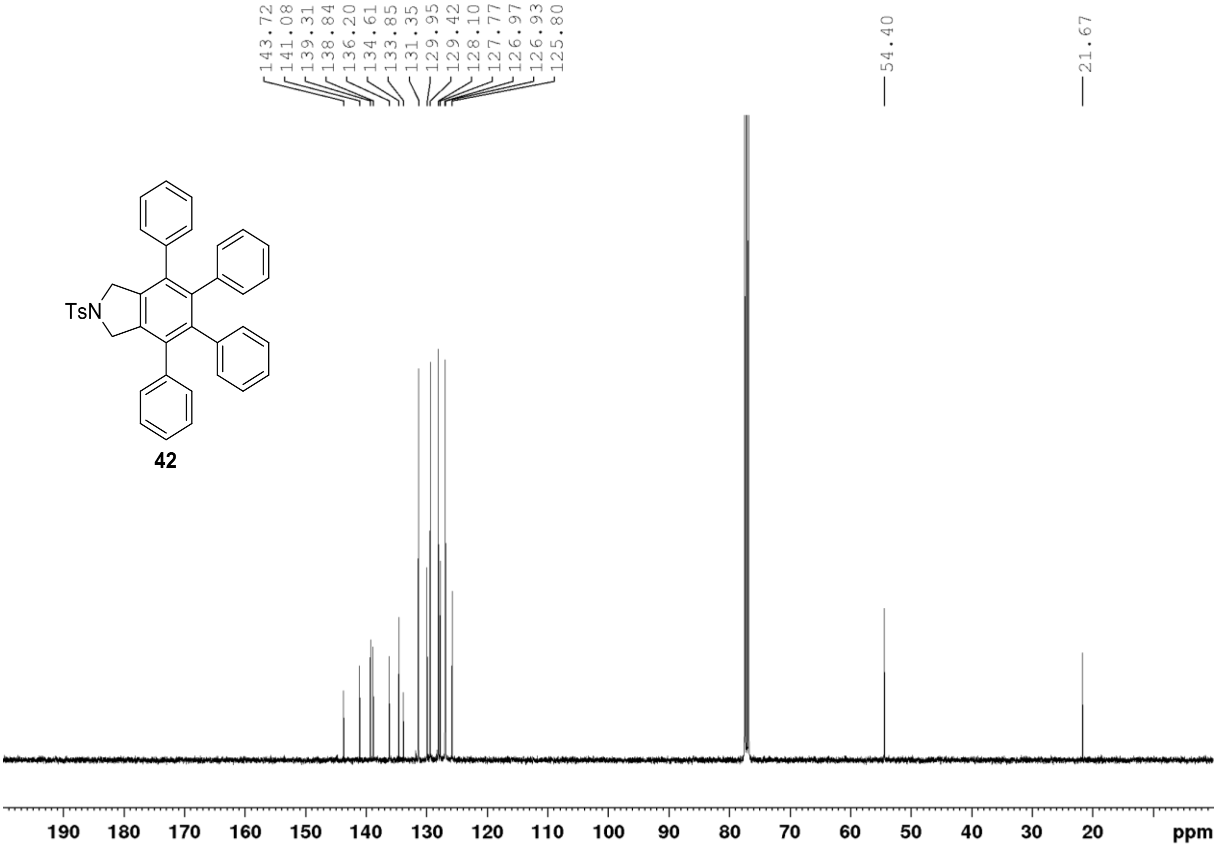

Copies of ^{1}H and ^{13}C NMR spectra of **43**

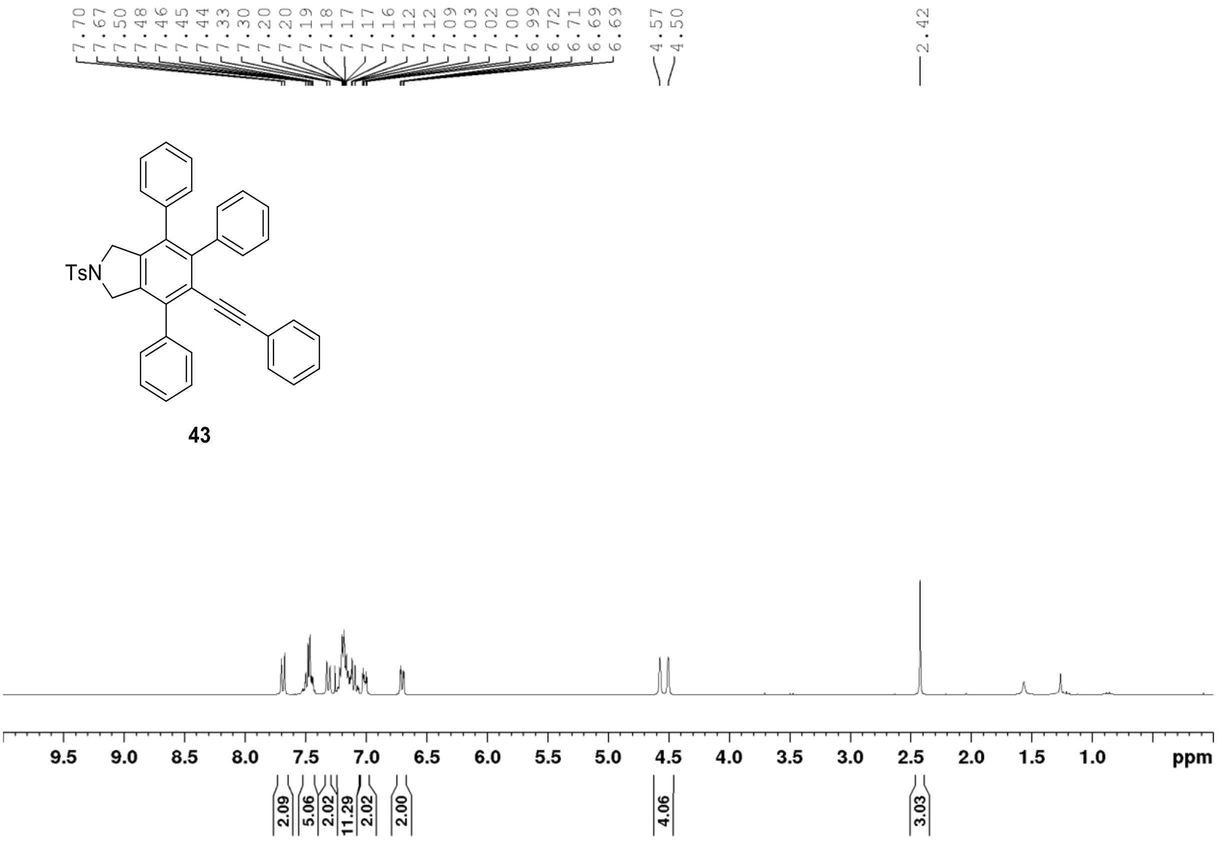

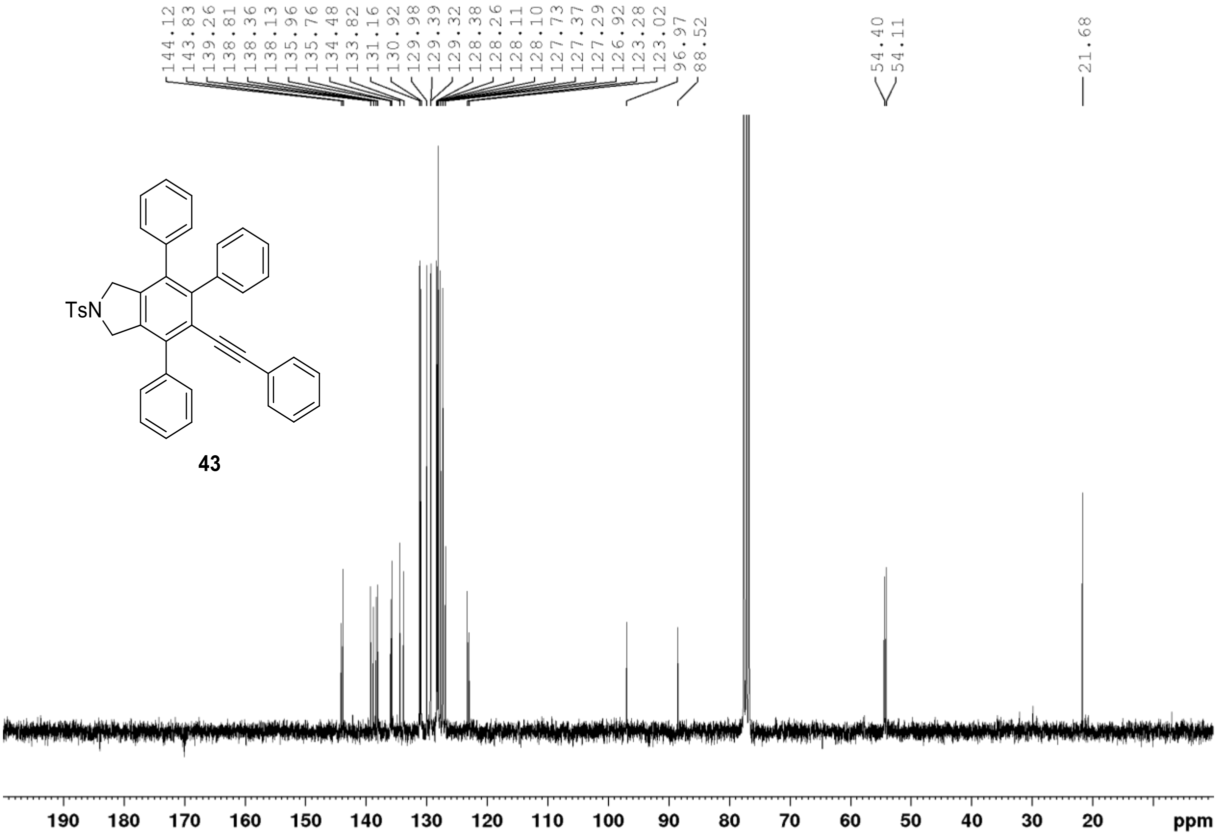

Copies of ^{1}H and ^{13}C NMR spectra of **44**

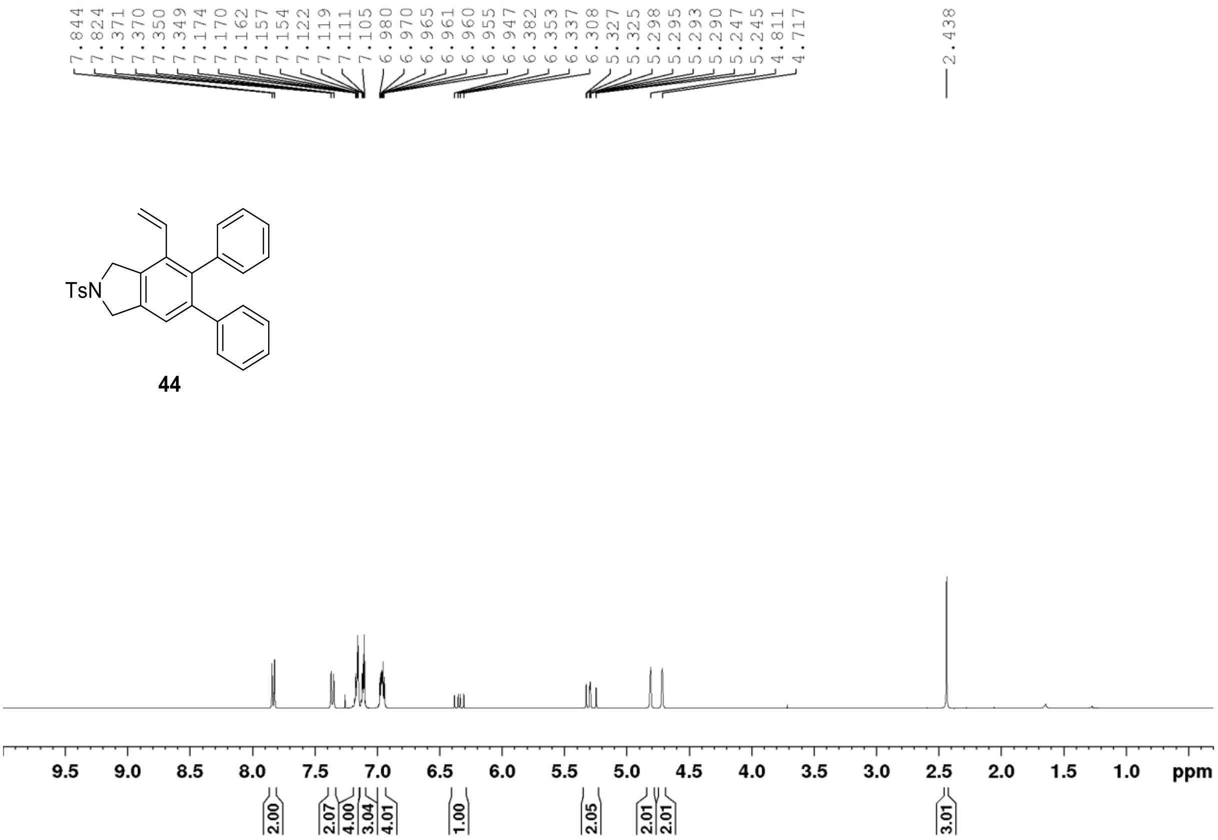

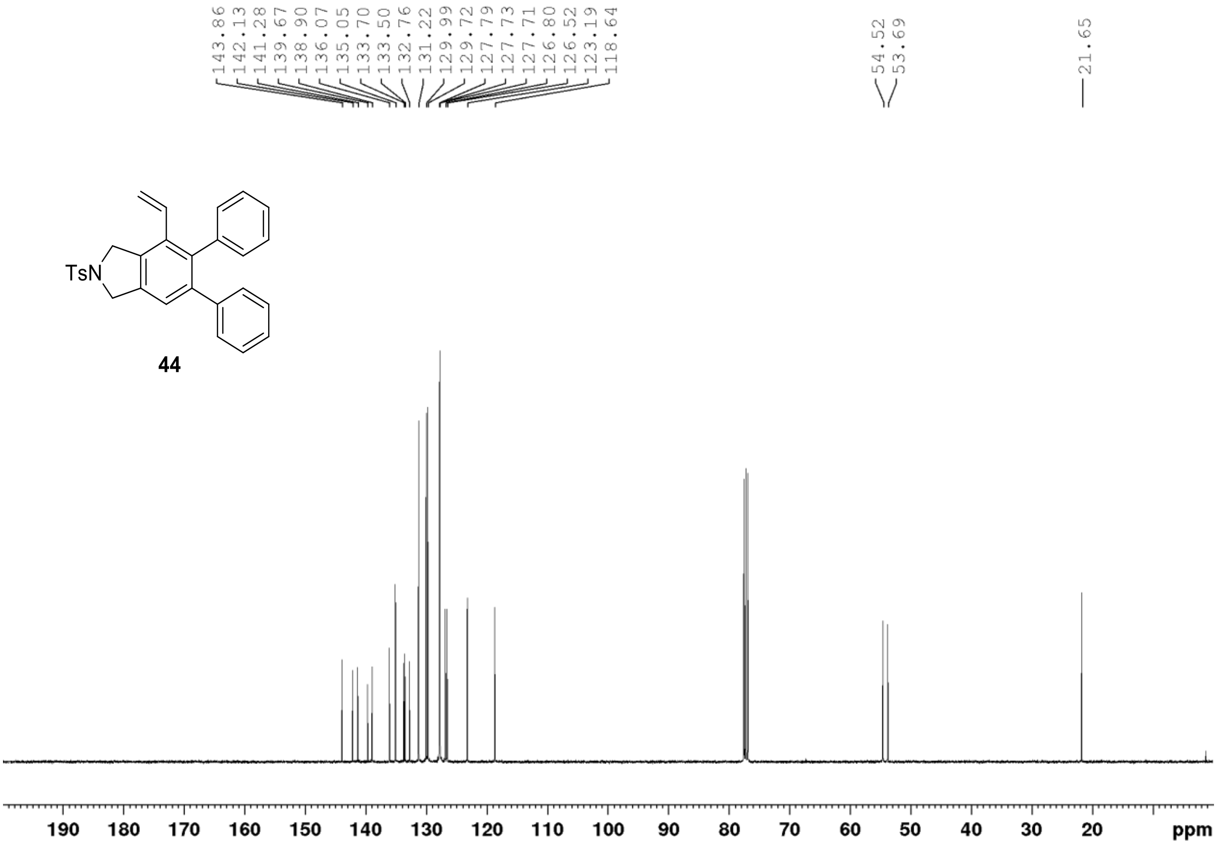

Copies of ^{1}H and ^{13}C NMR spectra of **45**

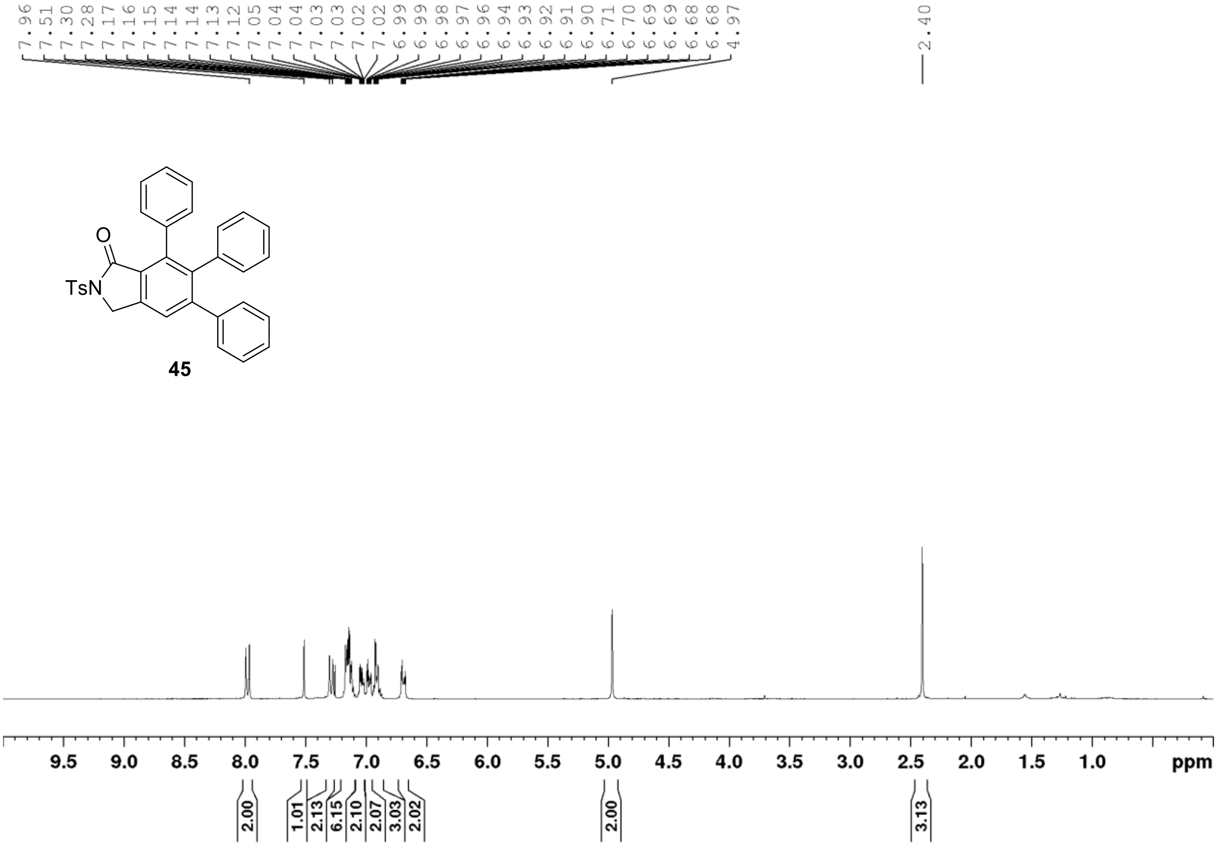

45

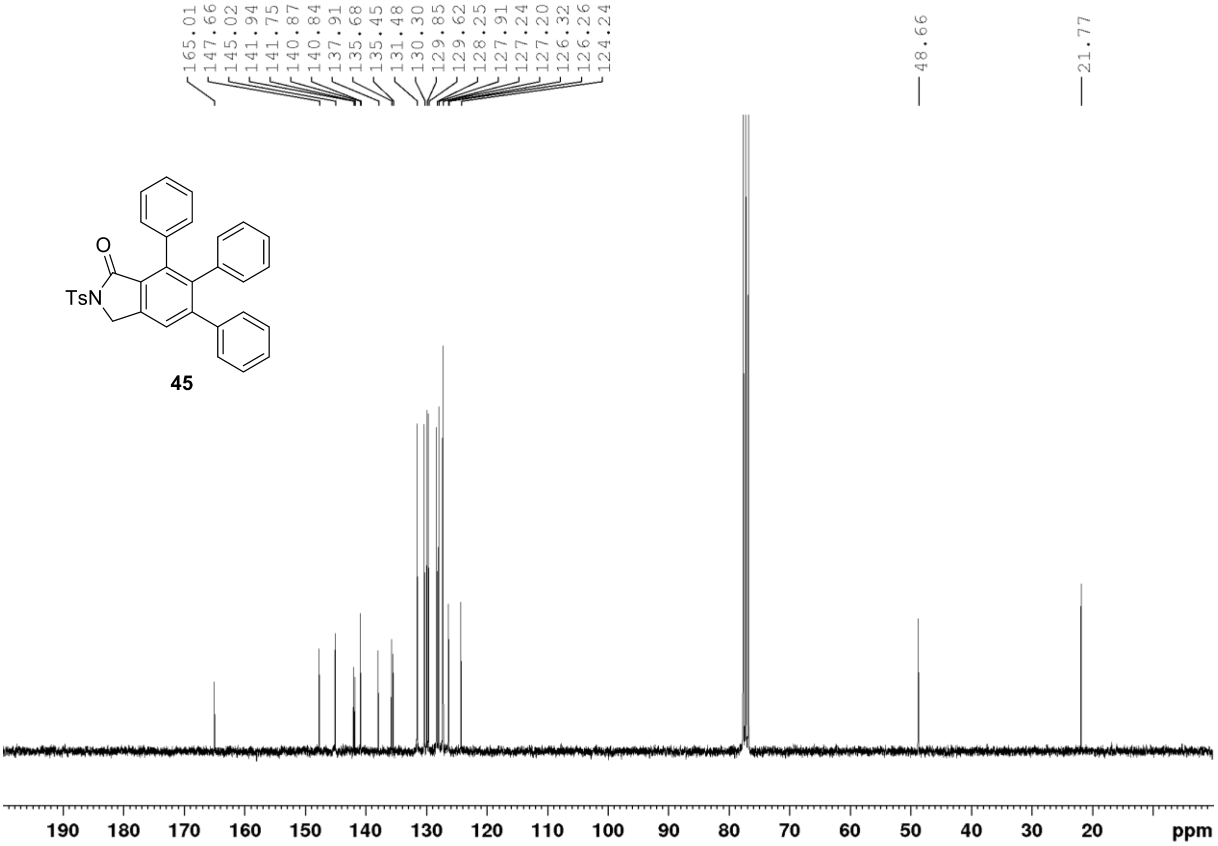

45

Copies of ^{1}H and ^{13}C NMR spectra of **46**

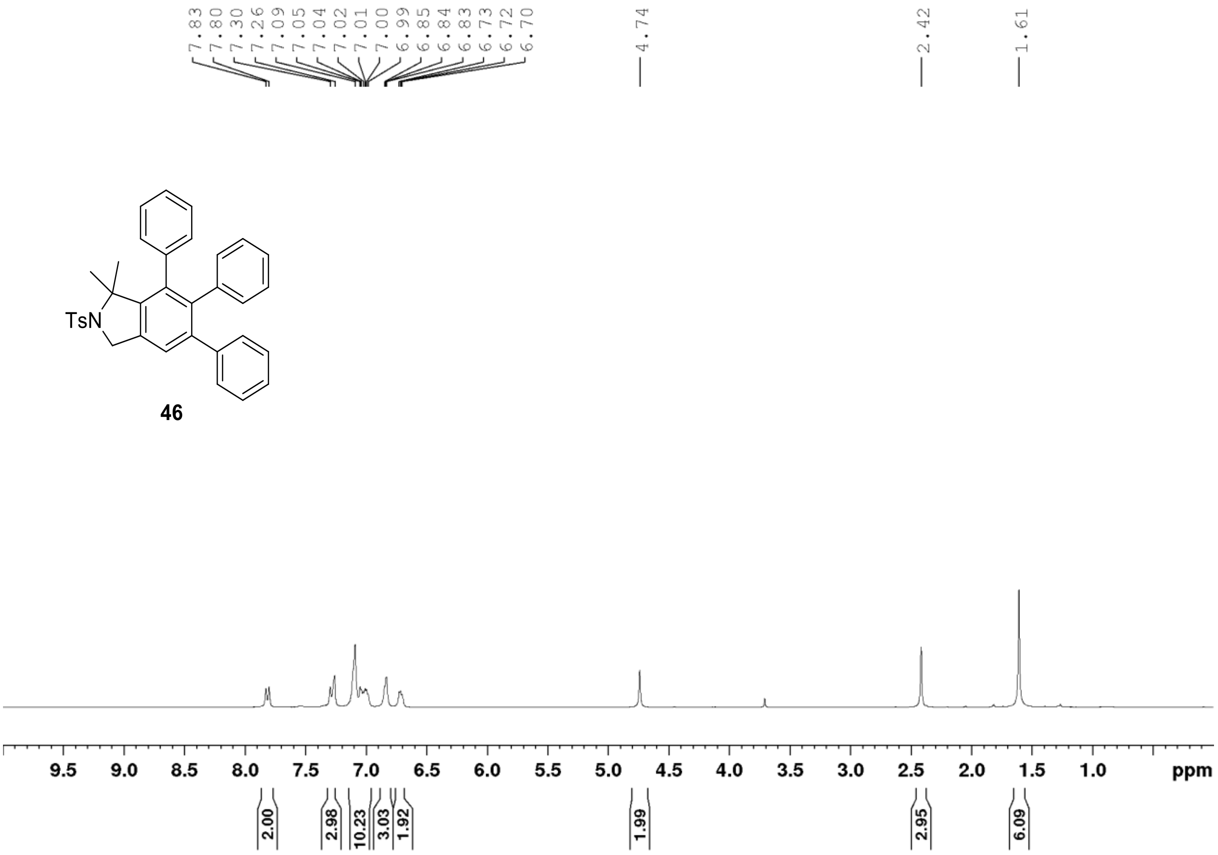

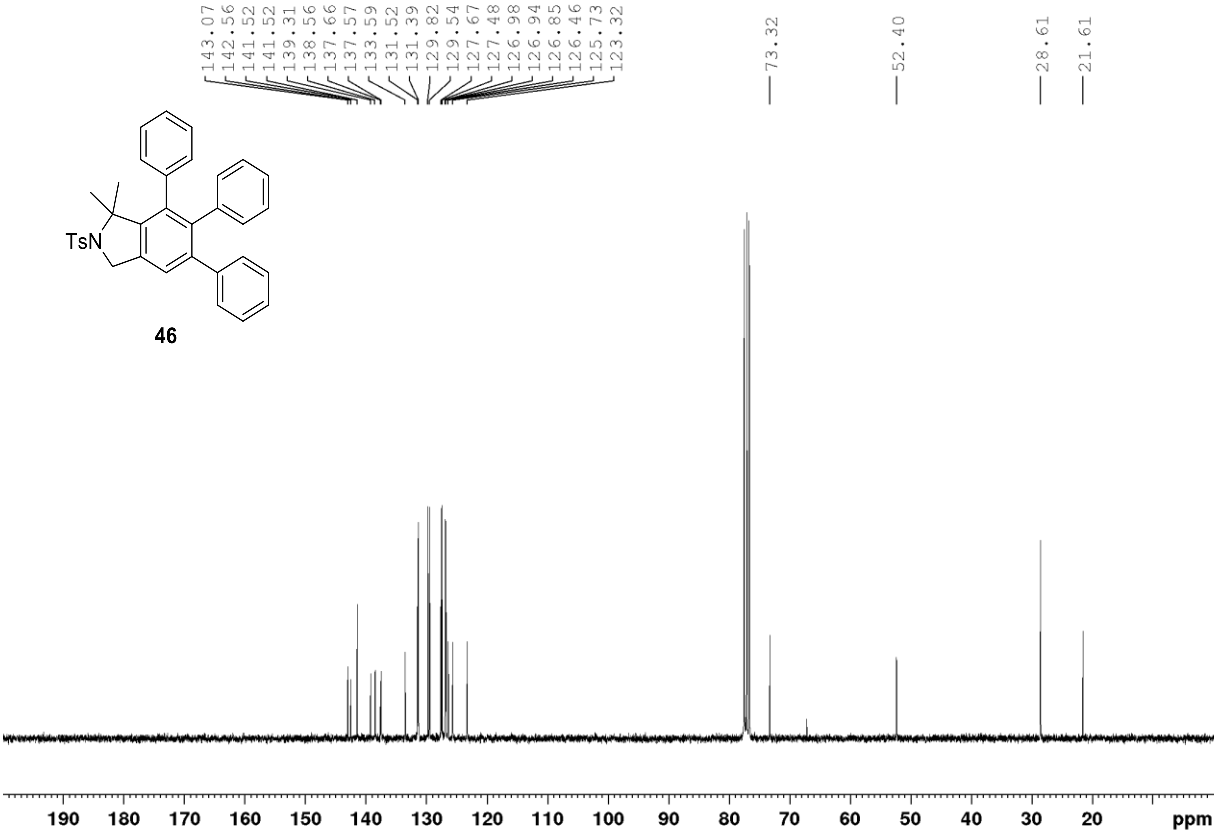

Copies of ^{1}H and ^{13}C NMR spectra of **47**

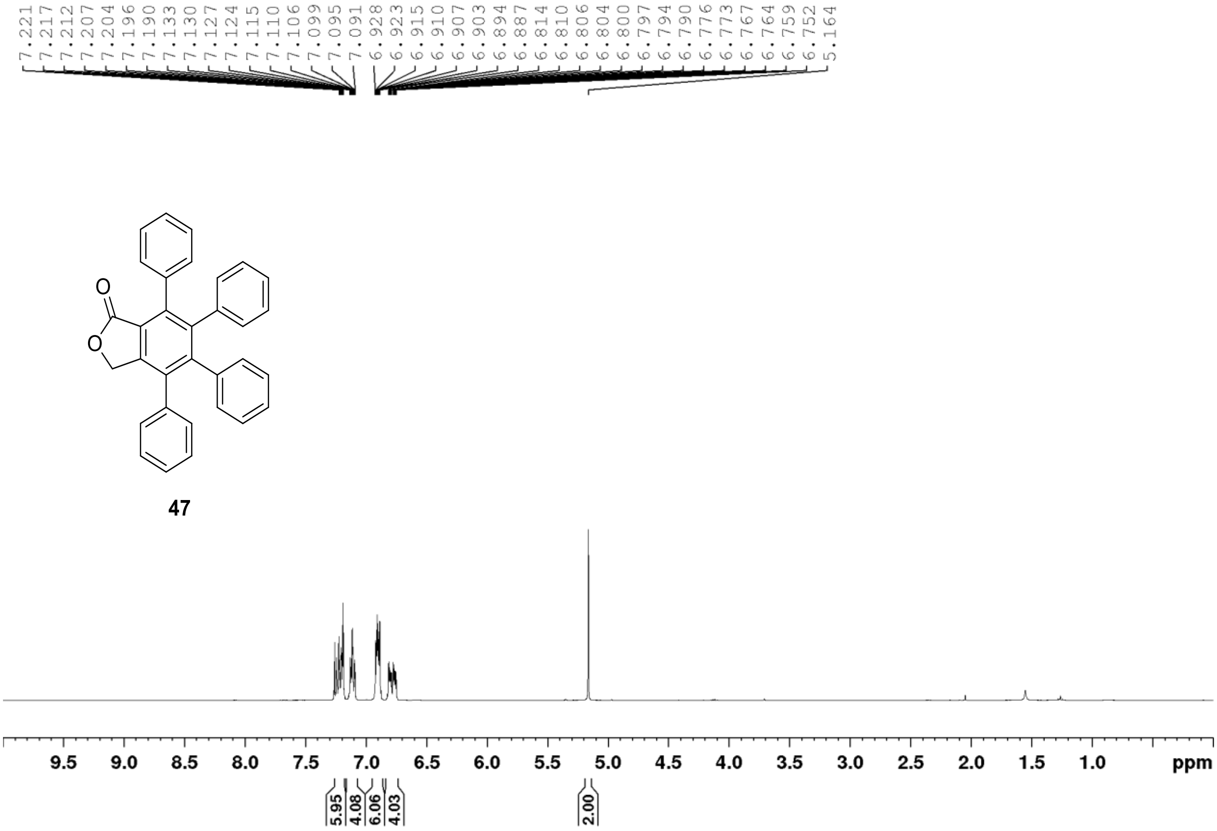

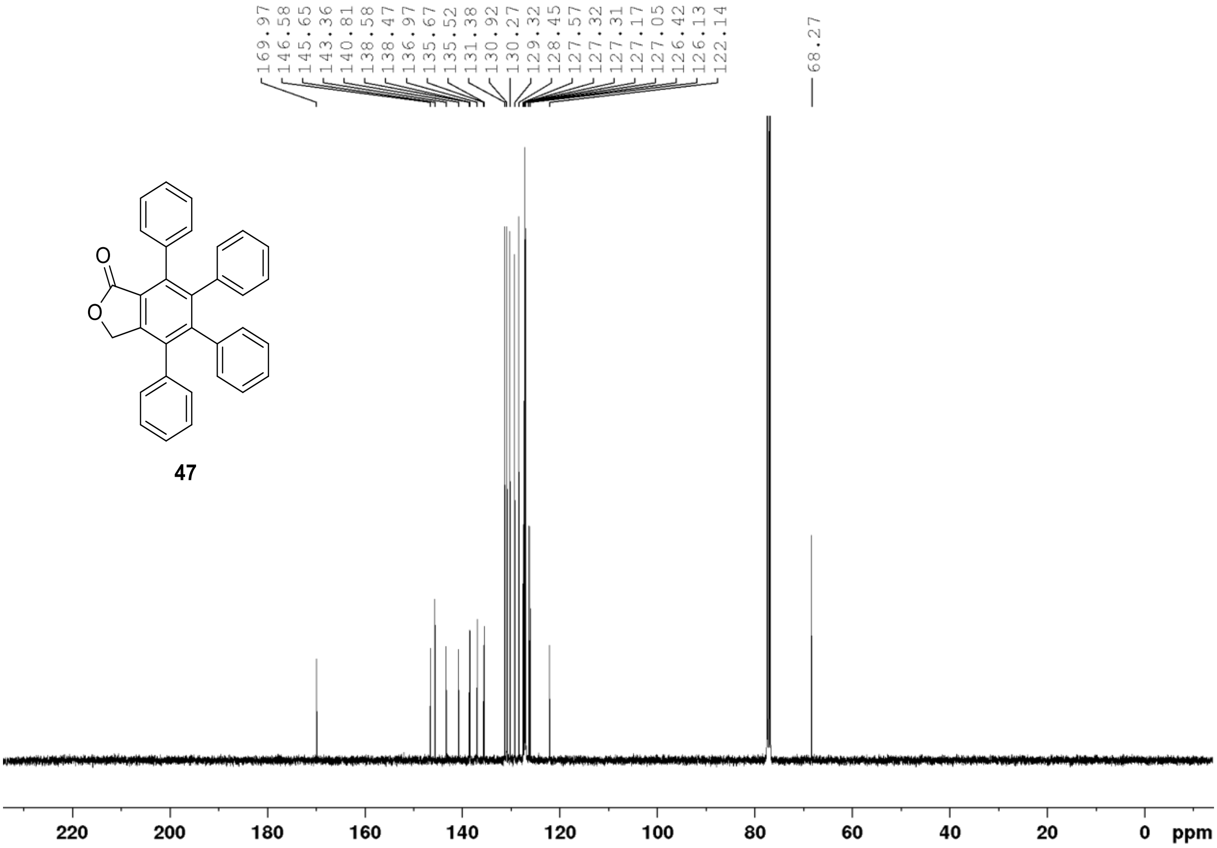

Copies of ^{1}H and ^{13}C NMR spectra of **48**

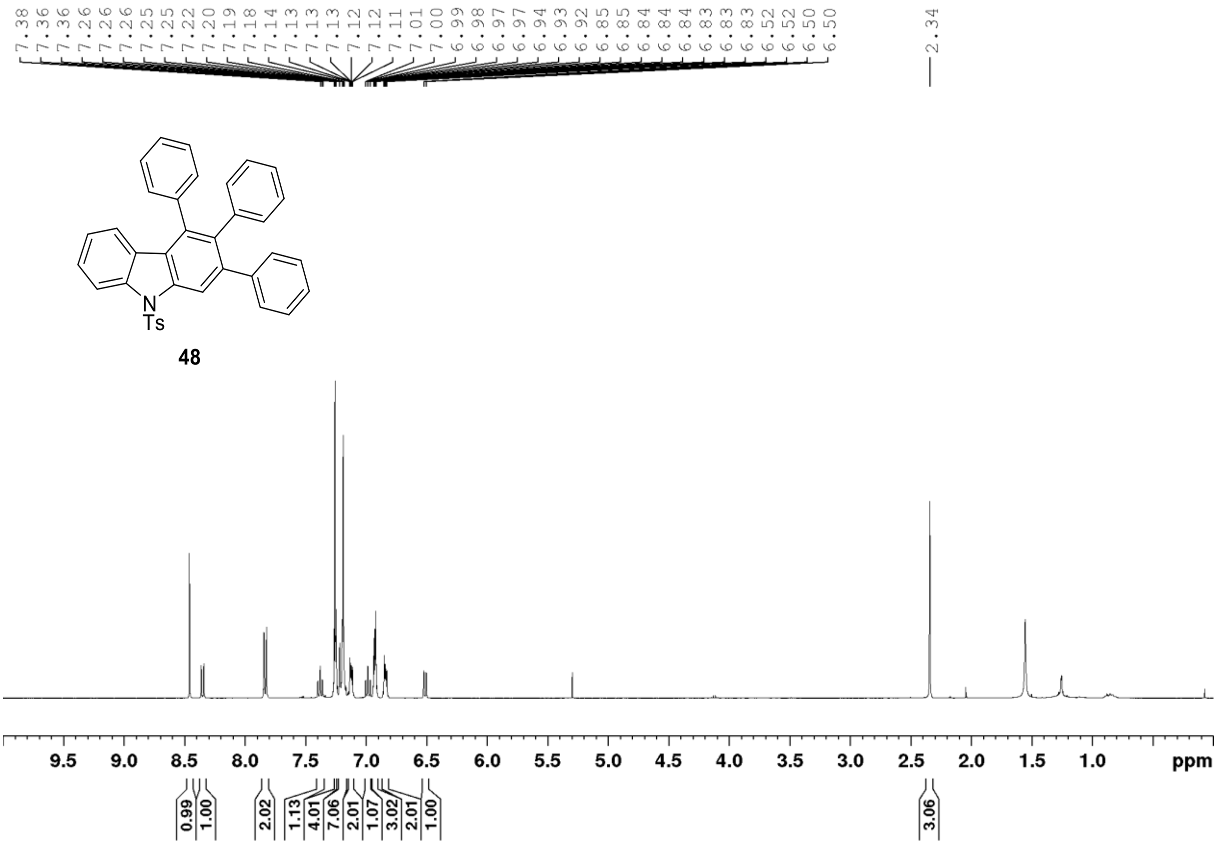

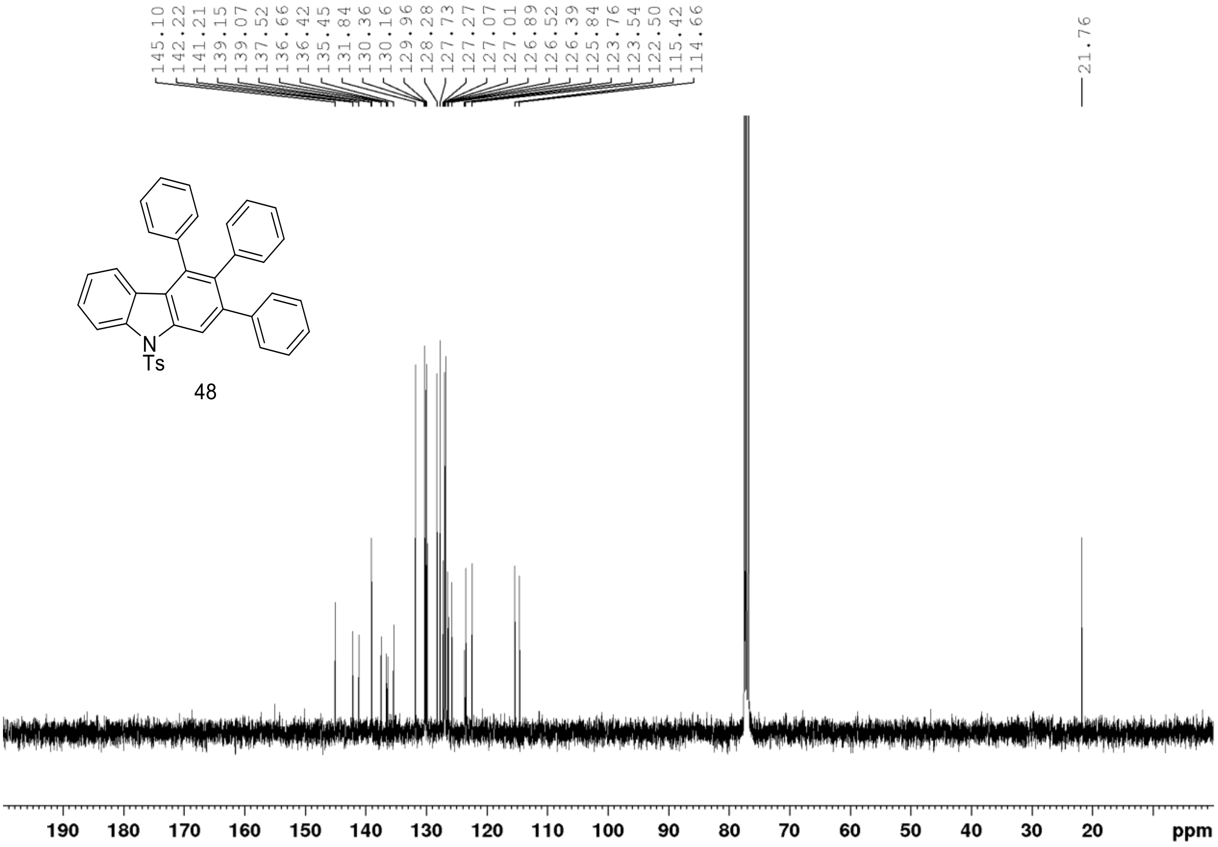

Copies of ^{1}H and ^{13}C NMR spectra of **49**

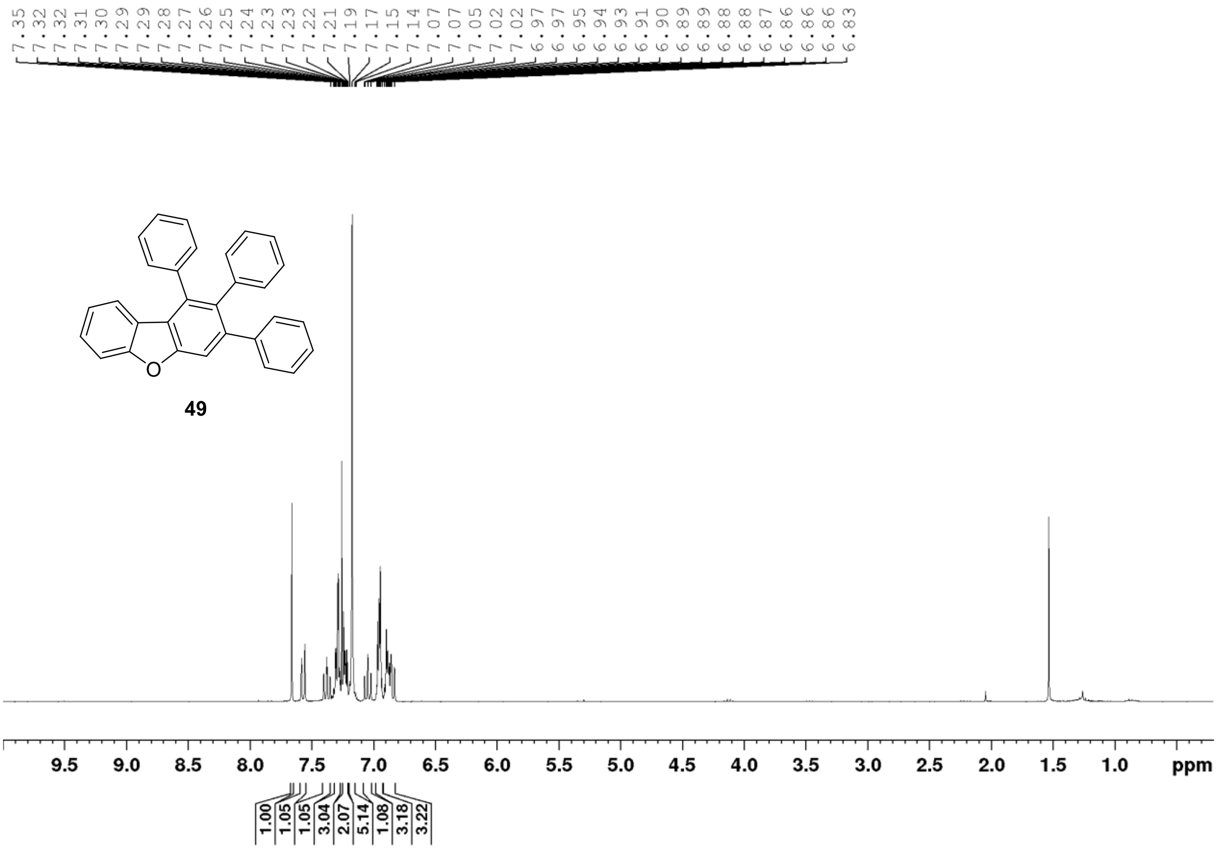

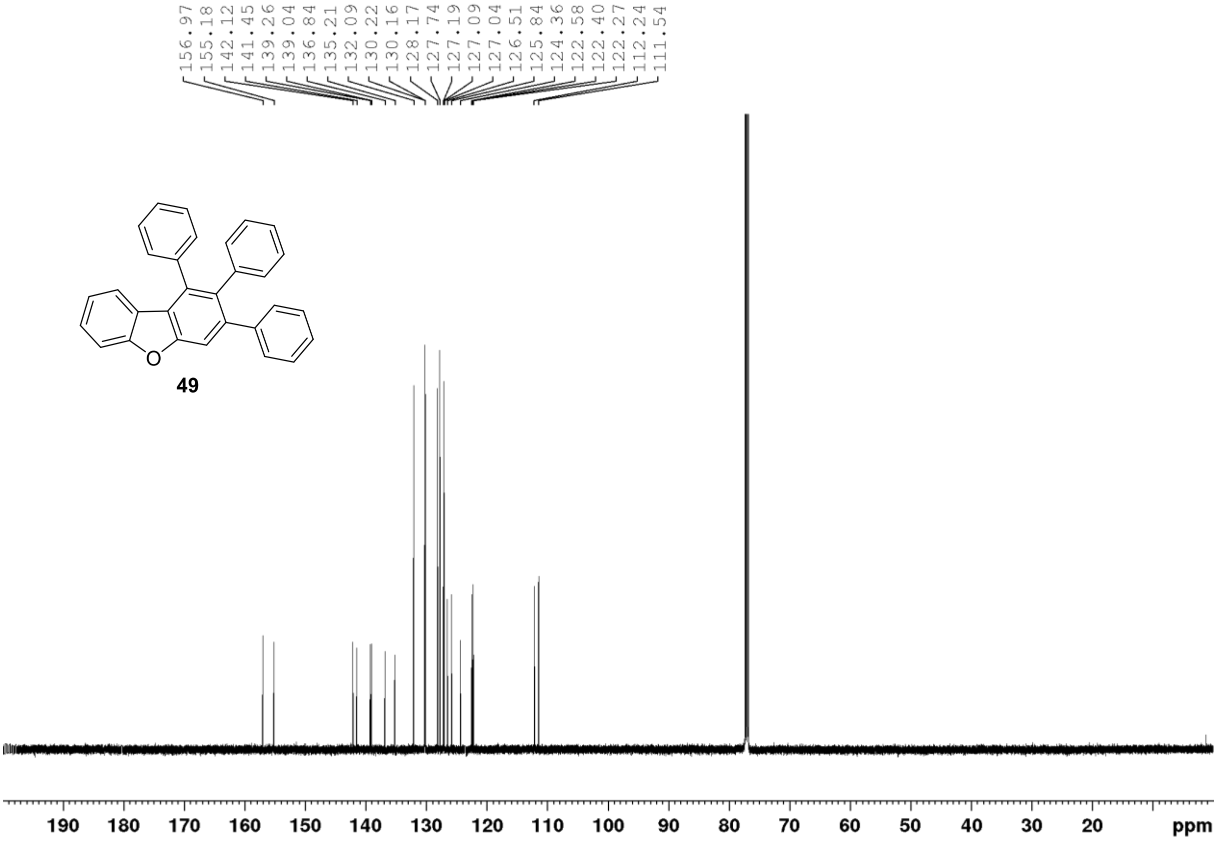

Copies of ^{1}H and ^{13}C NMR spectra of **50**

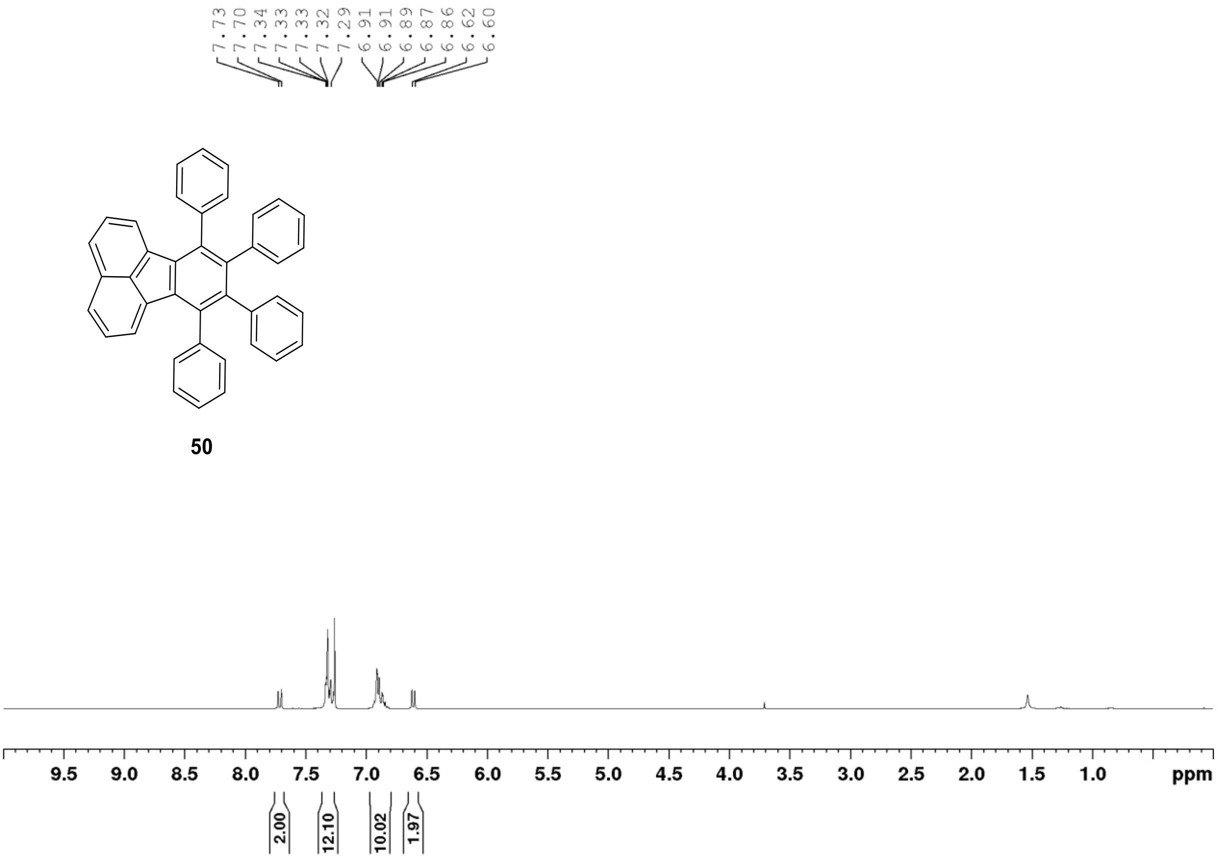

50

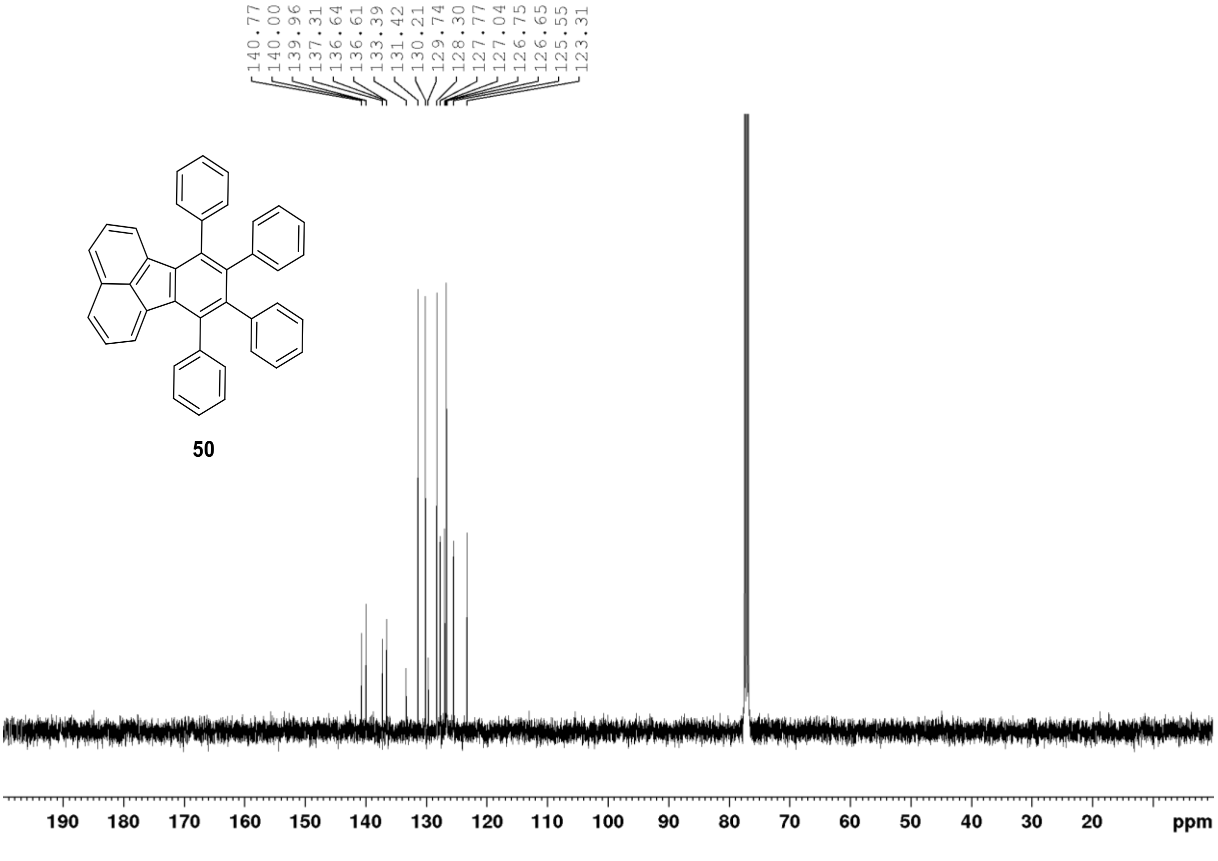

50

Copies of ^{1}H and ^{13}C NMR spectra of **51**

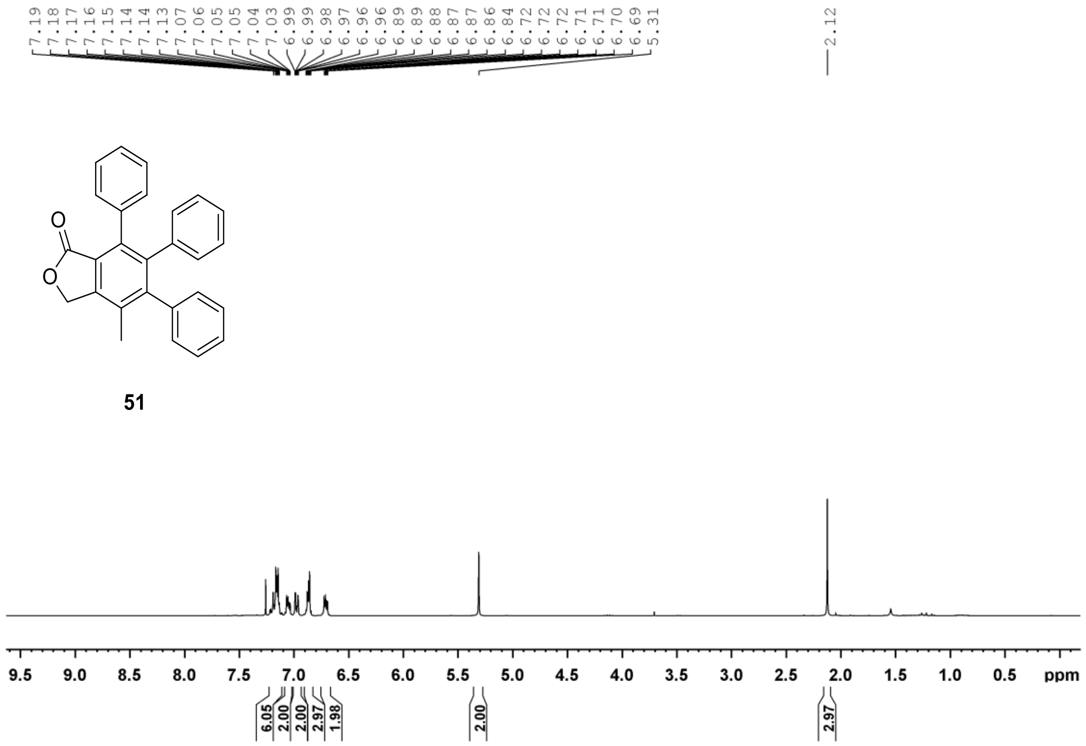

51

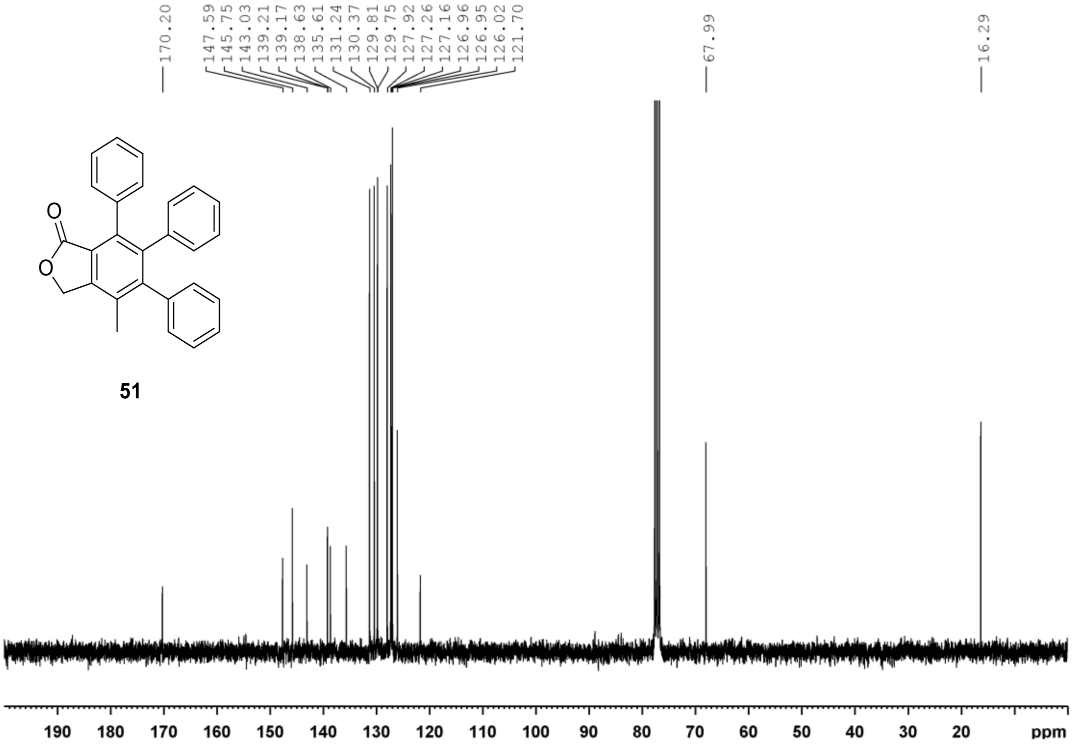

51

Copies of ^{1}H and ^{13}C NMR spectra of **52**

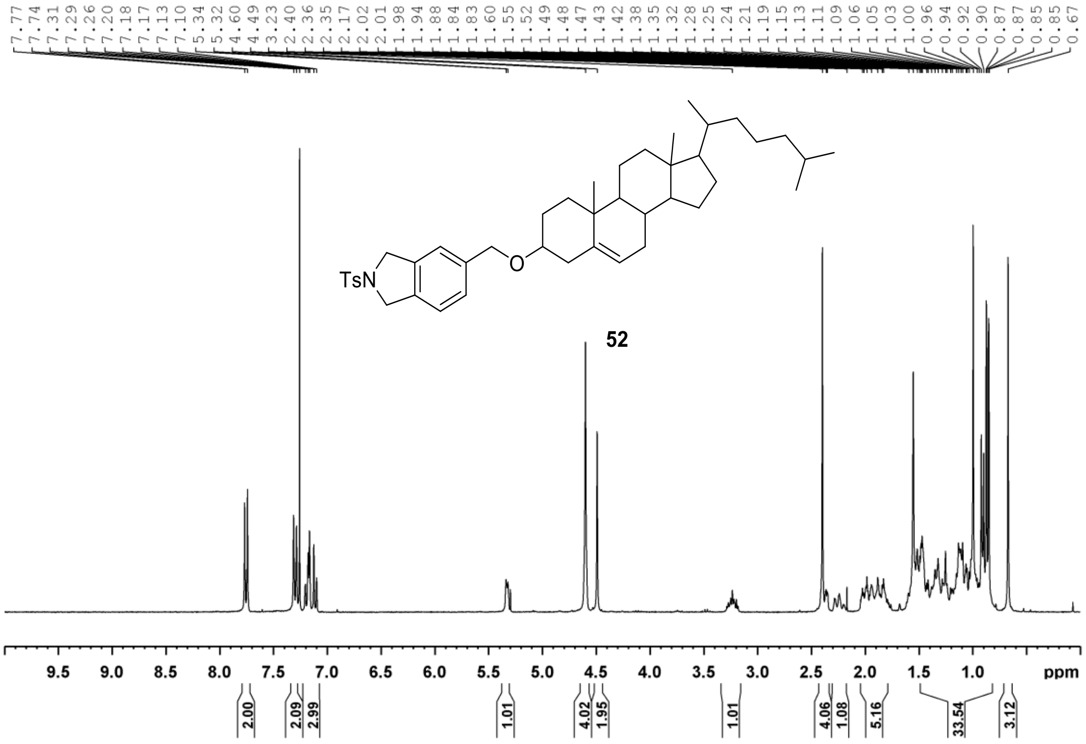

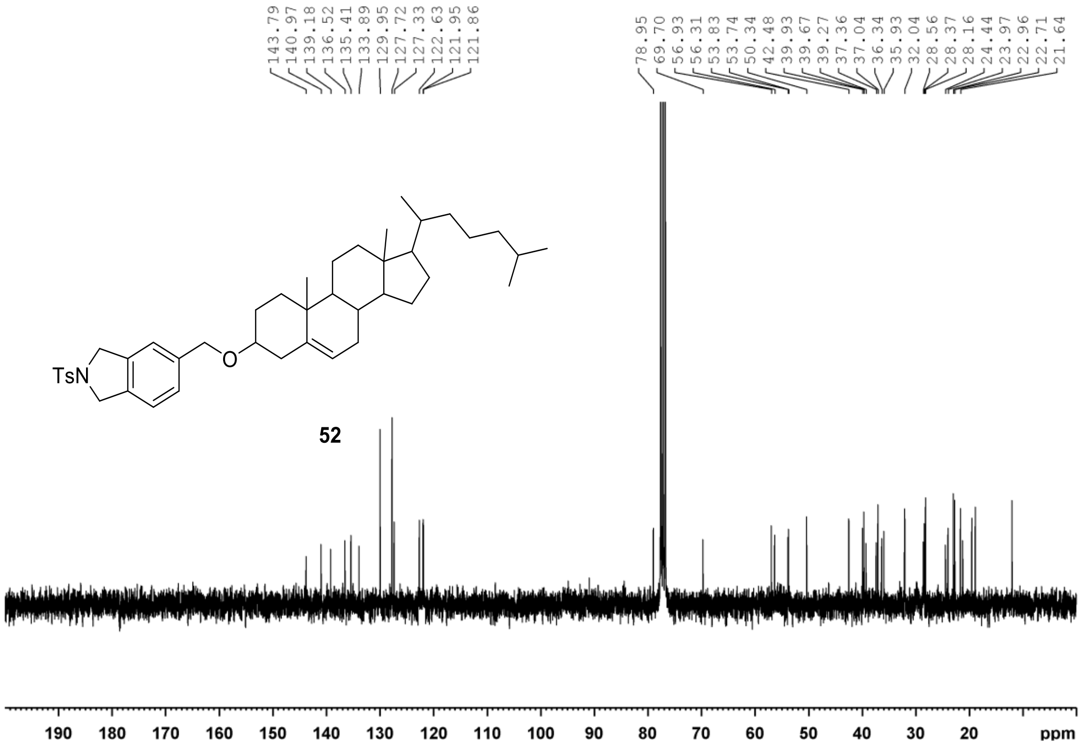

Copies of ^{1}H and ^{13}C NMR spectra of **53**

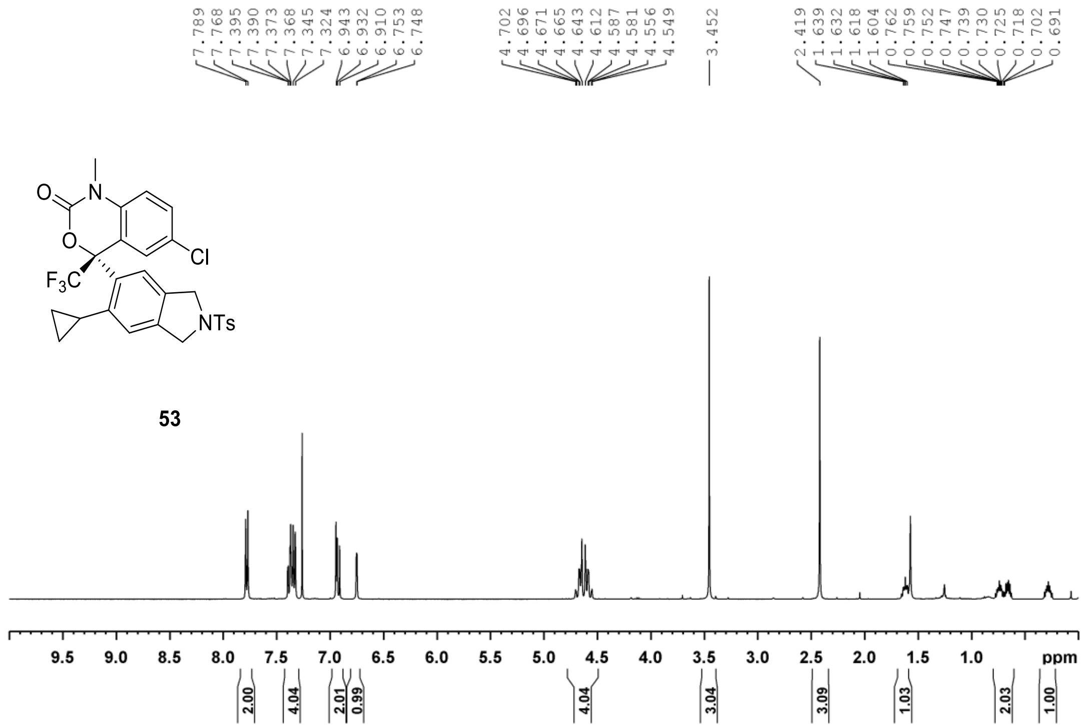

53

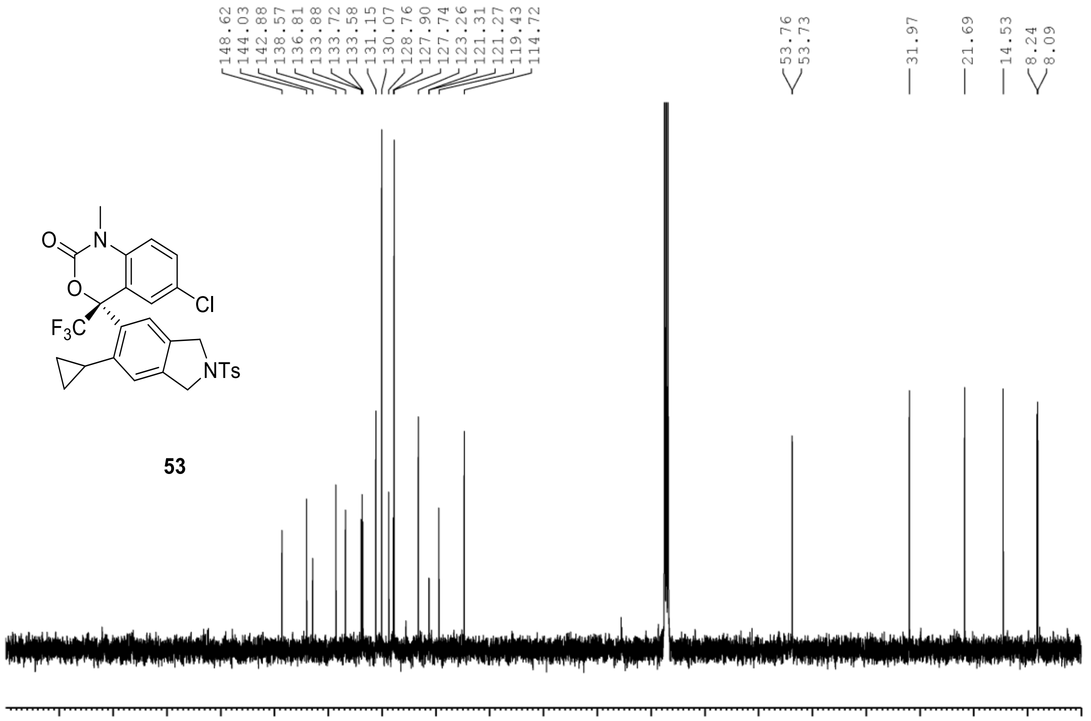

53

Copies of ^{1}H and ^{13}C NMR spectra of **54**

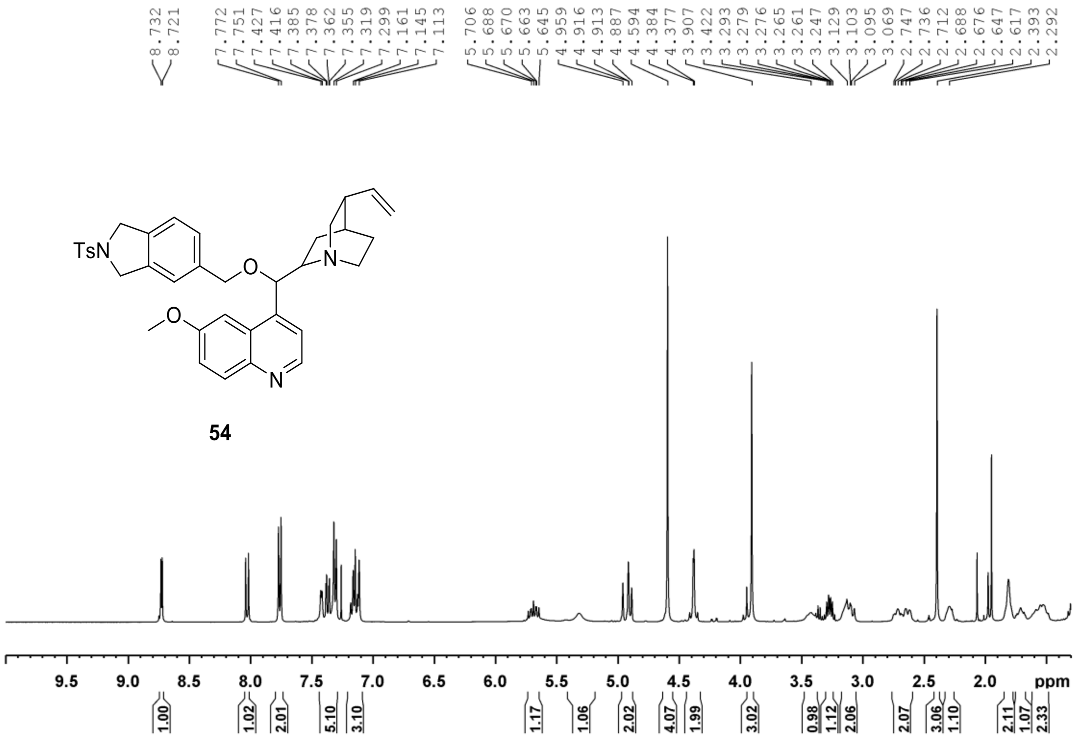

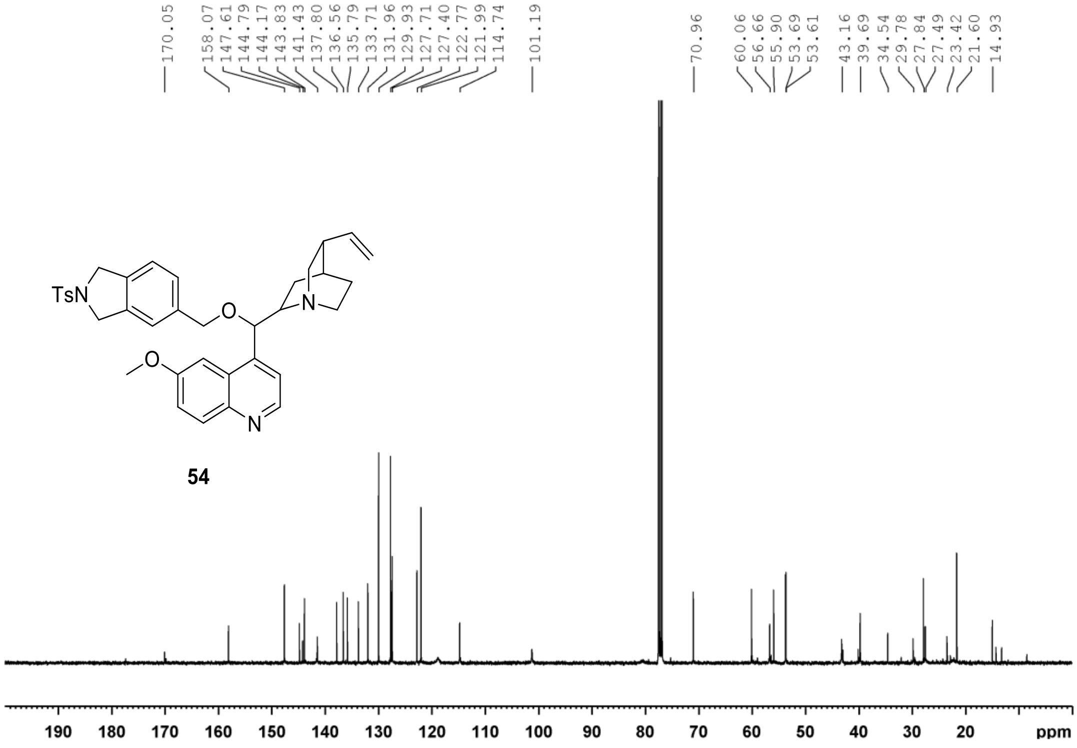

Copies of ^{1}H and ^{13}C NMR spectra of **55**

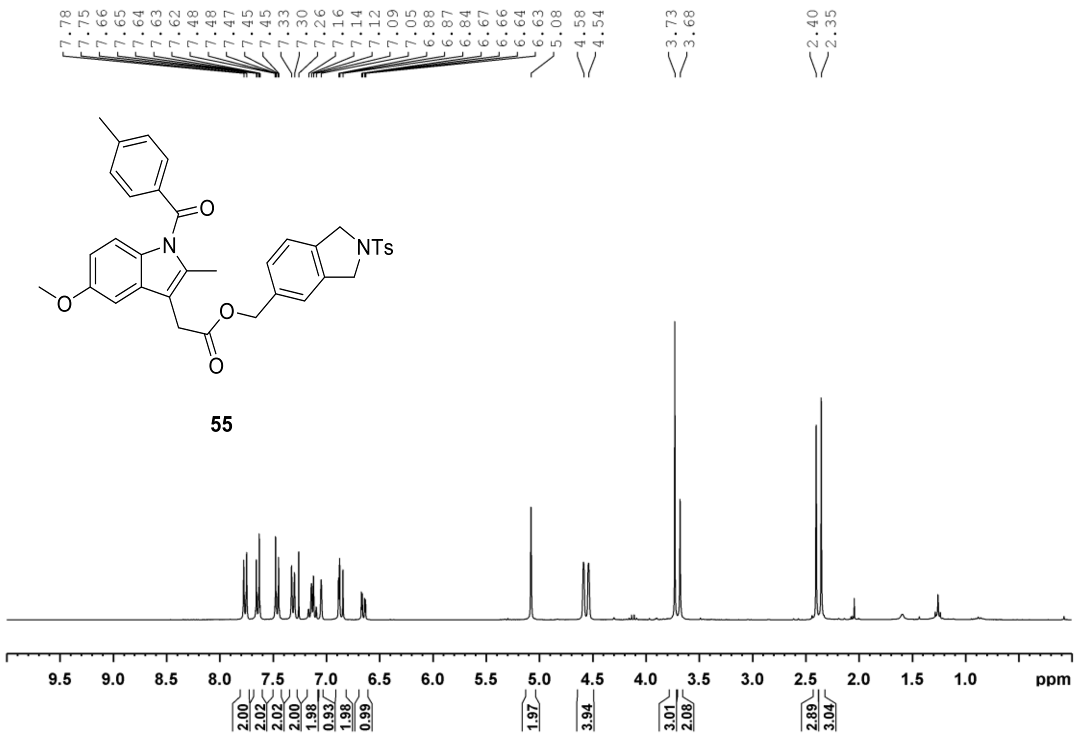

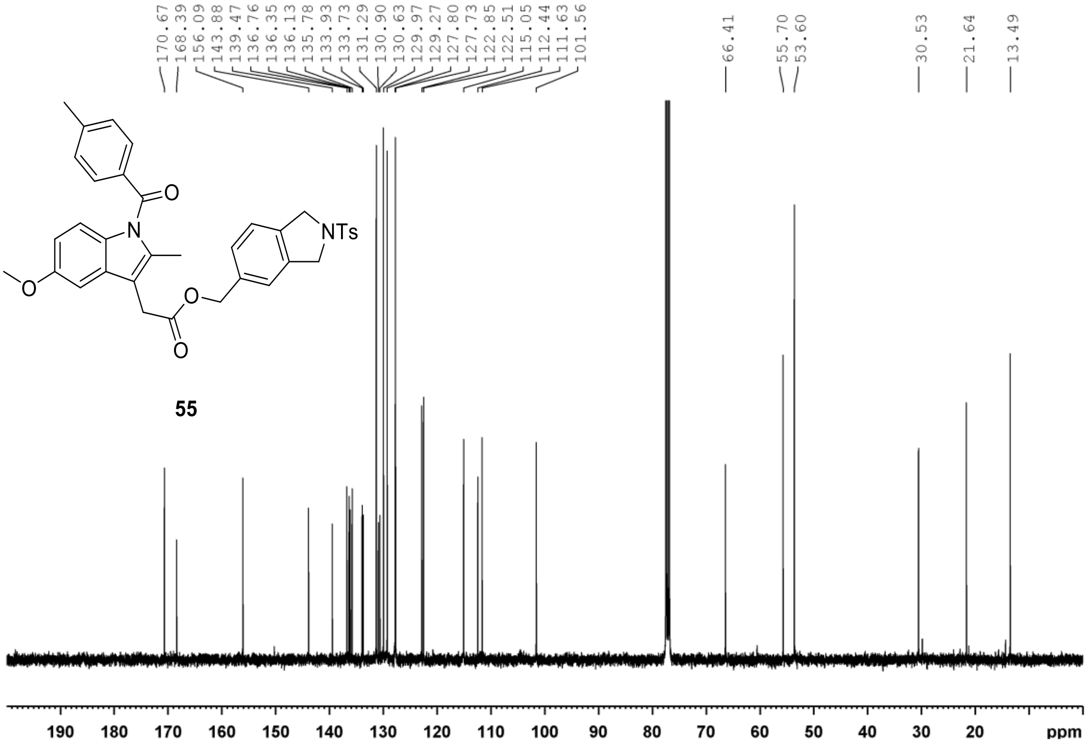

Copies of ^{1}H and ^{13}C NMR spectra of **56**

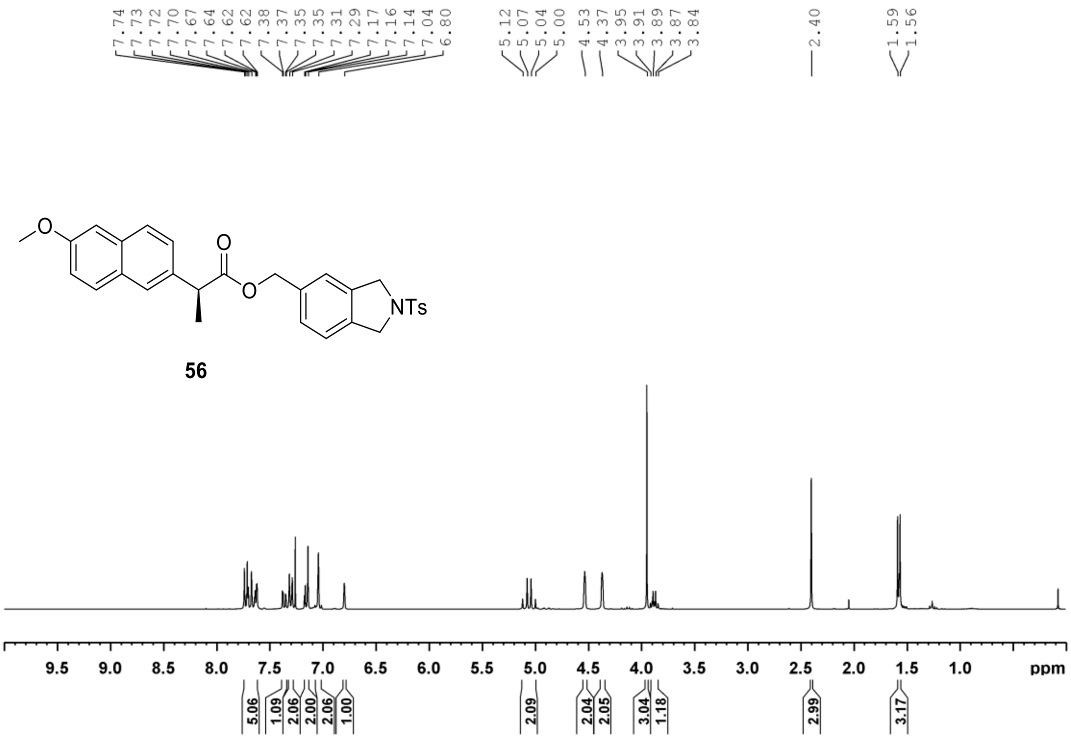

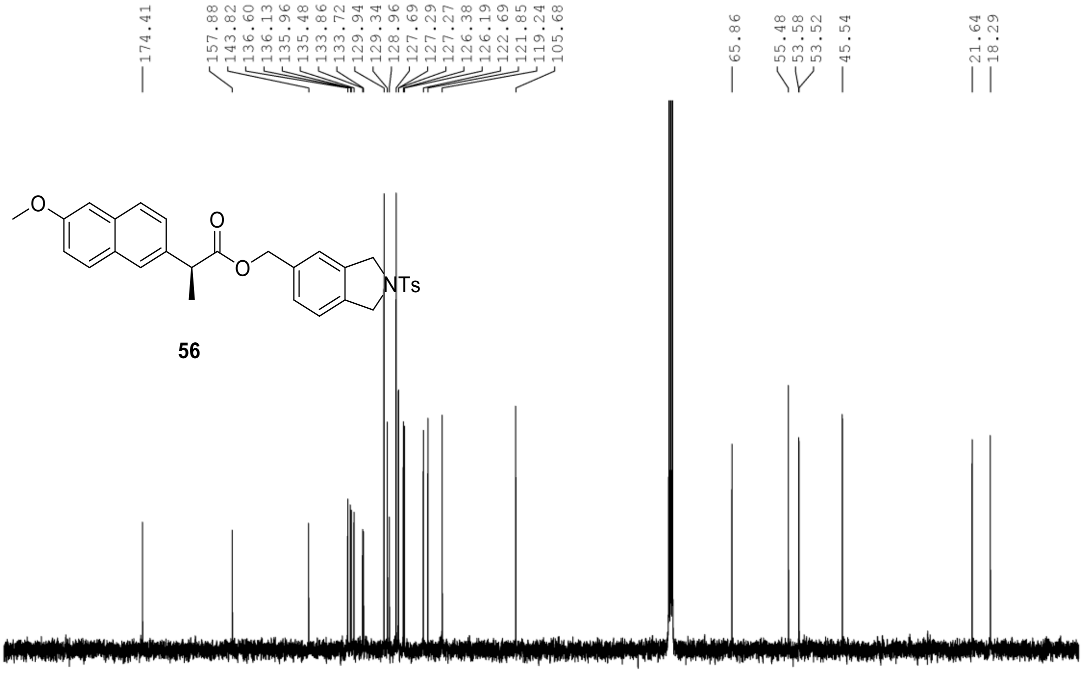

Copies of ^{1}H and ^{13}C NMR spectra of **57**

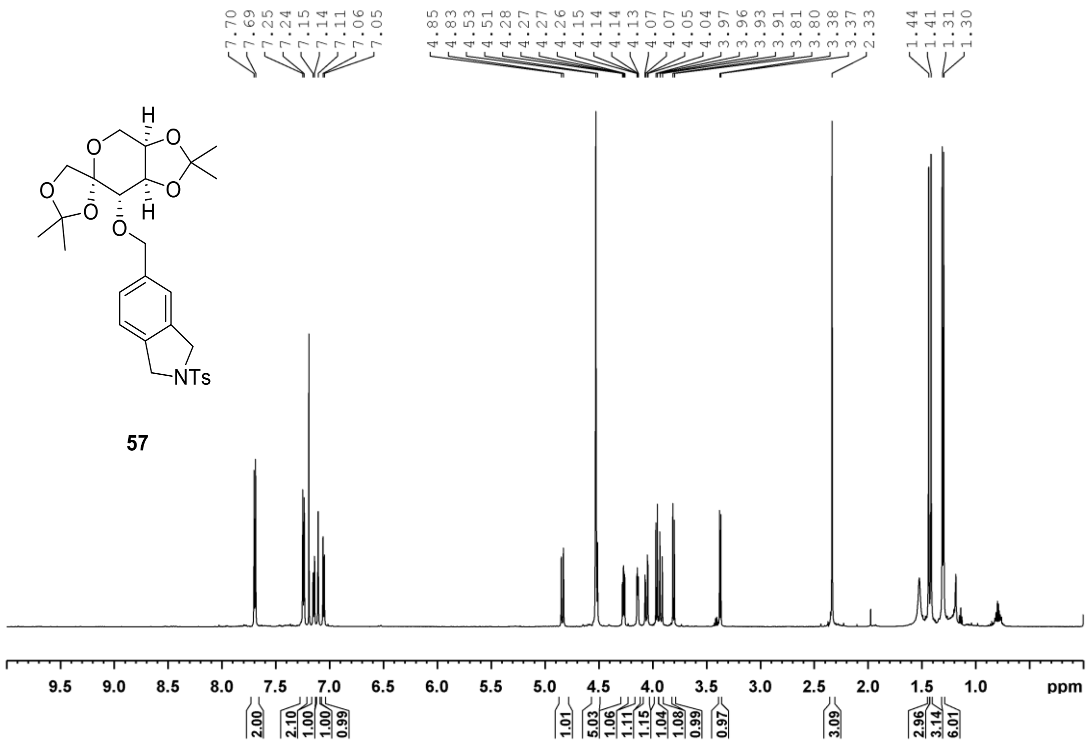

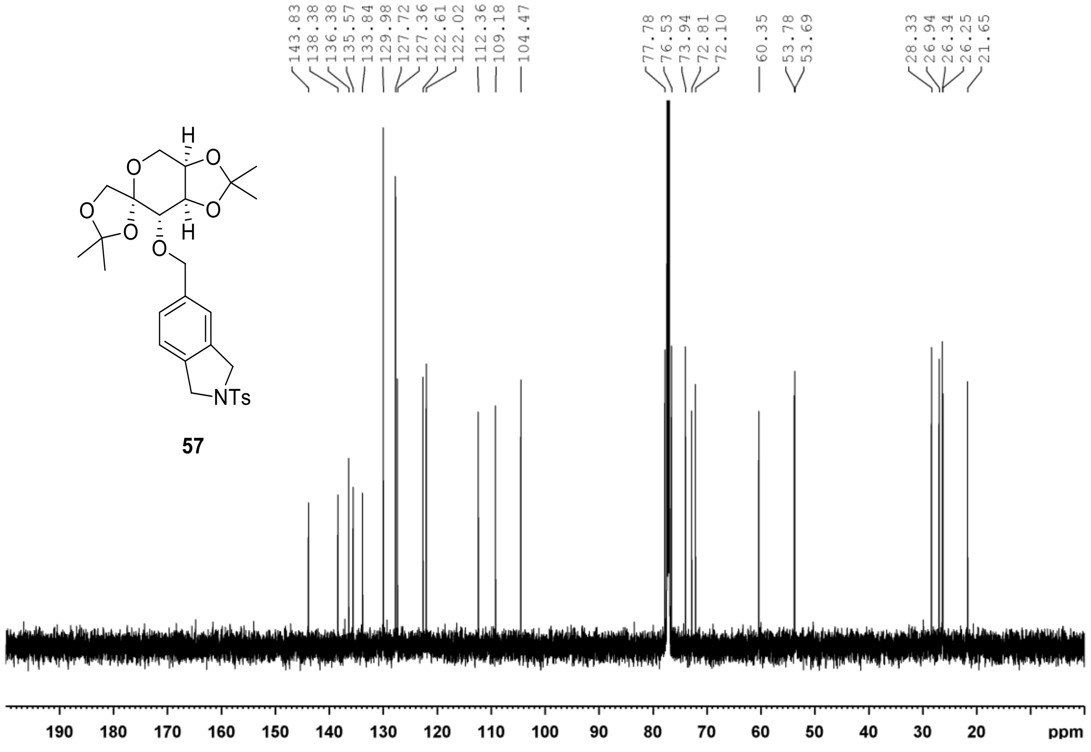

7. X-ray Crystal Data

X-rax Crystal Data of Compound **4**

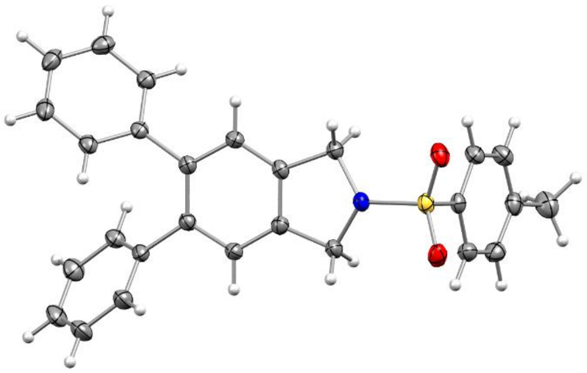

*Table 1: Crystal data and structure refinement for **4** (CCDC 242 4455).*

Empirical formula	$C_{27}H_{23}NO_2S$
Formula weight	425.52
Temperature/K	150(2)
Crystal system	monoclinic
Space group	P 1 21/c 1
a/Å	24.5158(14)
b/Å	10.6327(6)
c/Å	8.3506(5)
α/°	90
β/°	92.461(2)
γ/°	90
Volume/Å^3	2174.7(2)
Z	4
ρ_{calc}/Mg·m^{-3}	1.300
μ/mm^{-1}	0.173
F(000)	896
Crystal size/mm^3	0.238 x 0.300 x 0.321
Wavelength/Å	0.71073
Theta-range for data collection/°	3.10 to 25.40
Limiting indices	-29<=h<=29, -12<=k<=12, -10<=l<=10

Reflections collected/ unique	67229 / 3990 [R(int) = 0.0370]
Data/restraints/parameters	3990 / 0 / 281
Goodness-of-fit on F^2	1.002
Final R indexes [I>2σ (I)]	R1 = 0.0320, wR2 = 0.0863
Final R indexes [all data]	R1 = 0.0335, wR2 = 0.0878
Largest diff. Peak/hole /e $Å^{-3}$	0.239/-0.430

X-rax Crystal Data of Compound **8**

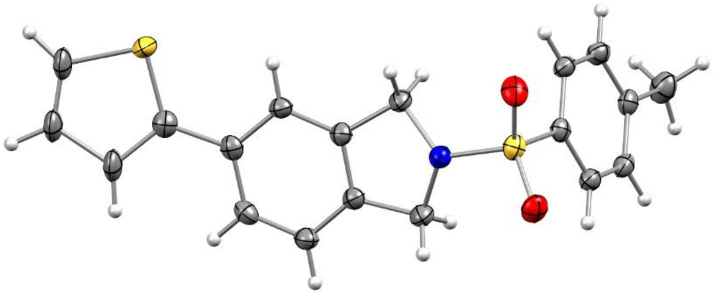

*Table 2: Crystal data and structure refinement for **8** (CCDC 242 4445).*

Empirical formula	$C_{19}H_{17}NO_2S_2$
Formula weight	355.45
Temperature/K	150(2)
Crystal system	monoclinic
Space group	P 1 21 1
a/Å	7.9146(11)
b/Å	5.9945(7)
c/Å	17.597(2)
α/°	90
β/°	97.497(5)
γ/°	90
Volume/Å^3	827.74(19)
Z	2
ρ_{calc}/Mg·m^{-3}	1.426
μ/mm^{-1}	0.333
F(000)	372
Crystal size/mm^3	0.026 x 0.050 x 0.420
Wavelength/Å	0.71073
Theta-range for data collection/°	2.33 to 25.40
Limiting indices	-9<=h<=9, -7<=k<=7, -21<=l<=21
Reflections collected/ unique	28686/ 3059 [R(int) = 0.0616]

Data/restraints/parameters	3059 / 1 / 231
Goodness-of-fit on F^2	1.044
Final R indexes [I>2σ (I)]	R1 = 0.0328, wR2 = 0.0796
Final R indexes [all data]	R1 = 0.0362, wR2 = 0.0822
Largest diff. Peak/hole /e $Å^{-3}$	0.145/-0.287

X-rax Crystal Data of Compound **10**

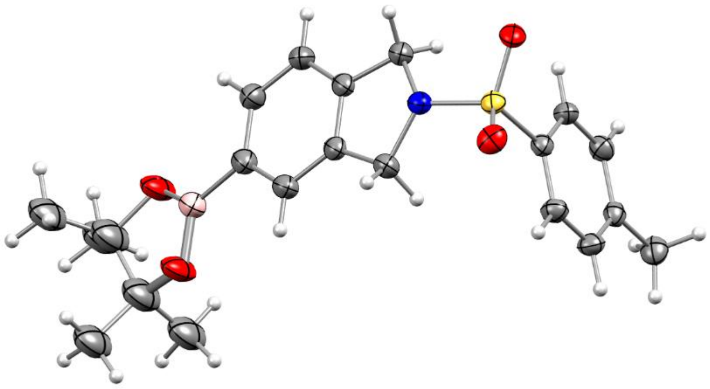

*Table 3: Crystal data and structure refinement for **10** (CCDC 242 4452).*

Empirical formula	$C_{21}H_{26}BNO_4S$
Formula weight	399.30
Temperature/K	150(2)
Crystal system	monoclinic
Space group	P 1 21/c 1
a/Å	16.4307(7)
b/Å	11.3893(6)
c/Å	11.2010(5)
α/°	90
β/°	100.518(2)
γ/°	90
Volume/Å^3	2060.87(17)
Z	4
ρ_{calc}/Mg·m^{-3}	1.287
μ/mm^{-1}	0.184
F(000)	848
Crystal size/mm^3	0.065 x 0.102 x 0.244
Wavelength/Å	0.71073
Theta-range for data collection/°	2.19 to 25.40
Limiting indices	-19<=h<=19, -13<=k<=13, -13<=l<=13

Reflections collected/ unique	97107/ 3784 [R(int) = 0.0563]
Data/restraints/parameters	3784 / 28 / 251
Goodness-of-fit on F^2	0.979
Final R indexes [I>2σ (I)]	R1 = 0.0621, wR2 = 0.1750
Final R indexes [all data]	R1 = 0.0663, wR2 = 0.1810
Largest diff. Peak/hole /e $Å^{-3}$	0.738/-0.683

X-rax Crystal Data of Compound **13**

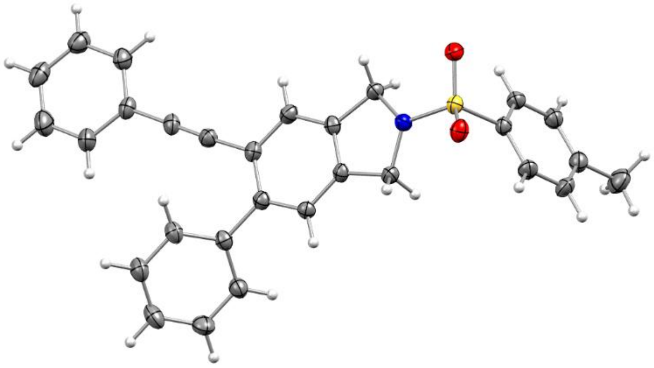

*Table 4: Crystal data and structure refinement for **13** (CCDC 242 4456).*

Empirical formula	$C_{29}H_{23}NO_2S$
Formula weight	449.54
Temperature/K	150(2)
Crystal system	triclinic
Space group	P 1
a/Å	12.7539(10)
b/Å	13.0543(9)
c/Å	14.5363(11)
α/°	76.740(2)
β/°	76.708(3)
γ/°	89.109(2)
Volume/Å^3	2290.8(3)
Z	4
ρ_{calc}/Mg·m^{-3}	1.303
μ/mm^{-1}	0.168
F(000)	944
Crystal size/mm^3	0.062 x 0.169 x 0.544
Wavelength/Å	0.71073
Theta-range for data collection/°	1.91 to 25.40
Limiting indices	-15<=h<=15, -15<=k<=15, -17<=l<=17

Reflections collected/ unique	98055/ 16828 [R(int) = 0.0689]
Data/restraints/parameters	16828 / 3 / 1194
Goodness-of-fit on F^2	1.067
Final R indexes [I>2σ (I)]	R1 = 0.0462, wR2 = 0.1007
Final R indexes [all data]	R1 = 0.0589, wR2 = 0.1099
Largest diff. Peak/hole /e $Å^{-3}$	0.231/-0.344

X-rax Crystal Data of Compound **42**

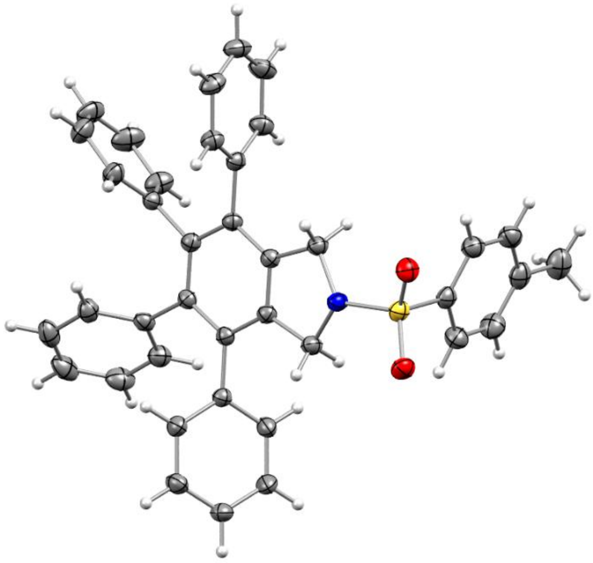

*Table 5: Crystal data and structure refinement for **42** (CCDC 242 4454).*

Empirical formula	$C_{39}H_{31}NO_2S$
Formula weight	577.71
Temperature/K	150(2)
Crystal system	orthorhombic
Space group	P b c a
a/Å	24.1013(10)
b/Å	11.7400(4)
c/Å	43.0477(19)
α/°	90
β/°	90
γ/°	90
Volume/Å^3	12180.3(8)
Z	16
ρ_{calc}/Mg·m^{-3}	1.260
μ/mm^{-1}	0.142
F(000)	4864
Crystal size/mm^3	0.059 x 0.128 x 0.634

Wavelength/Å	0.71073
Theta-range for data collection/°	2.54 to 25.40
Limiting indices	-29<=h<=29, -14<=k<=13, -51<=l<=51
Reflections collected/ unique	178586/ 11181 [R(int) = 0.0615]
Data/restraints/parameters	11181 / 0 / 777
Goodness-of-fit on F^2	1.036
Final R indexes [I>2σ (I)]	R1 = 0.0398, wR2 = 0.1019
Final R indexes [all data]	R1 = 0.0506, wR2 = 0.1126
Largest diff. Peak/hole /e $Å^{-3}$	0.238/-0.461

X-rax Crystal Data of Compound **47**

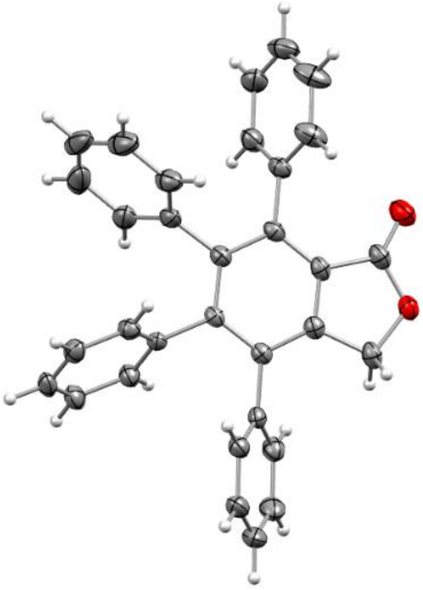

*Table 7: Crystal data and structure refinement for **47** (CCDC 242 4453).*

Empirical formula	$C_{32}H_{22}O_2$
Formula weight	438.49
Temperature/K	150(2)
Crystal system	monoclinic
Space group	C 1 2/c 1
a/Å	34.6314(18)
b/Å	11.4416(6)
c/Å	12.0640(6)
α/°	90
β/°	102.178(2)
γ/°	90
Volume/Å^3	4672.7(4)
Z	8
ρ_{calc}/Mg·m^{-3}	1.247
μ/mm^{-1}	0.076
F(000)	1840
Crystal size/mm^3	0.072 x 0.232 x 0.304
Wavelength/Å	0.71073
Theta-range for data collection/°	2.46 to 25.40
Limiting indices	--41<=h<=41, -13<=k<=13, -14<=l<=14
Reflections collected/ unique	85914/ 4301 [R(int) = 0.0562]

Data/restraints/parameters	4301 / 0 / 307
Goodness-of-fit on F^2	1.035
Final R indexes [I>2σ (I)]	R1 = 0.0513, wR2 = 0.1359
Final R indexes [all data]	R1 = 0.0557, wR2 = 0.1400
Largest diff. Peak/hole /e $Å^{-3}$	1.080/-0.295

X-rax Crystal Data of Compound **48**

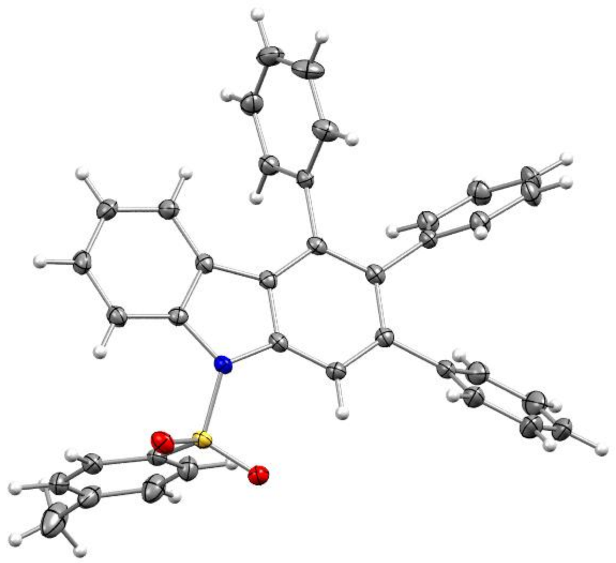

*Table 8: Crystal data and structure refinement for **48** (CCDC 242 4457).*

Empirical formula	$C_{37}H_{27}NO_2S$
Formula weight	549.65
Temperature/K	150(2)
Crystal system	triclinic
Space group	P -1
a/Å	9.7701(7)
b/Å	12.6404(10)
c/Å	12.8796(10)
α/°	63.487(3)
β/°	84.443(3)
γ/°	75.291(3)
Volume/Å^3	1376.43(19)
Z	2
ρ_{calc}/Mg·m^{-3}	1.326
μ/mm^{-1}	0.154
F(000)	576
Crystal size/mm^3	0.133 x 0.138 x 0.189

Wavelength/Å	0.71073
Theta-range for data collection/°	1.92 to 25.39
Limiting indices	-11<=h<=11, -15<=k<=15, -15<=l<=15
Reflections collected/ unique	62849/ 5063 [R(int) = 0.0467]
Data/restraints/parameters	5063 / 0 / 371
Goodness-of-fit on F^2	0.998
Final R indexes [I>2σ (I)]	R1 = 0.0346, wR2 = 0.0896
Final R indexes [all data]	R1 = 0.0381, wR2 = 0.0931
Largest diff. Peak/hole /e $Å^{-3}$	0.318/-0.464

X-rax Crystal Data of Compound **49**

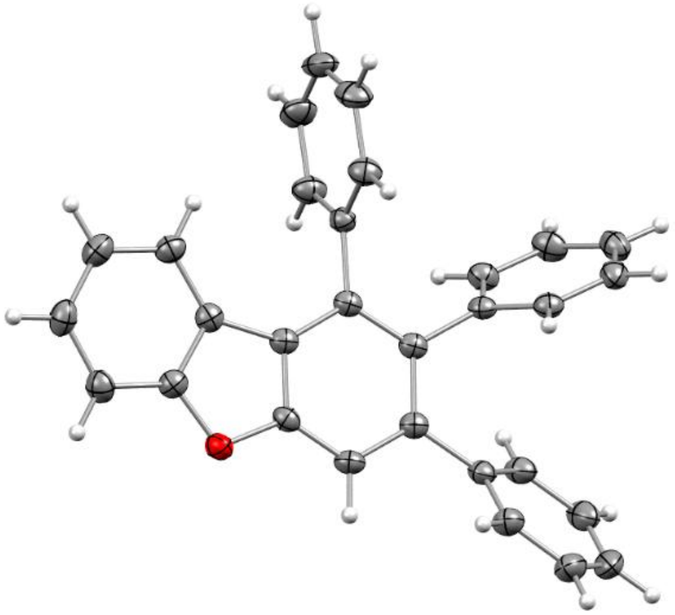

*Table 9: Crystal data and structure refinement for **49** (CCDC 242 4450).*

Empirical formula	$C_{30}H_{20}O$
Formula weight	396.46
Temperature/K	150(2)
Crystal system	triclinic
Space group	P -1
a/Å	9.7072(4)
b/Å	9.8345(5)
c/Å	12.9497(6)
α/°	69.530(2)
β/°	72.893(2)
γ/°	64.774(2)
Volume/Å^3	1032.26(9)
Z	2
ρ_{calc}/Mg·m^{-3}	1.276
μ/mm^{-1}	0.076
F(000)	416
Crystal size/mm^3	0.230 x 0.402 x 0.627
Wavelength/Å	0.71073
Theta-range for data collection/°	2.35 to 25.39

Limiting indices	-11<=h<=11, -11<=k<=11, -15<=l<=15
Reflections collected/ unique	45906/ 3779 [R(int) = 0.0368]
Data/restraints/parameters	3779 / 0 / 280
Goodness-of-fit on F^2	1.096
Final R indexes [I>2σ (I)]	R1 = 0.0383, wR2 = 0.0999
Final R indexes [all data]	R1 = 0.0399, wR2 = 0.1015
Largest diff. Peak/hole /e $Å^{-3}$	0.223/-0.229
